# Supplementary material for: Comparing trends in mid-life ‘deaths of despair’ in the USA, Canada and UK, 2001–2019: is the USA an anomaly?
Source: BMJ Open. 2023 Aug 17;13(8):e069905. doi: 10.1136/bmjopen-2022-069905 (PMC10441077; doi:10.1136/bmjopen-2022-069905)
Supplement: Supplementary data [file bmjopen-2022-069905supp001.pdf]

Supplemental Figures and Tables: Comparing trends in Mid-life "Deaths of Despair" in the US, Canada, and UK, 2001-2019: Is the US an Anomaly? Dowd, et al 2023.

Figure S1 - All-cause mortality in ages 35-64 by sex in Canada, the UK and the USA

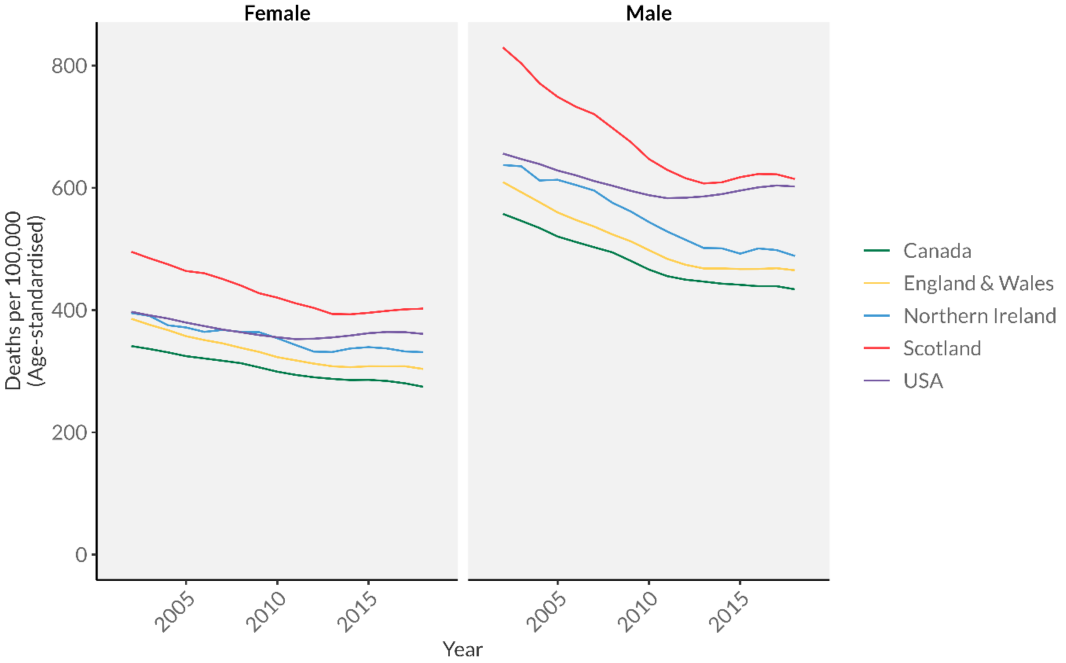

Figure S2 – Combined 'deaths of despair' mortality in ages 35-64 by sex in Canada, the UK and the USA

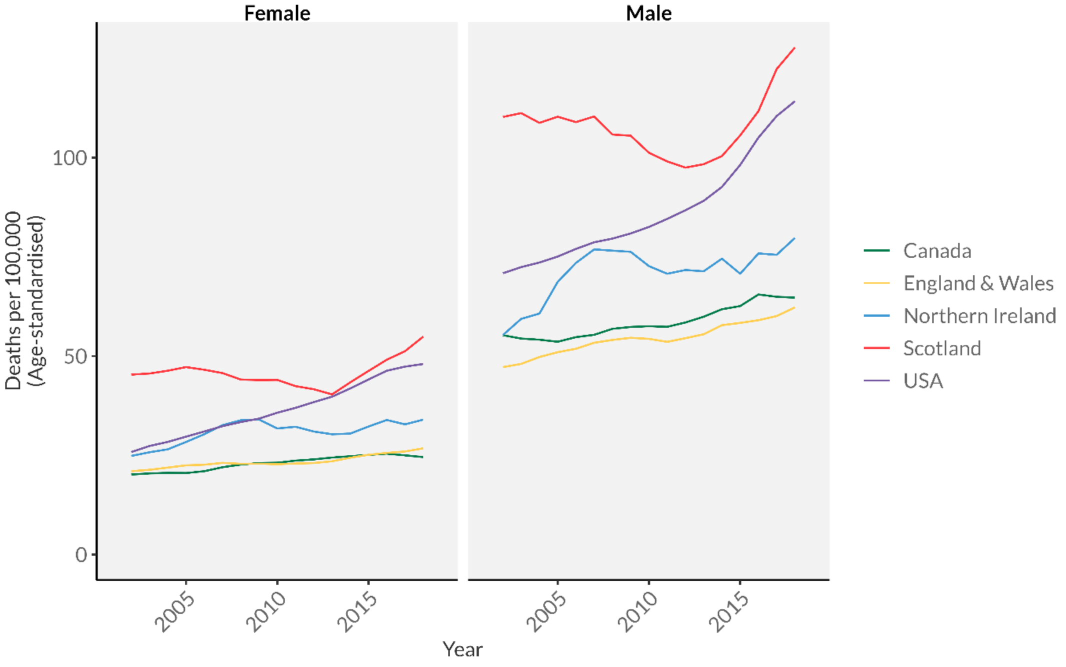

Figure S2a - Drug-related mortality in ages 35-64 by sex in Canada, the UK and the USA

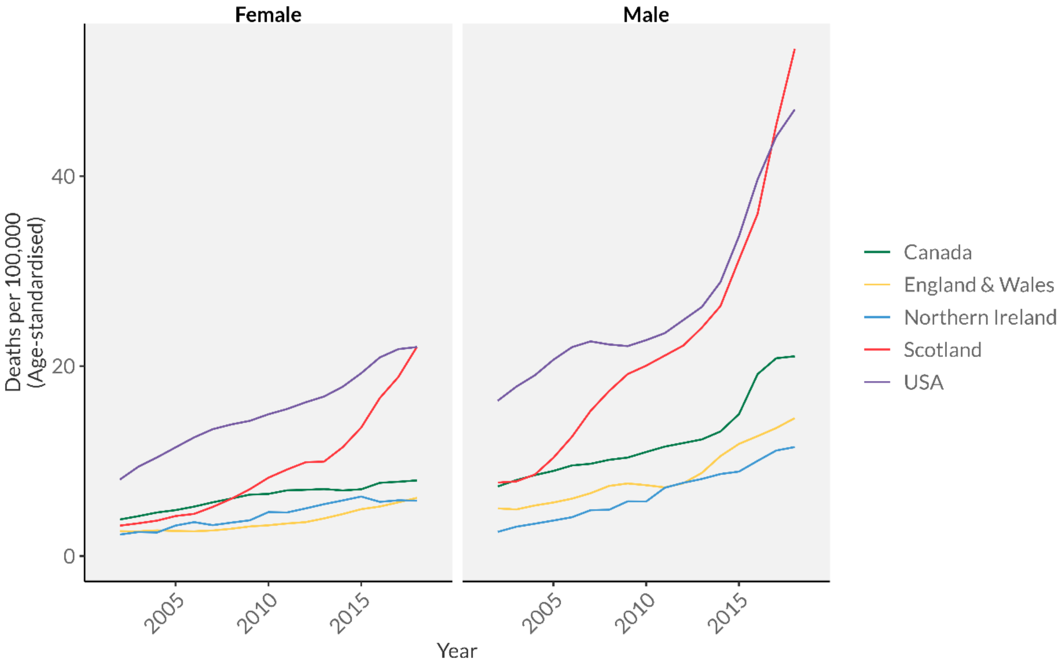

Figure S2b - Suicide mortality in ages 35-64 by sex in Canada, the UK and the USA

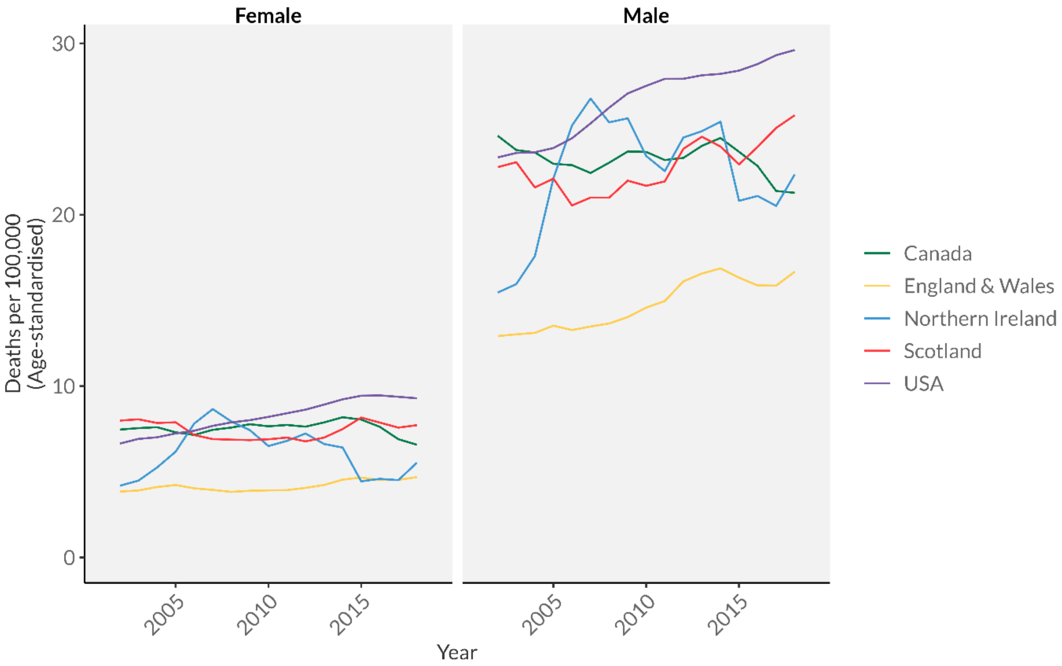

Dowd JB, *et al. BMJ Open* 2023; 13:e069905. doi: 10.1136/bmjopen-2022-069905

Figure S2c – Alcohol-specific mortality in ages 35-64 by sex in Canada, the UK and the USA

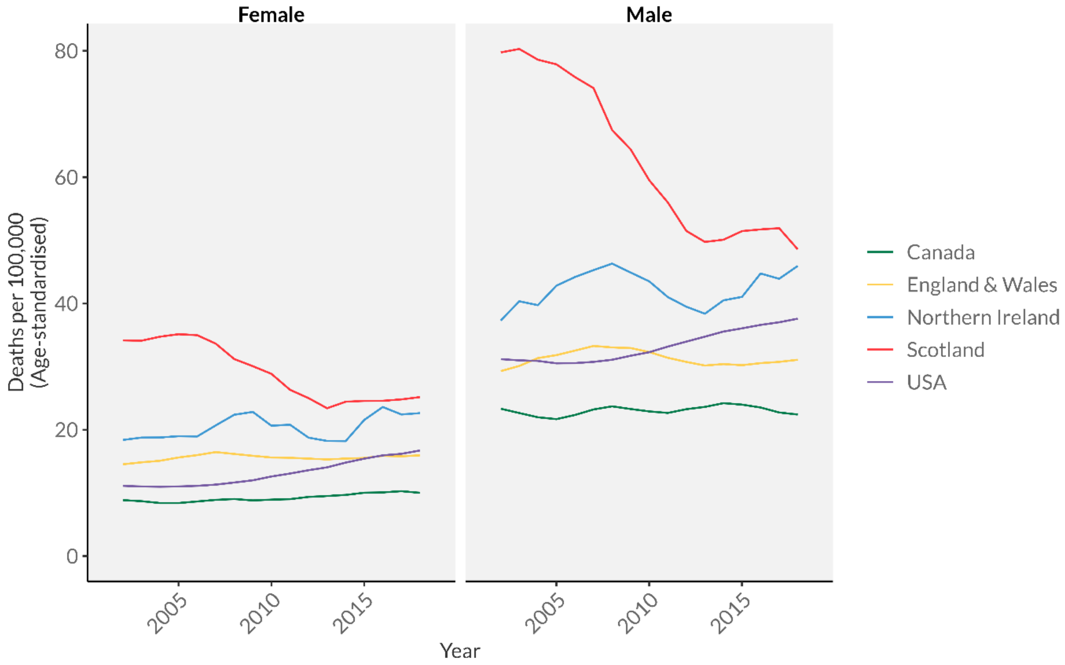

Figure S3 - Mortality from combined 'deaths of despair' by age and sex in Canada, the UK and the US, 2001-2019

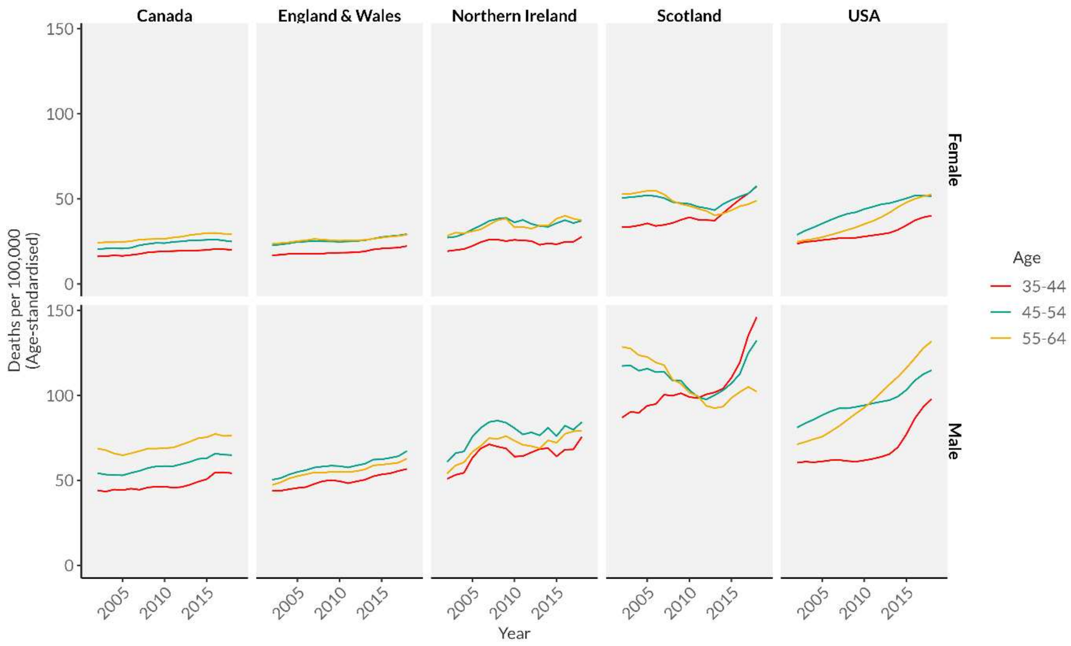

Figure S3a - Drug-related mortality by age and sex in Canada, the UK and the US, 2001-2019

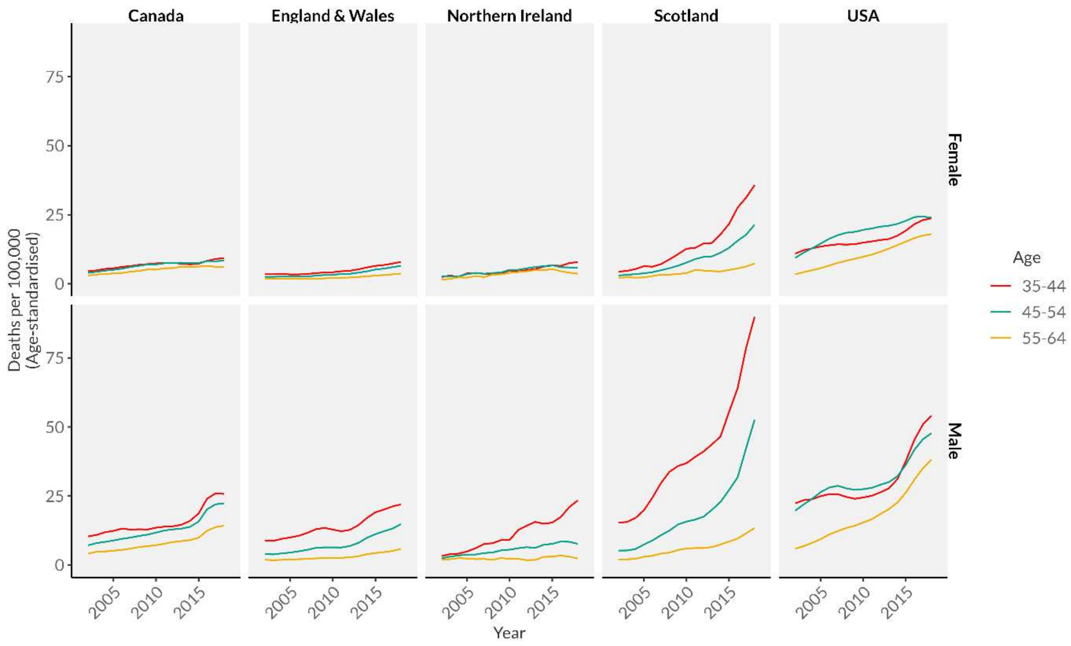

Dowd JB, *et al. BMJ Open* 2023; 13:e069905. doi: 10.1136/bmjopen-2022-069905

Figure S3b - Suicide mortality by age and sex in Canada, the UK and the US, 2001-2019

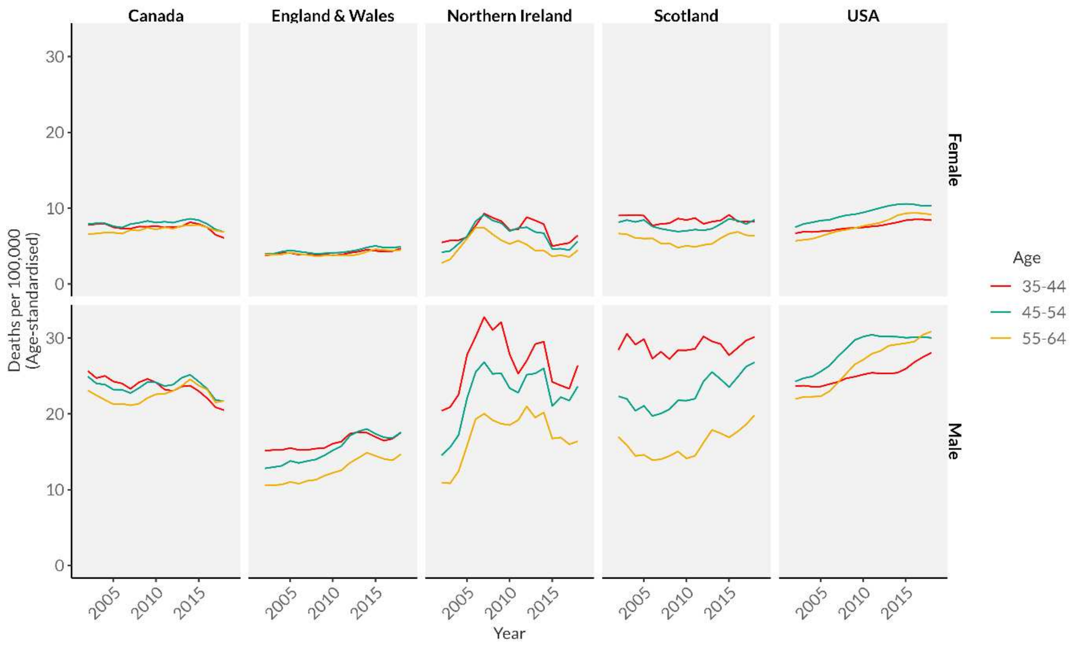

Figure S3c – alcohol-specific mortality by age and sex in Canada, the UK and the US, 2001-2019

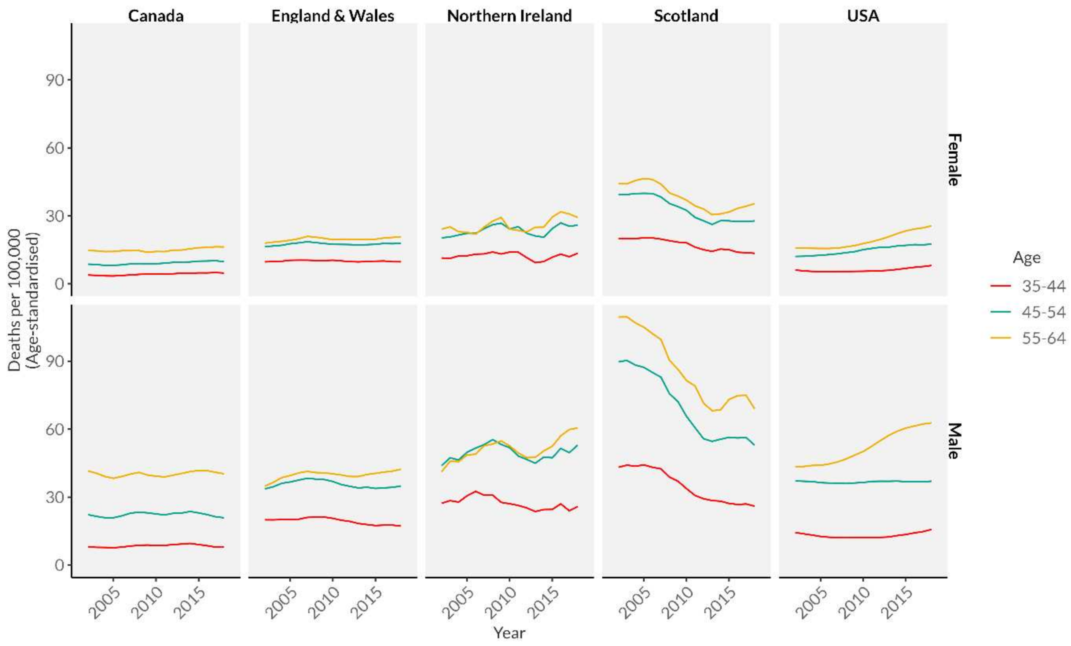

Figure S4a - Lexis surface showing age-specific mortality rates for males by cause, year and country

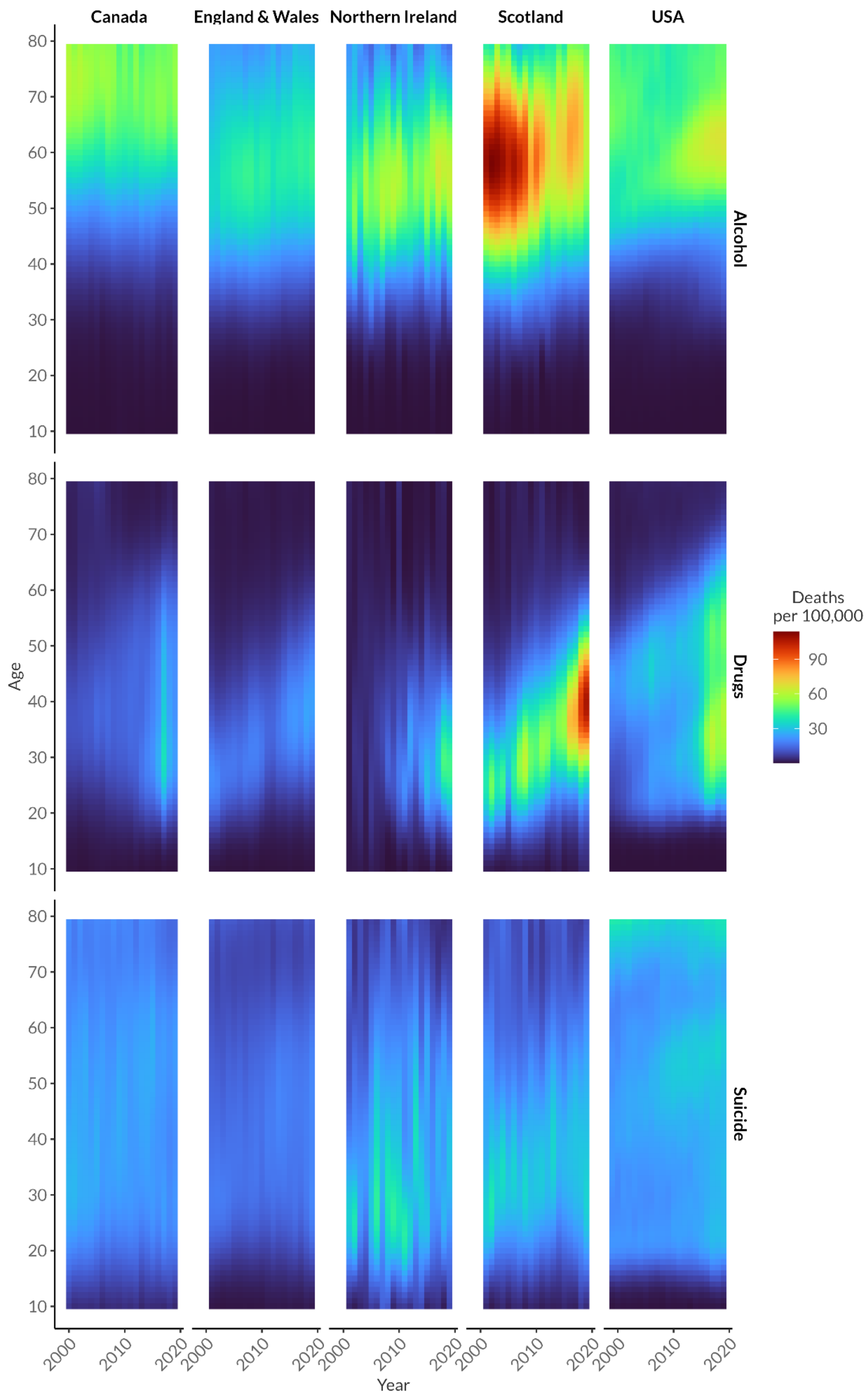

Dowd JB, *et al. BMJ Open* 2023; 13:e069905. doi: 10.1136/bmjopen-2022-069905

Figure S4b - Lexis surface showing age-specific mortality rates for females by cause, year and country

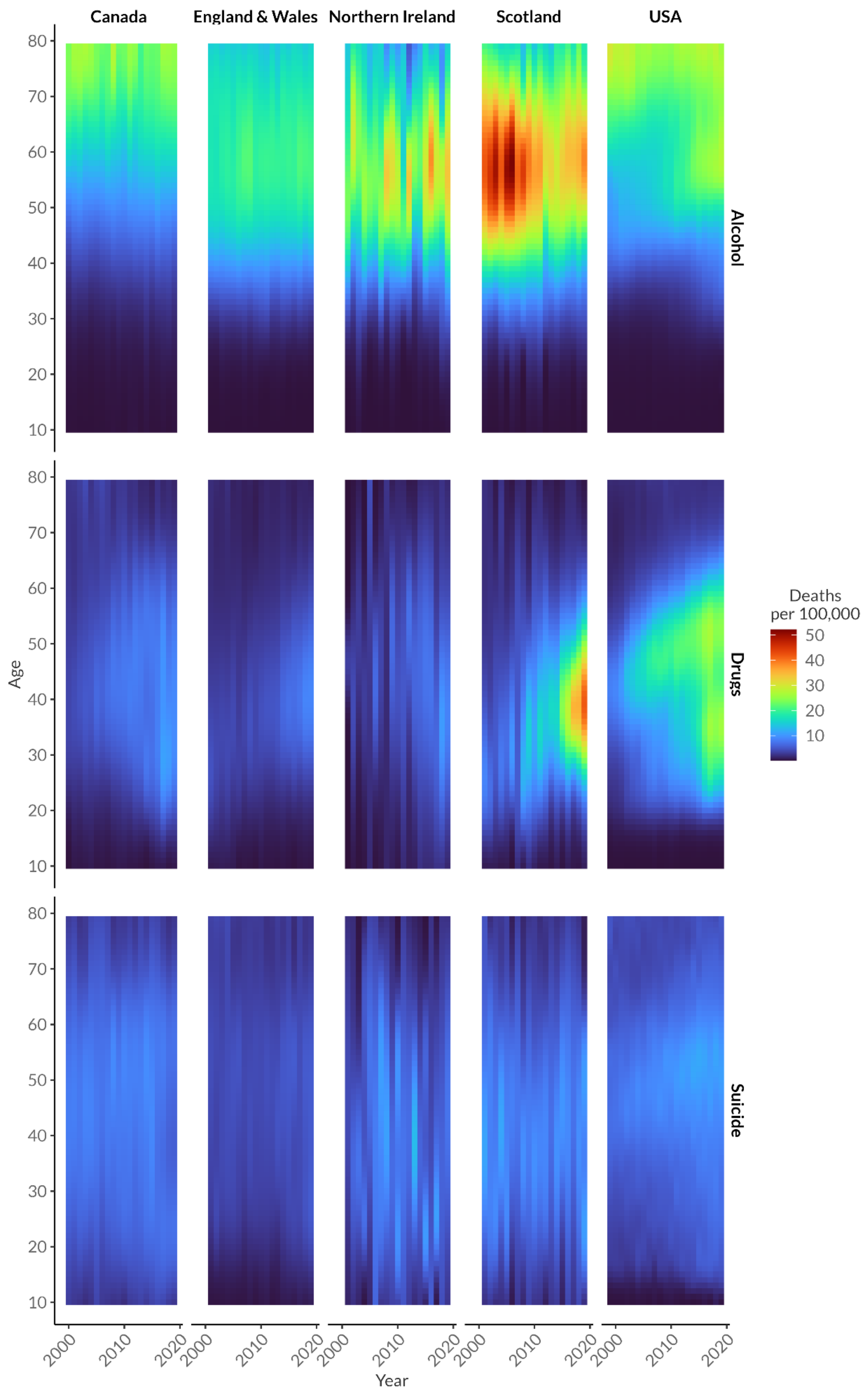

7

Figure S5a - Proportion of all deaths accounted for by alcohol, drugs and suicide among ages 35-44

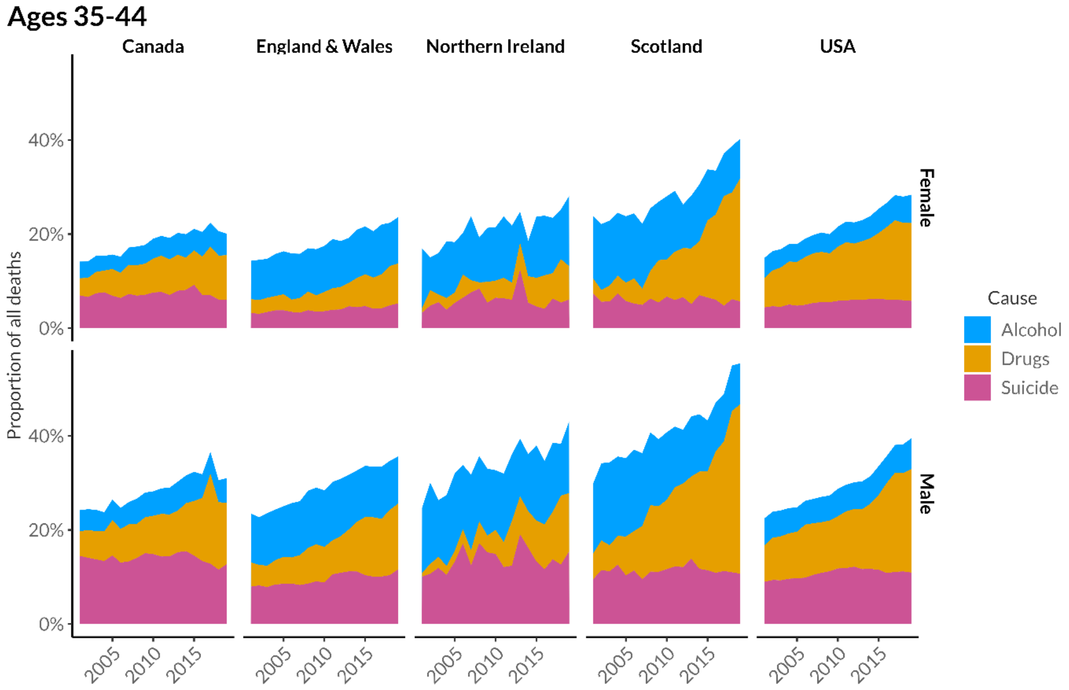

Figure S5b - Proportion of all deaths accounted for by alcohol, drugs and suicide among ages 45-54

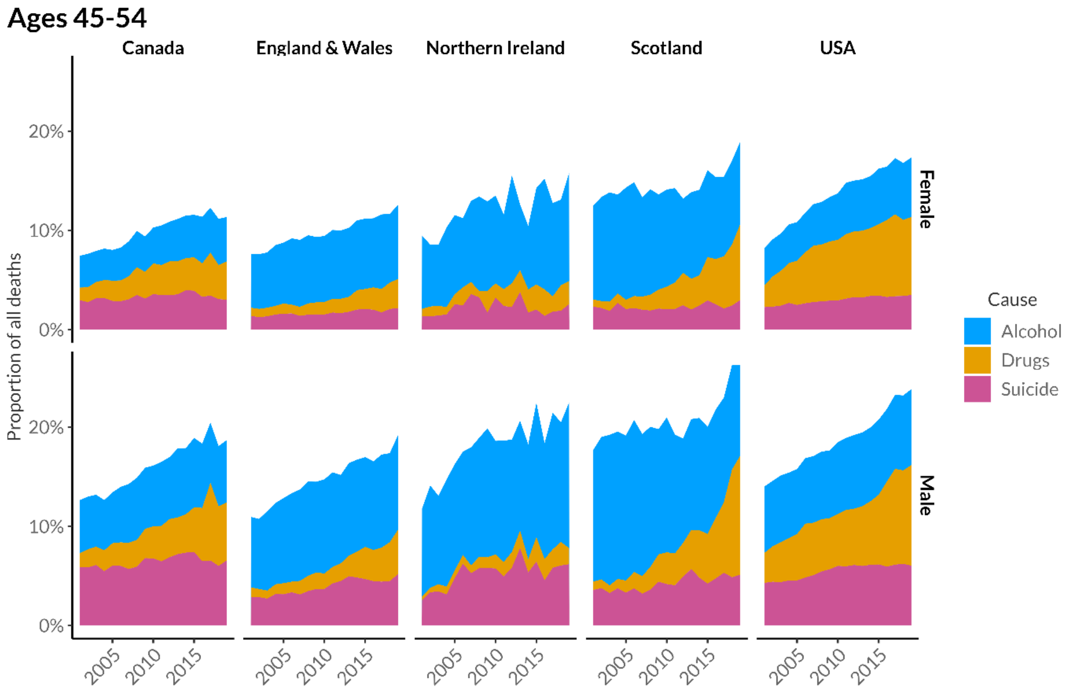

Figure S5c - Proportion of all deaths accounted for by alcohol, drugs and suicide among ages 55-64

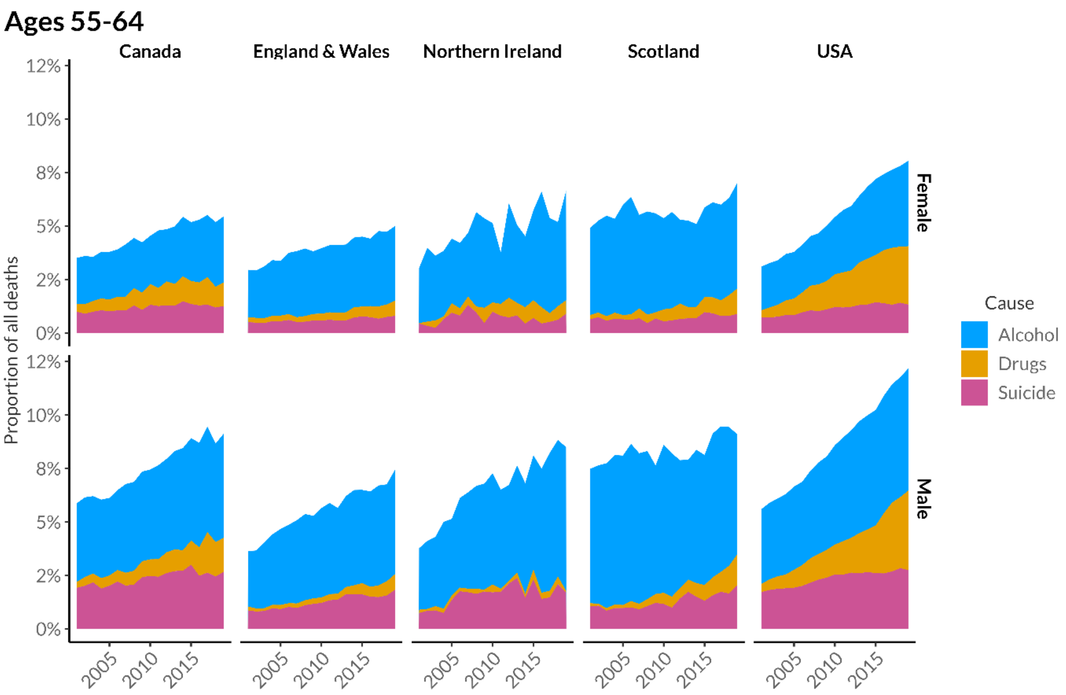

Table S1 - ICD-10 codes used to define 'deaths of despair' categories

**Cause group**	**ICD-10 codes included**
Suicide	U03, X60-84, Y87
Alcohol-related	K70, K73-74, F10, X45, Y15
Drug-related	F11-16, F18-19, X40-44, X85, Y10-14

Dowd JB, *et al. BMJ Open* 2023; 13:e069905. doi: 10.1136/bmjopen-2022-069905

Table S2a - Male mortality rates (per 100,000 population) by cause, age group and year for Canada

Age group	2001	2002	2003	2004	2005	2006	2007	2008	2009	2010	2011	2012	2013	2014	2015	2016	2017	2018	2019
All-Cause																			
35-44	183.6	179.4	182.6	177.9	179.1	174.9	173.3	170.6	169.0	164.1	157.8	157.5	156.2	157.2	158.0	162.4	167.9	167.9	162.0
45-54	424.3	419.3	415.8	404.1	399.2	388.9	388.2	383.5	373.5	361.3	350.2	347.6	347.7	344.4	340.3	344.6	342.0	344.2	334.8
55-64	1156.0	1135.9	1104.2	1075.0	1045.1	1006.9	1010.8	988.8	949.3	917.0	901.3	886.8	884.5	877.6	860.8	863.1	845.6	845.1	825.7
Combined 'Deaths of Despair'																			
35-44	44.4	43.8	44.3	42.3	47.4	43.2	44.9	45.5	47.2	46.3	45.5	45.6	47.3	49.6	51.1	51.6	61.4	51.3	50.1
45-54	53.7	54.5	54.8	51.1	53.6	54.3	55.4	57.1	59.4	58.2	57.8	59.0	62.1	61.6	64.3	63.2	70.0	62.3	62.6
55-64	67.9	69.9	68.6	65.0	64.0	65.4	68.5	68.0	69.8	68.3	69.0	70.5	73.6	74.2	76.8	75.2	80.0	73.3	75.5
Alcohol																			
35-44	8.2	8.0	8.2	7.2	7.9	7.8	8.2	9.1	8.9	8.5	8.6	9.0	9.6	9.2	9.8	8.2	7.7	7.9	8.4
45-54	22.7	22.2	21.8	20.5	20.5	21.7	23.0	24.0	23.0	22.1	22.6	21.7	24.2	23.0	23.8	22.3	20.7	20.9	21.0
55-64	42.5	42.3	39.9	39.5	37.9	37.6	41.9	41.0	39.7	38.5	39.6	38.7	40.7	41.9	41.2	42.2	41.7	38.9	40.2
Drugs																			
35-44	9.7	10.5	11.0	11.2	13.4	12.5	13.7	12.4	12.9	13.4	14.3	14.0	13.8	16.0	18.3	21.8	32.1	24.0	21.1
45-54	6.1	7.7	7.7	8.5	9.0	9.2	10.3	10.3	11.0	11.7	12.5	13.4	12.9	13.3	15.4	18.5	26.8	20.7	19.6
55-64	3.2	4.7	4.6	5.2	5.0	5.4	6.1	6.4	7.1	7.1	7.5	8.6	9.0	8.4	9.8	11.5	16.1	13.6	13.1
Suicide																			
35-44	26.6	25.3	25.1	23.8	26.1	22.9	23.1	24.0	25.4	24.4	22.6	22.6	23.8	24.4	23.0	21.7	21.5	19.4	20.6
45-54	24.9	24.5	25.4	22.1	24.1	23.4	22.1	22.8	25.4	24.4	22.6	23.9	25.0	25.3	25.2	22.3	22.4	20.7	22.0
55-64	22.3	22.9	24.1	20.3	21.1	22.3	20.5	20.5	23.0	22.7	22.0	23.2	23.9	23.9	25.9	21.5	22.3	20.7	22.1

Table S2b - Female mortality rates (per 100,000 population) by cause, age group and year in Canada

Age group	2001	2002	2003	2004	2005	2006	2007	2008	2009	2010	2011	2012	2013	2014	2015	2016	2017	2018	2019
All-Cause																			
35-44	112.1	111.7	110.5	106.9	108.8	106.3	105.2	108.2	107.3	100.2	100.6	100.8	96.3	98.9	95.4	98.6	96.1	95.5	94.6
45-54	268.6	268.4	264.8	257.6	257.2	251.8	252.1	251.8	246.4	235.9	234.3	232.9	226.2	227.2	223.4	227.3	219.6	213.2	212.6
55-64	685.8	685.2	672.6	657.5	647.5	631.1	629.3	617.5	597.5	586.7	576.6	569.2	564.0	560.8	566.1	564.0	555.1	540.0	530.0
Combined 'Deaths of Despair'																			
35-44	15.9	15.8	17.0	16.4	17.0	16.1	18.0	18.8	19.0	19.1	19.7	19.3	19.5	19.7	20.1	20.2	21.5	19.7	19.0
45-54	20.0	20.5	21.0	21.0	20.7	20.8	22.4	25.1	23.1	24.3	24.6	25.3	25.2	26.1	25.8	25.9	27.0	23.8	24.1
55-64	24.1	24.7	24.0	25.0	24.6	24.7	26.1	27.5	25.4	26.7	27.7	27.6	28.1	30.5	29.4	29.9	30.7	28.0	28.9
Alcohol																			
35-44	4.0	3.9	3.8	3.3	3.3	3.7	3.9	4.3	4.3	4.2	4.2	4.6	4.5	4.9	4.3	5.2	4.8	5.0	4.3
45-54	8.6	9.0	8.3	8.2	8.0	8.3	8.9	9.2	8.8	8.5	9.4	9.3	9.6	9.8	9.5	10.7	9.9	9.9	9.5
55-64	14.7	15.4	13.9	14.3	14.4	14.0	15.4	14.4	14.1	13.2	15.5	13.9	15.2	15.6	15.6	16.5	16.1	16.3	16.4
Drugs																			
35-44	4.1	4.5	5.0	4.9	6.2	5.7	6.5	7.0	7.1	7.2	7.8	7.6	7.4	6.8	7.0	8.1	9.9	8.9	9.0
45-54	3.4	4.0	4.4	4.7	5.3	5.3	5.9	7.1	6.6	7.3	7.1	7.9	7.6	7.3	7.6	7.7	9.5	7.3	8.2
55-64	2.5	3.1	3.4	3.6	3.6	4.0	4.1	5.0	4.8	5.7	4.9	6.3	5.8	6.5	6.1	6.1	7.1	5.3	5.8
Suicide																			
35-44	7.8	7.4	8.2	8.1	7.5	6.8	7.6	7.5	7.6	7.6	7.7	7.1	7.6	8.0	8.8	6.9	6.7	5.8	5.7
45-54	8.0	7.4	8.4	8.2	7.4	7.2	7.7	8.8	7.7	8.4	8.1	8.1	8.1	9.0	8.7	7.5	7.6	6.5	6.5
55-64	6.9	6.2	6.7	7.0	6.6	6.7	6.6	8.0	6.5	7.8	7.3	7.4	7.2	8.3	7.7	7.3	7.4	6.5	6.7

Dowd JB, *et al. BMJ Open* 2023; 13:e069905. doi: 10.1136/bmjopen-2022-069905

Table S3a - Male mortality rates (per 100,000 population) by cause, age group and year for England & Wales

Age group	2001	2002	2003	2004	2005	2006	2007	2008	2009	2010	2011	2012	2013	2014	2015	2016	2017	2018	2019
All-Cause																			
35-44	190.7	188.3	188.4	183.7	181.3	181.5	177.1	179.8	177.7	168.9	162.8	155.2	161.4	159.5	160.7	162.9	161.7	168.0	162.8
45-54	461.4	453.4	451.1	433.0	428.0	424.2	412.7	410.8	402.8	393.9	382.2	368.3	375.6	370.5	372.9	374.7	374.4	382.0	371.0
55-64	1280.1	1251.8	1226.2	1159.3	1129.0	1100.6	1070.2	1044.7	1003.7	991.9	954.9	929.1	926.8	913.1	917.4	916.9	907.3	910.9	888.6
Combined 'Deaths of Despair'																			
35-44	44.8	42.8	44.4	44.7	45.4	46.7	46.2	51.0	51.5	48.0	49.2	47.9	51.2	52.1	54.1	54.5	54.1	58.3	58.0
45-54	50.5	48.7	52.0	53.5	55.0	56.6	56.6	59.6	58.4	58.0	59.0	55.9	61.5	62.0	63.3	62.0	64.5	66.4	71.1
55-64	46.6	45.9	49.5	51.3	52.8	53.6	54.4	56.1	53.1	56.0	56.3	52.5	57.6	59.3	59.6	58.9	60.9	61.6	66.1
Alcohol																			
35-44	19.8	19.1	21.0	19.7	19.5	21.0	20.3	21.9	21.4	20.3	20.2	18.9	18.7	17.4	17.5	17.5	17.9	17.5	16.3
45-54	32.7	32.0	36.1	35.5	36.7	37.9	38.0	39.0	36.9	37.4	36.4	32.8	35.0	34.3	33.7	33.5	35.1	34.3	35.3
55-64	33.1	33.8	37.5	38.2	40.1	40.3	41.6	42.1	38.7	41.4	40.7	37.2	39.4	40.6	40.0	40.8	42.3	41.3	43.3
Drugs																			
35-44	9.8	8.2	8.6	9.5	10.4	10.1	11.3	13.6	14.0	12.8	11.8	12.1	14.4	16.9	19.9	20.6	19.9	23.3	22.8
45-54	4.6	3.7	3.6	4.3	4.8	4.5	5.6	6.4	6.7	6.1	6.3	6.6	7.8	9.7	12.1	11.8	12.9	15.0	16.6
55-64	2.3	1.8	1.6	1.9	2.2	1.9	2.3	2.4	2.6	2.5	2.8	2.6	3.1	3.9	4.8	4.3	5.0	6.0	6.5
Suicide																			
35-44	15.2	15.4	14.8	15.5	15.4	15.6	14.7	15.5	16.1	14.9	17.2	16.9	18.1	17.8	16.7	16.4	16.3	17.4	18.9
45-54	13.2	13.0	12.3	13.7	13.5	14.2	12.9	14.2	14.8	14.5	16.2	16.5	18.7	17.9	17.5	16.7	16.5	17.1	19.1
55-64	11.1	10.3	10.3	11.2	10.5	11.4	10.5	11.6	11.8	12.1	12.8	12.8	15.1	14.7	14.8	13.8	13.5	14.3	16.3

Dowd JB, *et al. BMJ Open* 2023; 13:e069905. doi: 10.1136/bmjopen-2022-069905

Table S3b - Female mortality rates (per 100,000 population) by cause, age group and year in England & Wales

Age group	2001	2002	2003	2004	2005	2006	2007	2008	2009	2010	2011	2012	2013	2014	2015	2016	2017	2018	2019
All-Cause																			
35-44	117.5	115.0	116.4	112.2	111.6	110.4	109.3	110.2	105.6	104.8	100.9	97.4	97.8	98.8	99.0	99.3	97.8	99.2	98.7
45-54	302.5	297.0	295.1	282.4	281.3	276.6	273.2	272.8	262.3	260.4	253.5	249.5	246.7	244.2	247.4	251.2	245.3	248.1	243.0
55-64	802.5	784.8	770.3	735.2	722.1	700.6	687.0	680.8	651.2	641.2	629.7	623.5	612.7	600.7	608.3	621.5	599.8	608.3	587.8
Combined 'Deaths of Despair'																			
35-44	16.8	16.7	17.1	17.7	18.2	17.5	17.3	18.7	17.7	18.3	19.1	18.0	18.8	20.7	21.4	20.4	21.6	22.2	23.2
45-54	23.0	22.6	23.0	24.1	24.7	25.5	24.7	26.0	24.5	24.6	25.5	24.9	25.3	26.9	27.7	28.2	28.5	28.9	30.5
55-64	23.7	23.0	24.0	25.1	24.3	26.3	26.3	26.9	24.9	25.4	25.9	25.6	25.4	26.8	27.4	27.4	28.6	28.8	29.4
Alcohol																			
35-44	9.6	9.8	9.6	10.0	10.2	10.8	10.2	10.2	10.4	10.2	10.6	9.5	9.4	10.1	10.1	9.8	10.4	9.1	9.7
45-54	16.4	16.4	16.4	17.3	17.3	18.5	18.4	18.8	17.3	17.3	17.6	17.2	17.2	17.2	17.5	17.5	18.5	17.2	18.2
55-64	17.7	17.4	18.7	19.1	18.4	20.1	21.2	21.5	19.0	19.6	19.7	19.7	19.4	19.6	19.9	19.6	21.1	20.6	20.5
Drugs																			
35-44	3.5	3.4	3.5	3.4	3.8	2.9	3.4	4.4	3.7	4.3	4.6	4.7	4.9	6.2	6.7	6.6	7.1	8.3	8.4
45-54	2.5	2.5	2.6	2.5	2.9	2.4	2.5	3.1	3.2	3.3	3.5	3.6	3.7	4.8	5.0	5.7	5.8	6.6	7.1
55-64	1.9	1.8	1.8	1.8	1.9	1.9	1.6	1.8	2.0	2.1	2.2	2.2	2.3	2.9	2.8	3.2	3.4	3.5	4.2
Suicide																			
35-44	3.8	3.5	4.0	4.2	4.2	3.8	3.6	4.2	3.7	3.8	3.9	3.9	4.5	4.4	4.6	4.1	4.1	4.8	5.2
45-54	4.2	3.7	4.0	4.3	4.5	4.5	3.8	4.1	4.0	4.0	4.4	4.1	4.4	4.9	5.1	5.1	4.3	5.2	5.3
55-64	4.1	3.8	3.6	4.1	4.0	4.3	3.6	3.6	3.8	3.7	3.9	3.7	3.6	4.4	4.8	4.6	4.0	4.6	4.7

Dowd JB, *et al. BMJ Open* 2023; 13:e069905. doi: 10.1136/bmjopen-2022-069905

Table S4a - Male mortality rates (per 100,000 population) by cause, age group and year for Northern Ireland

Age group	2001	2002	2003	2004	2005	2006	2007	2008	2009	2010	2011	2012	2013	2014	2015	2016	2017	2018	2019
All-Cause																			
35-44	192.7	200.4	170.8	199.3	199.3	211.6	223.7	199.2	204.4	207.7	177.2	189.6	188.9	173.4	184.3	172.7	193.7	183.7	190.4
45-54	465.7	497.4	438.8	479.3	450.5	475.5	480.3	440.9	431.9	443.7	396.4	398.5	417.9	375.3	393.1	388.9	405.0	395.8	378.1
55-64	1324.3	1385.5	1282.8	1282.1	1214.4	1205.1	1170.8	1137.3	1067.7	1092.9	1042.6	971.1	1011.6	940.0	977.1	977.9	967.5	947.7	876.9
Combined 'Deaths of Despair'																			
35-44	47.6	60.2	45.0	54.7	63.8	71.6	71.1	71.1	67.5	67.9	56.7	68.4	74.4	62.6	70.0	59.9	74.7	70.4	81.9
45-54	54.9	70.2	57.4	70.5	73.3	83.4	86.3	83.5	85.9	82.6	73.9	74.7	86.3	68.5	88.3	71.5	86.9	81.0	85.0
55-64	50.0	57.0	55.2	64.2	62.5	73.8	74.8	76.1	72.6	79.6	67.9	65.3	77.3	64.1	79.3	73.3	79.6	83.7	74.5
Alcohol																			
35-44	26.7	34.7	20.5	30.1	32.8	28.9	35.9	27.6	29.1	26.3	25.8	26.8	23.1	21.0	29.4	23.3	28.4	20.2	29.0
45-54	41.2	51.3	39.1	51.7	48.3	49.5	57.1	52.9	56.2	50.8	48.5	45.2	46.3	43.4	53.3	45.4	55.8	47.7	55.5
55-64	38.1	44.0	41.5	52.0	43.5	50.5	52.7	54.7	52.8	56.8	48.2	43.4	50.6	48.5	52.1	56.7	62.3	60.5	58.8
Drugs																			
35-44	1.6	4.1	4.1	3.7	4.6	6.5	7.2	9.2	7.3	10.6	9.4	18.0	15.3	13.6	16.1	16.5	19.6	26.9	23.6
45-54	1.8	2.2	3.2	3.8	3.5	4.2	3.8	5.1	4.7	6.3	5.8	6.3	7.4	5.0	9.7	8.3	7.5	9.4	6.2
55-64	2.0	1.2	2.5	2.6	2.4	2.0	2.0	2.6	1.0	4.1	1.5	1.2	2.6	1.7	4.6	2.8	3.0	3.4	0.8
Suicide																			
35-44	19.4	21.4	20.4	20.8	26.4	36.2	27.9	34.2	31.1	31.0	21.4	23.5	36.0	28.1	24.5	20.1	26.6	23.2	29.3
45-54	11.9	16.7	15.0	15.0	21.5	29.7	25.3	25.5	25.0	25.5	19.6	23.3	32.6	20.1	25.3	17.7	23.6	24.0	23.4
55-64	9.9	11.8	11.2	9.6	16.6	21.3	20.1	18.7	18.7	18.7	18.2	20.6	24.1	13.8	22.6	13.8	14.3	19.9	15.0

Table S4b - Female mortality rates (per 100,000 population) by cause, age group and year in Northern Ireland

Age group	2001	2002	2003	2004	2005	2006	2007	2008	2009	2010	2011	2012	2013	2014	2015	2016	2017	2018	2019
All-Cause																			
35-44	119.4	121.7	121.7	119.8	110.2	122.8	121.5	127.5	116.4	122.3	112.9	108.8	102.3	107.8	111.8	98.3	103.7	105.9	115.0
45-54	310.2	311.2	301.8	300.3	275.8	298.1	292.2	297.0	288.4	292.7	274.7	267.2	259.3	267.3	281.2	257.1	261.0	267.6	274.0
55-64	809.9	819.0	772.8	774.6	722.8	740.3	711.3	710.9	727.4	704.9	656.3	652.0	659.5	663.0	689.5	688.4	654.1	663.1	639.7
Combined 'Deaths of Despair'																			
35-44	20.1	18.3	19.4	22.1	20.1	24.8	28.9	24.6	24.8	26.3	26.8	23.7	25.3	20.0	26.5	23.5	24.3	26.7	32.3
45-54	29.4	26.6	25.8	30.9	31.8	33.5	37.9	39.8	37.3	39.6	31.8	41.5	32.7	27.9	40.2	39.1	33.3	35.0	43.3
55-64	24.6	32.6	28.0	29.8	31.9	31.2	33.3	40.2	39.0	36.3	24.7	39.6	33.3	30.1	39.6	45.6	35.2	34.4	42.5
Alcohol																			
35-44	15.0	8.5	10.7	14.4	11.8	10.9	16.6	12.2	13.4	13.9	14.7	13.2	6.7	8.1	14.6	12.4	12.2	11.2	17.1
45-54	22.9	19.4	18.6	24.0	22.0	20.8	23.9	28.3	26.0	25.9	20.6	29.1	17.2	17.1	27.4	28.7	24.5	23.1	29.9
55-64	20.8	28.2	23.4	23.7	21.8	22.6	21.2	31.3	30.4	26.1	15.8	28.8	24.0	22.0	29.0	37.2	29.1	26.1	32.7
Drugs																			
35-44	1.2	4.0	2.0	3.0	2.4	6.0	3.1	1.7	5.1	4.4	4.9	4.0	5.9	6.1	6.8	7.0	5.6	9.8	8.2
45-54	2.4	3.1	2.9	2.3	2.8	5.4	3.6	1.9	6.3	4.2	4.7	6.3	5.7	6.2	7.1	6.9	4.0	6.9	6.4
55-64	0.2	1.6	2.7	1.0	3.2	2.5	2.8	2.0	5.2	3.1	3.6	6.0	3.8	5.1	5.7	5.2	2.6	4.2	4.1
Suicide																			
35-44	3.9	5.8	6.7	4.7	5.9	7.9	9.2	10.7	6.3	7.9	7.2	6.6	12.7	5.8	5.1	4.0	6.5	5.7	7.0
45-54	4.1	4.2	4.3	4.6	7.1	7.3	10.5	9.7	5.0	9.5	6.5	6.2	9.8	4.6	5.7	3.6	4.7	5.1	7.0
55-64	3.6	2.8	1.9	5.1	6.9	6.1	9.4	6.8	3.5	7.1	5.3	4.8	5.5	3.0	4.9	3.1	3.4	4.1	5.8

Dowd JB, *et al. BMJ Open* 2023; 13:e069905. doi: 10.1136/bmjopen-2022-069905

Table S5a - Male mortality rates (per 100,000 population) by cause, age group and year for Scotland

Age group	2001	2002	2003	2004	2005	2006	2007	2008	2009	2010	2011	2012	2013	2014	2015	2016	2017	2018	2019
All-Cause																			
35-44	274.9	267.0	254.2	259.0	254.9	268.9	262.0	261.1	249.4	244.0	237.5	232.9	240.2	231.4	237.8	265.8	266.1	273.8	284.2
45-54	649.0	634.1	606.2	591.9	580.9	580.7	567.1	557.3	530.8	520.1	495.1	493.0	502.7	491.9	507.0	536.6	520.6	525.7	529.8
55-64	1708.3	1712.4	1635.3	1538.6	1468.8	1429.7	1406.5	1370.3	1287.4	1257.4	1194.7	1175.9	1149.4	1121.3	1172.9	1163.9	1101.5	1106.0	1076.2
Combined 'Deaths of Despair'																			
35-44	82.2	91.2	87.4	92.3	89.9	99.7	95.2	106.4	98.0	99.5	99.7	96.2	106.0	103.2	102.9	125.2	130.2	150.5	157.5
45-54	114.9	120.5	116.5	115.7	111.3	120.3	109.5	111.6	105.1	109.1	95.3	92.9	104.6	102.9	101.5	116.9	119.6	138.1	139.2
55-64	127.9	131.0	126.7	125.0	119.1	123.7	115.5	114.0	98.4	108.3	98.0	92.8	90.9	93.9	95.4	106.3	104.3	104.4	98.0
Alcohol																			
35-44	40.9	43.8	44.8	43.8	42.4	46.5	40.6	40.4	35.6	35.0	30.6	26.6	30.6	28.2	25.8	27.6	26.9	26.4	24.7
45-54	86.1	91.1	92.0	87.9	85.0	88.9	81.3	78.7	67.0	70.8	59.3	51.9	56.2	55.7	54.8	58.7	54.9	55.4	48.5
55-64	107.3	110.7	110.7	107.4	102.6	105.0	98.9	94.8	77.3	87.0	80.0	70.2	64.4	69.8	71.3	78.2	74.9	71.7	60.4
Drugs																			
35-44	15.1	16.8	14.1	15.9	21.1	22.5	29.7	36.9	34.8	36.0	40.0	41.4	42.0	47.8	50.0	68.7	73.3	94.0	102.4
45-54	5.5	5.5	4.7	5.6	7.1	9.7	10.0	12.8	14.7	16.6	15.9	16.9	19.8	23.5	25.4	32.7	37.1	57.1	63.6
55-64	2.3	1.7	2.0	2.6	2.1	4.3	3.7	4.5	5.3	6.6	6.1	5.8	6.6	7.2	8.6	9.8	10.3	14.4	15.6
Suicide																			
35-44	26.2	30.6	28.4	32.6	26.3	30.6	24.9	29.1	27.7	28.5	29.1	28.2	33.4	27.1	27.2	28.9	30.0	30.1	30.4
45-54	23.2	23.9	19.8	22.2	19.2	21.8	18.2	20.2	23.4	21.7	20.1	24.2	28.6	23.7	21.4	25.5	27.7	25.6	27.1
55-64	18.3	18.6	14.0	15.0	14.4	14.4	12.9	14.7	15.8	14.7	11.9	16.8	19.9	16.9	15.5	18.3	19.2	18.3	22.0

Dowd JB, *et al. BMJ Open* 2023; 13:e069905. doi: 10.1136/bmjopen-2022-069905

Table S5b - Female mortality rates (per 100,000 population) by cause, age group and year in Scotland

Age group	2001	2002	2003	2004	2005	2006	2007	2008	2009	2010	2011	2012	2013	2014	2015	2016	2017	2018	2019
All-Cause																			
35-44	149.2	147.2	139.8	148.5	146.7	146.0	143.7	144.5	144.9	133.4	142.0	132.1	129.8	133.1	141.3	146.4	141.8	149.7	153.4
45-54	387.9	382.8	376.1	366.9	367.0	363.6	362.0	347.5	344.8	328.6	336.3	317.0	317.7	314.8	327.2	333.5	327.8	338.9	341.1
55-64	1014.3	1009.6	1019.0	934.6	932.2	919.3	909.7	865.6	838.1	835.8	810.4	794.0	778.8	748.0	766.4	766.3	754.3	769.4	759.9
Combined 'Deaths of Despair'																			
35-44	35.6	32.6	32.0	36.4	34.9	35.6	31.9	36.8	38.9	37.4	41.4	34.7	36.5	40.7	47.7	49.0	52.6	58.0	61.7
45-54	48.4	51.2	52.0	49.9	52.5	54.0	48.4	49.2	47.0	46.4	48.0	41.9	44.0	44.4	52.6	51.3	50.5	57.8	64.7
55-64	49.9	53.1	55.9	49.9	55.8	58.5	50.3	49.2	46.9	44.9	45.9	42.1	40.9	38.1	45.0	46.8	45.3	48.6	53.3
Alcohol																			
35-44	19.9	20.6	19.3	19.9	20.7	20.2	19.8	19.2	18.0	17.9	18.4	12.3	14.5	16.1	15.3	13.7	12.8	14.8	12.8
45-54	36.7	40.2	41.4	36.5	41.5	41.7	36.3	36.9	33.1	32.2	32.0	23.7	27.7	27.2	28.7	27.5	26.3	28.7	28.4
55-64	41.3	43.4	47.9	41.0	47.9	50.4	39.8	41.7	38.8	35.6	36.4	31.1	31.5	28.9	32.2	34.0	33.7	34.9	37.5
Drugs																			
35-44	4.8	3.7	4.7	5.6	5.7	7.8	5.0	8.5	13.0	10.5	14.6	13.8	15.4	15.3	23.2	26.5	33.1	34.0	40.2
45-54	2.8	2.7	3.5	3.5	3.4	4.4	4.8	5.5	6.6	7.5	9.0	10.4	9.8	9.6	14.3	15.2	17.2	20.9	26.1
55-64	2.0	2.2	2.2	2.7	1.8	2.4	4.1	3.4	2.4	4.7	4.6	5.7	4.1	3.9	5.4	5.8	5.5	7.5	9.0
Suicide																			
35-44	10.9	8.2	7.9	11.0	8.4	7.6	7.1	9.1	7.9	8.9	8.5	8.7	6.6	9.3	9.2	8.8	6.8	9.2	8.7
45-54	9.0	8.3	7.1	9.9	7.6	7.9	7.3	6.7	7.3	6.7	7.0	7.8	6.4	7.6	9.6	8.5	7.0	8.2	10.2
55-64	6.6	7.5	5.9	6.3	6.0	5.7	6.3	4.0	5.7	4.6	4.8	5.3	5.4	5.3	7.5	7.1	6.0	6.2	6.8

Table S6a - Male mortality rates (per 100,000 population) by cause, age group and year for the USA

Age group	2001	2002	2003	2004	2005	2006	2007	2008	2009	2010	2011	2012	2013	2014	2015	2016	2017	2018	2019
All-Cause																			
35-44	259.2	258.0	254.3	244.0	244.0	240.4	233.5	225.5	223.9	212.3	213.6	211.8	212.9	216.6	226.2	244.2	252.2	251.7	257.3
45-54	551.3	557.1	561.1	551.0	556.2	548.7	536.6	531.3	524.2	508.9	507.4	500.0	497.9	494.0	494.0	496.7	495.4	490.2	484.9
55-64	1226.1	1216.1	1200.0	1157.0	1153.1	1126.4	1108.7	1110.6	1098.0	1082.8	1081.4	1090.8	1101.8	1112.5	1119.2	1124.2	1122.5	1124.4	1106.7
Combined 'Deaths of Despair'																			
35-44	58.2	61.6	61.4	60.1	60.5	63.0	62.1	61.0	61.2	61.0	63.2	63.8	64.6	68.1	76.0	87.3	96.0	96.2	101.6
45-54	77.2	81.2	84.9	84.9	87.8	92.5	91.6	93.2	92.5	94.1	95.9	96.0	96.9	98.7	102.5	108.4	115.2	113.7	115.5
55-64	68.7	71.7	73.2	73.0	76.8	77.5	81.9	86.5	88.6	93.0	96.7	101.3	107.0	111.1	114.6	122.6	128.4	132.3	134.8
Alcohol																			
35-44	14.9	14.3	14.0	13.1	12.6	12.1	12.1	12.1	11.9	12.4	12.1	12.0	12.6	12.7	13.8	14.0	14.8	15.5	16.9
45-54	36.9	36.8	37.8	36.4	36.5	36.2	36.0	36.2	35.7	36.8	36.8	37.1	37.0	36.8	37.4	36.3	36.9	37.0	37.1
55-64	42.9	43.5	43.7	43.5	44.8	43.8	45.3	47.5	47.9	50.3	52.3	54.8	57.6	59.3	60.4	61.7	62.1	63.0	63.2
Drugs																			
35-44	20.1	23.2	23.8	23.5	24.2	27.1	25.6	24.3	24.1	23.6	25.7	26.0	27.2	30.1	36.0	46.9	53.2	52.6	56.6
45-54	16.7	19.9	22.5	23.5	26.0	29.8	28.4	28.0	27.0	26.7	28.8	28.4	30.1	31.7	34.7	42.6	47.9	46.2	49.2
55-64	4.6	6.1	7.0	7.5	9.7	11.0	12.6	13.3	14.3	15.1	16.9	17.8	20.7	22.2	25.0	31.7	36.1	37.4	41.1
Suicide																			
35-44	23.3	24.1	23.6	23.5	23.7	23.7	24.4	24.5	25.1	25.1	25.4	25.8	24.8	25.3	26.1	26.4	28.0	28.1	28.1
45-54	23.7	24.6	24.5	25.0	25.3	26.5	27.2	28.9	29.7	30.6	30.3	30.5	29.9	30.2	30.4	29.5	30.4	30.5	29.3
55-64	21.2	22.2	22.5	22.0	22.3	22.7	24.0	25.6	26.4	27.6	27.5	28.7	28.7	29.6	29.2	29.2	30.2	31.9	30.5

Dowd JB, *et al. BMJ Open* 2023; 13:e069905. doi: 10.1136/bmjopen-2022-069905

Table S6b - Female mortality rates (per 100,000 population) by cause, age group and year in the USA

Age group	2001	2002	2003	2004	2005	2006	2007	2008	2009	2010	2011	2012	2013	2014	2015	2016	2017	2018	2019
All-Cause																			
35-44	147.6	148.2	146.9	142.4	142.0	140.4	135.8	133.7	134.0	128.1	129.6	127.9	129.4	133.1	134.6	141.0	142.9	141.6	142.8
45-54	319.8	321.9	322.5	318.1	323.8	320.7	317.2	318.8	319.9	311.7	314.4	311.1	311.6	313.9	313.5	314.1	308.8	301.0	295.1
55-64	773.4	758.0	753.7	724.8	713.0	697.9	677.4	673.1	662.4	647.9	647.4	649.3	653.5	664.3	668.9	675.4	678.4	671.6	667.8
Combined 'Deaths of Despair'																			
35-44	22.0	24.3	24.6	25.4	25.4	26.7	26.9	27.2	26.8	27.7	29.4	28.7	29.7	31.8	34.1	37.6	40.4	39.6	40.5
45-54	26.3	29.3	31.2	33.7	35.1	37.8	40.1	41.0	42.7	42.8	46.6	46.7	47.2	48.6	50.9	51.6	53.4	50.6	51.2
55-64	24.1	24.8	25.7	26.8	27.0	28.8	30.7	31.4	33.3	35.1	37.2	38.6	42.0	45.5	48.2	50.2	51.8	52.5	53.8
Alcohol																			
35-44	6.4	6.1	5.7	5.3	5.4	5.5	5.3	5.5	5.4	5.5	5.7	5.8	5.8	6.3	6.9	7.2	7.6	7.9	8.6
45-54	12.1	12.0	12.1	12.4	12.6	12.8	13.3	13.7	14.3	14.6	16.3	15.9	16.1	16.4	17.6	16.9	17.5	17.3	17.7
55-64	15.8	15.7	15.7	15.7	15.5	15.6	15.7	16.1	17.2	17.3	18.8	19.6	20.4	22.4	23.7	24.0	24.7	25.3	26.7
Drugs																			
35-44	9.2	11.3	12.3	13.0	13.2	14.3	14.3	14.3	13.9	14.7	16.1	15.2	16.2	17.2	18.8	21.8	24.2	23.3	23.6
45-54	7.0	9.8	11.3	12.8	14.4	16.5	18.0	18.3	19.1	19.0	20.5	20.6	21.0	21.5	22.6	24.4	25.5	23.1	23.2
55-64	2.6	3.5	4.2	4.9	5.5	6.5	7.8	8.4	8.8	9.9	10.7	11.0	13.0	14.2	14.8	16.8	18.1	17.6	18.2
Suicide																			
35-44	6.5	6.9	6.7	7.1	6.8	7.0	7.3	7.4	7.4	7.4	7.6	7.8	7.7	8.3	8.4	8.5	8.6	8.4	8.2
45-54	7.2	7.5	7.8	8.5	8.1	8.5	8.8	9.1	9.3	9.2	9.8	10.2	10.1	10.7	10.7	10.3	10.4	10.2	10.3
55-64	5.7	5.6	5.8	6.1	6.0	6.8	7.2	6.9	7.4	7.9	7.7	8.0	8.6	8.9	9.7	9.4	9.0	9.5	8.9

Dowd JB, *et al. BMJ Open* 2023; 13:e069905. doi: 10.1136/bmjopen-2022-069905
