# Supplementary material for: Comparing trends in mid-life ‘deaths of despair’ in the USA, Canada and UK, 2001–2019: is the USA an anomaly?
Source: BMJ Open. 2023 Aug 17;13(8):e069905. doi: 10.1136/bmjopen-2022-069905 (PMC10441077; doi:10.1136/bmjopen-2022-069905)
Supplement: Author's manuscript [file bmjopen-2022-069905.draft_revisions.pdf]

BMJ Open is committed to open peer review. As part of this commitment we make the peer review history of every article we publish publicly available.

When an article is published we post the peer reviewers' comments and the authors' responses online. We also post the versions of the paper that were used during peer review. These are the versions that the peer review comments apply to.

The versions of the paper that follow are the versions that were submitted during the peer review process. They are not the versions of record or the final published versions. They should not be cited or distributed as the published version of this manuscript.

BMJ Open is an open access journal and the full, final, typeset and author-corrected version of record of the manuscript is available on our site with no access controls, subscription charges or pay-per-view fees (http://bmjopen.bmj.com).

If you have any questions on BMJ Open's open peer review process please email info.bmjopen@bmj.com

BMJ Open

Mid-life "Deaths of Despair" Trends in the US, Canada, and UK, 2001-2019: Is the US an Anomaly?

Journal:	*BMJ Open*
Manuscript ID	bmjopen-2022-069905
Article Type:	Original research
Date Submitted by the Author:	01-Dec-2022
Complete List of Authors:	Dowd, Jennifer; University of Oxford, Leverhulme Centre for Demographic Science; University of Oxford Nuffield College<br>Angus, Colin; University of Sheffield, ScHARR<br>Zajacova, Anna; University of Western Ontario,<br>Tilstra, Andrea; University of Oxford, Leverhulme Centre for Demographic Science; University of Oxford Nuffield College
Keywords:	Substance misuse < PSYCHIATRY, Suicide & self-harm < PSYCHIATRY, PUBLIC HEALTH

SCHOLARONE™
Manuscripts

I, the Submitting Author has the right to grant and does grant on behalf of all authors of the Work (as defined in the below author licence), an exclusive licence and/or a non-exclusive licence for contributions from authors who are: i) UK Crown employees; ii) where BMJ has agreed a CC-BY licence shall apply, and/or iii) in accordance with the terms applicable for US Federal Government officers or employees acting as part of their official duties; on a worldwide, perpetual, irrevocable, royalty-free basis to BMJ Publishing Group Ltd ("BMJ") its licensees and where the relevant Journal is co-owned by BMJ to the co-owners of the Journal, to publish the Work in this journal and any other BMJ products and to exploit all rights, as set out in our licence.

The Submitting Author accepts and understands that any supply made under these terms is made by BMJ to the Submitting Author unless you are acting as an employee on behalf of your employer or a postgraduate student of an affiliated institution which is paying any applicable article publishing charge ("APC") for Open Access articles. Where the Submitting Author wishes to make the Work available on an Open Access basis (and intends to pay the relevant APC), the terms of reuse of such Open Access shall be governed by a Creative Commons licence – details of these licences and which Creative Commons licence will apply to this Work are set out in our licence referred to above.

Other than as permitted in any relevant BMJ Author's Self Archiving Policies, I confirm this Work has not been accepted for publication elsewhere, is not being considered for publication elsewhere and does not duplicate material already published. I confirm all authors consent to publication of this Work and authorise the granting of this licence.

For peer review only

Mid-life "Deaths of Despair" Trends in the US, Canada, and UK, 2001-2019: Is the US an Anomaly?

Jennifer Beam Dowd*[1,2], Colin Angus[3], Anna Zajacova[4], Andrea M. Tilstra[1,2]

[1] Leverhulme Centre for Demographic Science, Department of Sociology, University of Oxford, Oxford, UK
[2] Nuffield College, University of Oxford, Oxford, UK
[3] School of Health and Related Research, University of Sheffield, Sheffield, UK
[4] Department of Sociology, University of Western Ontario, London, Ontario, Canada

*Corresponding Author
Jennifer Beam Dowd
Leverhulme Centre for Demographic Science
University of Oxford
42-42 Park End Street
Oxford OX1 1JD
United Kingdom
Jennifer.dowd@sociology.ox.ac.uk
+ 44 7986830169

Key Words: Demography, Mortality, Suicide, Substance Abuse

Word Count: 2742

For peer review only

Abstract

Objectives

Over the past decade, "deaths of despair" were strongly implicated in rising mid-life mortality in the US. We examine whether despair deaths and mid-life mortality trends are also changing in the peer countries such as the UK and Canada.

Methods

We compared all-cause and "despair" mortality trends at mid-life in the US, the UK (constituent nations England & Wales, Northern Ireland, and Scotland) and Canada from 2000-2019, using publicly available mortality data, stratified by three age groups (35-44, 45-54 and 55-64) and by sex. We examined trends in all-cause mortality and mortality by causes categorized as 1) suicides 2) alcohol-specific deaths 3) drug-related deaths. We employ several descriptive approaches to visually inspect age, period, and cohort trends in these causes of death.

Results

The US and Scotland both saw large increases and high absolute levels of drug-related deaths. The rest of the UK and Canada saw relative increases but much lower absolute levels in by comparison. Alcohol-specific deaths showed less consistent trends that did not track other "despair" causes, with older groups in Scotland seeing steep declines over time. Suicide deaths trended slowly upward in most countries.

Conclusions

In the UK, Scotland has suffered increases in drug-related mortality comparable to the US, while Canada and other UK constituent nations did not see dramatic increases. Alcohol-specific and suicide mortality generally follow different patterns to drug-related deaths across countries and over time, questioning the utility of a cohesive "deaths of despair" narrative.

For peer review only

Strengths and Limitations of this Study:

- We directly compare trends in "deaths of despair" in mid-life across the US, UK, and Canada.
- We leverage demographic approaches to understand period vs. cohort trends in these causes of death.
- Coding of deaths caused by alcohol, drug overdose, and suicide may vary across country and time.
- Disentangling age, period, and cohort effects in mortality trends is challenging.
- The study is descriptive and cannot speak to the causes of trends in drug overdose, suicide, and alcohol-specific deaths.

For peer review only

Introduction

The United States (US) has persistently lower life expectancy than its OECD peers, including 1.7-3.2 years below the United Kingdom (UK) and Canada in 2019 [1], two countries with a shared cultural and linguistic heritage. US life expectancy fell for three consecutive years starting in 2014, recovering slightly by 2019 prior to further declines in 2020 and 2021 during the COVID-19 pandemic [2, 3]. Explanations for stagnating life expectancy in the U.S and divergence from peer countries have highlighted increases in "deaths of despair" (i.e., deaths due to drug and alcohol poisoning, and suicide)[4, 5]. Yet, in the 2010's there was evidence that life expectancy may also be stalling in England and Wales [6, 7] and Scotland [8] raising the question of whether these mortality dynamics reflect a broader phenomenon.

The US, the UK, and Canada have all documented rapid increases in drug-related mortality in recent years, with the US on the leading edge with an initial increase in deaths due to prescription opioids, followed by heroin and now fentanyl [9, 10]. Increases in opioid use in Canada may be related to exposure to US pharmaceutical promotional material through US media and direct marketing [11]. Within the UK, Scotland stands out with a dramatic rise in drug deaths (rising 450% since 1996) driven initially by prescription benzodiazepines and more recently by so-called 'street' benzodiazepines [12]. England and Wales have had a less extreme rise in drug-related deaths since 2012, driven by increasing numbers of opioid and cocaine-related deaths, and less noticeable increases due to benzodiazepines.[1]

Besides changes in the supply and marketing of drugs, increases in drug mortality may reflect demand side factors related to the loss of economic opportunity, particularly among socioeconomically disadvantaged groups [13, 14]. In Scotland, cohort trends in drug-related

[1] https://www.ons.gov.uk/peoplepopulationandcommunity/birthsdeathsandmarriages/deaths/bulletins/deathsrelatedtodrugpoisoninginenglandandwales/2020#drug-poisonings-from-selected-substances

For peer review only

deaths and suicide were evident particularly in men from socioeconomically deprived areas coming of age in the 1980s [15, 16]. In the US and the UK, lack of economic opportunity in middle-aged cohorts may contribute to poorer mental health and subsequent coping behaviors [17]. In Canada, socioeconomic inequalities in health measured by educational attainment and household income have increased over time, but less is known about whether this has translated into despair related causes of death [18, 19].

This paper compares recent trends in mid-life "deaths of despair" and its component causes in the US to those in the UK and Canada from 2000-2019. We find that while increases in drug-related mortality were common across all nations, absolute levels are especially high in the US and Scotland. Overall, patterns in drug-related mortality were distinct from alcohol-specific and suicide deaths, questioning the utility of a cohesive "deaths of despair" framework.

Data

Publicly available cause-specific mortality by age and sex were analysed for all countries. Because data are collected by separate statistical offices for the nations of the UK, and because of historically divergent mortality trends for Scotland, we estimate trends separately for UK nations, except for England and Wales whose data is released together. For England and Wales, we analyzed the 21st Century Mortality dataset from 2001-2019,[2] a publicly available resource that includes all registered deaths by cause as well as mid-year population counts by five-year age groups. Comparable data for Scotland is available from National Records of Scotland's Vital Events Reference Tables[3] and for Northern Ireland from The Registrar General's Annual

[2] https://www.ons.gov.uk/peoplepopulationandcommunity/birthsdeathsandmarriages/deaths/datasets/the21stcenturymortalityfilesdeathsdataset

[3] https://www.nrscotland.gov.uk/statistics-and-data/statistics/statistics-by-theme/vital-events/general-publications/vital-events-reference-tables/archive

For peer review only

Reports.[4] For Canada, death counts by cause of death were accessed from publicly available Statistics Canada Table 13-10-0392-01[5]. These records include all registered deaths in Canada each year from 2000-2019 by sex and 5-year age-group. US mortality data by year and single year of age come from CDC Wonder.[6] Population counts by age and sex for all countries were obtained from the Human Mortality Database.

Methods

We focus on mid-life mortality where increases in mortality have been seen in the U.S, specifically age groups 35-44, 45-54 and 55-64. We examine trends mortality by causes categorized as 1) suicides, 2) alcohol-specific deaths 3) drug-related deaths, and a combined 4) “deaths of despair” category as well as all-cause mortality. (ICD-10 codes available in supplemental Table S1). In order to mitigate the impact of random variation in the cause-and age-specific mortality rates we used a 1-dimensional P-spline approach for each country, cause, sex and calendar year to smooth the observed death rates over age [20]. The resulting single year of age-specific mortality rates were then age-standardised within each of the three broad age groups using the European Standard Population [21]. We visually inspected cohort vs. period trends using plots of the 3-year rolling averages within age-groups over time, supplemented with Lexis surfaces. Results are fully reproducible, and all data and code can be accessed here.

Patient and Public Involvement

None

Results

[4] https://www.nisra.gov.uk/publications/registrar-general-annual-reports-2011-2019
[5] Statistics Canada mortality data are accessible at: https://www150.statcan.gc.ca/t1/tbl1/en/cv.action?pid=1310039201
[6] CDC Wonder data are accessible at: https://wonder.cdc.gov/.

For peer review only

Full results for all age group, sex, country and cause groupings for all calendar years are shown in Appendix Tables S2a – S6b, with summary data for 2001, 2010 and 2019 presented in Tables 1a-b.

For peer review only

Table 1a - Male mortality rates (per 100,000 population) by cause and age group

	Canada			England & Wales			Northern Ireland			Scotland			USA		
Age group	2001	2010	2019	2001	2010	2019	2001	2010	2019	2001	2010	2019	2001	2010	2019
All-Cause															
35-44	183.6	164.1	162.0	190.7	168.9	162.8	192.7	207.7	190.4	274.9	244.0	284.2	259.2	212.3	257.3
45-54	424.3	361.3	334.8	461.4	393.9	371.0	465.7	443.7	378.1	649.0	520.1	529.8	551.3	508.9	484.9
55-64	1156.0	917.0	825.7	1280.1	991.9	888.6	1324.3	1092.9	876.9	1708.3	1257.4	1076.2	1226.1	1082.8	1106.7
Combined 'Deaths of Despair'															
35-44	44.4	46.3	50.1	44.8	48.0	58.0	47.6	67.9	81.9	82.2	99.5	157.5	58.2	61.0	101.6
45-54	53.7	58.2	62.6	50.5	58.0	71.1	54.9	82.6	85.0	114.9	109.1	139.2	77.2	94.1	115.5
55-64	67.9	68.3	75.5	46.6	56.0	66.1	50.0	79.6	74.5	127.9	108.3	98.0	68.7	93.0	134.8
Alcohol															
35-44	8.2	8.5	8.4	19.8	20.3	16.3	26.7	26.3	29.0	40.9	35.0	24.7	14.9	12.4	16.9
45-54	22.7	22.1	21.0	32.7	37.4	35.3	41.2	50.8	55.5	86.1	70.8	48.5	36.9	36.8	37.1
55-64	42.5	38.5	40.2	33.1	41.4	43.3	38.1	56.8	58.8	107.3	87.0	60.4	42.9	50.3	63.2
Drugs															
35-44	9.7	13.4	21.1	9.8	12.8	22.8	1.6	10.6	23.6	15.1	36.0	102.4	20.1	23.6	56.6
45-54	6.1	11.7	19.6	4.6	6.1	16.6	1.8	6.3	6.2	5.5	16.6	63.6	16.7	26.7	49.2
55-64	3.2	7.1	13.1	2.3	2.5	6.5	2.0	4.1	0.8	2.3	6.6	15.6	4.6	15.1	41.1
Suicide															
35-44	26.6	24.4	20.6	15.2	14.9	18.9	19.4	31.0	29.3	26.2	28.5	30.4	23.3	25.1	28.1
45-54	24.9	24.4	22.0	13.2	14.5	19.1	11.9	25.5	23.4	23.2	21.7	27.1	23.7	30.6	29.3
55-64	22.3	22.7	22.1	11.1	12.1	16.3	9.9	18.7	15.0	18.3	14.7	22.0	21.2	27.6	30.5

Table 1b - Female mortality rates (per 100,000 population) by cause and age group

	Canada			England & Wales			Northern Ireland			Scotland			USA		
Age group	2001	2010	2019	2001	2010	2019	2001	2010	2019	2001	2010	2019	2001	2010	2019
All-Cause															
35-44	112.1	100.2	94.6	117.5	104.8	98.7	119.4	122.3	115.0	149.2	133.4	153.4	147.6	128.1	142.8
45-54	268.6	235.9	212.6	302.5	260.4	243.0	310.2	292.7	274.0	387.9	328.6	341.1	319.8	311.7	295.1
55-64	685.8	586.7	530.0	802.5	641.2	587.8	809.9	704.9	639.7	1014.3	835.8	759.9	773.4	647.9	667.8
Combined 'Deaths of Despair'															
35-44	15.9	19.1	19.0	16.8	18.3	23.2	20.1	26.3	32.3	35.6	37.4	61.7	22.0	27.7	40.5
45-54	20.0	24.3	24.1	23.0	24.6	30.5	29.4	39.6	43.3	48.4	46.4	64.7	26.3	42.8	51.2
55-64	24.1	26.7	28.9	23.7	25.4	29.4	24.6	36.3	42.5	49.9	44.9	53.3	24.1	35.1	53.8
Alcohol															
35-44	4.0	4.2	4.3	9.6	10.2	9.7	15.0	13.9	17.1	19.9	17.9	12.8	6.4	5.5	8.6
45-54	8.6	8.5	9.5	16.4	17.3	18.2	22.9	25.9	29.9	36.7	32.2	28.4	12.1	14.6	17.7
55-64	14.7	13.2	16.4	17.7	19.6	20.5	20.8	26.1	32.7	41.3	35.6	37.5	15.8	17.3	26.7
Drugs															
35-44	4.1	7.2	9.0	3.5	4.3	8.4	1.2	4.4	8.2	4.8	10.5	40.2	9.2	14.7	23.6
45-54	3.4	7.3	8.2	2.5	3.3	7.1	2.4	4.2	6.4	2.8	7.5	26.1	7.0	19.0	23.2
55-64	2.5	5.7	5.8	1.9	2.1	4.2	0.2	3.1	4.1	2.0	4.7	9.0	2.6	9.9	18.2
Suicide															
35-44	7.8	7.6	5.7	3.8	3.8	5.2	3.9	7.9	7.0	10.9	8.9	8.7	6.5	7.4	8.2
45-54	8.0	8.4	6.5	4.2	4.0	5.3	4.1	9.5	7.0	9.0	6.7	10.2	7.2	9.2	10.3
55-64	6.9	7.8	6.7	4.1	3.7	4.7	3.6	7.1	5.8	6.6	4.6	6.8	5.7	7.9	8.9

For peer review only - http://bmjopen.bmj.com/site/about/guidelines.xhtml

For peer review only

Figure 1 shows all-cause mortality rates from 2001-2019 by age and sex group. Observed mortality patterns differ across nations and by sex. In Canada and England & Wales, mortality rates are low and decline consistently for most age-sex combinations, except for 35-44 year olds for which mortality trends are flat or increasing. Upticks in mortality were more noticeable in Scotland and the US in 35–44-year-olds, with steeper increases for males compared to females. In the US, all-cause mortality among males 35-44 reached a low point of 211.8/100,000 in 2012 before rising steadily to 257.3/100,000 in 2019 (Appendix Table S6a**)**. Males aged 35-44 in Scotland followed a similar trend, with slightly higher absolute levels of mortality compared to US males. This youngest age group also showed signs of stalling improvements or slight increases in mortality in Canada, England & Wales, and Northern Ireland. Noticeable increases in overall mortality were also seen in Scotland for males and females ages 45-64, but not in the 55–64-year-old age group. The US was notable for its stagnation or increases in mortality across almost all age-sex groups.

Figure 2 shows trends in "despair" deaths over the same period. The US and Scotland again stand out for substantially higher and largely increasing rates across the period. Males aged 35-44 show some of the worst trends among analyzed countries, with rates in the US and Scotland increasing almost 2-fold over the period. While the absolute increases for males in England and Wales and Northern Ireland are much smaller, they reflect large relative increases (for example a 41% increase in despair-related deaths for males ages 45-54 over this period in England and Wales). Canada is notable for its low levels and relatively small increases in deaths of despair, especially for women for whom rates hardly increase. While females in most nations saw only small increases in deaths of despair, the US stood out with a trend of high and rising for both males and females in all age groups.

For peer review only - http://bmjopen.bmj.com/site/about/guidelines.xhtml

Figures 2a-c break down these "despair" deaths into individual causes, where we see the salience of increasing drug-related mortality in the US and Scotland. The magnitude of the increases for males ages 35-44 in both countries was staggering (an almost 6-fold increase in Scotland from 15.1/100,000 to 102.3/100,000). In the US these increases are seen across all age groups, while in Scotland the increases are significantly less pronounced for 55–64-year-olds. While showing lower absolute levels, drug-related mortality also increased in Canada, England and Wales and Northern Ireland over the period, especially among younger males (for example an increase of 118% from 9.1/100,000 to 21.1/100,000 for Canadian males aged 35-44).

Suicide mortality saw more year-to-year variability, with evidence of small increases among males in England & Wales, Scotland, and the US. The magnitude of these changes is very small compared to changes in drug-related deaths. Suicide rates were higher for males in all nations. Canada consistently had the lowest levels of suicide mortality across all age-sex groups. The US generally had the highest levels of suicide deaths, except for males ages 35-44 where Scotland and Northern Ireland had higher levels. Alcohol-specific mortality across most nations was relatively stable, but with sharp declines over time in Scotland from previously very high levels, especially at the older ages. In contrast, increases in alcohol-specific mortality in the US were seen in ages 55-64, especially for men. Taken together, these results suggest that drug-related mortality is the primary cause of increases in overall "despair-related" mortality over this period, with small but perceptible increases in England & Wales, Canada, and Northern Ireland, and very pronounced increases in the US and Scotland.

Figures S1a-c show an alternative view of the same data to examine period vs. cohort trends. Here we look for "non-parallelism" in the age-specific mortality curves over time within each country [22]. If the age-specific curves are parallel to each other, this likely reflects the

For peer review only

salience of period influences. Non-parallel age-specific trends suggest more importance for cohort effects (For a detailed discussion and simulations of these dynamics, see [23], pages 204-217). For overall deaths of despair, age-specific trends over time are mostly parallel for Canada, England & Wales, and Northern Ireland, suggesting period-based dynamics affecting all cohorts. The US and Scotland show some deviations from parallelism, with the youngest age group showing sharper increases than the older groups in Scotland, and the 55–64-year-olds in the US increasing more steeply (and earlier) than younger groups. For drug-related deaths, parallel trends are largely suggestive of period-based effects for Canada, England & Wales, and the US. Non-parallelism in Northern Ireland & Scotland suggests younger born cohorts have been hit harder by recent increases in drug deaths than the oldest cohort. For suicide, most trends are parallel suggesting period fluctuations, except for the US where the oldest age group has increased more steeply. Alcohol-specific mortality mostly follows period-based parallel trends, except for the US where again the oldest age group has increased over time. Cohort-driven declines in alcohol-related mortality were seen in Scotland, with the older groups declining steeply from high levels compared to lower and flatter trends in the youngest age group. Supplemental Figures S2a-b and show alternative visualizations for cohort patterns in mortality using Lexis surfaces. Overall, these visualizations highlight some limited cohort trends but also very strong period effects of increased drug deaths that affected most age groups and nations. In the US, increases in drug-related deaths affected all age groups over time, but sharper increases in alcohol-specific deaths and suicide were only seen in the oldest age group (earliest born cohort). This oldest group largely reflects the Baby Boom cohort (born 1946-1964) passing this 55-64 age group over the observed period.

For peer review only

Discussion

Recent increases in mid-life mortality in the US set off a robust debate about the underlying causes of this concerning trend, with a strong focus on “deaths of despair.” One narrative emphasizes the role of cumulative disadvantage triggered by a worsening of labor market opportunities for white Americans with low levels of education, leading to psychological distress and unhealthy behavioral coping mechanisms [5, 24, 25]. A second hypothesis emphasizes the strong period trend in drug-related mortality due to changes in the availability of prescription opioids, heroin, and synthetic opioids (e.g., fentanyl)[26, 27]. While the first hypothesis implies that all “despair” deaths share a fundamental cause and move together, the latter argues that the trends in suicide and alcohol-specific mortality do not fit well within the overall deaths of despair narrative [28, 29].

Our study extends this work by comparing mid-life mortality trends in the US to Canada and the UK, two countries with shared sociocultural histories with the US but different economic, political and health care contexts. The UK has also experienced recent slowdowns in mortality improvements, raising the question of whether the US is the leading edge of more general mortality trends. Overall, we find evidence that the leveling off and increases in mid-life mortality seen in the US may be emerging more broadly, but the magnitude of these changes in other countries is muted. Scotland is the exception, with upward trends in drug-related deaths surpassing the high levels in the US and contributing to increases in overall mortality trends for males ages 35-54. While drug-related deaths have increased on a relative scale in the other UK nations and Canada, deaths from these causes are at much lower absolute levels than the US and Scotland. There is little evidence of significant trends in suicide or alcohol-specific deaths contributing to changes in all-cause mortality.

For peer review only

Our results suggest that increases in drug-related mortality are not necessarily unique to the US, with smaller increases also seen in Canada, England & Wales, and Northern Ireland, particularly among younger males. Scotland stands out as trending even higher than the US in drug-related mortality at younger ages, while the US saw parallel increases in drug-mortality across all age-ranges, suggesting a strong period effect. We found some evidence of cohort effects in alcohol-specific and suicide mortality in the US, with the oldest cohort trending up relative to younger cohorts. Suicide mortality rates were generally stable across countries for women, with slight increases for men and women in the US. Overall, with the exception of the oldest born (i.e. baby boom) male cohort in the US, we find that individual "deaths of despair" do not follow similar age and cohort patterns and thus may not reflect the same fundamental causes.

The fact that drug-related deaths have increased in other countries but not to the degree of the US is consistent with differences in prescribing practices and health care contexts. In the UK, there is no direct to consumer marketing by pharmaceutical companies, and the primary care system likely mitigates patients receiving prescriptions from multiple sources [30]. Nonetheless, prescribing for non-cancer pain did increase dramatically since the late 1990s when consensus statements supporting the use of opioids for the treatment of chronic pain [31, 32]. Despite this increase in overall opioid prescribing, a review of the UK Clinical Practice Research Datalink (CRPD) did not find an increase in the rate of diagnosed opioid use disorders in the UK between 2008 and 2012 [33]. While deaths from fentanyl in the UK have grown, they are still small relative to deaths from heroin or morphine, suggesting the context for drug prescribing and misuse may be different in the UK [34]. The dramatic rise in drug deaths in Scotland, with rates

For peer review only - http://bmjopen.bmj.com/site/about/guidelines.xhtml

far outstripping those elsewhere else in Europe[7] has arisen through a somewhat different mechanism to the US' prescription opioid-driven crises. A combination of economic, social and political factors experienced by those living in deprived areas in the 1980s led to a rise in drug-related death rates in the 1990s [15, 16], driven by opioids such as heroin, often taken in combination with prescription benzodiazepines such as diazepam [35]. Although steps were taken to reduce the prescribing of benzodiazepines in the late 2000s, these were swiftly replaced by a range of 'synethic benzodiazepines' or 'street benzos' such as etizolam, which became widely and cheaply available through illicit markets [12]. The explosion in the use of street benzos has played a huge role in the rapid rise in Scottish drug deaths since 2012 when street benzos were implicated in 3% of all drug deaths compared to 66% in 2020, with concurrent use with opioids in particular the key factor – less than 1% of drug deaths in 2020 involved street benzos alone[8]. While concern over the opioid epidemic in Canada is rising, the absence of overall increases in mortality thus far has minimized public or scholarly discussion of a similar epidemic of "deaths of despair."

Conclusions

"Deaths of despair" due to alcohol, drug-overdoses, and suicide have been implicated in rising mid-life mortality in the US, but how these trends compare to peer countries is less well-known. We find that the US is unique in its large relative and absolute increases in drug-related mortality for all mid-life age-sex-groups, but that Scotland has seen a dramatic upward trend in drug deaths that now exceed the U.S. for younger age groups. The huge increase in drug mortality across all ages in the US from 2001-2019 is most consistent with period explanations,

[7] https://www.nrscotland.gov.uk/files//statistics/drug-related-deaths/20/drug-related-deaths-20-pub.pdf

[8] https://www.nrscotland.gov.uk/statistics-and-data/statistics/statistics-by-theme/vital-events/deaths/drug-related-deaths-in-scotland/2020

For peer review only - http://bmjopen.bmj.com/site/about/guidelines.xhtml

while alcohol and suicide upward trends are more focused in the oldest cohort. Overall, the trends in individual causes of despair deaths are not consistent across countries or age-groups, casting doubt on the underlying unity of a "deaths of despair" narrative.

Ethics Approval Statement

The study does not involve human subjects, as it uses publicly available death counts from national vital statistics and no individual-level data.

Contributorship statement

The authors confirm contribution to the paper as follows: study conception and design: JBD; data collection CA, JBD, AZ; analysis and interpretation of results: CA, JBD; draft manuscript preparation: JBD. All authors critically reviewed the results and approved the final version of the manuscript.

Competing interests

The authors have not competing interests to declare.

Data sharing statement

Results are fully reproducible, and all data and code can be accessed here.

Funding Acknowledgement

The authors acknowledge funding support from the European Research Council (ERC-2021-CoG-101002587) and The Leverhulme Trust (Large Centre Grant). The funders played no direct role on this research.

For peer review only

References

1. Aburto JM, Schöley J, Kashnitsky I, Zhang L, Rahal C, Missov TI, et al. Quantifying impacts of the COVID-19 pandemic through life-expectancy losses: a population-level study of 29 countries. International Journal of Epidemiology. 2021. doi: 10.1093/ije/dyab207.

2. Murphy SL, Xu J, Kochanek KD, Arias E. Mortality in the united states, 2017. 2018.

3. Schöley J, Aburto JM, Kashnitsky I, Kniffka MS, Zhang L, Jaadla H, et al. Bounce backs amid continued losses: Life expectancy changes since COVID-19. medRxiv. 2022:2022.02.23.22271380. doi: 10.1101/2022.02.23.22271380.

4. Tilstra AM, Simon DH, Masters RK. Trends in "Deaths of Despair" among Working Aged White and Black Americans, 1990-2017. American journal of epidemiology.

5. Case A, Deaton A. Mortality and morbidity in the 21st century. Brookings Papers on Economic Activity. 2017.

6. Hiam L, Dorling D, Harrison D, McKee M. Why has mortality in England and Wales been increasing? An iterative demographic analysis. Journal of the Royal Society of Medicine. 2017;110(4):153-62.

7. Murphy ML, Marc; Torrisi, Orsola. Stalling of mortality in the United Kingdom and Europe: an analytical review of the evidence. LSE Department of Social Policy Working Paper Series2019.

8. Minton J, Fletcher E, Ramsay J, Little K, McCartney G. How bad are life expectancy trends across the UK, and what would it take to get back to previous trends? J Epidemiol Community Health. 2020;74(9):741-6.

9. Ciccarone D. The triple wave epidemic: Supply and demand drivers of the US opioid overdose crisis. International Journal of Drug Policy. 2019;71:183-8. doi: https://doi.org/10.1016/j.drugpo.2019.01.010.

10. John E, Butt A, McQuade G. Deaths related to drug poisoning in England and Wales: 2018 registrations. Office for National Statistics, Aug; 2019.

11. Lexchin J, Kohler JC. The danger of imperfect regulation: OxyContin use in the United States and Canada. International Journal of Risk & Safety in Medicine. 2011;23(4):233-40.

For peer review only

12. McAuley A, Matheson C, Robertson JR. From the clinic to the street: the changing role of benzodiazepines in the Scottish overdose epidemic. International Journal of Drug Policy. 2022;100:103512. doi: https://doi.org/10.1016/j.drugpo.2021.103512.

13. O'Brien R, Bair EF, Venkataramani AS. Death by Robots? Automation and Working-Age Mortality in the United States. Demography. 2022;59(2):607-28. doi: 10.1215/00703370-9774819.

14. Ruhm CJ. Drivers of the fatal drug epidemic. Journal of health economics. 2019;64:25-42.

15. Parkinson J, Minton J, Lewsey J, Bouttell J, McCartney G. Drug-related deaths in Scotland 1979–2013: evidence of a vulnerable cohort of young men living in deprived areas. BMC Public Health. 2018;18(1):357. doi: 10.1186/s12889-018-5267-2.

16. Parkinson J, Minton J, Lewsey J, Bouttell J, McCartney G. Recent cohort effects in suicide in Scotland: a legacy of the 1980s? Journal of Epidemiology and Community Health. 2017;71(2):194. doi: 10.1136/jech-2016-207296.

17. Marmot M. Social causes of the slowdown in health improvement. BMJ Publishing Group Ltd; 2018.

18. Hajizadeh M, Mitnitski A, Rockwood K. Socioeconomic gradient in health in Canada: Is the gap widening or narrowing? Health Policy. 2016;120(9):1040-50.

19. Walsh D, McCartney G, Minton J, Parkinson J, Shipton D, Whyte B. Deaths from ‘diseases of despair’ in Britain: comparing suicide, alcohol-related and drug-related mortality for birth cohorts in Scotland, England and Wales, and selected cities. Journal of Epidemiology and Community Health. 2021;75(12):1195. doi: 10.1136/jech-2020-216220.

20. Camarda CG. MortalitySmooth: An R package for smoothing Poisson counts with P-splines. Journal of Statistical Software. 2012;50:1-24.

21. Pace M, Lanzieri G, Glickman M, Zupanič T. Revision of the European Standard Population: report of Eurostat's task force: Publications Office of the European Union; 2013.

22. Kupper LL, Janis JM, Karmous A, Greenberg BG. Statistical age-period-cohort analysis: a review and critique. Journal of chronic diseases. 1985;38(10):811-30.

23. National Academies of Sciences E, Medicine. High and rising mortality rates among working-age adults2021.

24. Case A, Deaton A. Deaths of Despair and the Future of Capitalism. Deaths of Despair and the Future of Capitalism: Princeton University Press; 2020.

25. Zajacova A, Grol-Prokopczyk H, Zimmer Z. Pain trends among american adults, 2002–2018: Patterns, disparities, and correlates. Demography. 2021;58(2):711-38.

For peer review only

26. Masters RK, Tilstra AM, Simon DH. Mortality from suicide, chronic liver disease, and drug poisonings among middle-aged US white men and women, 1980–2013. Biodemography and social biology. 2017;63(1):31-7.

27. Alpert A, Evans WN, Lieber EMJ, Powell D. Origins of the Opioid Crisis and its Enduring Impacts*. The Quarterly Journal of Economics. 2022;137(2):1139-79. doi: 10.1093/qje/qjab043.

28. Masters RK, Tilstra AM, Simon DH. Explaining recent mortality trends among younger and middle-aged White Americans. International journal of epidemiology. 2017;47(1):81-8.

29. Ruhm CJ. Deaths of despair or drug problems? National Bureau of Economic Research, 2018.

30. Stannard C. Opioid prescribing in the UK: can we avert a public health disaster? British journal of pain. 2012;6(1):7.

31. Max MB, Donovan M, Miaskowski CA, Ward SE, Gordon D, Bookbinder M, et al. Quality improvement guidelines for the treatment of acute pain and cancer pain. Jama. 1995;274(23):1874-80.

32. Zin CS, Chen LC, Knaggs RD. Changes in trends and pattern of strong opioid prescribing in primary care. European journal of pain. 2014;18(9):1343-51.

33. Cooper AJ, Willis J, Fuller J, Benecke H, Leighton-Scott J, Andersohn F, et al. Prevalence and incidence trends for diagnosed prescription opioid use disorders in the United Kingdom. Pain and therapy. 2017;6(1):73-84.

34. Torjesen I. Fentanyl misuse in the UK: will we see a surge in deaths? bmj. 2018;361:k1564.

35. Jones JD, Mogali S, Comer SD. Polydrug abuse: a review of opioid and benzodiazepine combination use. Drug and alcohol dependence. 2012;125(1-2):8-18.

For peer review only

Figure Legends

Figure 1: All-cause mortality by age and sex in Canada, the UK and the USA, 2001-2019

Figure 2: Combined 'deaths of despair' mortality by age and sex in Canada, the UK and the USA, 2001-2019

Figure 2a – Drug-related mortality by age and sex in Canada, the UK and the USA, 2001-2019

Figure 2b - Suicide mortality by age and sex in Canada, the UK and the USA, 2001-2019

Figure 2c – Alcohol-specific mortality by age and sex in Canada, the UK and the USA, 2001-2019

For peer review only

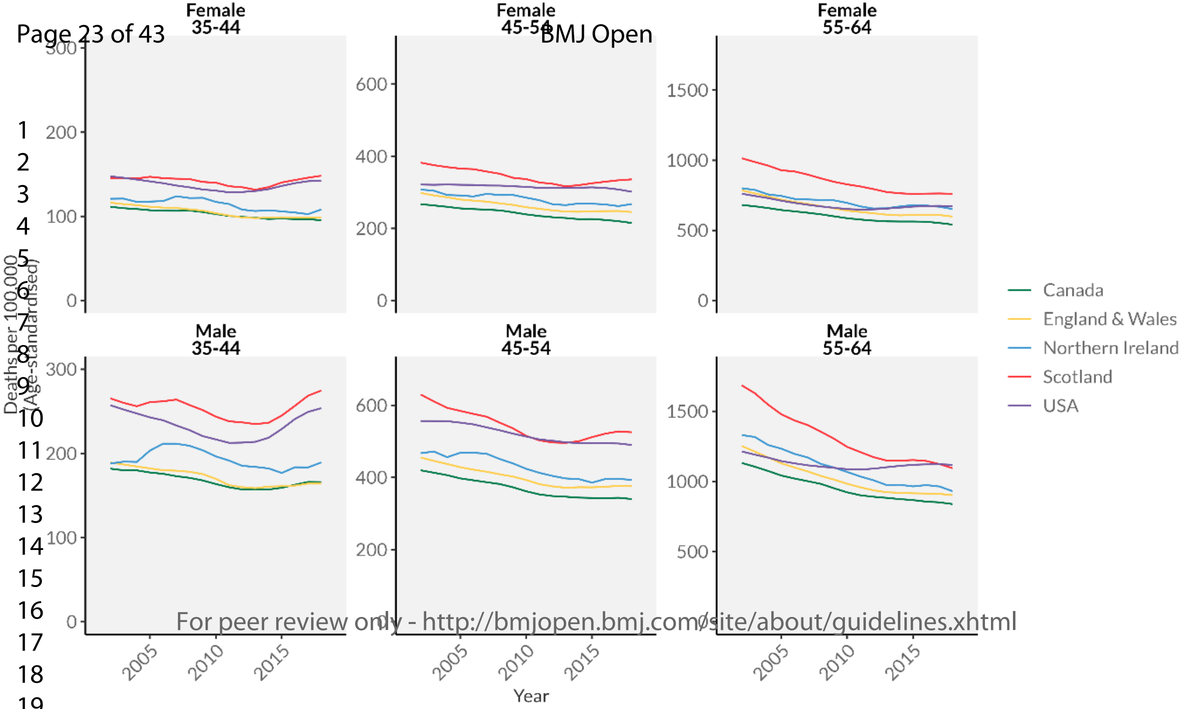

Female 35-44
Female 45-54
Female 55-64
Male 35-44
Male 45-54
Male 55-64
Deaths per 100,000 (Age-standardised)
Year
2005
2010
2015
Canada
England & Wales
Northern Ireland
Scotland
USA

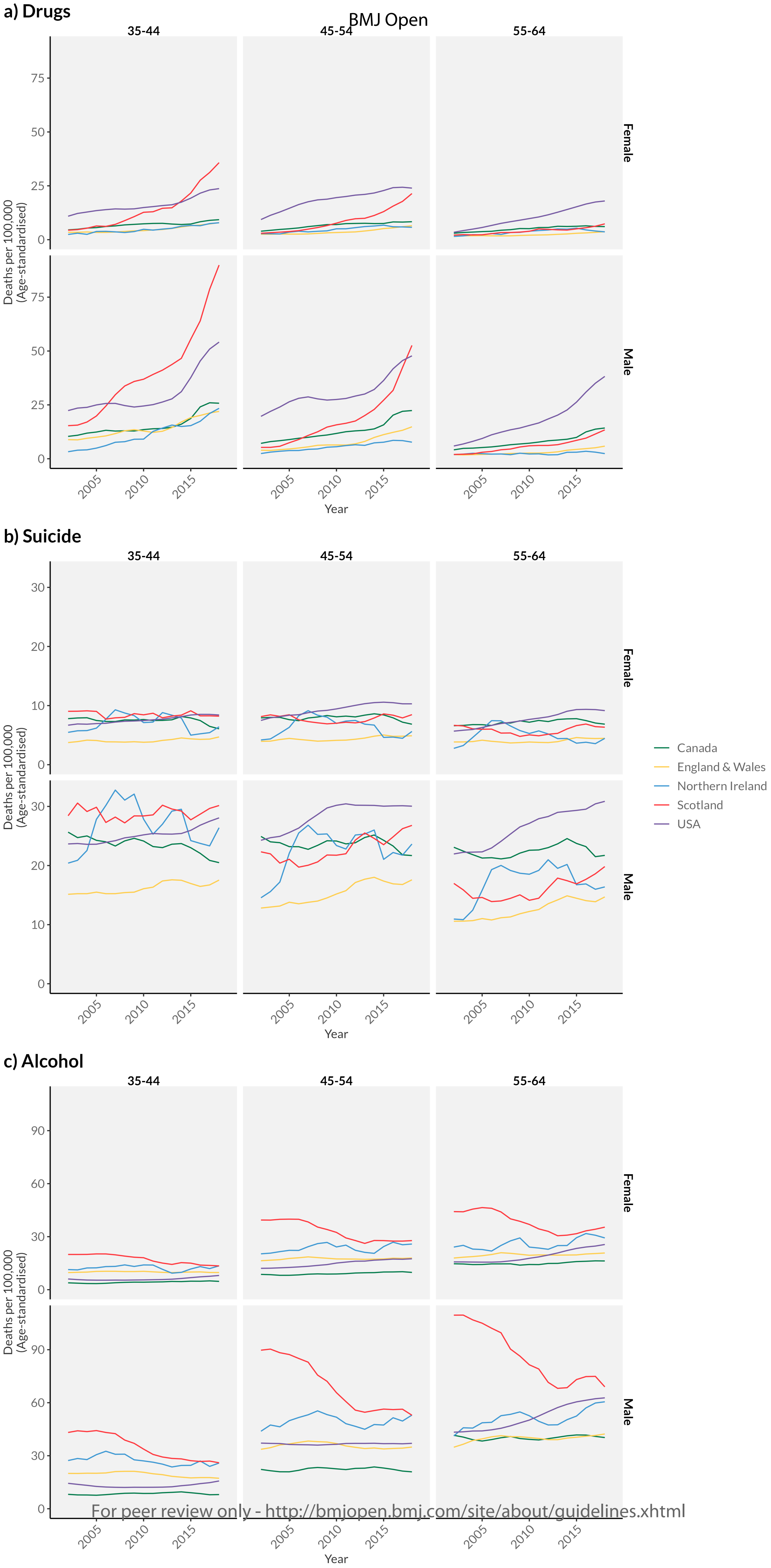
a) Drugs
b) Suicide
c) Alcohol
35-44
45-54
55-64
Female
Male
Deaths per 100,000
(Age-standardised)
Year
2005
2010
2015
Canada
England & Wales
Northern Ireland
Scotland
USA

Supplemental Figures and Tables

Figure S1 - Mortality from combined 'deaths of despair' by age and sex in Canada, the UK and the US, 2001-2019

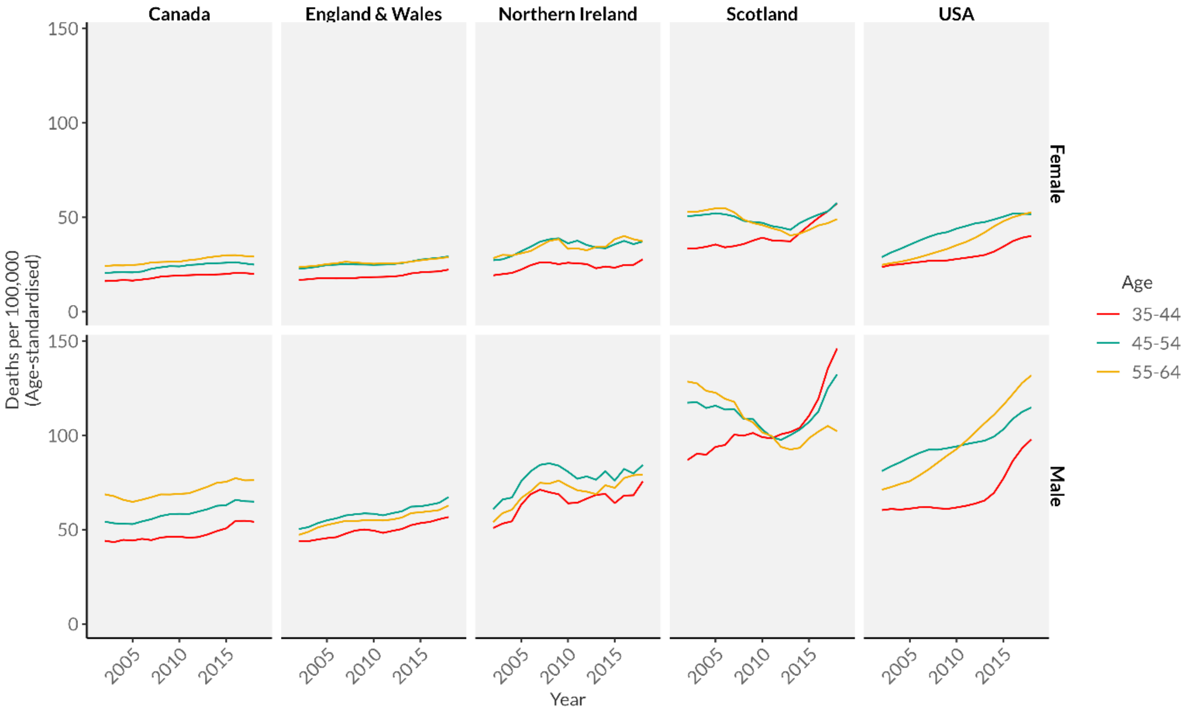

Figure S1a - Drug-related mortality by age and sex in Canada, the UK and the US, 2001-2019

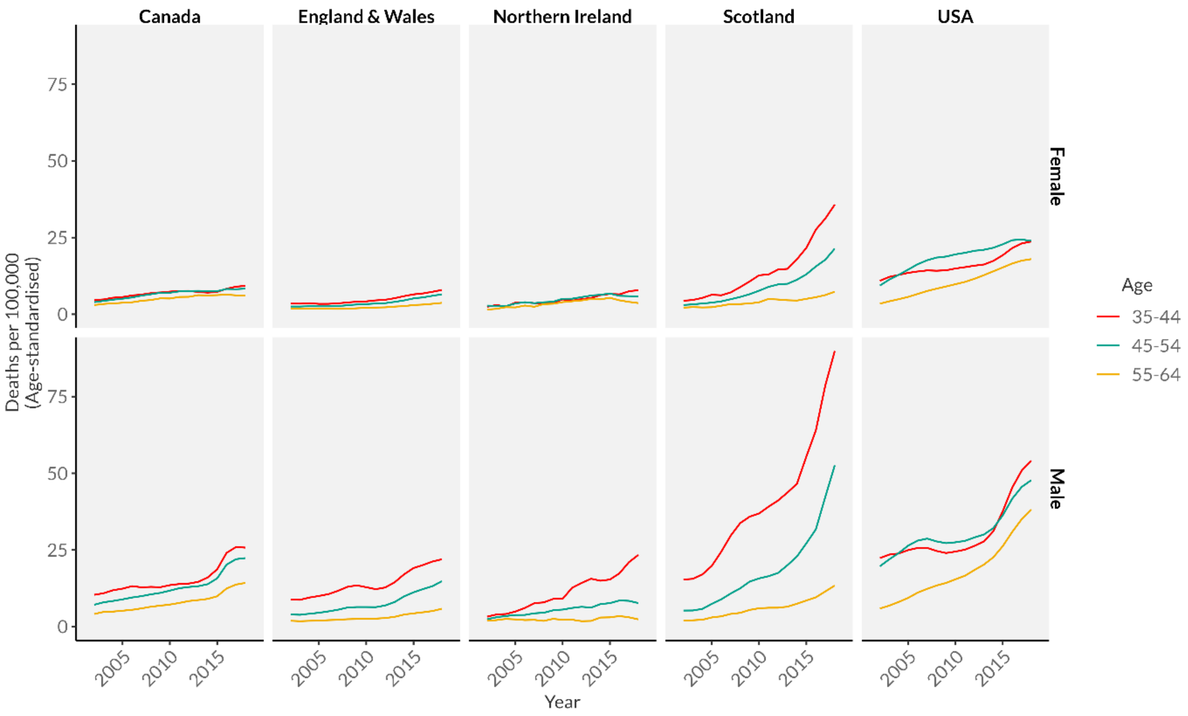

Figure S1b - Suicide mortality by age and sex in Canada, the UK and the US, 2001-2019

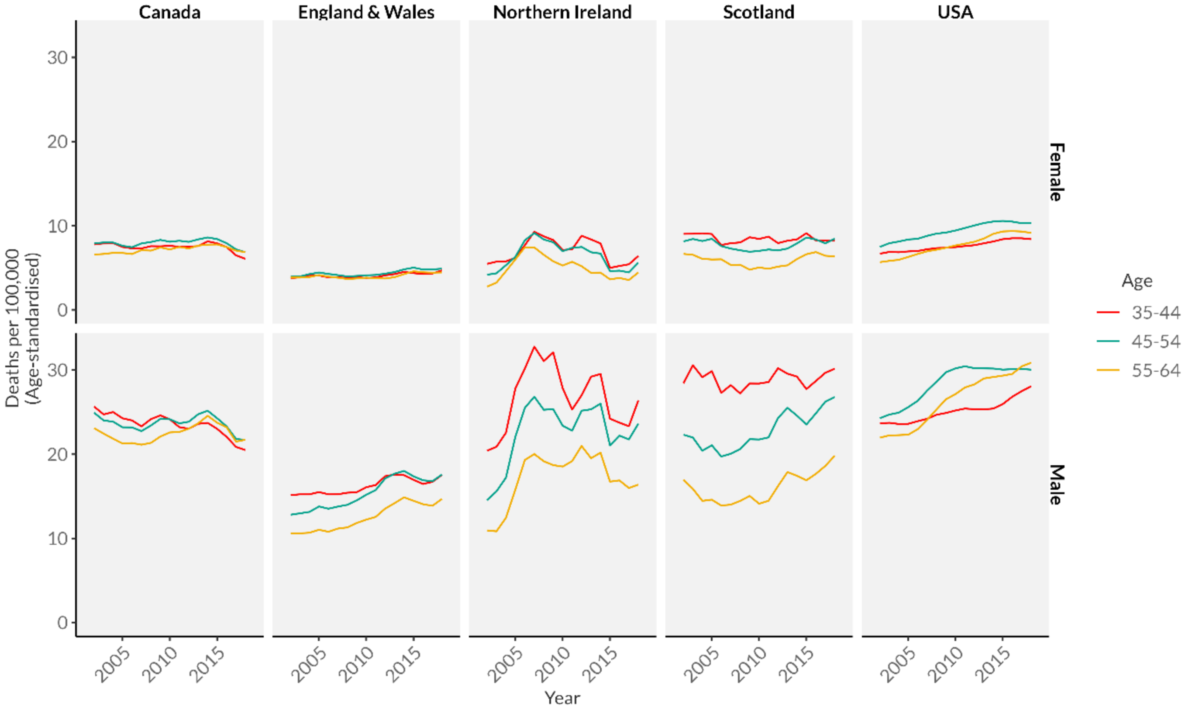

Figure S1c – alcohol-specific mortality by age and sex in Canada, the UK and the US, 2001-2019

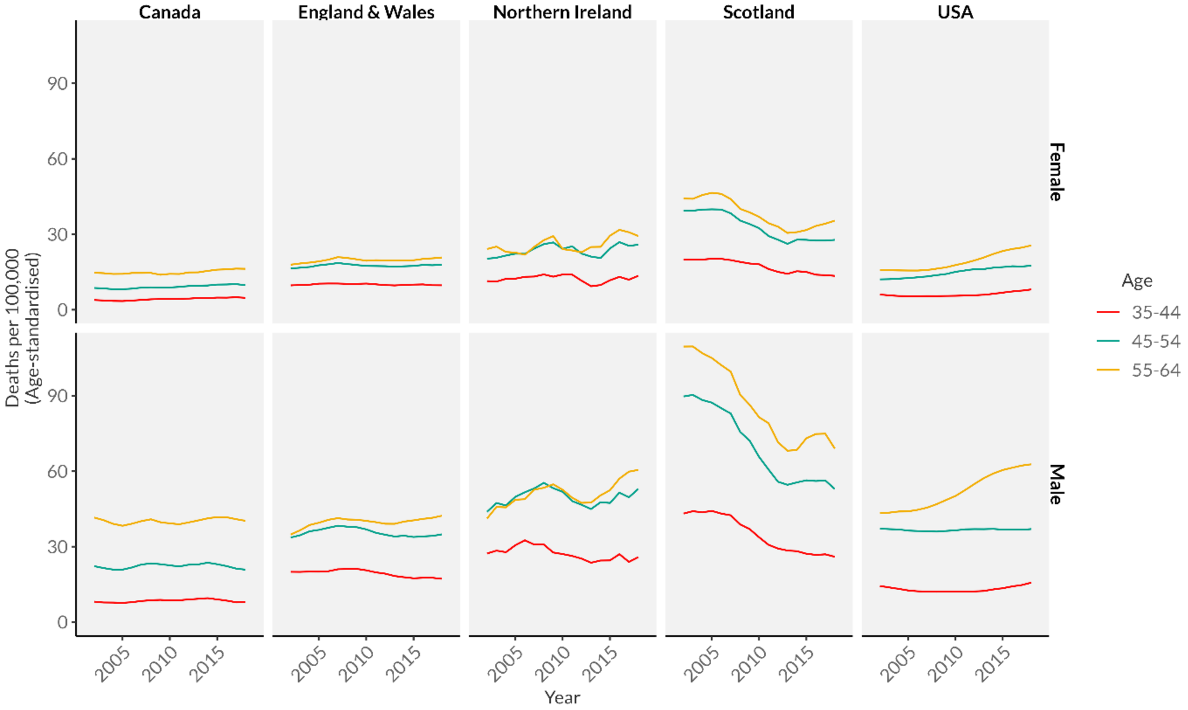

Figure S2a - Lexis surface showing age-specific mortality rates for males by cause, year and country

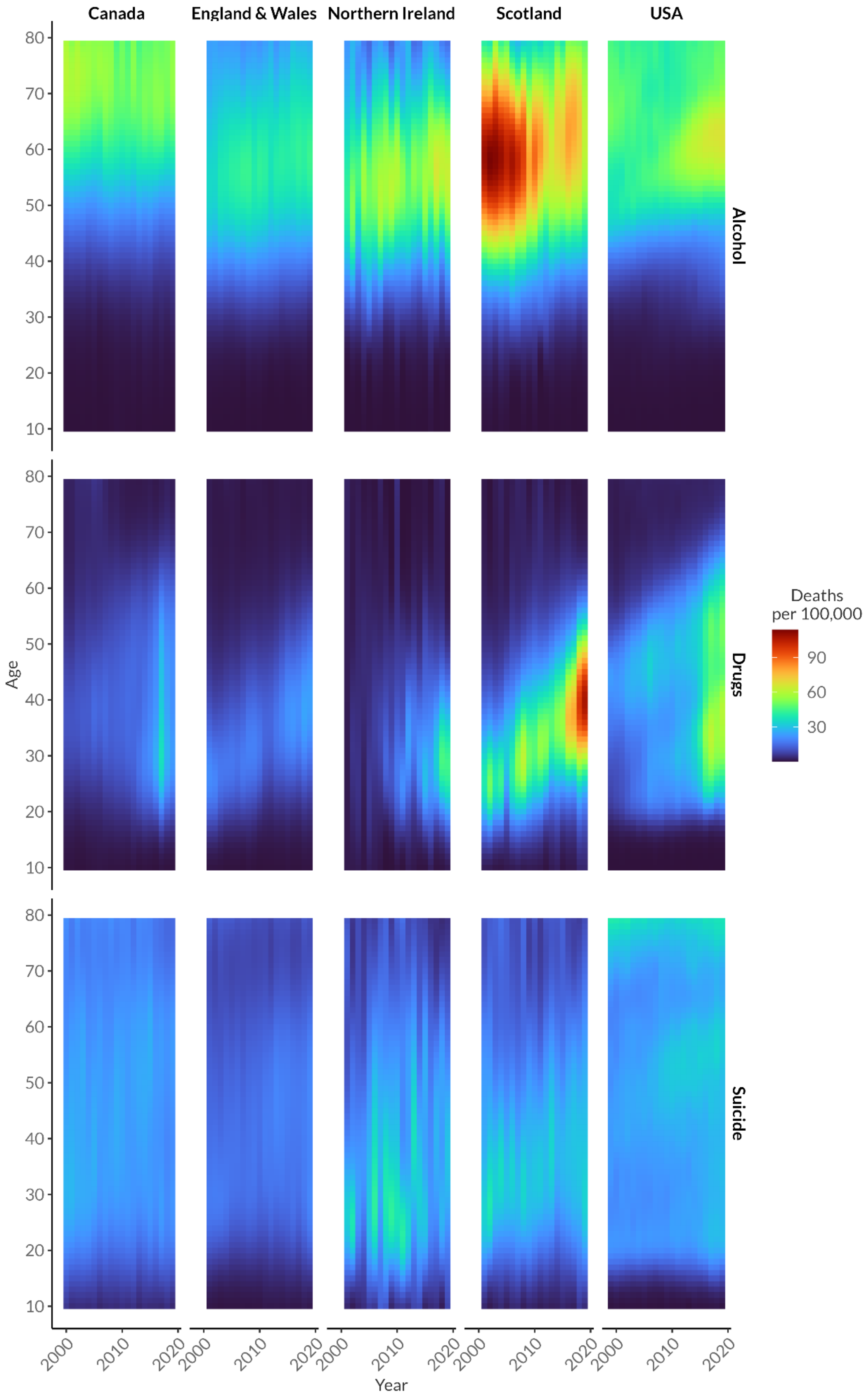

Figure S2b - Lexis surface showing age-specific mortality rates for females by cause, year and country

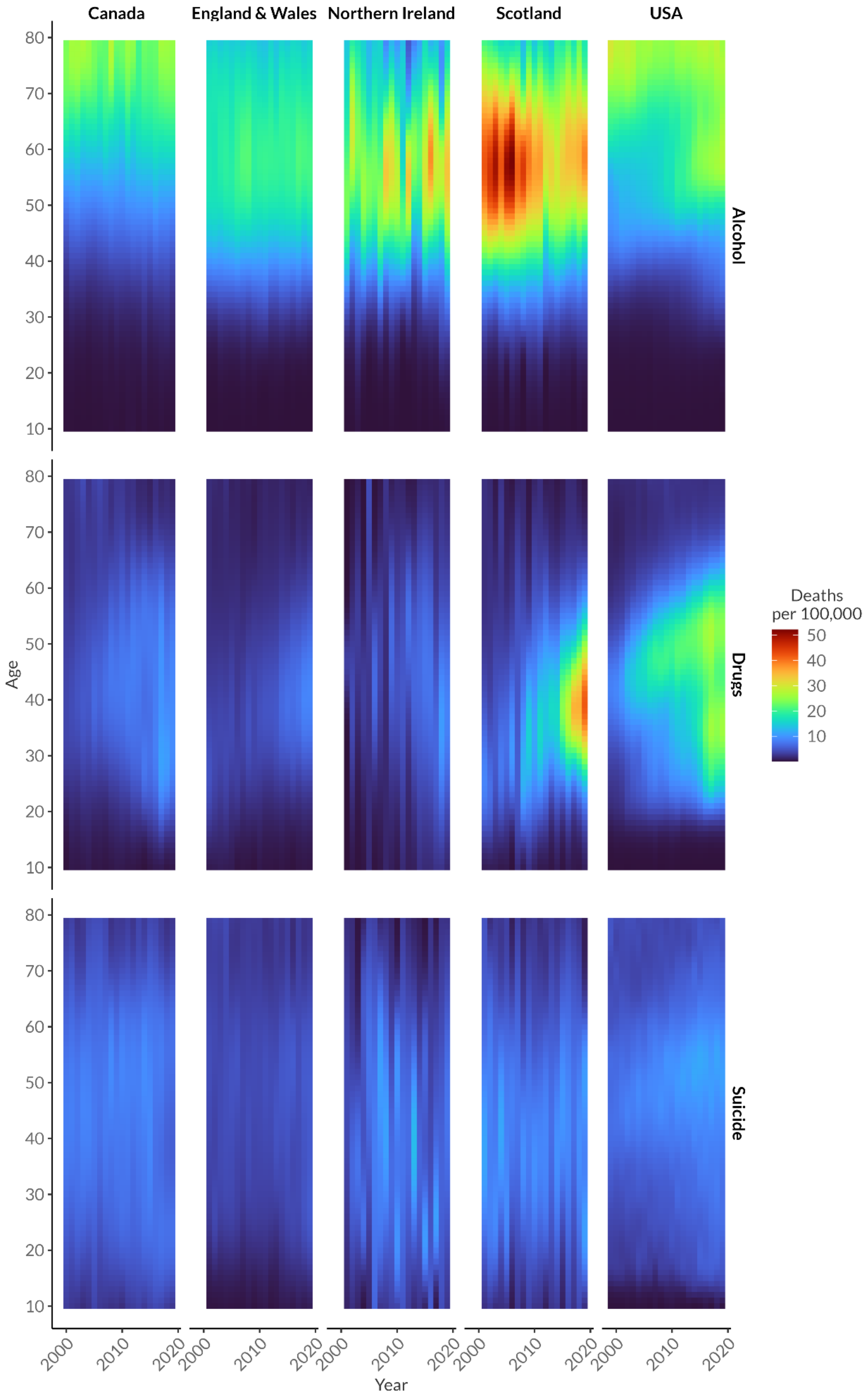

Table S1 - ICD-10 codes used to define 'deaths of despair' categories

**Cause group**	**ICD-10 codes included**
Suicide	U03, X60-84, Y87
Alcohol-related	K70, K73-74, F10, X45, Y15
Drug-related	F11-16, F18-19, X40-44, X85, Y10-14

Table S2a - Male mortality rates (per 100,000 population) by cause, age group and year for Canada

Age group	2001	2002	2003	2004	2005	2006	2007	2008	2009	2010	2011	2012	2013	2014	2015	2016	2017	2018	2019
All-Cause																			
35-44	183.6	179.4	182.6	177.9	179.1	174.9	173.3	170.6	169.0	164.1	157.8	157.5	156.2	157.2	158.0	162.4	167.9	167.9	162.0
45-54	424.3	419.3	415.8	404.1	399.2	388.9	388.2	383.5	373.5	361.3	350.2	347.6	347.7	344.4	340.3	344.6	342.0	344.2	334.8
55-64	1156.0	1135.9	1104.2	1075.0	1045.1	1006.9	1010.8	988.8	949.3	917.0	901.3	886.8	884.5	877.6	860.8	863.1	845.6	845.1	825.7
Combined 'Deaths of Despair'																			
35-44	44.4	43.8	44.3	42.3	47.4	43.2	44.9	45.5	47.2	46.3	45.5	45.6	47.3	49.6	51.1	51.6	61.4	51.3	50.1
45-54	53.7	54.5	54.8	51.1	53.6	54.3	55.4	57.1	59.4	58.2	57.8	59.0	62.1	61.6	64.3	63.2	70.0	62.3	62.6
55-64	67.9	69.9	68.6	65.0	64.0	65.4	68.5	68.0	69.8	68.3	69.0	70.5	73.6	74.2	76.8	75.2	80.0	73.3	75.5
Alcohol																			
35-44	8.2	8.0	8.2	7.2	7.9	7.8	8.2	9.1	8.9	8.5	8.6	9.0	9.6	9.2	9.8	8.2	7.7	7.9	8.4
45-54	22.7	22.2	21.8	20.5	20.5	21.7	23.0	24.0	23.0	22.1	22.6	21.7	24.2	23.0	23.8	22.3	20.7	20.9	21.0
55-64	42.5	42.3	39.9	39.5	37.9	37.6	41.9	41.0	39.7	38.5	39.6	38.7	40.7	41.9	41.2	42.2	41.7	38.9	40.2
Drugs																			
35-44	9.7	10.5	11.0	11.2	13.4	12.5	13.7	12.4	12.9	13.4	14.3	14.0	13.8	16.0	18.3	21.8	32.1	24.0	21.1
45-54	6.1	7.7	7.7	8.5	9.0	9.2	10.3	10.3	11.0	11.7	12.5	13.4	12.9	13.3	15.4	18.5	26.8	20.7	19.6
55-64	3.2	4.7	4.6	5.2	5.0	5.4	6.1	6.4	7.1	7.1	7.5	8.6	9.0	8.4	9.8	11.5	16.1	13.6	13.1
Suicide																			
35-44	26.6	25.3	25.1	23.8	26.1	22.9	23.1	24.0	25.4	24.4	22.6	22.6	23.8	24.4	23.0	21.7	21.5	19.4	20.6
45-54	24.9	24.5	25.4	22.1	24.1	23.4	22.1	22.8	25.4	24.4	22.6	23.9	25.0	25.3	25.2	22.3	22.4	20.7	22.0
55-64	22.3	22.9	24.1	20.3	21.1	22.3	20.5	20.5	23.0	22.7	22.0	23.2	23.9	23.9	25.9	21.5	22.3	20.7	22.1

Table S2b - Female mortality rates (per 100,000 population) by cause, age group and year in Canada

Age group	2001	2002	2003	2004	2005	2006	2007	2008	2009	2010	2011	2012	2013	2014	2015	2016	2017	2018	2019
All-Cause																			
35-44	112.1	111.7	110.5	106.9	108.8	106.3	105.2	108.2	107.3	100.2	100.6	100.8	96.3	98.9	95.4	98.6	96.1	95.5	94.6
45-54	268.6	268.4	264.8	257.6	257.2	251.8	252.1	251.8	246.4	235.9	234.3	232.9	226.2	227.2	223.4	227.3	219.6	213.2	212.6
55-64	685.8	685.2	672.6	657.5	647.5	631.1	629.3	617.5	597.5	586.7	576.6	569.2	564.0	560.8	566.1	564.0	555.1	540.0	530.0
Combined 'Deaths of Despair'																			
35-44	15.9	15.8	17.0	16.4	17.0	16.1	18.0	18.8	19.0	19.1	19.7	19.3	19.5	19.7	20.1	20.2	21.5	19.7	19.0
45-54	20.0	20.5	21.0	21.0	20.7	20.8	22.4	25.1	23.1	24.3	24.6	25.3	25.2	26.1	25.8	25.9	27.0	23.8	24.1
55-64	24.1	24.7	24.0	25.0	24.6	24.7	26.1	27.5	25.4	26.7	27.7	27.6	28.1	30.5	29.4	29.9	30.7	28.0	28.9
Alcohol																			
35-44	4.0	3.9	3.8	3.3	3.3	3.7	3.9	4.3	4.3	4.2	4.2	4.6	4.5	4.9	4.3	5.2	4.8	5.0	4.3
45-54	8.6	9.0	8.3	8.2	8.0	8.3	8.9	9.2	8.8	8.5	9.4	9.3	9.6	9.8	9.5	10.7	9.9	9.9	9.5
55-64	14.7	15.4	13.9	14.3	14.4	14.0	15.4	14.4	14.1	13.2	15.5	13.9	15.2	15.6	15.6	16.5	16.1	16.3	16.4
Drugs																			
35-44	4.1	4.5	5.0	4.9	6.2	5.7	6.5	7.0	7.1	7.2	7.8	7.6	7.4	6.8	7.0	8.1	9.9	8.9	9.0
45-54	3.4	4.0	4.4	4.7	5.3	5.3	5.9	7.1	6.6	7.3	7.1	7.9	7.6	7.3	7.6	7.7	9.5	7.3	8.2
55-64	2.5	3.1	3.4	3.6	3.6	4.0	4.1	5.0	4.8	5.7	4.9	6.3	5.8	6.5	6.1	6.1	7.1	5.3	5.8
Suicide																			
35-44	7.8	7.4	8.2	8.1	7.5	6.8	7.6	7.5	7.6	7.6	7.7	7.1	7.6	8.0	8.8	6.9	6.7	5.8	5.7
45-54	8.0	7.4	8.4	8.2	7.4	7.2	7.7	8.8	7.7	8.4	8.1	8.1	8.1	9.0	8.7	7.5	7.6	6.5	6.5
55-64	6.9	6.2	6.7	7.0	6.6	6.7	6.6	8.0	6.5	7.8	7.3	7.4	7.2	8.3	7.7	7.3	7.4	6.5	6.7

For peer review only - http://bmjopen.bmj.com/site/about/guidelines.xhtml

Table S3a - Male mortality rates (per 100,000 population) by cause, age group and year for England & Wales

Age group	2001	2002	2003	2004	2005	2006	2007	2008	2009	2010	2011	2012	2013	2014	2015	2016	2017	2018	2019
All-Cause																			
35-44	190.7	188.3	188.4	183.7	181.3	181.5	177.1	179.8	177.7	168.9	162.8	155.2	161.4	159.5	160.7	162.9	161.7	168.0	162.8
45-54	461.4	453.4	451.1	433.0	428.0	424.2	412.7	410.8	402.8	393.9	382.2	368.3	375.6	370.5	372.9	374.7	374.4	382.0	371.0
55-64	1280.1	1251.8	1226.2	1159.3	1129.0	1100.6	1070.2	1044.7	1003.7	991.9	954.9	929.1	926.8	913.1	917.4	916.9	907.3	910.9	888.6
Combined 'Deaths of Despair'																			
35-44	44.8	42.8	44.4	44.7	45.4	46.7	46.2	51.0	51.5	48.0	49.2	47.9	51.2	52.1	54.1	54.5	54.1	58.3	58.0
45-54	50.5	48.7	52.0	53.5	55.0	56.6	56.6	59.6	58.4	58.0	59.0	55.9	61.5	62.0	63.3	62.0	64.5	66.4	71.1
55-64	46.6	45.9	49.5	51.3	52.8	53.6	54.4	56.1	53.1	56.0	56.3	52.5	57.6	59.3	59.6	58.9	60.9	61.6	66.1
Alcohol																			
35-44	19.8	19.1	21.0	19.7	19.5	21.0	20.3	21.9	21.4	20.3	20.2	18.9	18.7	17.4	17.5	17.5	17.9	17.5	16.3
45-54	32.7	32.0	36.1	35.5	36.7	37.9	38.0	39.0	36.9	37.4	36.4	32.8	35.0	34.3	33.7	33.5	35.1	34.3	35.3
55-64	33.1	33.8	37.5	38.2	40.1	40.3	41.6	42.1	38.7	41.4	40.7	37.2	39.4	40.6	40.0	40.8	42.3	41.3	43.3
Drugs																			
35-44	9.8	8.2	8.6	9.5	10.4	10.1	11.3	13.6	14.0	12.8	11.8	12.1	14.4	16.9	19.9	20.6	19.9	23.3	22.8
45-54	4.6	3.7	3.6	4.3	4.8	4.5	5.6	6.4	6.7	6.1	6.3	6.6	7.8	9.7	12.1	11.8	12.9	15.0	16.6
55-64	2.3	1.8	1.6	1.9	2.2	1.9	2.3	2.4	2.6	2.5	2.8	2.6	3.1	3.9	4.8	4.3	5.0	6.0	6.5
Suicide																			
35-44	15.2	15.4	14.8	15.5	15.4	15.6	14.7	15.5	16.1	14.9	17.2	16.9	18.1	17.8	16.7	16.4	16.3	17.4	18.9
45-54	13.2	13.0	12.3	13.7	13.5	14.2	12.9	14.2	14.8	14.5	16.2	16.5	18.7	17.9	17.5	16.7	16.5	17.1	19.1
55-64	11.1	10.3	10.3	11.2	10.5	11.4	10.5	11.6	11.8	12.1	12.8	12.8	15.1	14.7	14.8	13.8	13.5	14.3	16.3

view only

Table S3b - Female mortality rates (per 100,000 population) by cause, age group and year in England & Wales

Age group	2001	2002	2003	2004	2005	2006	2007	2008	2009	2010	2011	2012	2013	2014	2015	2016	2017	2018	2019
All-Cause																			
35-44	117.5	115.0	116.4	112.2	111.6	110.4	109.3	110.2	105.6	104.8	100.9	97.4	97.8	98.8	99.0	99.3	97.8	99.2	98.7
45-54	302.5	297.0	295.1	282.4	281.3	276.6	273.2	272.8	262.3	260.4	253.5	249.5	246.7	244.2	247.4	251.2	245.3	248.1	243.0
55-64	802.5	784.8	770.3	735.2	722.1	700.6	687.0	680.8	651.2	641.2	629.7	623.5	612.7	600.7	608.3	621.5	599.8	608.3	587.8
Combined 'Deaths of Despair'																			
35-44	16.8	16.7	17.1	17.7	18.2	17.5	17.3	18.7	17.7	18.3	19.1	18.0	18.8	20.7	21.4	20.4	21.6	22.2	23.2
45-54	23.0	22.6	23.0	24.1	24.7	25.5	24.7	26.0	24.5	24.6	25.5	24.9	25.3	26.9	27.7	28.2	28.5	28.9	30.5
55-64	23.7	23.0	24.0	25.1	24.3	26.3	26.3	26.9	24.9	25.4	25.9	25.6	25.4	26.8	27.4	27.4	28.6	28.8	29.4
Alcohol																			
35-44	9.6	9.8	9.6	10.0	10.2	10.8	10.2	10.2	10.4	10.2	10.6	9.5	9.4	10.1	10.1	9.8	10.4	9.1	9.7
45-54	16.4	16.4	16.4	17.3	17.3	18.5	18.4	18.8	17.3	17.3	17.6	17.2	17.2	17.2	17.5	17.5	18.5	17.2	18.2
55-64	17.7	17.4	18.7	19.1	18.4	20.1	21.2	21.5	19.0	19.6	19.7	19.7	19.4	19.6	19.9	19.6	21.1	20.6	20.5
Drugs																			
35-44	3.5	3.4	3.5	3.4	3.8	2.9	3.4	4.4	3.7	4.3	4.6	4.7	4.9	6.2	6.7	6.6	7.1	8.3	8.4
45-54	2.5	2.5	2.6	2.5	2.9	2.4	2.5	3.1	3.2	3.3	3.5	3.6	3.7	4.8	5.0	5.7	5.8	6.6	7.1
55-64	1.9	1.8	1.8	1.8	1.9	1.9	1.6	1.8	2.0	2.1	2.2	2.2	2.3	2.9	2.8	3.2	3.4	3.5	4.2
Suicide																			
35-44	3.8	3.5	4.0	4.2	4.2	3.8	3.6	4.2	3.7	3.8	3.9	3.9	4.5	4.4	4.6	4.1	4.1	4.8	5.2
45-54	4.2	3.7	4.0	4.3	4.5	4.5	3.8	4.1	4.0	4.0	4.4	4.1	4.4	4.9	5.1	5.1	4.3	5.2	5.3
55-64	4.1	3.8	3.6	4.1	4.0	4.3	3.6	3.6	3.8	3.7	3.9	3.7	3.6	4.4	4.8	4.6	4.0	4.6	4.7

Table S4a - Male mortality rates (per 100,000 population) by cause, age group and year for Northern Ireland

Age group	2001	2002	2003	2004	2005	2006	2007	2008	2009	2010	2011	2012	2013	2014	2015	2016	2017	2018	2019
All-Cause																			
35-44	192.7	200.4	170.8	199.3	199.3	211.6	223.7	199.2	204.4	207.7	177.2	189.6	188.9	173.4	184.3	172.7	193.7	183.7	190.4
45-54	465.7	497.4	438.8	479.3	450.5	475.5	480.3	440.9	431.9	443.7	396.4	398.5	417.9	375.3	393.1	388.9	405.0	395.8	378.1
55-64	1324.3	1385.5	1282.8	1282.1	1214.4	1205.1	1170.8	1137.3	1067.7	1092.9	1042.6	971.1	1011.6	940.0	977.1	977.9	967.5	947.7	876.9
Combined 'Deaths of Despair'																			
35-44	47.6	60.2	45.0	54.7	63.8	71.6	71.1	71.1	67.5	67.9	56.7	68.4	74.4	62.6	70.0	59.9	74.7	70.4	81.9
45-54	54.9	70.2	57.4	70.5	73.3	83.4	86.3	83.5	85.9	82.6	73.9	74.7	86.3	68.5	88.3	71.5	86.9	81.0	85.0
55-64	50.0	57.0	55.2	64.2	62.5	73.8	74.8	76.1	72.6	79.6	67.9	65.3	77.3	64.1	79.3	73.3	79.6	83.7	74.5
Alcohol																			
35-44	26.7	34.7	20.5	30.1	32.8	28.9	35.9	27.6	29.1	26.3	25.8	26.8	23.1	21.0	29.4	23.3	28.4	20.2	29.0
45-54	41.2	51.3	39.1	51.7	48.3	49.5	57.1	52.9	56.2	50.8	48.5	45.2	46.3	43.4	53.3	45.4	55.8	47.7	55.5
55-64	38.1	44.0	41.5	52.0	43.5	50.5	52.7	54.7	52.8	56.8	48.2	43.4	50.6	48.5	52.1	56.7	62.3	60.5	58.8
Drugs																			
35-44	1.6	4.1	4.1	3.7	4.6	6.5	7.2	9.2	7.3	10.6	9.4	18.0	15.3	13.6	16.1	16.5	19.6	26.9	23.6
45-54	1.8	2.2	3.2	3.8	3.5	4.2	3.8	5.1	4.7	6.3	5.8	6.3	7.4	5.0	9.7	8.3	7.5	9.4	6.2
55-64	2.0	1.2	2.5	2.6	2.4	2.0	2.0	2.6	1.0	4.1	1.5	1.2	2.6	1.7	4.6	2.8	3.0	3.4	0.8
Suicide																			
35-44	19.4	21.4	20.4	20.8	26.4	36.2	27.9	34.2	31.1	31.0	21.4	23.5	36.0	28.1	24.5	20.1	26.6	23.2	29.3
45-54	11.9	16.7	15.0	15.0	21.5	29.7	25.3	25.5	25.0	25.5	19.6	23.3	32.6	20.1	25.3	17.7	23.6	24.0	23.4
55-64	9.9	11.8	11.2	9.6	16.6	21.3	20.1	18.7	18.7	18.7	18.2	20.6	24.1	13.8	22.6	13.8	14.3	19.9	15.0

eview only

Table S4b - Female mortality rates (per 100,000 population) by cause, age group and year in Northern Ireland

Age group	2001	2002	2003	2004	2005	2006	2007	2008	2009	2010	2011	2012	2013	2014	2015	2016	2017	2018	2019
All-Cause																			
35-44	119.4	121.7	121.7	119.8	110.2	122.8	121.5	127.5	116.4	122.3	112.9	108.8	102.3	107.8	111.8	98.3	103.7	105.9	115.0
45-54	310.2	311.2	301.8	300.3	275.8	298.1	292.2	297.0	288.4	292.7	274.7	267.2	259.3	267.3	281.2	257.1	261.0	267.6	274.0
55-64	809.9	819.0	772.8	774.6	722.8	740.3	711.3	710.9	727.4	704.9	656.3	652.0	659.5	663.0	689.5	688.4	654.1	663.1	639.7
Combined 'Deaths of Despair'																			
35-44	20.1	18.3	19.4	22.1	20.1	24.8	28.9	24.6	24.8	26.3	26.8	23.7	25.3	20.0	26.5	23.5	24.3	26.7	32.3
45-54	29.4	26.6	25.8	30.9	31.8	33.5	37.9	39.8	37.3	39.6	31.8	41.5	32.7	27.9	40.2	39.1	33.3	35.0	43.3
55-64	24.6	32.6	28.0	29.8	31.9	31.2	33.3	40.2	39.0	36.3	24.7	39.6	33.3	30.1	39.6	45.6	35.2	34.4	42.5
Alcohol																			
35-44	15.0	8.5	10.7	14.4	11.8	10.9	16.6	12.2	13.4	13.9	14.7	13.2	6.7	8.1	14.6	12.4	12.2	11.2	17.1
45-54	22.9	19.4	18.6	24.0	22.0	20.8	23.9	28.3	26.0	25.9	20.6	29.1	17.2	17.1	27.4	28.7	24.5	23.1	29.9
55-64	20.8	28.2	23.4	23.7	21.8	22.6	21.2	31.3	30.4	26.1	15.8	28.8	24.0	22.0	29.0	37.2	29.1	26.1	32.7
Drugs																			
35-44	1.2	4.0	2.0	3.0	2.4	6.0	3.1	1.7	5.1	4.4	4.9	4.0	5.9	6.1	6.8	7.0	5.6	9.8	8.2
45-54	2.4	3.1	2.9	2.3	2.8	5.4	3.6	1.9	6.3	4.2	4.7	6.3	5.7	6.2	7.1	6.9	4.0	6.9	6.4
55-64	0.2	1.6	2.7	1.0	3.2	2.5	2.8	2.0	5.2	3.1	3.6	6.0	3.8	5.1	5.7	5.2	2.6	4.2	4.1
Suicide																			
35-44	3.9	5.8	6.7	4.7	5.9	7.9	9.2	10.7	6.3	7.9	7.2	6.6	12.7	5.8	5.1	4.0	6.5	5.7	7.0
45-54	4.1	4.2	4.3	4.6	7.1	7.3	10.5	9.7	5.0	9.5	6.5	6.2	9.8	4.6	5.7	3.6	4.7	5.1	7.0
55-64	3.6	2.8	1.9	5.1	6.9	6.1	9.4	6.8	3.5	7.1	5.3	4.8	5.5	3.0	4.9	3.1	3.4	4.1	5.8

Table S5a - Male mortality rates (per 100,000 population) by cause, age group and year for Scotland

Age group	2001	2002	2003	2004	2005	2006	2007	2008	2009	2010	2011	2012	2013	2014	2015	2016	2017	2018	2019
All-Cause																			
35-44	274.9	267.0	254.2	259.0	254.9	268.9	262.0	261.1	249.4	244.0	237.5	232.9	240.2	231.4	237.8	265.8	266.1	273.8	284.2
45-54	649.0	634.1	606.2	591.9	580.9	580.7	567.1	557.3	530.8	520.1	495.1	493.0	502.7	491.9	507.0	536.6	520.6	525.7	529.8
55-64	1708.3	1712.4	1635.3	1538.6	1468.8	1429.7	1406.5	1370.3	1287.4	1257.4	1194.7	1175.9	1149.4	1121.3	1172.9	1163.9	1101.5	1106.0	1076.2
Combined 'Deaths of Despair'																			
35-44	82.2	91.2	87.4	92.3	89.9	99.7	95.2	106.4	98.0	99.5	99.7	96.2	106.0	103.2	102.9	125.2	130.2	150.5	157.5
45-54	114.9	120.5	116.5	115.7	111.3	120.3	109.5	111.6	105.1	109.1	95.3	92.9	104.6	102.9	101.5	116.9	119.6	138.1	139.2
55-64	127.9	131.0	126.7	125.0	119.1	123.7	115.5	114.0	98.4	108.3	98.0	92.8	90.9	93.9	95.4	106.3	104.3	104.4	98.0
Alcohol																			
35-44	40.9	43.8	44.8	43.8	42.4	46.5	40.6	40.4	35.6	35.0	30.6	26.6	30.6	28.2	25.8	27.6	26.9	26.4	24.7
45-54	86.1	91.1	92.0	87.9	85.0	88.9	81.3	78.7	67.0	70.8	59.3	51.9	56.2	55.7	54.8	58.7	54.9	55.4	48.5
55-64	107.3	110.7	110.7	107.4	102.6	105.0	98.9	94.8	77.3	87.0	80.0	70.2	64.4	69.8	71.3	78.2	74.9	71.7	60.4
Drugs																			
35-44	15.1	16.8	14.1	15.9	21.1	22.5	29.7	36.9	34.8	36.0	40.0	41.4	42.0	47.8	50.0	68.7	73.3	94.0	102.4
45-54	5.5	5.5	4.7	5.6	7.1	9.7	10.0	12.8	14.7	16.6	15.9	16.9	19.8	23.5	25.4	32.7	37.1	57.1	63.6
55-64	2.3	1.7	2.0	2.6	2.1	4.3	3.7	4.5	5.3	6.6	6.1	5.8	6.6	7.2	8.6	9.8	10.3	14.4	15.6
Suicide																			
35-44	26.2	30.6	28.4	32.6	26.3	30.6	24.9	29.1	27.7	28.5	29.1	28.2	33.4	27.1	27.2	28.9	30.0	30.1	30.4
45-54	23.2	23.9	19.8	22.2	19.2	21.8	18.2	20.2	23.4	21.7	20.1	24.2	28.6	23.7	21.4	25.5	27.7	25.6	27.1
55-64	18.3	18.6	14.0	15.0	14.4	14.4	12.9	14.7	15.8	14.7	11.9	16.8	19.9	16.9	15.5	18.3	19.2	18.3	22.0

Table S5b - Female mortality rates (per 100,000 population) by cause, age group and year in Scotland

Age group	2001	2002	2003	2004	2005	2006	2007	2008	2009	2010	2011	2012	2013	2014	2015	2016	2017	2018	2019
All-Cause																			
35-44	149.2	147.2	139.8	148.5	146.7	146.0	143.7	144.5	144.9	133.4	142.0	132.1	129.8	133.1	141.3	146.4	141.8	149.7	153.4
45-54	387.9	382.8	376.1	366.9	367.0	363.6	362.0	347.5	344.8	328.6	336.3	317.0	317.7	314.8	327.2	333.5	327.8	338.9	341.1
55-64	1014.3	1009.6	1019.0	934.6	932.2	919.3	909.7	865.6	838.1	835.8	810.4	794.0	778.8	748.0	766.4	766.3	754.3	769.4	759.9
Combined 'Deaths of Despair'																			
35-44	35.6	32.6	32.0	36.4	34.9	35.6	31.9	36.8	38.9	37.4	41.4	34.7	36.5	40.7	47.7	49.0	52.6	58.0	61.7
45-54	48.4	51.2	52.0	49.9	52.5	54.0	48.4	49.2	47.0	46.4	48.0	41.9	44.0	44.4	52.6	51.3	50.5	57.8	64.7
55-64	49.9	53.1	55.9	49.9	55.8	58.5	50.3	49.2	46.9	44.9	45.9	42.1	40.9	38.1	45.0	46.8	45.3	48.6	53.3
Alcohol																			
35-44	19.9	20.6	19.3	19.9	20.7	20.2	19.8	19.2	18.0	17.9	18.4	12.3	14.5	16.1	15.3	13.7	12.8	14.8	12.8
45-54	36.7	40.2	41.4	36.5	41.5	41.7	36.3	36.9	33.1	32.2	32.0	23.7	27.7	27.2	28.7	27.5	26.3	28.7	28.4
55-64	41.3	43.4	47.9	41.0	47.9	50.4	39.8	41.7	38.8	35.6	36.4	31.1	31.5	28.9	32.2	34.0	33.7	34.9	37.5
Drugs																			
35-44	4.8	3.7	4.7	5.6	5.7	7.8	5.0	8.5	13.0	10.5	14.6	13.8	15.4	15.3	23.2	26.5	33.1	34.0	40.2
45-54	2.8	2.7	3.5	3.5	3.4	4.4	4.8	5.5	6.6	7.5	9.0	10.4	9.8	9.6	14.3	15.2	17.2	20.9	26.1
55-64	2.0	2.2	2.2	2.7	1.8	2.4	4.1	3.4	2.4	4.7	4.6	5.7	4.1	3.9	5.4	5.8	5.5	7.5	9.0
Suicide																			
35-44	10.9	8.2	7.9	11.0	8.4	7.6	7.1	9.1	7.9	8.9	8.5	8.7	6.6	9.3	9.2	8.8	6.8	9.2	8.7
45-54	9.0	8.3	7.1	9.9	7.6	7.9	7.3	6.7	7.3	6.7	7.0	7.8	6.4	7.6	9.6	8.5	7.0	8.2	10.2
55-64	6.6	7.5	5.9	6.3	6.0	5.7	6.3	4.0	5.7	4.6	4.8	5.3	5.4	5.3	7.5	7.1	6.0	6.2	6.8

For peer review only - http://bmjopen.bmj.com/site/about/guidelines.xhtml

Table S6a - Male mortality rates (per 100,000 population) by cause, age group and year for the USA

Age group	2001	2002	2003	2004	2005	2006	2007	2008	2009	2010	2011	2012	2013	2014	2015	2016	2017	2018	2019
All-Cause																			
35-44	259.2	258.0	254.3	244.0	244.0	240.4	233.5	225.5	223.9	212.3	213.6	211.8	212.9	216.6	226.2	244.2	252.2	251.7	257.3
45-54	551.3	557.1	561.1	551.0	556.2	548.7	536.6	531.3	524.2	508.9	507.4	500.0	497.9	494.0	494.0	496.7	495.4	490.2	484.9
55-64	1226.1	1216.1	1200.0	1157.0	1153.1	1126.4	1108.7	1110.6	1098.0	1082.8	1081.4	1090.8	1101.8	1112.5	1119.2	1124.2	1122.5	1124.4	1106.7
Combined 'Deaths of Despair'																			
35-44	58.2	61.6	61.4	60.1	60.5	63.0	62.1	61.0	61.2	61.0	63.2	63.8	64.6	68.1	76.0	87.3	96.0	96.2	101.6
45-54	77.2	81.2	84.9	84.9	87.8	92.5	91.6	93.2	92.5	94.1	95.9	96.0	96.9	98.7	102.5	108.4	115.2	113.7	115.5
55-64	68.7	71.7	73.2	73.0	76.8	77.5	81.9	86.5	88.6	93.0	96.7	101.3	107.0	111.1	114.6	122.6	128.4	132.3	134.8
Alcohol																			
35-44	14.9	14.3	14.0	13.1	12.6	12.1	12.1	12.1	11.9	12.4	12.1	12.0	12.6	12.7	13.8	14.0	14.8	15.5	16.9
45-54	36.9	36.8	37.8	36.4	36.5	36.2	36.0	36.2	35.7	36.8	36.8	37.1	37.0	36.8	37.4	36.3	36.9	37.0	37.1
55-64	42.9	43.5	43.7	43.5	44.8	43.8	45.3	47.5	47.9	50.3	52.3	54.8	57.6	59.3	60.4	61.7	62.1	63.0	63.2
Drugs																			
35-44	20.1	23.2	23.8	23.5	24.2	27.1	25.6	24.3	24.1	23.6	25.7	26.0	27.2	30.1	36.0	46.9	53.2	52.6	56.6
45-54	16.7	19.9	22.5	23.5	26.0	29.8	28.4	28.0	27.0	26.7	28.8	28.4	30.1	31.7	34.7	42.6	47.9	46.2	49.2
55-64	4.6	6.1	7.0	7.5	9.7	11.0	12.6	13.3	14.3	15.1	16.9	17.8	20.7	22.2	25.0	31.7	36.1	37.4	41.1
Suicide																			
35-44	23.3	24.1	23.6	23.5	23.7	23.7	24.4	24.5	25.1	25.1	25.4	25.8	24.8	25.3	26.1	26.4	28.0	28.1	28.1
45-54	23.7	24.6	24.5	25.0	25.3	26.5	27.2	28.9	29.7	30.6	30.3	30.5	29.9	30.2	30.4	29.5	30.4	30.5	29.3
55-64	21.2	22.2	22.5	22.0	22.3	22.7	24.0	25.6	26.4	27.6	27.5	28.7	28.7	29.6	29.2	29.2	30.2	31.9	30.5

Table S6b - Female mortality rates (per 100,000 population) by cause, age group and year in the USA

Age group	2001	2002	2003	2004	2005	2006	2007	2008	2009	2010	2011	2012	2013	2014	2015	2016	2017	2018	2019
All-Cause																			
35-44	147.6	148.2	146.9	142.4	142.0	140.4	135.8	133.7	134.0	128.1	129.6	127.9	129.4	133.1	134.6	141.0	142.9	141.6	142.8
45-54	319.8	321.9	322.5	318.1	323.8	320.7	317.2	318.8	319.9	311.7	314.4	311.1	311.6	313.9	313.5	314.1	308.8	301.0	295.1
55-64	773.4	758.0	753.7	724.8	713.0	697.9	677.4	673.1	662.4	647.9	647.4	649.3	653.5	664.3	668.9	675.4	678.4	671.6	667.8
Combined 'Deaths of Despair'																			
35-44	22.0	24.3	24.6	25.4	25.4	26.7	26.9	27.2	26.8	27.7	29.4	28.7	29.7	31.8	34.1	37.6	40.4	39.6	40.5
45-54	26.3	29.3	31.2	33.7	35.1	37.8	40.1	41.0	42.7	42.8	46.6	46.7	47.2	48.6	50.9	51.6	53.4	50.6	51.2
55-64	24.1	24.8	25.7	26.8	27.0	28.8	30.7	31.4	33.3	35.1	37.2	38.6	42.0	45.5	48.2	50.2	51.8	52.5	53.8
Alcohol																			
35-44	6.4	6.1	5.7	5.3	5.4	5.5	5.3	5.5	5.4	5.5	5.7	5.8	5.8	6.3	6.9	7.2	7.6	7.9	8.6
45-54	12.1	12.0	12.1	12.4	12.6	12.8	13.3	13.7	14.3	14.6	16.3	15.9	16.1	16.4	17.6	16.9	17.5	17.3	17.7
55-64	15.8	15.7	15.7	15.7	15.5	15.6	15.7	16.1	17.2	17.3	18.8	19.6	20.4	22.4	23.7	24.0	24.7	25.3	26.7
Drugs																			
35-44	9.2	11.3	12.3	13.0	13.2	14.3	14.3	14.3	13.9	14.7	16.1	15.2	16.2	17.2	18.8	21.8	24.2	23.3	23.6
45-54	7.0	9.8	11.3	12.8	14.4	16.5	18.0	18.3	19.1	19.0	20.5	20.6	21.0	21.5	22.6	24.4	25.5	23.1	23.2
55-64	2.6	3.5	4.2	4.9	5.5	6.5	7.8	8.4	8.8	9.9	10.7	11.0	13.0	14.2	14.8	16.8	18.1	17.6	18.2
Suicide																			
35-44	6.5	6.9	6.7	7.1	6.8	7.0	7.3	7.4	7.4	7.4	7.6	7.8	7.7	8.3	8.4	8.5	8.6	8.4	8.2
45-54	7.2	7.5	7.8	8.5	8.1	8.5	8.8	9.1	9.3	9.2	9.8	10.2	10.1	10.7	10.7	10.3	10.4	10.2	10.3
55-64	5.7	5.6	5.8	6.1	6.0	6.8	7.2	6.9	7.4	7.9	7.7	8.0	8.6	8.9	9.7	9.4	9.0	9.5	8.9

The RECORD statement – checklist of items, extended from the STROBE statement, that should be reported in observational studies using routinely collected health data.

	Item No.	STROBE items	Location in manuscript where items are reported	RECORD items	Location in manuscript where items are reported
**Title and abstract**					
	1	(a) Indicate the study's design with a commonly used term in the title or the abstract (b) Provide in the abstract an informative and balanced summary of what was done and what was found		RECORD 1.1: The type of data used should be specified in the title or abstract. When possible, the name of the databases used should be included.<br><br>RECORD 1.2: If applicable, the geographic region and timeframe within which the study took place should be reported in the title or abstract.<br><br>RECORD 1.3: If linkage between databases was conducted for the study, this should be clearly stated in the title or abstract.	Title and Abstract- we use publicly available death counts, so not individual level data
**Introduction**					
Background rationale	2	Explain the scientific background and rationale for the investigation being reported			p 1-4
Objectives	3	State specific objectives, including any prespecified hypotheses			p 1-4
**Methods**					
Study Design	4	Present key elements of study design early in the paper			p 5
Setting	5	Describe the setting, locations, and relevant dates, including periods of recruitment, exposure, follow-up, and data collection			p 5

For peer review only - http://bmjopen.bmj.com/site/about/guidelines.xhtml

Participants	6	*(a) Cohort study* - Give the eligibility criteria, and the sources and methods of selection of participants. Describe methods of follow-up<br>*Case-control study* - Give the eligibility criteria, and the sources and methods of case ascertainment and control selection. Give the rationale for the choice of cases and controls<br>*Cross-sectional study* - Give the eligibility criteria, and the sources and methods of selection of participants<br><br>*(b) Cohort study* - For matched studies, give matching criteria and number of exposed and unexposed<br>*Case-control study* - For matched studies, give matching criteria and the number of controls per case		RECORD 6.1: The methods of study population selection (such as codes or algorithms used to identify subjects) should be listed in detail. If this is not possible, an explanation should be provided.<br><br>RECORD 6.2: Any validation studies of the codes or algorithms used to select the population should be referenced. If validation was conducted for this study and not published elsewhere, detailed methods and results should be provided.<br><br>RECORD 6.3: If the study involved linkage of databases, consider use of a flow diagram or other graphical display to demonstrate the data linkage process, including the number of individuals with linked data at each stage.	N/A– We use national level death counts by age and sex, no individual records.
Variables	7	Clearly define all outcomes, exposures, predictors, potential confounders, and effect modifiers. Give diagnostic criteria, if applicable.		RECORD 7.1: A complete list of codes and algorithms used to classify exposures, outcomes, confounders, and effect modifiers should be provided. If these cannot be reported, an explanation should be provided.	N/A– descriptive analysis of mortality trends. Full code included and available.
Data sources/ measurement	8	For each variable of interest, give sources of data and details of methods of assessment (measurement).<br>Describe comparability of assessment methods if there is more than one group			

For peer review only

Bias	9	Describe any efforts to address potential sources of bias			
Study size	10	Explain how the study size was arrived at			Full national statistics
Quantitative variables	11	Explain how quantitative variables were handled in the analyses. If applicable, describe which groupings were chosen, and why			
Statistical methods	12	(a) Describe all statistical methods, including those used to control for confounding<br>(b) Describe any methods used to examine subgroups and interactions<br>(c) Explain how missing data were addressed<br>(d) *Cohort study* - If applicable, explain how loss to follow-up was addressed<br>*Case-control study* - If applicable, explain how matching of cases and controls was addressed<br>*Cross-sectional study* - If applicable, describe analytical methods taking account of sampling strategy<br>(e) Describe any sensitivity analyses			N/A- descriptive analysis of national mortality trends, not individual level data.
Data access and cleaning methods		..		RECORD 12.1: Authors should describe the extent to which the investigators had access to the database population used to create the study population.	N/A– publicly available national death counts

For peer review only

				RECORD 12.2: Authors should provide information on the data cleaning methods used in the study.	
Linkage		..		RECORD 12.3: State whether the study included person-level, institutional-level, or other data linkage across two or more databases. The methods of linkage and methods of linkage quality evaluation should be provided.	N/A
**Results**					
Participants	13	(a) Report the numbers of individuals at each stage of the study (*e.g.*, numbers potentially eligible, examined for eligibility, confirmed eligible, included in the study, completing follow-up, and analysed)<br>(b) Give reasons for non-participation at each stage.<br>(c) Consider use of a flow diagram		RECORD 13.1: Describe in detail the selection of the persons included in the study (*i.e.,* study population selection) including filtering based on data quality, data availability and linkage. The selection of included persons can be described in the text and/or by means of the study flow diagram.	N/A– publicly available national death counts
Descriptive data	14	(a) Give characteristics of study participants (*e.g.*, demographic, clinical, social) and information on exposures and potential confounders<br>(b) Indicate the number of participants with missing data for each variable of interest<br>(c) *Cohort study* - summarise follow-up time (*e.g.*, average and total amount)			N/A– publicly available national death counts
Outcome data	15	*Cohort study* - Report numbers of outcome events or summary measures over time<br>*Case-control study* - Report numbers in each exposure			N/A– publicly available national death counts

For peer review only

		category, or summary measures of exposure<br>*Cross-sectional study* - Report numbers of outcome events or summary measures			
Main results	16	(a) Give unadjusted estimates and, if applicable, confounder-adjusted estimates and their precision (e.g., 95% confidence interval). Make clear which confounders were adjusted for and why they were included<br>(b) Report category boundaries when continuous variables were categorized<br>(c) If relevant, consider translating estimates of relative risk into absolute risk for a meaningful time period			N/A– publicly available national death counts
Other analyses	17	Report other analyses done—e.g., analyses of subgroups and interactions, and sensitivity analyses			N/A– publicly available national death counts
**Discussion**					
Key results	18	Summarise key results with reference to study objectives			
Limitations	19	Discuss limitations of the study, taking into account sources of potential bias or imprecision. Discuss both direction and magnitude of any potential bias		RECORD 19.1: Discuss the implications of using data that were not created or collected to answer the specific research question(s). Include discussion of misclassification bias, unmeasured confounding, missing data, and changing eligibility over time, as they pertain to the study being reported.	N/A– publicly available national death counts
Interpretation	20	Give a cautious overall interpretation of results considering objectives,			Page 9-11

		limitations, multiplicity of analyses, results from similar studies, and other relevant evidence			
Generalisability	21	Discuss the generalisability (external validity) of the study results			Page 9-11
**Other Information**					
Funding	22	Give the source of funding and the role of the funders for the present study and, if applicable, for the original study on which the present article is based			Page 2- Funding acknowledgement
Accessibility of protocol, raw data, and programming code		..		RECORD 22.1: Authors should provide information on how to access any supplemental information such as the study protocol, raw data, or programming code.	Page 6– Link to reproducible data and code available

*Reference: Benchimol EI, Smeeth L, Guttmann A, Harron K, Moher D, Petersen I, Sørensen HT, von Elm E, Langan SM, the RECORD Working Committee. The REporting of studies Conducted using Observational Routinely-collected health Data (RECORD) Statement. *PLoS Medicine* 2015; in press.

*Checklist is protected under Creative Commons Attribution (CC BY) license.

For peer review only

BMJ Open

Comparing trends in Mid-life "Deaths of Despair" in the US, Canada, and UK, 2001-2019: Is the US an Anomaly?

Journal:	*BMJ Open*
Manuscript ID	bmjopen-2022-069905.R1
Article Type:	Original research
Date Submitted by the Author:	12-May-2023
Complete List of Authors:	Dowd, Jennifer; University of Oxford, Leverhulme Centre for Demographic Science; University of Oxford Nuffield College<br>Angus, Colin; University of Sheffield, ScHARR<br>Zajacova, Anna; University of Western Ontario,<br>Tilstra, Andrea; University of Oxford, Leverhulme Centre for Demographic Science; University of Oxford Nuffield College
**Primary Subject Heading**:	Public health
Secondary Subject Heading:	Epidemiology
Keywords:	Substance misuse < PSYCHIATRY, Suicide & self-harm < PSYCHIATRY, PUBLIC HEALTH

SCHOLARONE™
Manuscripts

I, the Submitting Author has the right to grant and does grant on behalf of all authors of the Work (as defined in the below author licence), an exclusive licence and/or a non-exclusive licence for contributions from authors who are: i) UK Crown employees; ii) where BMJ has agreed a CC-BY licence shall apply, and/or iii) in accordance with the terms applicable for US Federal Government officers or employees acting as part of their official duties; on a worldwide, perpetual, irrevocable, royalty-free basis to BMJ Publishing Group Ltd ("BMJ") its licensees and where the relevant Journal is co-owned by BMJ to the co-owners of the Journal, to publish the Work in this journal and any other BMJ products and to exploit all rights, as set out in our licence.

The Submitting Author accepts and understands that any supply made under these terms is made by BMJ to the Submitting Author unless you are acting as an employee on behalf of your employer or a postgraduate student of an affiliated institution which is paying any applicable article publishing charge ("APC") for Open Access articles. Where the Submitting Author wishes to make the Work available on an Open Access basis (and intends to pay the relevant APC), the terms of reuse of such Open Access shall be governed by a Creative Commons licence – details of these licences and which Creative Commons licence will apply to this Work are set out in our licence referred to above.

Other than as permitted in any relevant BMJ Author's Self Archiving Policies, I confirm this Work has not been accepted for publication elsewhere, is not being considered for publication elsewhere and does not duplicate material already published. I confirm all authors consent to publication of this Work and authorise the granting of this licence.

For peer review only

Comparing trends in Mid-life "Deaths of Despair" in the US, Canada, and UK, 2001-2019: Is the US an Anomaly?

Jennifer Beam Dowd*[1,2], Colin Angus[3], Anna Zajacova[4], Andrea M. Tilstra[1,2]

[1] Leverhulme Centre for Demographic Science, Department of Sociology, University of Oxford, Oxford, UK
[2] Nuffield College, University of Oxford, Oxford, UK
[3] School of Health and Related Research, University of Sheffield, Sheffield, UK
[4] Department of Sociology, University of Western Ontario, London, Ontario, Canada

*Corresponding Author
Jennifer Beam Dowd
Leverhulme Centre for Demographic Science
University of Oxford
42-42 Park End Street
Oxford OX1 1JD
United Kingdom
Jennifer.dowd@sociology.ox.ac.uk
+ 44 7986830169

Key Words: Demography, Mortality, Suicide, Substance Abuse

Word Count: 3336

For peer review only

Abstract

Objectives: In recent years, "deaths of despair" due to drugs, alcohol, and suicide have contributed to rising mid-life mortality in the US. We examine whether despair-related deaths and mid-life mortality trends are also changing in peer countries the UK and Canada.

Design: Descriptive analysis of population mortality rates.

Setting: United States, United Kingdom (and constituent nations England & Wales, Northern Ireland, and Scotland), Canada, 2001-2019.

Participants: Full population aged 35-64.

Outcome Measures: We compared all-cause and "despair"-related mortality trends at mid-life across countries using publicly available mortality data, stratified by three age groups (35-44, 45-54 and 55-64) and by sex. We examined trends in all-cause mortality and mortality by causes categorized as 1) suicides 2) alcohol-specific deaths 3) drug-related deaths. We employ several descriptive approaches to visually inspect age, period, and cohort trends in these causes of death.

Results: The US and Scotland both saw large relative increases and high absolute levels of drug-related deaths. The rest of the UK and Canada saw relative increases but much lower absolute levels in comparison. Alcohol-specific deaths showed less consistent trends that did not track other "despair" causes, with older groups in Scotland seeing steep declines over time. Suicide deaths trended slowly upward in most countries.

Conclusions: In the UK, Scotland has suffered increases in drug-related mortality comparable to the US, while Canada and other UK constituent nations did not see dramatic increases. Alcohol-specific and suicide mortality generally follow different patterns to drug-related deaths across countries and over time, questioning the utility of a cohesive "deaths of despair" narrative.

For peer review only

Strengths and Limitations of this Study:

- We directly compare trends in "deaths of despair" in mid-life across the US, UK, and Canada.
- We leverage demographic approaches to understand period vs. cohort trends in these causes of death.
- Coding of deaths caused by alcohol, drug overdose, and suicide may vary across country and time, complicating comparisons.
- Disentangling age, period, and cohort effects in mortality trends is challenging.
- The study is descriptive and cannot speak to the causes of trends in drug overdose, suicide, and alcohol-specific deaths.

For peer review only

Introduction

The United States (US) has persistently lower life expectancy than its OECD peers, including 1.7-3.2 years below the United Kingdom (UK) and Canada in 2019 [1], two countries with a shared cultural, socio-political, and linguistic heritage. US life expectancy fell for three consecutive years starting in 2014, recovering slightly by 2019 prior to further declines in 2020 and 2021 during the COVID-19 pandemic [2, 3]. Explanations for stagnating life expectancy in the US and divergence from peer countries have highlighted increases in "deaths of despair" (i.e., deaths due to drug and alcohol poisoning, and suicide)[4, 5]. Life expectancy improvements have been stalling in England and Wales [6, 7] and Scotland [8] since the 2010's, raising the question of whether these US mortality dynamics reflect a broader phenomenon.

The US, the UK, and Canada have all documented rapid increases in drug-related mortality in recent years, with the US on the leading edge with an initial increase in deaths due to prescription opioids, followed by heroin and now fentanyl [9, 10]. Increases in opioid use in Canada may be related to exposure to US pharmaceutical promotional material through US media and direct marketing [11]. Within the UK, Scotland stands out with a dramatic rise in drug deaths (rising 450% since 1996) driven initially by prescription benzodiazepines and more recently by so-called 'street' benzodiazepines [12]. England and Wales have had a less extreme rise in drug-related deaths since 2012, driven by increasing numbers of opioid and cocaine-related deaths, and less noticeable increases due to benzodiazepines [13].

Besides changes in the supply and marketing of prescription and illicit drugs, increases in drug mortality may reflect demand side factors related to the loss of economic opportunity, particularly among socioeconomically disadvantaged groups [14, 15]. In Scotland, cohort trends in drug-related deaths and suicide were evident particularly in men from socioeconomically deprived areas coming of age in the 1980s [16, 17]. In the US and

For peer review only - http://bmjopen.bmj.com/site/about/guidelines.xhtml

the UK, lack of economic opportunity in middle-aged cohorts may contribute to poorer mental health and subsequent unhealthy coping behaviours [18, 19]. In Canada, socioeconomic inequalities in health measured by educational attainment and household income have increased over time, but less is known about whether this has translated into despair-related causes of death [20].

This paper compares recent trends in mid-life “deaths of despair” and its component causes in the US to those in the UK and Canada from 2000-2019. The UK was chosen as a comparison country also witnessing stalls in life expectancy. Thus far, whether UK patterns of mid-life mortality and despair-related are following the US is not known. Canada shares close economic and geographic ties to the US and has seen spill over effects of changing drug supply and the opioid epidemic [21].

Data

Publicly available all-cause and cause-specific mortality by age and sex were analysed for all countries. Because data are collected by separate statistical offices for the nations of the UK, and because of historically divergent mortality trends for Scotland, we estimate trends separately for UK nations, except for England and Wales whose data is released together. For England and Wales, we analyzed the 21st Century Mortality dataset from 2001-2019 [22], a publicly available resource that includes all registered deaths by cause as well as mid-year population counts by five-year age groups. Comparable data for Scotland is available from National Records of Scotland’s Vital Events Reference Tables [23]. and for Northern Ireland from The Registrar General’s Annual Reports [24]. For Canada, death counts by cause of death were accessed from publicly available Statistics Canada Table 13-10-0392-01 [25]. These records include all registered deaths in Canada each year from 2000-2019 by sex and 5-year age-group. US mortality data by year and single year of age come from CDC Wonder

For peer review only

[26]. Population counts by age and sex for all countries were obtained from the Human Mortality Database. Results are fully reproducible, and all metadata and code used in analysis can be accessed here.

Methods

We focus on mid-life mortality where increases have been documented in the U.S, specifically age groups 35-44, 45-54 and 55-64. We examine trends mortality by causes categorized as 1) suicides, 2) alcohol-specific deaths 3) drug-related deaths, and a combined 4) "deaths of despair" category summing all three causes, as well as all-cause mortality. (ICD-10 codes available in supplemental Table S1). In order to mitigate the impact of random variation in the cause-and age-specific mortality rates we used a 1-dimensional P-spline approach for each country, cause, sex and calendar year to smooth the observed death rates over age [27]. The resulting single year of age-specific mortality rates were then age-standardised within each of the three broad age groups using the European Standard Population [28]. We visually inspected cohort vs. period trends using plots of the 3-year rolling averages within age-groups over time, supplemented with Lexis surfaces.

Patient and Public Involvement

None

Results

Full results for all age group, sex, country and cause groupings for all calendar years are shown in Appendix Tables S2a – S6b, with summary data for 2001, 2010 and 2019 presented in Tables 1a-b and 2a-b. All-cause and cause-specific mortality trends for the combined age range of 35-64 years of age are shown in Supplemental figures S4 and S5a-c.

Table 1a: Male mortality rates (per 100,000 population) by cause and age group for England & Wales, Northern Ireland and Scotland

Age group	England & Wales			Northern Ireland			Scotland		
	2001	2010	2019	2001	2010	2019	2001	2010	2019
All-Cause									
35-44	190.7	168.9	162.8	192.7	207.7	190.4	274.9	244.0	284.2
45-54	461.4	393.9	371.0	465.7	443.7	378.1	649.0	520.1	529.8
55-64	1280.1	991.9	888.6	1324.3	1092.9	876.9	1708.3	1257.4	1076.2
Combined 'Deaths of Despair'									
35-44	44.8	48.0	58.0	47.6	67.9	81.9	82.2	99.5	157.5
45-54	50.5	58.0	71.1	54.9	82.6	85.0	114.9	109.1	139.2
55-64	46.6	56.0	66.1	50.0	79.6	74.5	127.9	108.3	98.0
Alcohol									
35-44	19.8	20.3	16.3	26.7	26.3	29.0	40.9	35.0	24.7
45-54	32.7	37.4	35.3	41.2	50.8	55.5	86.1	70.8	48.5
55-64	33.1	41.4	43.3	38.1	56.8	58.8	107.3	87.0	60.4
Drugs									
35-44	9.8	12.8	22.8	1.6	10.6	23.6	15.1	36.0	102.4
45-54	4.6	6.1	16.6	1.8	6.3	6.2	5.5	16.6	63.6
55-64	2.3	2.5	6.5	2.0	4.1	0.8	2.3	6.6	15.6
Suicide									
35-44	15.2	14.9	18.9	19.4	31.0	29.3	26.2	28.5	30.4
45-54	13.2	14.5	19.1	11.9	25.5	23.4	23.2	21.7	27.1
55-64	11.1	12.1	16.3	9.9	18.7	15.0	18.3	14.7	22.0

For peer review only

Table 1b: Male mortality rates (per 100,000 population) by cause and age group for Canada and the USA

Age group	Canada 2001	Canada 2010	Canada 2019	USA 2001	USA 2010	USA 2019
All-Cause						
35-44	183.6	164.1	161.7	259.2	212.2	257.3
45-54	424.3	361.3	334.6	551.3	508.9	484.9
55-64	1155.9	916.9	826.3	1226.1	1082.7	1106.7
Combined 'Deaths of Despair'						
35-44	44.4	46.3	50.1	58.2	61.0	101.6
45-54	53.7	58.2	62.6	77.2	94.1	115.5
55-64	67.9	68.3	75.5	68.7	93.0	134.8
Alcohol						
35-44	8.2	8.5	8.4	14.9	12.4	16.9
45-54	22.7	22.1	21.0	36.9	36.8	37.1
55-64	42.5	38.5	40.2	42.9	50.3	63.2
Drugs						
35-44	9.7	13.4	21.1	20.1	23.6	56.6
45-54	6.1	11.7	19.6	16.7	26.7	49.2
55-64	3.2	7.1	13.1	4.6	15.1	41.1
Suicide						
35-44	26.6	24.4	20.6	23.3	25.1	28.1
45-54	24.9	24.4	22.0	23.7	30.6	29.3
55-64	22.3	22.7	22.1	21.2	27.6	30.5

For peer review only

Table 2a: Female mortality rates (per 100,000 population) by cause and age group for England & Wales, Northern Ireland and Scotland

Age group	England & Wales			Northern Ireland			Scotland		
	2001	2010	2019	2001	2010	2019	2001	2010	2019
All-Cause									
35-44	117.5	104.8	98.7	119.4	122.3	115.0	149.2	133.4	153.4
45-54	302.5	260.4	243.0	310.2	292.7	274.0	387.9	328.6	341.1
55-64	802.5	641.2	587.8	809.9	704.9	639.7	1014.3	835.8	759.9
Combined 'Deaths of Despair'									
35-44	16.8	18.3	23.2	20.1	26.3	32.3	35.6	37.4	61.7
45-54	23.0	24.6	30.5	29.4	39.6	43.3	48.4	46.4	64.7
55-64	23.7	25.4	29.4	24.6	36.3	42.5	49.9	44.9	53.3
Alcohol									
35-44	9.6	10.2	9.7	15.0	13.9	17.1	19.9	17.9	12.8
45-54	16.4	17.3	18.2	22.9	25.9	29.9	36.7	32.2	28.4
55-64	17.7	19.6	20.5	20.8	26.1	32.7	41.3	35.6	37.5
Drugs									
35-44	3.5	4.3	8.4	1.2	4.4	8.2	4.8	10.5	40.2
45-54	2.5	3.3	7.1	2.4	4.2	6.4	2.8	7.5	26.1
55-64	1.9	2.1	4.2	0.2	3.1	4.1	2.0	4.7	9.0
Suicide									
35-44	3.8	3.8	5.2	3.9	7.9	7.0	10.9	8.9	8.7
45-54	4.2	4.0	5.3	4.1	9.5	7.0	9.0	6.7	10.2
55-64	4.1	3.7	4.7	3.6	7.1	5.8	6.6	4.6	6.8

For peer review only

Table 2b: Female mortality rates (per 100,000 population) by cause and age group for Canada and the USA

	Canada			USA		
Age group	2001	2010	2019	2001	2010	2019
All-Cause						
35-44	112.1	100.2	94.6	147.6	128.1	142.8
45-54	268.6	235.9	212.6	319.8	311.7	295.1
55-64	685.9	586.7	529.9	773.4	647.8	667.8
Combined 'Deaths of Despair'						
35-44	15.9	19.1	19.0	22.0	27.7	40.5
45-54	20.0	24.3	24.1	26.3	42.8	51.2
55-64	24.1	26.7	28.9	24.1	35.1	53.8
Alcohol						
35-44	4.0	4.2	4.3	6.4	5.5	8.6
45-54	8.6	8.5	9.5	12.1	14.6	17.7
55-64	14.7	13.2	16.4	15.8	17.3	26.7
Drugs						
35-44	4.1	7.2	9.0	9.2	14.7	23.6
45-54	3.4	7.3	8.2	7.0	19.0	23.2
55-64	2.5	5.7	5.8	2.6	9.9	18.2
Suicide						
35-44	7.8	7.6	5.7	6.5	7.4	8.2
45-54	8.0	8.4	6.5	7.2	9.2	10.3
55-64	6.9	7.8	6.7	5.7	7.9	8.9

For peer review only

Figure 1 shows all-cause mortality rates from 2001-2019 by age and sex group. Observed mortality patterns differ across nations and by sex. In Canada and England & Wales, mortality rates are low and decline consistently for most age-sex combinations, except for 35–44-year-olds for which mortality trends are flat or increasing. Upticks in mortality were more noticeable in Scotland and the US in 35–44-year-olds, with steeper increases for males compared to females. In the US, all-cause mortality among males 35-44 reached a low point of 211.8/100,000 in 2012 before rising steadily to 257.3/100,000 in 2019 (Supplemental Table S6a**)**. Males aged 35-44 in Scotland followed a similar trend, with slightly higher absolute levels of mortality compared to US males. This youngest age group also showed signs of stalling improvements or slight increases in mortality in Canada, England & Wales, and Northern Ireland. Noticeable increases in overall mortality were also seen in Scotland for males and females ages 45-64, but not in the 55–64-year-old age group. The US was notable for its stagnation or increases in mortality across almost all age-sex groups.

Figure 2 shows trends in despair-related deaths over the same period. The US and Scotland again stand out for substantially higher and largely increasing rates across the period. Males aged 35-44 show some of the worst trends among analyzed countries, with rates in the US and Scotland increasing almost 2-fold over the period. While the absolute increases for males in England and Wales and Northern Ireland are much smaller, they reflect large relative increases (for example a 41% increase in despair-related deaths for males ages 45-54 over this period in England and Wales). Canada is notable for its low levels and relatively small increases in deaths of despair, especially for women for whom rates hardly increase. While females in most nations saw only small increases in deaths of despair, the US stood out with a trend of high and rising mortality for both males and females in all age groups.

For peer review only

Figures 2a-c break down these “despair” deaths into individual causes, where we see the salience of increasing drug-related mortality in the US and Scotland. The magnitude of the increases for males ages 35-44 in both countries was staggering (an almost 6-fold increase in Scotland from 15.1/100,000 to 102.3/100,000). In the US, these increases are seen across all age groups, while in Scotland the increases are significantly less pronounced for 55–64-year-olds. Although at lower absolute levels, drug-related mortality also increased in Canada, England and Wales and Northern Ireland over the period, especially among younger males (for example an increase of 118% from 9.1/100,000 to 21.1/100,000 for Canadian males aged 35-44).

Suicide mortality saw more year-to-year variability, with evidence of small increases among males in England & Wales, Scotland, and the US. The magnitude of these changes is very small compared to changes in drug-related deaths. Suicide rates were higher for males in all nations. Canada consistently had the lowest levels of suicide mortality across all age-sex groups. The US generally had the highest levels of suicide deaths, except for males ages 35-44 where Scotland and Northern Ireland had higher levels. Alcohol-specific mortality across most nations was relatively stable, but with sharp declines over time in Scotland from previously very high levels, especially at the older ages. In contrast, increases in alcohol-specific mortality in the US were seen in ages 55-64, especially for men. Taken together, these results suggest that drug-related mortality is the primary cause of increases in overall “despair”-related mortality over this period, with small but perceptible increases in England & Wales, Canada, and Northern Ireland, and very pronounced increases in the US and Scotland.

Figures S1a-c show an alternative view of the same data to examine period vs. cohort trends. Here we look for “non-parallelism” in the age-specific mortality curves over time within each country [29]. If the age-specific curves are parallel to each other, this likely reflects the

For peer review only

salience of period influences. Non-parallel age-specific trends suggest more importance for cohort effects (For a detailed discussion and simulations of these dynamics, see [30], pages 204-217). For overall deaths of despair, age-specific trends over time are mostly parallel for Canada, England & Wales, and Northern Ireland, suggesting period-based dynamics affecting all cohorts. The US and Scotland show some deviations from parallelism, with the youngest age group showing sharper increases than the older groups in Scotland, and the 55–64-year-olds in the US increasing more steeply (and earlier) than younger groups. For drug-related deaths, parallel trends are largely suggestive of period-based effects for Canada, England & Wales, and the US. Non-parallelism in Northern Ireland & Scotland suggests younger born cohorts have been hit harder by recent increases in drug deaths than the oldest cohort. For suicide, most trends are parallel, suggesting period fluctuations, except for the US where the oldest age group has increased more steeply. Alcohol-specific mortality mostly follows period-based parallel trends, except for the US where again the oldest age group has increased over time. Cohort-driven declines in alcohol-related mortality were seen in Scotland, with the older groups declining steeply from high levels compared to lower and flatter trends in the youngest age group. Supplemental Figures S2a-b show alternative visualizations for cohort patterns in mortality using Lexis surfaces. Overall, these visualizations highlight some limited cohort trends but also very strong period effects of increased drug deaths that affected most age groups and nations. In the US, increases in drug-related deaths affected all age groups over time, but sharper increases in alcohol-specific deaths and suicide were only seen in the oldest age group (earliest born cohort). This oldest group largely reflects the Baby Boom cohort (born 1946-1964) passing this 55-64 age group over the observed period.

For peer review only

Supplemental Figures S3a-c show trends in the proportion all deaths due to the individual despair related causes over time, with drug-related mortality standing out as an increasing proportion of all-cause mortality. From Table 1a, we can see that the all-cause mortality rate increased by 45/100,000 for 35–44-year-old US males from 2010 to 2019. Of this increase, 73% could be accounted for by an increase in drug-related mortality, and 90% was accounted for by increases in all three despair-related causes. For 55–64-year-old males, all-cause mortality increased by 23.9/100,000 over the same period, which was more than fully accounted for by an increase in drug-related mortality of 26/100,000 from 2010 to 2019. Thus, in many age-sex groups all-cause mortality would have declined or plateaued rather than increased in the US if drug mortality had stayed constant.

Discussion

We describe large relative increases and high absolute levels of drug-related deaths in both the US and Scotland from 2001-2019, with lower levels and smaller increases in Canada and the rest of the UK. Alcohol-specific deaths showed less consistent trends that did not track other “despair” causes, with older groups in Scotland seeing steep declines over time. Suicide deaths trended slowly upward in most countries. We generally found more evidence for period rather than cohort effects for these trends, apart from baby boomers in the US, consistent with previous evidence of their cohort mortality disadvantage [31].

Increases in mid-life mortality in the last two decades in the US set off a robust debate about the underlying causes of this concerning trend, with a strong focus on “deaths of despair.” One narrative emphasizes the role of cumulative disadvantage triggered by worsening labour market opportunities for white Americans with low levels of education, leading to psychological

For peer review only

distress and unhealthy behavioural coping mechanisms [5, 32, 33]. A second hypothesis emphasizes the strong period trend in drug-related mortality due to changes in the availability of prescription and illicit opioids, including heroin, and synthetic opioids like fentanyl)[34, 35]. While the first hypothesis implies that "despair" deaths share a fundamental cause and typically move together, the latter argues that the trends in suicide and alcohol-specific mortality are distinct and do not fit well within the overall deaths of despair narrative [36, 37].

Our study extends this work by comparing mid-life mortality trends in the US to Canada and the UK, two countries with shared sociocultural histories with the US but different economic, political and health care contexts. The UK has also experienced recent slowdowns in mortality improvements, raising the question of whether the US is the leading edge of more general mortality trends. Overall, we find evidence that the levelling off and increases in mid-life mortality seen in the US may be emerging more broadly, but the magnitude of these changes in other countries is muted. Scotland is the exception, with upward trends in drug-related deaths surpassing the high levels in the US and contributing to increases in overall mortality trends for males ages 35-54. While drug-related deaths have increased on a relative scale in the other UK nations and Canada, deaths from these causes are at much lower absolute levels than the US and Scotland. There is little evidence of significant trends in suicide or alcohol-specific deaths contributing to changes in all-cause mortality.

Our results suggest that increases in drug-related mortality are not necessarily unique to the US, with smaller increases also seen in Canada, England & Wales, and Northern Ireland, particularly among younger males. Scotland stands out as trending even higher than the US in drug-related mortality at younger ages, while the US saw parallel increases in drug-mortality across all age-ranges, suggesting a strong period effect. We found some evidence of cohort

For peer review only

effects in alcohol-specific and suicide mortality in the US, with the oldest cohort trending up relative to younger cohorts. Suicide mortality rates were generally stable across countries for women, with slight increases for men and women in the US. Overall, with the exception of the oldest born (i.e. baby boom) male cohort in the US, we find that individual "deaths of despair" do not follow similar age and cohort patterns and thus may not reflect the same fundamental causes.

The fact that drug-related deaths have increased in other countries but not to the degree of the US is consistent with differences in prescribing practices and health care contexts. In the UK, there is no direct-to-consumer marketing by pharmaceutical companies, and the primary care system likely mitigates patients receiving prescriptions from multiple sources [38]. Nonetheless, prescribing for non-cancer pain did increase dramatically since the late 1990s when consensus statements supporting the use of opioids for the treatment of chronic pain [39, 40]. Despite this increase in overall opioid prescribing, a review of the UK Clinical Practice Research Datalink (CRPD) did not find an increase in the rate of diagnosed opioid use disorders in the UK between 2008 and 2012 [41]. While deaths from fentanyl in the UK have grown, they are still small relative to deaths from heroin or morphine, suggesting the context for drug prescribing and misuse may be different in the UK [42]. The dramatic rise in drug deaths in Scotland, with rates far outstripping those elsewhere in Europe[1] has arisen through a somewhat different mechanism to the US' initial prescription opioid-driven crises. A combination of economic, social and political factors experienced by those living in deprived areas in the 1980s led to a rise in drug-related death rates in the 1990s [16, 17], driven by opioids such as heroin, often taken in combination with prescription benzodiazepines such as diazepam [43]. Although steps were

[1] https://www.nrscotland.gov.uk/files//statistics/drug-related-deaths/20/drug-related-deaths-20-pub.pdf

For peer review only

taken to reduce the prescribing of benzodiazepines in the late 2000s, these were swiftly replaced by a range of 'synethic benzodiazepines' or 'street benzos' such as etizolam, which became widely and cheaply available through illicit markets [12]. The explosion in the use of street benzos has played a huge role in the rapid rise in Scottish drug deaths since 2012 when street benzos were implicated in 3% of all drug deaths compared to 66% in 2020, with concurrent use with opioids in particular the key factor – less than 1% of drug deaths in 2020 involved street benzos alone[2]. In England, austerity measures after the 2008 financial crisis have been suggested as one cause of stalling life expectancy [44, 45]. While many have focused on cuts in health and social care affecting older age mortality[46], some evidence has linked austerity to an increase in drug deaths affecting younger and middle ages [47]. While concern over the opioid epidemic and fentanyl in Canada is rising [48], the absence of overall increases in mortality thus far has minimized discussion of a similar epidemic of "deaths of despair."

Strengths and Limitations

Our study has several strengths and limitations. We use a demographic perspective to compare period and cohort trends in "despair" related deaths in the US, UK, and Canada. Disentangling age, period, and cohort effects in mortality is a persistent challenge, and we take a transparent visualization approach to ease interpretation. When examining external causes of death such as those due to alcohol, drug overdose, and suicide, there are differences in coding practices across country and time that could impact results. The overlapping nature of these causes also means there will be limitations to any set of definitions. We largely follow the original "despair" categorizations of Case and Deaton [5], and have previously demonstrated that the use of alternative categorizations are unlikely to substantively change our conclusions [49].

---

[2] https://www.nrscotland.gov.uk/statistics-and-data/statistics/statistics-by-theme/vital-events/deaths/drug-related-deaths-in-scotland/2020

For peer review only

Additionally, our study is descriptive and cannot speak to the causes of trends in drug overdose, suicide, and alcohol-specific deaths or related policy solutions. Finally, we examine trends only through 2019 and do not consider the specific dynamics of trends in despair-related mortality during the COVID-19 pandemic. Recent work showed drug deaths rising considerably in the US during the pandemic across all ages, which was not seen in England or Scotland [49]. Concerns about rising suicide rates during the pandemic seem unfounded, but alcohol-related deaths increased in both the US and UK [49]. The impact of the pandemic as a period and cohort shock to risk behaviours and causes of deaths will be an important area of future research.

Conclusions

"Deaths of despair" due to alcohol, drug overdoses, and suicide have been implicated in rising mid-life mortality in the US, but how these trends compare to peer countries is less well-known. We find that the US is unique in its large relative and absolute increases in drug-related mortality for all mid-life age-sex-groups, but that Scotland has seen a dramatic upward trend in drug deaths that now exceed the US for younger age groups. The huge increase in drug mortality across all ages in the US from 2001-2019 is most consistent with period explanations, while alcohol and suicide upward trends in the US are more focused in the oldest cohort. Overall, the trends in individual causes of despair deaths are not consistent across countries or age-groups, questioning the value of a cohesive "deaths of despair" narrative. This is consistent with other work finding different spatiotemporal patterns of these causes within the US [50]. Future research should consider the determinants of these specific causes across place and time without assuming they represent an underlying phenomenon with the same underlying drivers [51].

For peer review only

Ethics Approval Statement

The study does not involve human subjects, as it uses publicly available death counts from national vital statistics and no individual-level data.

Contributorship statement

The authors confirm contribution to the paper as follows: study conception and design: JBD; data collection CA, JBD, AZ; analysis and interpretation of results: CA, JBD; draft manuscript preparation: JBD. All authors critically reviewed the results and approved the final version of the manuscript.

Competing interests

The authors have no competing interests to declare.

Data sharing statement

Data are available in a public, open access repository. Results are fully reproducible, and all data and code can be accessed at:

https://github.com/VictimOfMaths/DeathsOfDespair/tree/master/Paper

Funding Acknowledgement

The authors acknowledge funding support from the European Research Council (ERC-2021-CoG-101002587) and The Leverhulme Trust (Large Centre Grant). The funders played no direct role on this research.

For peer review only

References

1. Aburto JM, Schöley J, Kashnitsky I, Zhang L, Rahal C, Missov TI, et al. Quantifying impacts of the COVID-19 pandemic through life-expectancy losses: a population-level study of 29 countries. International Journal of Epidemiology. 2021. doi: 10.1093/ije/dyab207.

2. Murphy SL, Xu J, Kochanek KD, Arias E. Mortality in the united states, 2017. 2018.

3. Schöley J, Aburto JM, Kashnitsky I, Kniffka MS, Zhang L, Jaadla H, et al. Bounce backs amid continued losses: Life expectancy changes since COVID-19. medRxiv. 2022:2022.02.23.22271380. doi: 10.1101/2022.02.23.22271380.

4. Tilstra AM, Simon DH, Masters RK. Trends in "Deaths of Despair" among Working Aged White and Black Americans, 1990-2017. American journal of epidemiology. 2021.

5. Case A, Deaton A. Mortality and morbidity in the 21st century. Brookings Papers on Economic Activity. 2017.

6. Hiam L, Dorling D, Harrison D, McKee M. Why has mortality in England and Wales been increasing? An iterative demographic analysis. Journal of the Royal Society of Medicine. 2017;110(4):153-62.

7. Murphy ML, Marc; Torrisi, Orsola. Stalling of mortality in the United Kingdom and Europe: an analytical review of the evidence. LSE Department of Social Policy Working Paper Series2019.

8. Minton J, Fletcher E, Ramsay J, Little K, McCartney G. How bad are life expectancy trends across the UK, and what would it take to get back to previous trends? J Epidemiol Community Health. 2020;74(9):741-6.

9. Ciccarone D. The triple wave epidemic: Supply and demand drivers of the US opioid overdose crisis. International Journal of Drug Policy. 2019;71:183-8. doi: https://doi.org/10.1016/j.drugpo.2019.01.010.

10. John E, Butt A, McQuade G. Deaths related to drug poisoning in England and Wales: 2018 registrations. Office for National Statistics, Aug; 2019.

11. Lexchin J, Kohler JC. The danger of imperfect regulation: OxyContin use in the United States and Canada. International Journal of Risk & Safety in Medicine. 2011;23(4):233-40.

12. McAuley A, Matheson C, Robertson JR. From the clinic to the street: the changing role of benzodiazepines in the Scottish overdose epidemic. International Journal of Drug Policy. 2022;100:103512. doi: https://doi.org/10.1016/j.drugpo.2021.103512.

13. Breen P, Manders B. Deaths related to drug poisoning in England and Wales: 2020 registrations. Off Natl Stat. 2021.

For peer review only

14. O'Brien R, Bair EF, Venkataramani AS. Death by Robots? Automation and Working-Age Mortality in the United States. Demography. 2022;59(2):607-28. doi: 10.1215/00703370-9774819.

15. Ruhm CJ. Drivers of the fatal drug epidemic. Journal of health economics. 2019;64:25-42.

16. Parkinson J, Minton J, Lewsey J, Bouttell J, McCartney G. Drug-related deaths in Scotland 1979–2013: evidence of a vulnerable cohort of young men living in deprived areas. BMC Public Health. 2018;18(1):357. doi: 10.1186/s12889-018-5267-2.

17. Parkinson J, Minton J, Lewsey J, Bouttell J, McCartney G. Recent cohort effects in suicide in Scotland: a legacy of the 1980s? Journal of Epidemiology and Community Health. 2017;71(2):194. doi: 10.1136/jech-2016-207296.

18. Marmot M. Social causes of the slowdown in health improvement. BMJ Publishing Group Ltd; 2018.

19. Walsh D, McCartney G, Minton J, Parkinson J, Shipton D, Whyte B. Deaths from 'diseases of despair' in Britain: comparing suicide, alcohol-related and drug-related mortality for birth cohorts in Scotland, England and Wales, and selected cities. Journal of Epidemiology and Community Health. 2021;75(12):1195. doi: 10.1136/jech-2020-216220.

20. Hajizadeh M, Mitnitski A, Rockwood K. Socioeconomic gradient in health in Canada: Is the gap widening or narrowing? Health Policy. 2016;120(9):1040-50.

21. Jannetto PJ. The north American opioid epidemic. Therapeutic Drug Monitoring. 2021;43(1):1-5.

22. [dataset] Deaths registered in England and Wales – 21st century mortality. Office for National Statistics; 2022. Doi: https://www.ons.gov.uk/peoplepopulationandcommunity/birthsdeathsandmarriages/deaths/datasets/the21stcenturymortalityfilesdeathsdataset

23. [dataset] Archive- Historical data back to 2001. National Records of Scotland. doi: https://www.nrscotland.gov.uk/statistics-and-data/statistics/statistics-by-theme/vital-events/general-publications/vital-events-reference-tables/archive

24. [dataset] Registrar General Annual Reports 2011-2019. Northern Ireland Statistics and Research Agency; 2021. doi: https://www.nisra.gov.uk/publications/registrar-general-annual-reports-2011-2019

25. [dataset] Statistics Canada. Deaths and Age-Specific Mortality rates. Table 13-10-0392-01 January 1, 2022. doi: https://www150.statcan.gc.ca/t1/tbl1/en/tv.action?pid=1310039201

26. [dataset] CDC Wonder. US Centers for Disease Control and Prevention. doi: https://wonder.cdc.gov/

For peer review only

27. Camarda CG. MortalitySmooth: An R package for smoothing Poisson counts with P-splines. Journal of Statistical Software. 2012;50:1-24.

28. Pace M, Lanzieri G, Glickman M, Zupanič T. Revision of the European Standard Population: report of Eurostat's task force: Publications Office of the European Union; 2013.

29. Kupper LL, Janis JM, Karmous A, Greenberg BG. Statistical age-period-cohort analysis: a review and critique. Journal of chronic diseases. 1985;38(10):811-30.

30. National Academies of Sciences E, Medicine. High and Rising Mortality Rates Among Working-Age Adults. 2021.

31. Acosta E, Gagnon A, Ouellette N, Bourbeau RR, Nepomuceno MR, van Raalte AA. The boomer penalty: excess mortality among baby boomers in Canada and the United States. Max Planck Institute for Demographic Research, Rostock, Germany, 2020.

32. Case A, Deaton A. Deaths of Despair and the Future of Capitalism. Deaths of Despair and the Future of Capitalism: Princeton University Press; 2020.

33. Zajacova A, Grol-Prokopczyk H, Zimmer Z. Pain trends among american adults, 2002–2018: Patterns, disparities, and correlates. Demography. 2021;58(2):711-38.

34. Masters RK, Tilstra AM, Simon DH. Mortality from suicide, chronic liver disease, and drug poisonings among middle-aged US white men and women, 1980–2013. Biodemography and social biology. 2017;63(1):31-7.

35. Alpert A, Evans WN, Lieber EMJ, Powell D. Origins of the Opioid Crisis and its Enduring Impacts*. The Quarterly Journal of Economics. 2022;137(2):1139-79. doi: 10.1093/qje/qjab043.

36. Masters RK, Tilstra AM, Simon DH. Explaining recent mortality trends among younger and middle-aged White Americans. International journal of epidemiology. 2017;47(1):81-8.

37. Ruhm CJ. Deaths of despair or drug problems? National Bureau of Economic Research, 2018.

38. Stannard C. Opioid prescribing in the UK: can we avert a public health disaster? British journal of pain. 2012;6(1):7.

39. Max MB, Donovan M, Miaskowski CA, Ward SE, Gordon D, Bookbinder M, et al. Quality improvement guidelines for the treatment of acute pain and cancer pain. Jama. 1995;274(23):1874-80.

40. Zin CS, Chen LC, Knaggs RD. Changes in trends and pattern of strong opioid prescribing in primary care. European journal of pain. 2014;18(9):1343-51.

For peer review only

41. Cooper AJ, Willis J, Fuller J, Benecke H, Leighton-Scott J, Andersohn F, et al. Prevalence and incidence trends for diagnosed prescription opioid use disorders in the United Kingdom. Pain and therapy. 2017;6(1):73-84.

42. Torjesen I. Fentanyl misuse in the UK: will we see a surge in deaths? bmj. 2018;361:k1564.

43. Jones JD, Mogali S, Comer SD. Polydrug abuse: a review of opioid and benzodiazepine combination use. Drug and alcohol dependence. 2012;125(1-2):8-18.

44. McCartney G, McMaster R, Popham F, Dundas R, Walsh D. Is austerity a cause of slower improvements in mortality in high-income countries? A panel analysis. Social Science & Medicine. 2022;313:115397. doi: https://doi.org/10.1016/j.socscimed.2022.115397.

45. Dorling D. Austerity bites: falling life expectancy in the UK. BMJ. 2019.

46. Hiam L, Harrison D, McKee M, Dorling D. Why is life expectancy in England and Wales 'stalling'? J Epidemiol Community Health. 2018;72(5):404-8.

47. Friebel R, Yoo KJ, Maynou L. Opioid abuse and austerity: evidence on health service use and mortality in England. Social Science & Medicine. 2022;298:114511.

48. Fischer B, Vojtila L, Rehm J. The 'fentanyl epidemic'in Canada–some cautionary observations focusing on opioid-related mortality. Preventive medicine. 2018;107:109-13.

49. Angus C, Buckley C, Tilstra AM, Dowd JB. Increases in 'deaths of despair' during the COVID-19 pandemic in the United States and the United Kingdom. Public Health. 2023;218:92-6. doi: https://doi.org/10.1016/j.puhe.2023.02.019.

50. Simon DH, Masters RK. Do deaths of despair move together? County-level mortality changes by sex and urbanization, 1990–2017. American Journal of Epidemiology. 2021;190(6):1169-71.

51. Tilstra AM. Stop Analyzing Suicides, Drug-and Alcohol-Related Mortality Together: Response to "Are We Undercounting the True Burden of Mortality". American journal of epidemiology. 2023.

For peer review only

Figure Legends

Figure 1: All-cause mortality by age and sex in Canada, the UK and the USA, 2001-2019

Figure 2: Combined 'deaths of despair' mortality (drug, suicide, and alcohol) by age and sex in Canada, the UK and the USA, 2001-2019

Figure 2a – Drug-related mortality by age and sex in Canada, the UK and the USA, 2001-2019

Figure 2b - Suicide mortality by age and sex in Canada, the UK and the USA, 2001-2019

Figure 2c – Alcohol-specific mortality by age and sex in Canada, the UK and the USA, 2001-2019

For peer review only

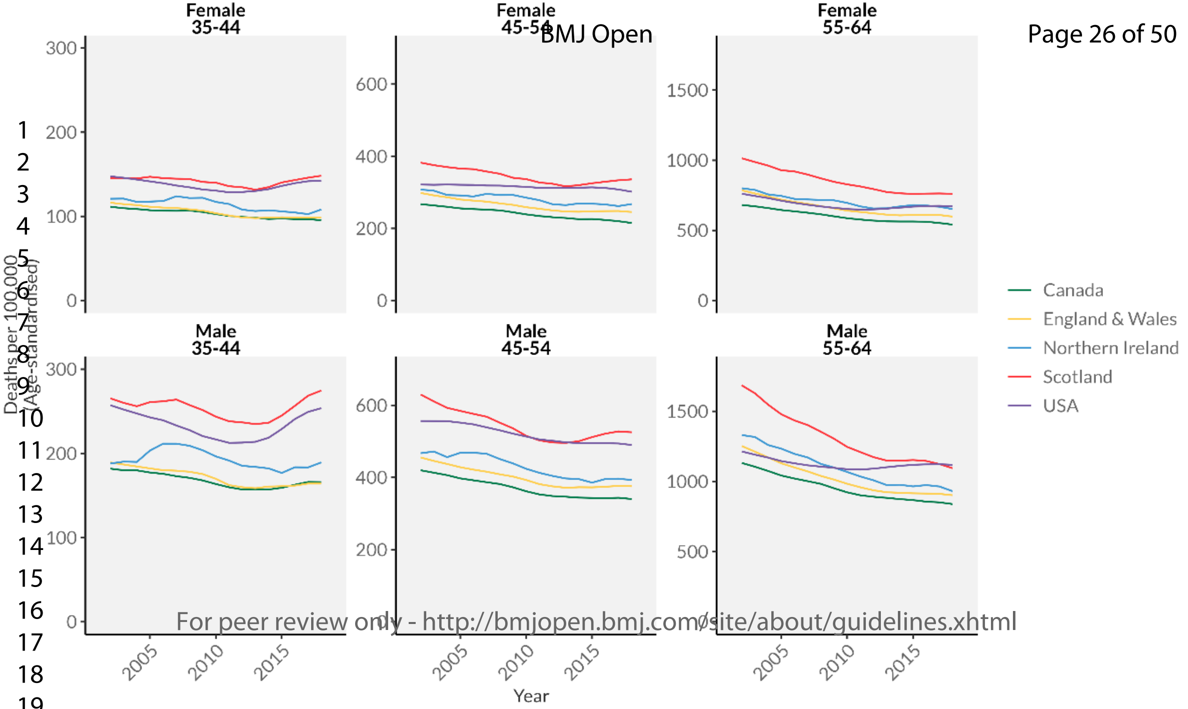
Female
35-44
Female
45-54
Female
55-64
Male
35-44
Male
45-54
Male
55-64
Deaths per 100,000
(Age-standardised)
Year
Canada
England & Wales
Northern Ireland
Scotland
USA

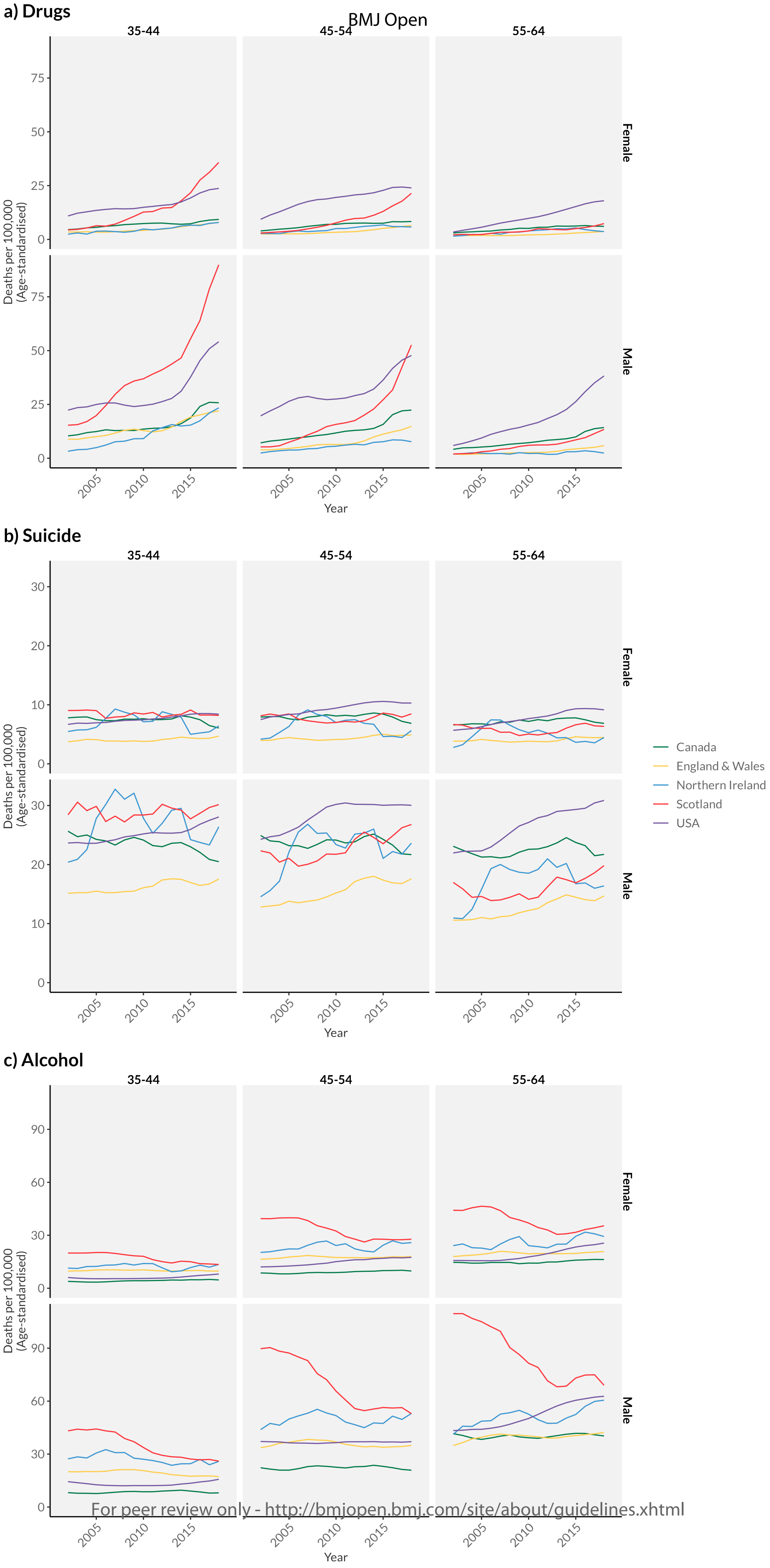
a) Drugs
b) Suicide
c) Alcohol
35-44
45-54
55-64
Female
Male
Deaths per 100,000
(Age-standardised)
Year
0
25
50
75
10
20
30
60
90
2005
2010
2015
Canada
England & Wales
Northern Ireland
Scotland
USA

Supplemental Figures and Tables

Figure S1 - Mortality from combined 'deaths of despair' by age and sex in Canada, the UK and the US, 2001-2019

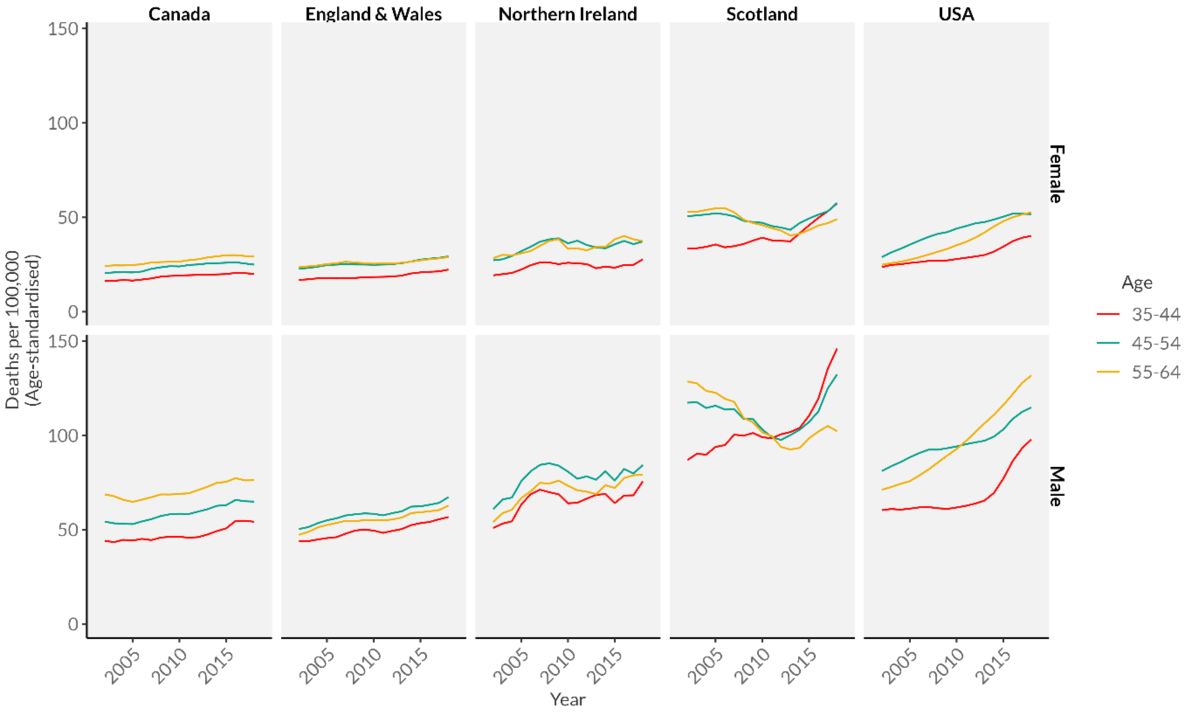

Figure S1a - Drug-related mortality by age and sex in Canada, the UK and the US, 2001-2019

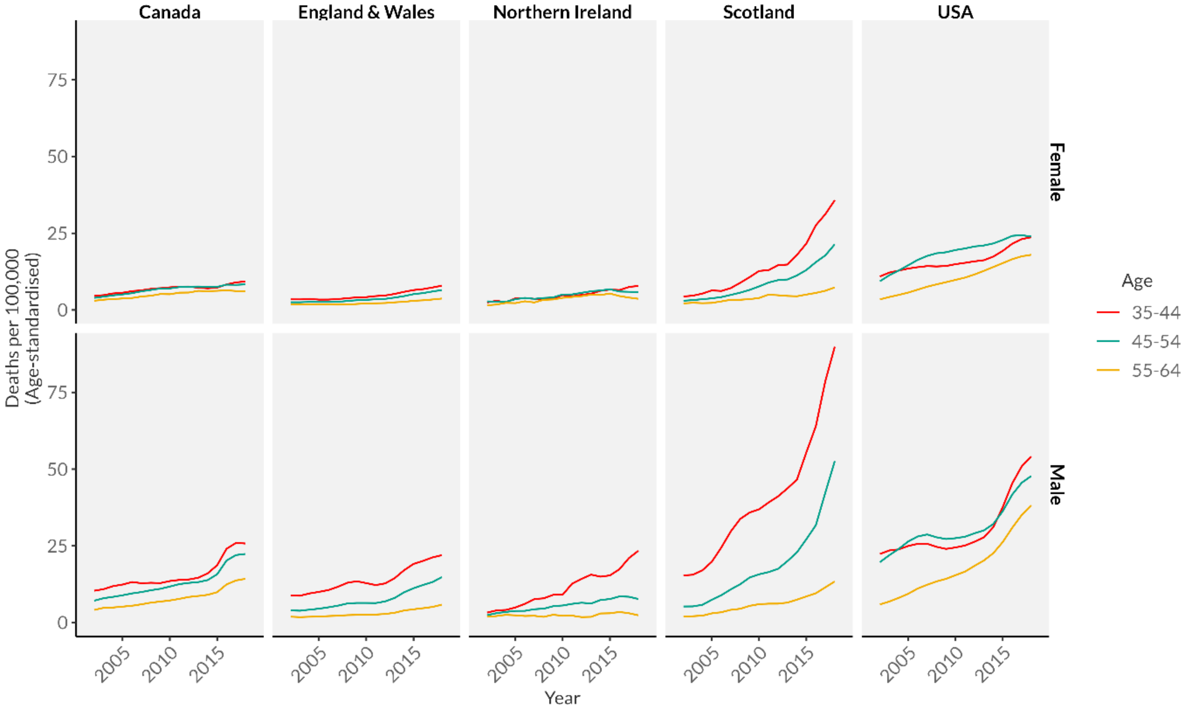

Figure S1b - Suicide mortality by age and sex in Canada, the UK and the US, 2001-2019

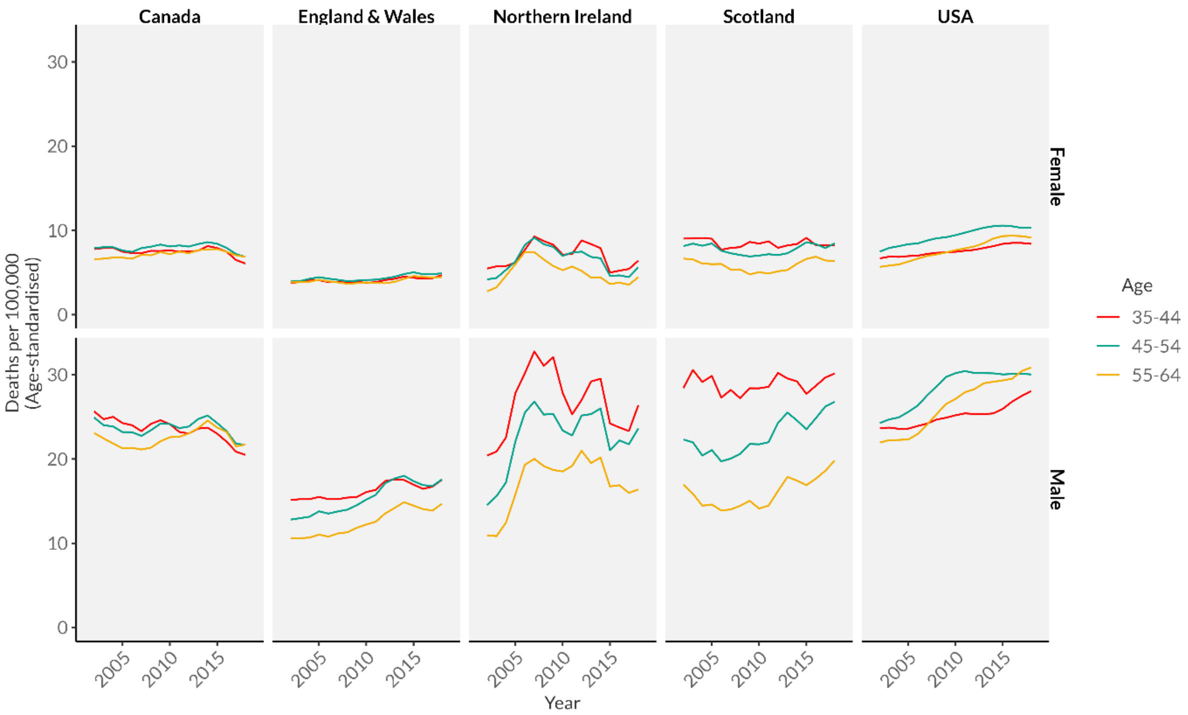

Figure S1c – alcohol-specific mortality by age and sex in Canada, the UK and the US, 2001-2019

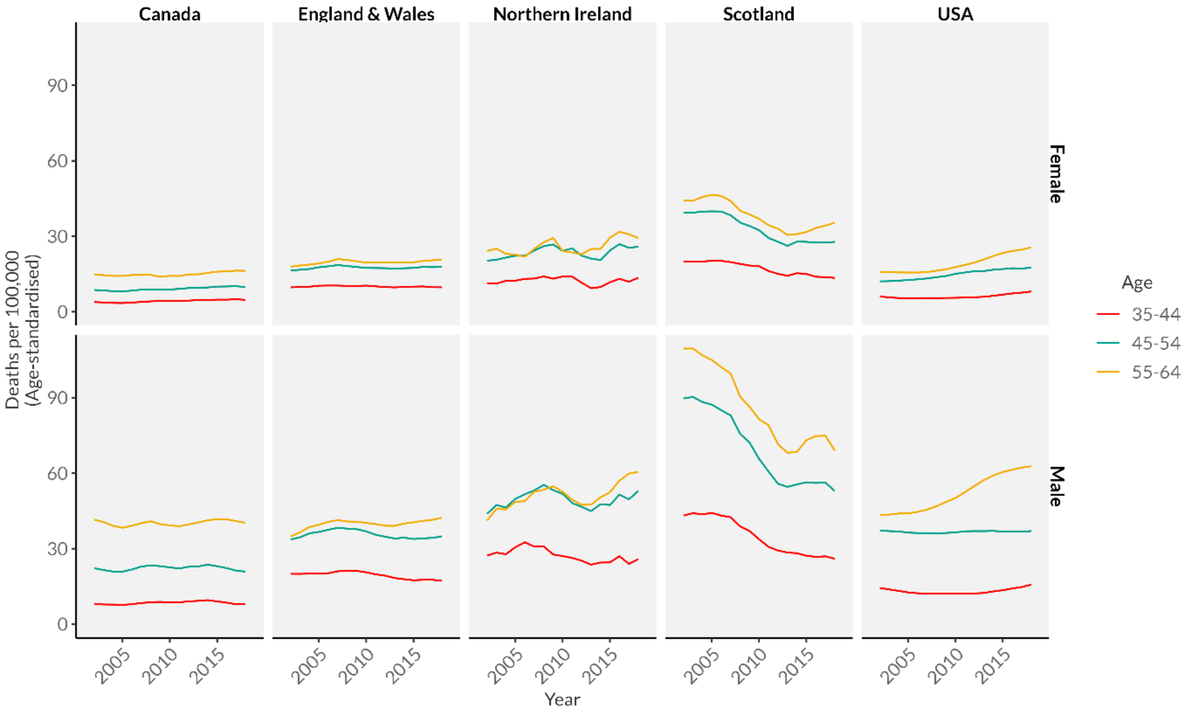

Figure S2a - Lexis surface showing age-specific mortality rates for males by cause, year and country

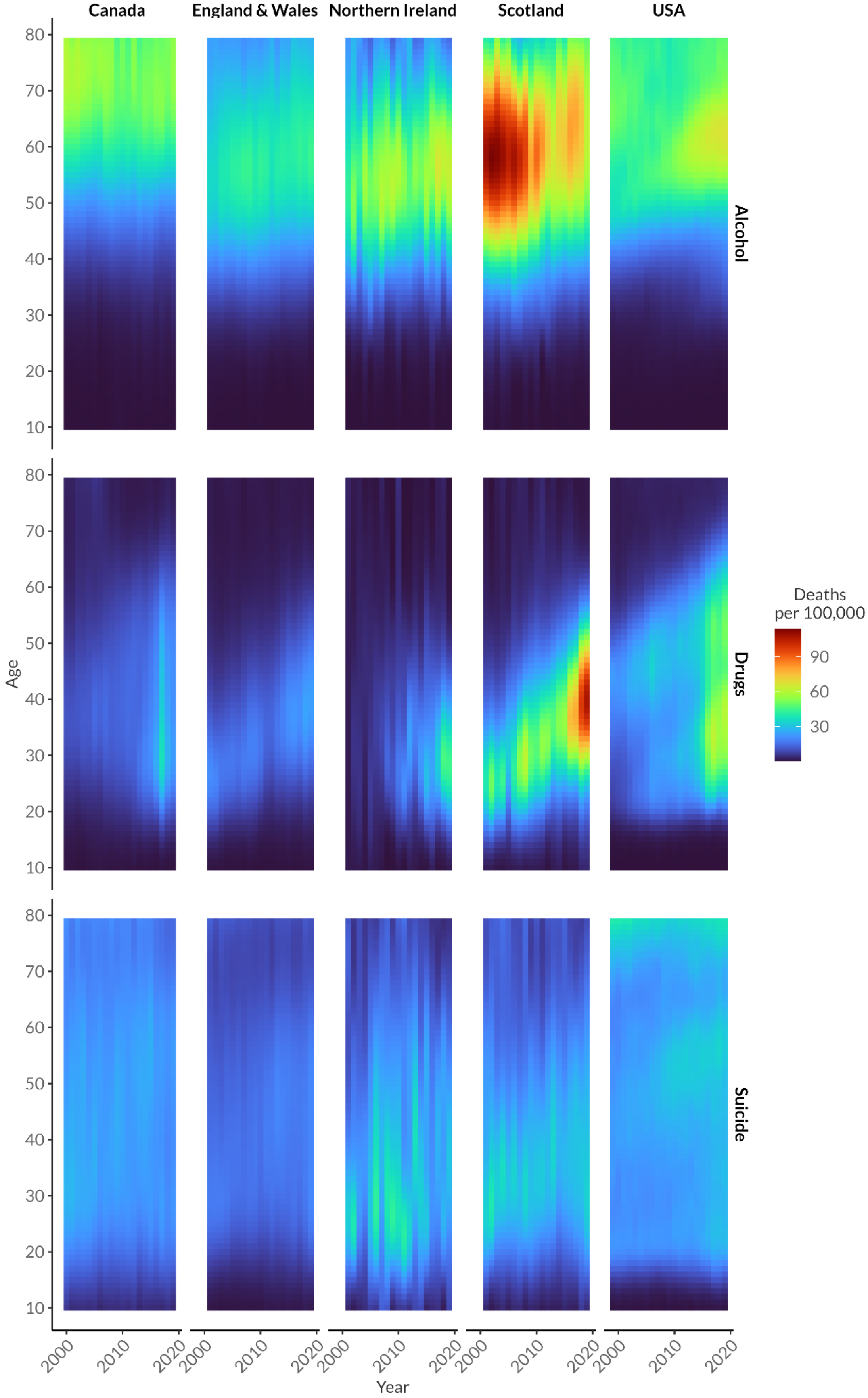

Figure S2b - Lexis surface showing age-specific mortality rates for females by cause, year and country

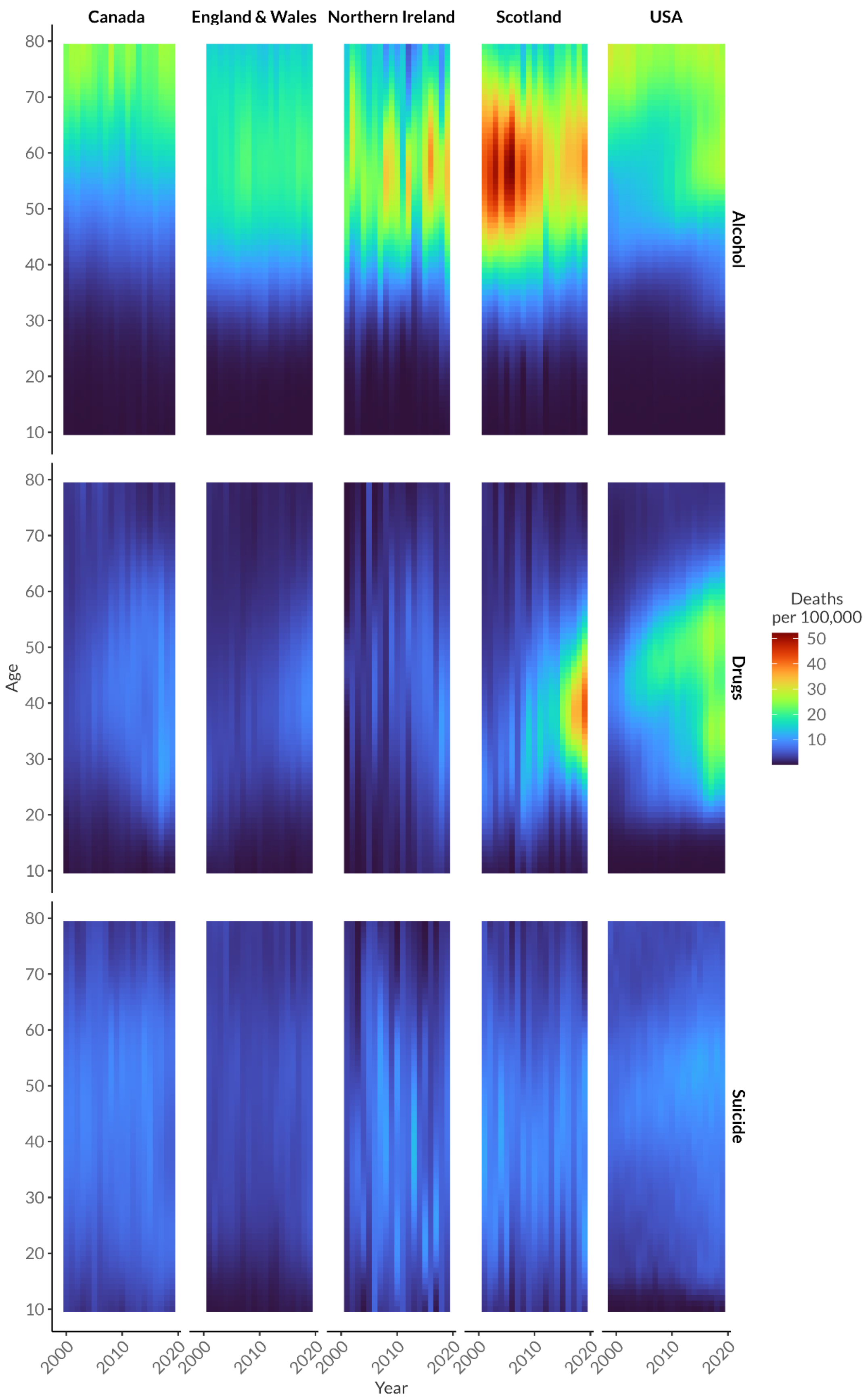

Figure S3a - Proportion of all deaths accounted for by alcohol, drugs and suicide among ages 35-44

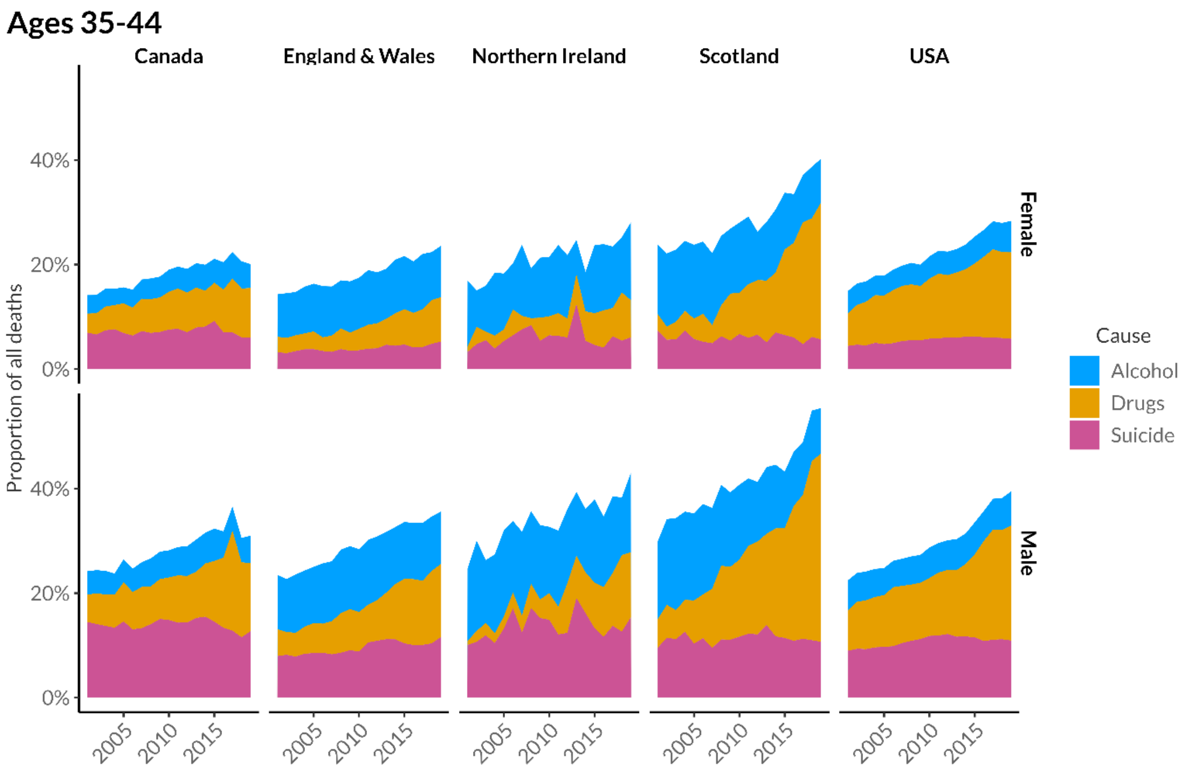

Figure S3b - Proportion of all deaths accounted for by alcohol, drugs and suicide among ages 45-54

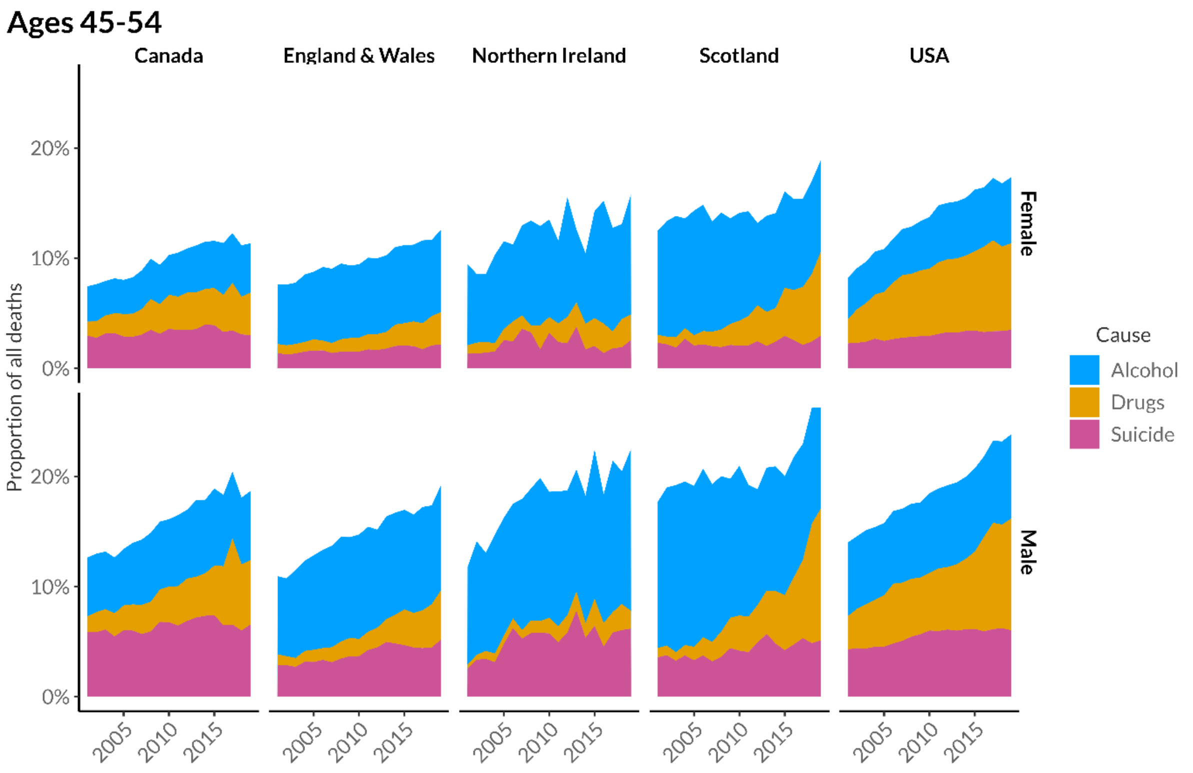

Figure S3c - Proportion of all deaths accounted for by alcohol, drugs and suicide among ages 55-64

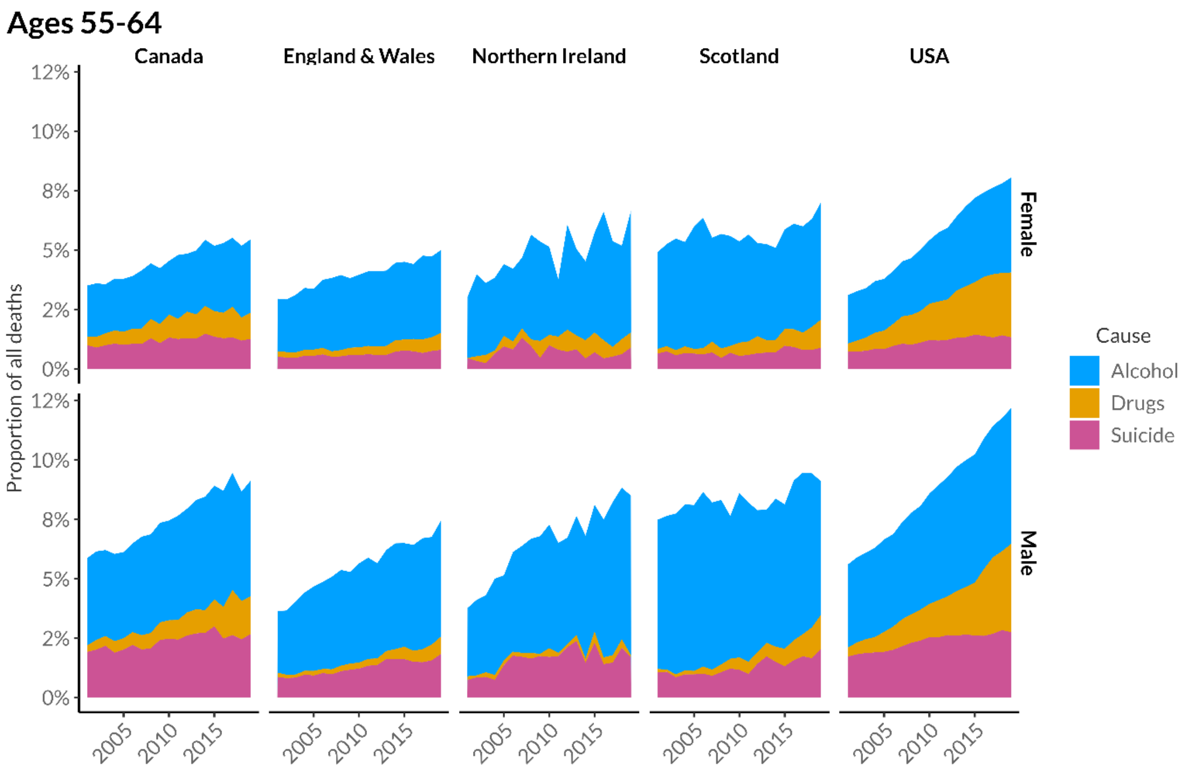

Figure S4 - All-cause mortality in ages 35-64 by sex in Canada, the UK and the USA

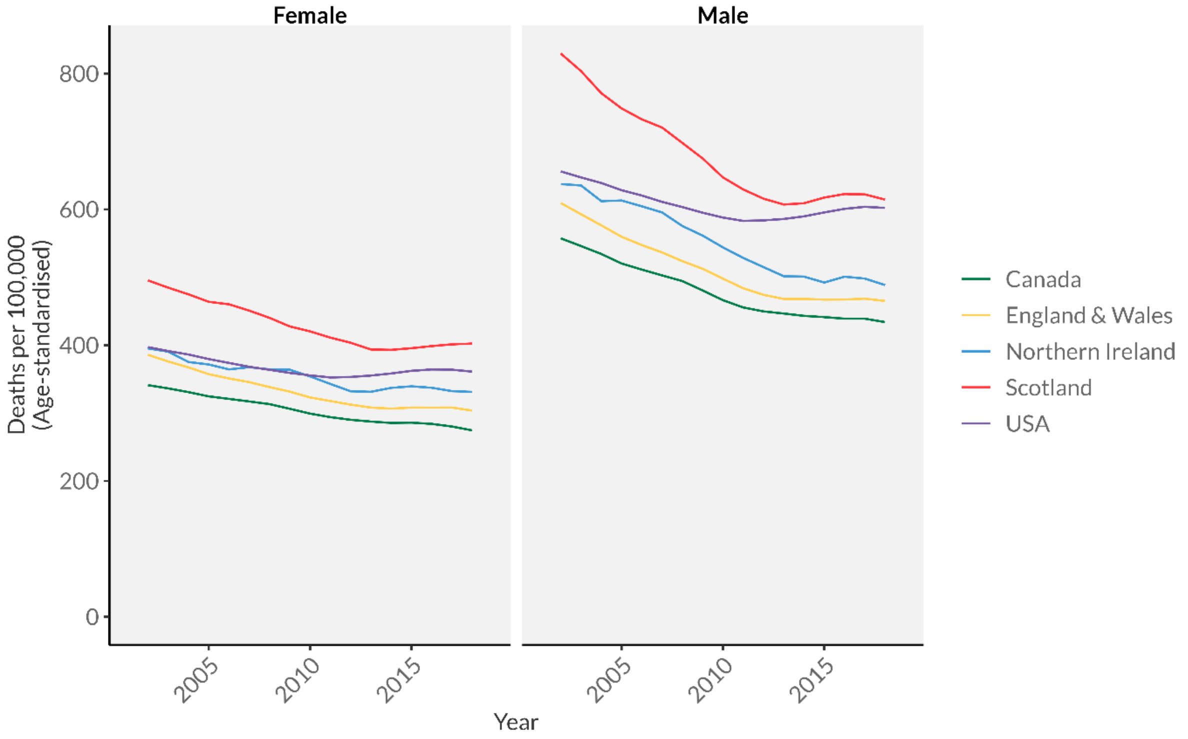

Figure S5 – Combined 'deaths of despair' mortality in ages 35-64 by sex in Canada, the UK and the USA

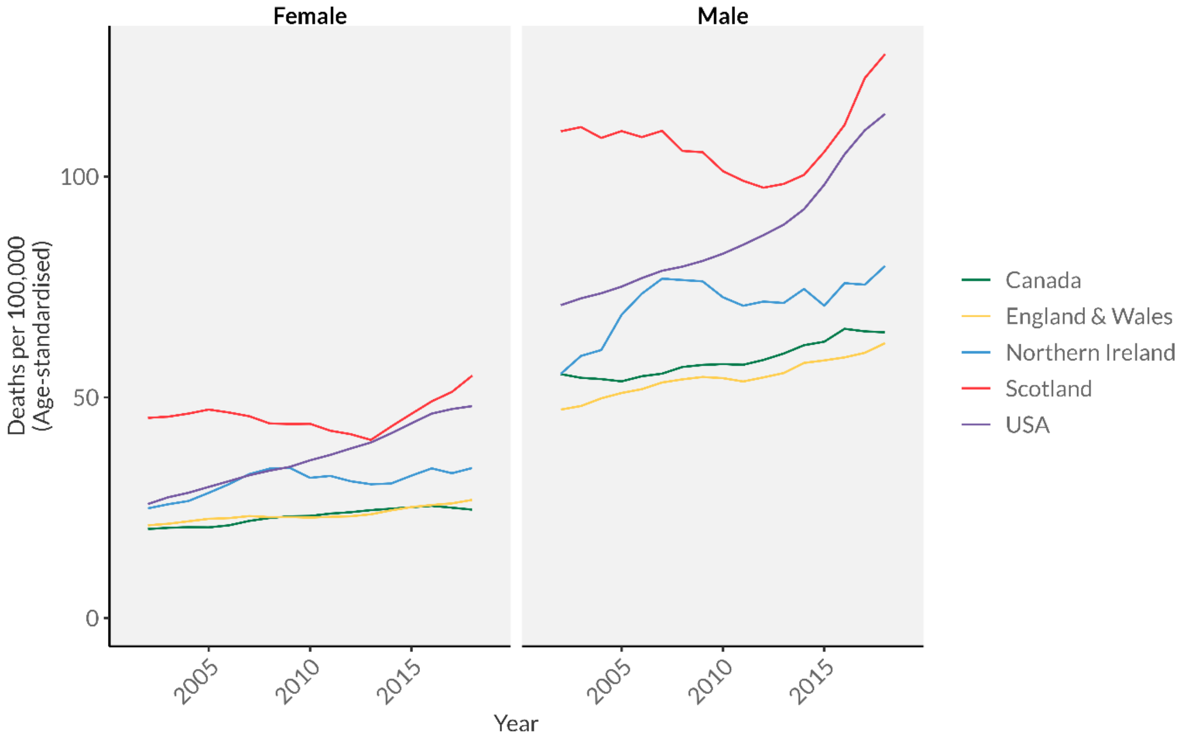

Figure S5a - Drug-related mortality in ages 35-64 by sex in Canada, the UK and the USA

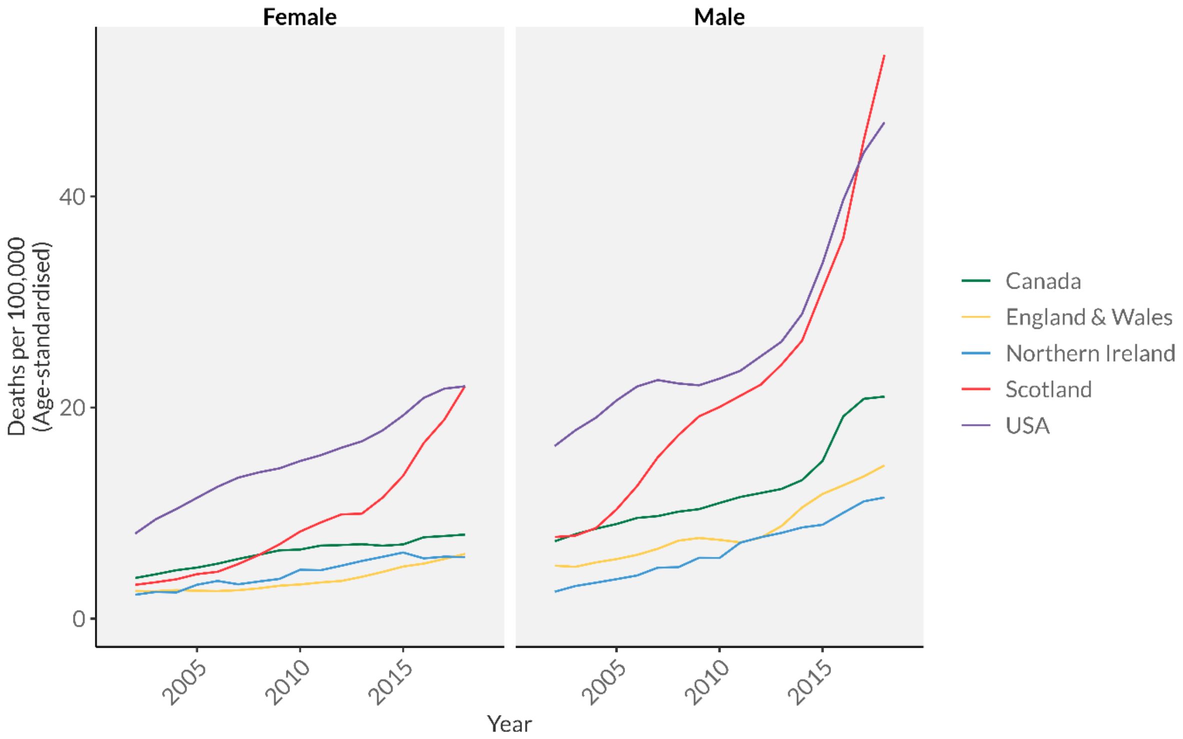

Figure S5b - Suicide mortality in ages 35-64 by sex in Canada, the UK and the USA

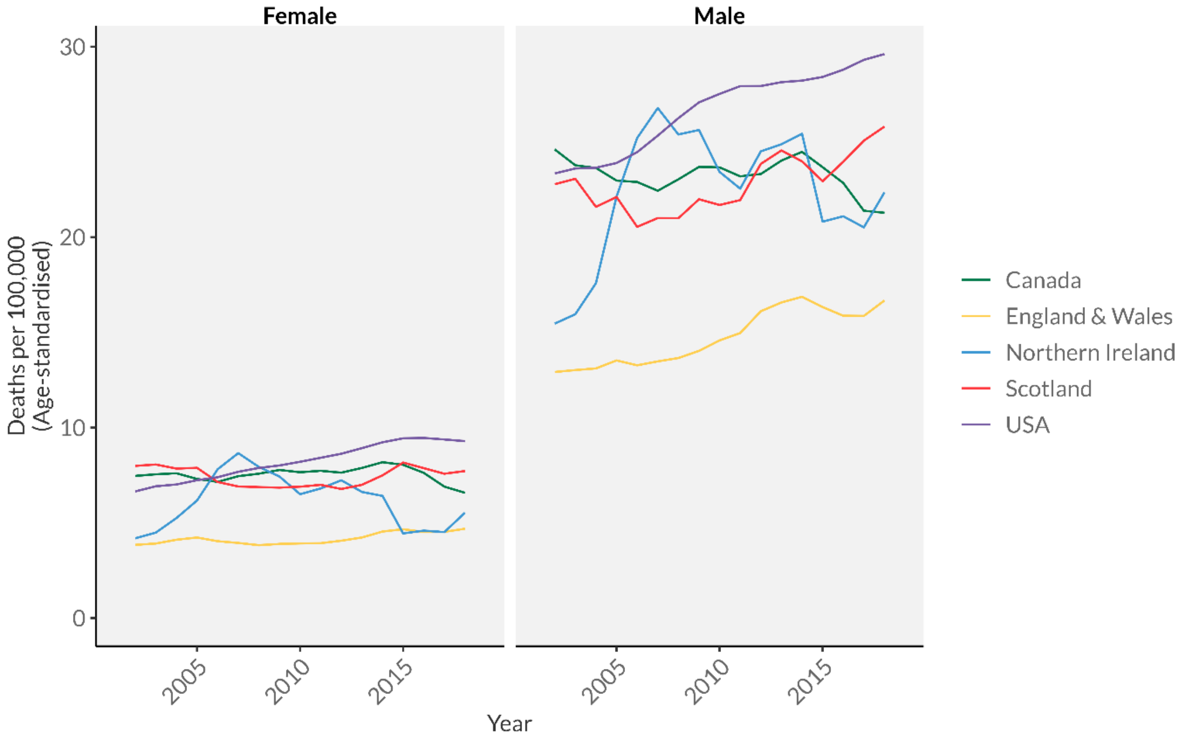

Figure S5c – Alcohol-specific mortality in ages 35-64 by sex in Canada, the UK and the USA

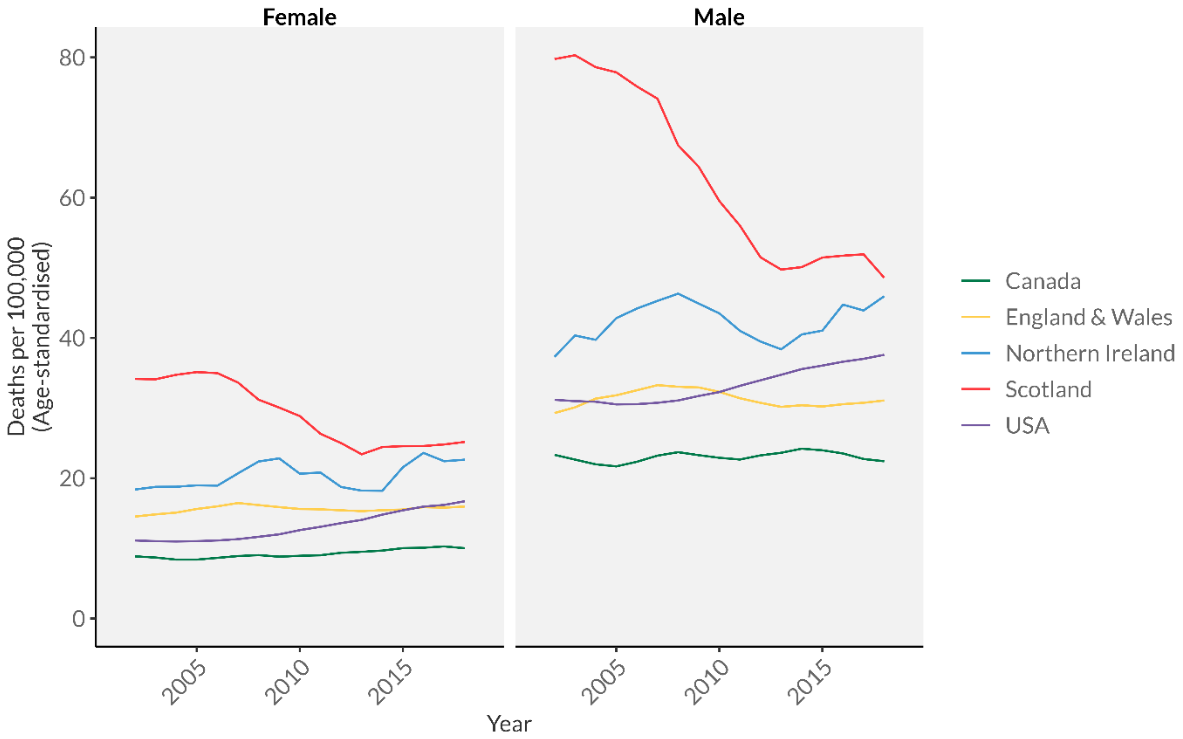

Table S1 - ICD-10 codes used to define 'deaths of despair' categories

Cause group	ICD-10 codes included
Suicide	U03, X60-84, Y87
Alcohol-related	K70, K73-74, F10, X45, Y15
Drug-related	F11-16, F18-19, X40-44, X85, Y10-14

Table S2a - Male mortality rates (per 100,000 population) by cause, age group and year for Canada

Age group	2001	2002	2003	2004	2005	2006	2007	2008	2009	2010	2011	2012	2013	2014	2015	2016	2017	2018	2019
All-Cause																			
35-44	183.6	179.4	182.6	177.9	179.1	174.9	173.3	170.6	169.0	164.1	157.8	157.5	156.2	157.2	158.0	162.4	167.9	167.9	162.0
45-54	424.3	419.3	415.8	404.1	399.2	388.9	388.2	383.5	373.5	361.3	350.2	347.6	347.7	344.4	340.3	344.6	342.0	344.2	334.8
55-64	1156.0	1135.9	1104.2	1075.0	1045.1	1006.9	1010.8	988.8	949.3	917.0	901.3	886.8	884.5	877.6	860.8	863.1	845.6	845.1	825.7
Combined 'Deaths of Despair'																			
35-44	44.4	43.8	44.3	42.3	47.4	43.2	44.9	45.5	47.2	46.3	45.5	45.6	47.3	49.6	51.1	51.6	61.4	51.3	50.1
45-54	53.7	54.5	54.8	51.1	53.6	54.3	55.4	57.1	59.4	58.2	57.8	59.0	62.1	61.6	64.3	63.2	70.0	62.3	62.6
55-64	67.9	69.9	68.6	65.0	64.0	65.4	68.5	68.0	69.8	68.3	69.0	70.5	73.6	74.2	76.8	75.2	80.0	73.3	75.5
Alcohol																			
35-44	8.2	8.0	8.2	7.2	7.9	7.8	8.2	9.1	8.9	8.5	8.6	9.0	9.6	9.2	9.8	8.2	7.7	7.9	8.4
45-54	22.7	22.2	21.8	20.5	20.5	21.7	23.0	24.0	23.0	22.1	22.6	21.7	24.2	23.0	23.8	22.3	20.7	20.9	21.0
55-64	42.5	42.3	39.9	39.5	37.9	37.6	41.9	41.0	39.7	38.5	39.6	38.7	40.7	41.9	41.2	42.2	41.7	38.9	40.2
Drugs																			
35-44	9.7	10.5	11.0	11.2	13.4	12.5	13.7	12.4	12.9	13.4	14.3	14.0	13.8	16.0	18.3	21.8	32.1	24.0	21.1
45-54	6.1	7.7	7.7	8.5	9.0	9.2	10.3	10.3	11.0	11.7	12.5	13.4	12.9	13.3	15.4	18.5	26.8	20.7	19.6
55-64	3.2	4.7	4.6	5.2	5.0	5.4	6.1	6.4	7.1	7.1	7.5	8.6	9.0	8.4	9.8	11.5	16.1	13.6	13.1
Suicide																			
35-44	26.6	25.3	25.1	23.8	26.1	22.9	23.1	24.0	25.4	24.4	22.6	22.6	23.8	24.4	23.0	21.7	21.5	19.4	20.6
45-54	24.9	24.5	25.4	22.1	24.1	23.4	22.1	22.8	25.4	24.4	22.6	23.9	25.0	25.3	25.2	22.3	22.4	20.7	22.0
55-64	22.3	22.9	24.1	20.3	21.1	22.3	20.5	20.5	23.0	22.7	22.0	23.2	23.9	23.9	25.9	21.5	22.3	20.7	22.1

Table S2b - Female mortality rates (per 100,000 population) by cause, age group and year in Canada

Age group	2001	2002	2003	2004	2005	2006	2007	2008	2009	2010	2011	2012	2013	2014	2015	2016	2017	2018	2019
All-Cause																			
35-44	112.1	111.7	110.5	106.9	108.8	106.3	105.2	108.2	107.3	100.2	100.6	100.8	96.3	98.9	95.4	98.6	96.1	95.5	94.6
45-54	268.6	268.4	264.8	257.6	257.2	251.8	252.1	251.8	246.4	235.9	234.3	232.9	226.2	227.2	223.4	227.3	219.6	213.2	212.6
55-64	685.8	685.2	672.6	657.5	647.5	631.1	629.3	617.5	597.5	586.7	576.6	569.2	564.0	560.8	566.1	564.0	555.1	540.0	530.0
Combined 'Deaths of Despair'																			
35-44	15.9	15.8	17.0	16.4	17.0	16.1	18.0	18.8	19.0	19.1	19.7	19.3	19.5	19.7	20.1	20.2	21.5	19.7	19.0
45-54	20.0	20.5	21.0	21.0	20.7	20.8	22.4	25.1	23.1	24.3	24.6	25.3	25.2	26.1	25.8	25.9	27.0	23.8	24.1
55-64	24.1	24.7	24.0	25.0	24.6	24.7	26.1	27.5	25.4	26.7	27.7	27.6	28.1	30.5	29.4	29.9	30.7	28.0	28.9
Alcohol																			
35-44	4.0	3.9	3.8	3.3	3.3	3.7	3.9	4.3	4.3	4.2	4.2	4.6	4.5	4.9	4.3	5.2	4.8	5.0	4.3
45-54	8.6	9.0	8.3	8.2	8.0	8.3	8.9	9.2	8.8	8.5	9.4	9.3	9.6	9.8	9.5	10.7	9.9	9.9	9.5
55-64	14.7	15.4	13.9	14.3	14.4	14.0	15.4	14.4	14.1	13.2	15.5	13.9	15.2	15.6	15.6	16.5	16.1	16.3	16.4
Drugs																			
35-44	4.1	4.5	5.0	4.9	6.2	5.7	6.5	7.0	7.1	7.2	7.8	7.6	7.4	6.8	7.0	8.1	9.9	8.9	9.0
45-54	3.4	4.0	4.4	4.7	5.3	5.3	5.9	7.1	6.6	7.3	7.1	7.9	7.6	7.3	7.6	7.7	9.5	7.3	8.2
55-64	2.5	3.1	3.4	3.6	3.6	4.0	4.1	5.0	4.8	5.7	4.9	6.3	5.8	6.5	6.1	6.1	7.1	5.3	5.8
Suicide																			
35-44	7.8	7.4	8.2	8.1	7.5	6.8	7.6	7.5	7.6	7.6	7.7	7.1	7.6	8.0	8.8	6.9	6.7	5.8	5.7
45-54	8.0	7.4	8.4	8.2	7.4	7.2	7.7	8.8	7.7	8.4	8.1	8.1	8.1	9.0	8.7	7.5	7.6	6.5	6.5
55-64	6.9	6.2	6.7	7.0	6.6	6.7	6.6	8.0	6.5	7.8	7.3	7.4	7.2	8.3	7.7	7.3	7.4	6.5	6.7

Table S3a - Male mortality rates (per 100,000 population) by cause, age group and year for England & Wales

Age group	2001	2002	2003	2004	2005	2006	2007	2008	2009	2010	2011	2012	2013	2014	2015	2016	2017	2018	2019
All-Cause																			
35-44	190.7	188.3	188.4	183.7	181.3	181.5	177.1	179.8	177.7	168.9	162.8	155.2	161.4	159.5	160.7	162.9	161.7	168.0	162.8
45-54	461.4	453.4	451.1	433.0	428.0	424.2	412.7	410.8	402.8	393.9	382.2	368.3	375.6	370.5	372.9	374.7	374.4	382.0	371.0
55-64	1280.1	1251.8	1226.2	1159.3	1129.0	1100.6	1070.2	1044.7	1003.7	991.9	954.9	929.1	926.8	913.1	917.4	916.9	907.3	910.9	888.6
Combined 'Deaths of Despair'																			
35-44	44.8	42.8	44.4	44.7	45.4	46.7	46.2	51.0	51.5	48.0	49.2	47.9	51.2	52.1	54.1	54.5	54.1	58.3	58.0
45-54	50.5	48.7	52.0	53.5	55.0	56.6	56.6	59.6	58.4	58.0	59.0	55.9	61.5	62.0	63.3	62.0	64.5	66.4	71.1
55-64	46.6	45.9	49.5	51.3	52.8	53.6	54.4	56.1	53.1	56.0	56.3	52.5	57.6	59.3	59.6	58.9	60.9	61.6	66.1
Alcohol																			
35-44	19.8	19.1	21.0	19.7	19.5	21.0	20.3	21.9	21.4	20.3	20.2	18.9	18.7	17.4	17.5	17.5	17.9	17.5	16.3
45-54	32.7	32.0	36.1	35.5	36.7	37.9	38.0	39.0	36.9	37.4	36.4	32.8	35.0	34.3	33.7	33.5	35.1	34.3	35.3
55-64	33.1	33.8	37.5	38.2	40.1	40.3	41.6	42.1	38.7	41.4	40.7	37.2	39.4	40.6	40.0	40.8	42.3	41.3	43.3
Drugs																			
35-44	9.8	8.2	8.6	9.5	10.4	10.1	11.3	13.6	14.0	12.8	11.8	12.1	14.4	16.9	19.9	20.6	19.9	23.3	22.8
45-54	4.6	3.7	3.6	4.3	4.8	4.5	5.6	6.4	6.7	6.1	6.3	6.6	7.8	9.7	12.1	11.8	12.9	15.0	16.6
55-64	2.3	1.8	1.6	1.9	2.2	1.9	2.3	2.4	2.6	2.5	2.8	2.6	3.1	3.9	4.8	4.3	5.0	6.0	6.5
Suicide																			
35-44	15.2	15.4	14.8	15.5	15.4	15.6	14.7	15.5	16.1	14.9	17.2	16.9	18.1	17.8	16.7	16.4	16.3	17.4	18.9
45-54	13.2	13.0	12.3	13.7	13.5	14.2	12.9	14.2	14.8	14.5	16.2	16.5	18.7	17.9	17.5	16.7	16.5	17.1	19.1
55-64	11.1	10.3	10.3	11.2	10.5	11.4	10.5	11.6	11.8	12.1	12.8	12.8	15.1	14.7	14.8	13.8	13.5	14.3	16.3

Table S3b - Female mortality rates (per 100,000 population) by cause, age group and year in England & Wales

Age group	2001	2002	2003	2004	2005	2006	2007	2008	2009	2010	2011	2012	2013	2014	2015	2016	2017	2018	2019
All-Cause																			
35-44	117.5	115.0	116.4	112.2	111.6	110.4	109.3	110.2	105.6	104.8	100.9	97.4	97.8	98.8	99.0	99.3	97.8	99.2	98.7
45-54	302.5	297.0	295.1	282.4	281.3	276.6	273.2	272.8	262.3	260.4	253.5	249.5	246.7	244.2	247.4	251.2	245.3	248.1	243.0
55-64	802.5	784.8	770.3	735.2	722.1	700.6	687.0	680.8	651.2	641.2	629.7	623.5	612.7	600.7	608.3	621.5	599.8	608.3	587.8
Combined 'Deaths of Despair'																			
35-44	16.8	16.7	17.1	17.7	18.2	17.5	17.3	18.7	17.7	18.3	19.1	18.0	18.8	20.7	21.4	20.4	21.6	22.2	23.2
45-54	23.0	22.6	23.0	24.1	24.7	25.5	24.7	26.0	24.5	24.6	25.5	24.9	25.3	26.9	27.7	28.2	28.5	28.9	30.5
55-64	23.7	23.0	24.0	25.1	24.3	26.3	26.3	26.9	24.9	25.4	25.9	25.6	25.4	26.8	27.4	27.4	28.6	28.8	29.4
Alcohol																			
35-44	9.6	9.8	9.6	10.0	10.2	10.8	10.2	10.2	10.4	10.2	10.6	9.5	9.4	10.1	10.1	9.8	10.4	9.1	9.7
45-54	16.4	16.4	16.4	17.3	17.3	18.5	18.4	18.8	17.3	17.3	17.6	17.2	17.2	17.2	17.5	17.5	18.5	17.2	18.2
55-64	17.7	17.4	18.7	19.1	18.4	20.1	21.2	21.5	19.0	19.6	19.7	19.7	19.4	19.6	19.9	19.6	21.1	20.6	20.5
Drugs																			
35-44	3.5	3.4	3.5	3.4	3.8	2.9	3.4	4.4	3.7	4.3	4.6	4.7	4.9	6.2	6.7	6.6	7.1	8.3	8.4
45-54	2.5	2.5	2.6	2.5	2.9	2.4	2.5	3.1	3.2	3.3	3.5	3.6	3.7	4.8	5.0	5.7	5.8	6.6	7.1
55-64	1.9	1.8	1.8	1.8	1.9	1.9	1.6	1.8	2.0	2.1	2.2	2.2	2.3	2.9	2.8	3.2	3.4	3.5	4.2
Suicide																			
35-44	3.8	3.5	4.0	4.2	4.2	3.8	3.6	4.2	3.7	3.8	3.9	3.9	4.5	4.4	4.6	4.1	4.1	4.8	5.2
45-54	4.2	3.7	4.0	4.3	4.5	4.5	3.8	4.1	4.0	4.0	4.4	4.1	4.4	4.9	5.1	5.1	4.3	5.2	5.3
55-64	4.1	3.8	3.6	4.1	4.0	4.3	3.6	3.6	3.8	3.7	3.9	3.7	3.6	4.4	4.8	4.6	4.0	4.6	4.7

For peer review only - http://bmjopen.bmj.com/site/about/guidelines.xhtml

Table S4a - Male mortality rates (per 100,000 population) by cause, age group and year for Northern Ireland

Age group	2001	2002	2003	2004	2005	2006	2007	2008	2009	2010	2011	2012	2013	2014	2015	2016	2017	2018	2019
All-Cause																			
35-44	192.7	200.4	170.8	199.3	199.3	211.6	223.7	199.2	204.4	207.7	177.2	189.6	188.9	173.4	184.3	172.7	193.7	183.7	190.4
45-54	465.7	497.4	438.8	479.3	450.5	475.5	480.3	440.9	431.9	443.7	396.4	398.5	417.9	375.3	393.1	388.9	405.0	395.8	378.1
55-64	1324.3	1385.5	1282.8	1282.1	1214.4	1205.1	1170.8	1137.3	1067.7	1092.9	1042.6	971.1	1011.6	940.0	977.1	977.9	967.5	947.7	876.9
Combined 'Deaths of Despair'																			
35-44	47.6	60.2	45.0	54.7	63.8	71.6	71.1	71.1	67.5	67.9	56.7	68.4	74.4	62.6	70.0	59.9	74.7	70.4	81.9
45-54	54.9	70.2	57.4	70.5	73.3	83.4	86.3	83.5	85.9	82.6	73.9	74.7	86.3	68.5	88.3	71.5	86.9	81.0	85.0
55-64	50.0	57.0	55.2	64.2	62.5	73.8	74.8	76.1	72.6	79.6	67.9	65.3	77.3	64.1	79.3	73.3	79.6	83.7	74.5
Alcohol																			
35-44	26.7	34.7	20.5	30.1	32.8	28.9	35.9	27.6	29.1	26.3	25.8	26.8	23.1	21.0	29.4	23.3	28.4	20.2	29.0
45-54	41.2	51.3	39.1	51.7	48.3	49.5	57.1	52.9	56.2	50.8	48.5	45.2	46.3	43.4	53.3	45.4	55.8	47.7	55.5
55-64	38.1	44.0	41.5	52.0	43.5	50.5	52.7	54.7	52.8	56.8	48.2	43.4	50.6	48.5	52.1	56.7	62.3	60.5	58.8
Drugs																			
35-44	1.6	4.1	4.1	3.7	4.6	6.5	7.2	9.2	7.3	10.6	9.4	18.0	15.3	13.6	16.1	16.5	19.6	26.9	23.6
45-54	1.8	2.2	3.2	3.8	3.5	4.2	3.8	5.1	4.7	6.3	5.8	6.3	7.4	5.0	9.7	8.3	7.5	9.4	6.2
55-64	2.0	1.2	2.5	2.6	2.4	2.0	2.0	2.6	1.0	4.1	1.5	1.2	2.6	1.7	4.6	2.8	3.0	3.4	0.8
Suicide																			
35-44	19.4	21.4	20.4	20.8	26.4	36.2	27.9	34.2	31.1	31.0	21.4	23.5	36.0	28.1	24.5	20.1	26.6	23.2	29.3
45-54	11.9	16.7	15.0	15.0	21.5	29.7	25.3	25.5	25.0	25.5	19.6	23.3	32.6	20.1	25.3	17.7	23.6	24.0	23.4
55-64	9.9	11.8	11.2	9.6	16.6	21.3	20.1	18.7	18.7	18.7	18.2	20.6	24.1	13.8	22.6	13.8	14.3	19.9	15.0

For peer review only

Table S4b - Female mortality rates (per 100,000 population) by cause, age group and year in Northern Ireland

Age group	2001	2002	2003	2004	2005	2006	2007	2008	2009	2010	2011	2012	2013	2014	2015	2016	2017	2018	2019
All-Cause																			
35-44	119.4	121.7	121.7	119.8	110.2	122.8	121.5	127.5	116.4	122.3	112.9	108.8	102.3	107.8	111.8	98.3	103.7	105.9	115.0
45-54	310.2	311.2	301.8	300.3	275.8	298.1	292.2	297.0	288.4	292.7	274.7	267.2	259.3	267.3	281.2	257.1	261.0	267.6	274.0
55-64	809.9	819.0	772.8	774.6	722.8	740.3	711.3	710.9	727.4	704.9	656.3	652.0	659.5	663.0	689.5	688.4	654.1	663.1	639.7
Combined 'Deaths of Despair'																			
35-44	20.1	18.3	19.4	22.1	20.1	24.8	28.9	24.6	24.8	26.3	26.8	23.7	25.3	20.0	26.5	23.5	24.3	26.7	32.3
45-54	29.4	26.6	25.8	30.9	31.8	33.5	37.9	39.8	37.3	39.6	31.8	41.5	32.7	27.9	40.2	39.1	33.3	35.0	43.3
55-64	24.6	32.6	28.0	29.8	31.9	31.2	33.3	40.2	39.0	36.3	24.7	39.6	33.3	30.1	39.6	45.6	35.2	34.4	42.5
Alcohol																			
35-44	15.0	8.5	10.7	14.4	11.8	10.9	16.6	12.2	13.4	13.9	14.7	13.2	6.7	8.1	14.6	12.4	12.2	11.2	17.1
45-54	22.9	19.4	18.6	24.0	22.0	20.8	23.9	28.3	26.0	25.9	20.6	29.1	17.2	17.1	27.4	28.7	24.5	23.1	29.9
55-64	20.8	28.2	23.4	23.7	21.8	22.6	21.2	31.3	30.4	26.1	15.8	28.8	24.0	22.0	29.0	37.2	29.1	26.1	32.7
Drugs																			
35-44	1.2	4.0	2.0	3.0	2.4	6.0	3.1	1.7	5.1	4.4	4.9	4.0	5.9	6.1	6.8	7.0	5.6	9.8	8.2
45-54	2.4	3.1	2.9	2.3	2.8	5.4	3.6	1.9	6.3	4.2	4.7	6.3	5.7	6.2	7.1	6.9	4.0	6.9	6.4
55-64	0.2	1.6	2.7	1.0	3.2	2.5	2.8	2.0	5.2	3.1	3.6	6.0	3.8	5.1	5.7	5.2	2.6	4.2	4.1
Suicide																			
35-44	3.9	5.8	6.7	4.7	5.9	7.9	9.2	10.7	6.3	7.9	7.2	6.6	12.7	5.8	5.1	4.0	6.5	5.7	7.0
45-54	4.1	4.2	4.3	4.6	7.1	7.3	10.5	9.7	5.0	9.5	6.5	6.2	9.8	4.6	5.7	3.6	4.7	5.1	7.0
55-64	3.6	2.8	1.9	5.1	6.9	6.1	9.4	6.8	3.5	7.1	5.3	4.8	5.5	3.0	4.9	3.1	3.4	4.1	5.8

ew only

Table S5a - Male mortality rates (per 100,000 population) by cause, age group and year for Scotland

Age group	2001	2002	2003	2004	2005	2006	2007	2008	2009	2010	2011	2012	2013	2014	2015	2016	2017	2018	2019
All-Cause																			
35-44	274.9	267.0	254.2	259.0	254.9	268.9	262.0	261.1	249.4	244.0	237.5	232.9	240.2	231.4	237.8	265.8	266.1	273.8	284.2
45-54	649.0	634.1	606.2	591.9	580.9	580.7	567.1	557.3	530.8	520.1	495.1	493.0	502.7	491.9	507.0	536.6	520.6	525.7	529.8
55-64	1708.3	1712.4	1635.3	1538.6	1468.8	1429.7	1406.5	1370.3	1287.4	1257.4	1194.7	1175.9	1149.4	1121.3	1172.9	1163.9	1101.5	1106.0	1076.2
Combined 'Deaths of Despair'																			
35-44	82.2	91.2	87.4	92.3	89.9	99.7	95.2	106.4	98.0	99.5	99.7	96.2	106.0	103.2	102.9	125.2	130.2	150.5	157.5
45-54	114.9	120.5	116.5	115.7	111.3	120.3	109.5	111.6	105.1	109.1	95.3	92.9	104.6	102.9	101.5	116.9	119.6	138.1	139.2
55-64	127.9	131.0	126.7	125.0	119.1	123.7	115.5	114.0	98.4	108.3	98.0	92.8	90.9	93.9	95.4	106.3	104.3	104.4	98.0
Alcohol																			
35-44	40.9	43.8	44.8	43.8	42.4	46.5	40.6	40.4	35.6	35.0	30.6	26.6	30.6	28.2	25.8	27.6	26.9	26.4	24.7
45-54	86.1	91.1	92.0	87.9	85.0	88.9	81.3	78.7	67.0	70.8	59.3	51.9	56.2	55.7	54.8	58.7	54.9	55.4	48.5
55-64	107.3	110.7	110.7	107.4	102.6	105.0	98.9	94.8	77.3	87.0	80.0	70.2	64.4	69.8	71.3	78.2	74.9	71.7	60.4
Drugs																			
35-44	15.1	16.8	14.1	15.9	21.1	22.5	29.7	36.9	34.8	36.0	40.0	41.4	42.0	47.8	50.0	68.7	73.3	94.0	102.4
45-54	5.5	5.5	4.7	5.6	7.1	9.7	10.0	12.8	14.7	16.6	15.9	16.9	19.8	23.5	25.4	32.7	37.1	57.1	63.6
55-64	2.3	1.7	2.0	2.6	2.1	4.3	3.7	4.5	5.3	6.6	6.1	5.8	6.6	7.2	8.6	9.8	10.3	14.4	15.6
Suicide																			
35-44	26.2	30.6	28.4	32.6	26.3	30.6	24.9	29.1	27.7	28.5	29.1	28.2	33.4	27.1	27.2	28.9	30.0	30.1	30.4
45-54	23.2	23.9	19.8	22.2	19.2	21.8	18.2	20.2	23.4	21.7	20.1	24.2	28.6	23.7	21.4	25.5	27.7	25.6	27.1
55-64	18.3	18.6	14.0	15.0	14.4	14.4	12.9	14.7	15.8	14.7	11.9	16.8	19.9	16.9	15.5	18.3	19.2	18.3	22.0

eview only

For peer review only - http://bmjopen.bmj.com/site/about/guidelines.xhtml

Table S5b - Female mortality rates (per 100,000 population) by cause, age group and year in Scotland

Age group	2001	2002	2003	2004	2005	2006	2007	2008	2009	2010	2011	2012	2013	2014	2015	2016	2017	2018	2019
All-Cause																			
35-44	149.2	147.2	139.8	148.5	146.7	146.0	143.7	144.5	144.9	133.4	142.0	132.1	129.8	133.1	141.3	146.4	141.8	149.7	153.4
45-54	387.9	382.8	376.1	366.9	367.0	363.6	362.0	347.5	344.8	328.6	336.3	317.0	317.7	314.8	327.2	333.5	327.8	338.9	341.1
55-64	1014.3	1009.6	1019.0	934.6	932.2	919.3	909.7	865.6	838.1	835.8	810.4	794.0	778.8	748.0	766.4	766.3	754.3	769.4	759.9
Combined 'Deaths of Despair'																			
35-44	35.6	32.6	32.0	36.4	34.9	35.6	31.9	36.8	38.9	37.4	41.4	34.7	36.5	40.7	47.7	49.0	52.6	58.0	61.7
45-54	48.4	51.2	52.0	49.9	52.5	54.0	48.4	49.2	47.0	46.4	48.0	41.9	44.0	44.4	52.6	51.3	50.5	57.8	64.7
55-64	49.9	53.1	55.9	49.9	55.8	58.5	50.3	49.2	46.9	44.9	45.9	42.1	40.9	38.1	45.0	46.8	45.3	48.6	53.3
Alcohol																			
35-44	19.9	20.6	19.3	19.9	20.7	20.2	19.8	19.2	18.0	17.9	18.4	12.3	14.5	16.1	15.3	13.7	12.8	14.8	12.8
45-54	36.7	40.2	41.4	36.5	41.5	41.7	36.3	36.9	33.1	32.2	32.0	23.7	27.7	27.2	28.7	27.5	26.3	28.7	28.4
55-64	41.3	43.4	47.9	41.0	47.9	50.4	39.8	41.7	38.8	35.6	36.4	31.1	31.5	28.9	32.2	34.0	33.7	34.9	37.5
Drugs																			
35-44	4.8	3.7	4.7	5.6	5.7	7.8	5.0	8.5	13.0	10.5	14.6	13.8	15.4	15.3	23.2	26.5	33.1	34.0	40.2
45-54	2.8	2.7	3.5	3.5	3.4	4.4	4.8	5.5	6.6	7.5	9.0	10.4	9.8	9.6	14.3	15.2	17.2	20.9	26.1
55-64	2.0	2.2	2.2	2.7	1.8	2.4	4.1	3.4	2.4	4.7	4.6	5.7	4.1	3.9	5.4	5.8	5.5	7.5	9.0
Suicide																			
35-44	10.9	8.2	7.9	11.0	8.4	7.6	7.1	9.1	7.9	8.9	8.5	8.7	6.6	9.3	9.2	8.8	6.8	9.2	8.7
45-54	9.0	8.3	7.1	9.9	7.6	7.9	7.3	6.7	7.3	6.7	7.0	7.8	6.4	7.6	9.6	8.5	7.0	8.2	10.2
55-64	6.6	7.5	5.9	6.3	6.0	5.7	6.3	4.0	5.7	4.6	4.8	5.3	5.4	5.3	7.5	7.1	6.0	6.2	6.8

iew only

Table S6a - Male mortality rates (per 100,000 population) by cause, age group and year for the USA

Age group	2001	2002	2003	2004	2005	2006	2007	2008	2009	2010	2011	2012	2013	2014	2015	2016	2017	2018	2019
All-Cause																			
35-44	259.2	258.0	254.3	244.0	244.0	240.4	233.5	225.5	223.9	212.3	213.6	211.8	212.9	216.6	226.2	244.2	252.2	251.7	257.3
45-54	551.3	557.1	561.1	551.0	556.2	548.7	536.6	531.3	524.2	508.9	507.4	500.0	497.9	494.0	494.0	496.7	495.4	490.2	484.9
55-64	1226.1	1216.1	1200.0	1157.0	1153.1	1126.4	1108.7	1110.6	1098.0	1082.8	1081.4	1090.8	1101.8	1112.5	1119.2	1124.2	1122.5	1124.4	1106.7
Combined 'Deaths of Despair'																			
35-44	58.2	61.6	61.4	60.1	60.5	63.0	62.1	61.0	61.2	61.0	63.2	63.8	64.6	68.1	76.0	87.3	96.0	96.2	101.6
45-54	77.2	81.2	84.9	84.9	87.8	92.5	91.6	93.2	92.5	94.1	95.9	96.0	96.9	98.7	102.5	108.4	115.2	113.7	115.5
55-64	68.7	71.7	73.2	73.0	76.8	77.5	81.9	86.5	88.6	93.0	96.7	101.3	107.0	111.1	114.6	122.6	128.4	132.3	134.8
Alcohol																			
35-44	14.9	14.3	14.0	13.1	12.6	12.1	12.1	12.1	11.9	12.4	12.1	12.0	12.6	12.7	13.8	14.0	14.8	15.5	16.9
45-54	36.9	36.8	37.8	36.4	36.5	36.2	36.0	36.2	35.7	36.8	36.8	37.1	37.0	36.8	37.4	36.3	36.9	37.0	37.1
55-64	42.9	43.5	43.7	43.5	44.8	43.8	45.3	47.5	47.9	50.3	52.3	54.8	57.6	59.3	60.4	61.7	62.1	63.0	63.2
Drugs																			
35-44	20.1	23.2	23.8	23.5	24.2	27.1	25.6	24.3	24.1	23.6	25.7	26.0	27.2	30.1	36.0	46.9	53.2	52.6	56.6
45-54	16.7	19.9	22.5	23.5	26.0	29.8	28.4	28.0	27.0	26.7	28.8	28.4	30.1	31.7	34.7	42.6	47.9	46.2	49.2
55-64	4.6	6.1	7.0	7.5	9.7	11.0	12.6	13.3	14.3	15.1	16.9	17.8	20.7	22.2	25.0	31.7	36.1	37.4	41.1
Suicide																			
35-44	23.3	24.1	23.6	23.5	23.7	23.7	24.4	24.5	25.1	25.1	25.4	25.8	24.8	25.3	26.1	26.4	28.0	28.1	28.1
45-54	23.7	24.6	24.5	25.0	25.3	26.5	27.2	28.9	29.7	30.6	30.3	30.5	29.9	30.2	30.4	29.5	30.4	30.5	29.3
55-64	21.2	22.2	22.5	22.0	22.3	22.7	24.0	25.6	26.4	27.6	27.5	28.7	28.7	29.6	29.2	29.2	30.2	31.9	30.5

For peer review only - http://bmjopen.bmj.com/site/about/guidelines.xhtml

Table S6b - Female mortality rates (per 100,000 population) by cause, age group and year in the USA

Age group	2001	2002	2003	2004	2005	2006	2007	2008	2009	2010	2011	2012	2013	2014	2015	2016	2017	2018	2019
All-Cause																			
35-44	147.6	148.2	146.9	142.4	142.0	140.4	135.8	133.7	134.0	128.1	129.6	127.9	129.4	133.1	134.6	141.0	142.9	141.6	142.8
45-54	319.8	321.9	322.5	318.1	323.8	320.7	317.2	318.8	319.9	311.7	314.4	311.1	311.6	313.9	313.5	314.1	308.8	301.0	295.1
55-64	773.4	758.0	753.7	724.8	713.0	697.9	677.4	673.1	662.4	647.9	647.4	649.3	653.5	664.3	668.9	675.4	678.4	671.6	667.8
Combined 'Deaths of Despair'																			
35-44	22.0	24.3	24.6	25.4	25.4	26.7	26.9	27.2	26.8	27.7	29.4	28.7	29.7	31.8	34.1	37.6	40.4	39.6	40.5
45-54	26.3	29.3	31.2	33.7	35.1	37.8	40.1	41.0	42.7	42.8	46.6	46.7	47.2	48.6	50.9	51.6	53.4	50.6	51.2
55-64	24.1	24.8	25.7	26.8	27.0	28.8	30.7	31.4	33.3	35.1	37.2	38.6	42.0	45.5	48.2	50.2	51.8	52.5	53.8
Alcohol																			
35-44	6.4	6.1	5.7	5.3	5.4	5.5	5.3	5.5	5.4	5.5	5.7	5.8	5.8	6.3	6.9	7.2	7.6	7.9	8.6
45-54	12.1	12.0	12.1	12.4	12.6	12.8	13.3	13.7	14.3	14.6	16.3	15.9	16.1	16.4	17.6	16.9	17.5	17.3	17.7
55-64	15.8	15.7	15.7	15.7	15.5	15.6	15.7	16.1	17.2	17.3	18.8	19.6	20.4	22.4	23.7	24.0	24.7	25.3	26.7
Drugs																			
35-44	9.2	11.3	12.3	13.0	13.2	14.3	14.3	14.3	13.9	14.7	16.1	15.2	16.2	17.2	18.8	21.8	24.2	23.3	23.6
45-54	7.0	9.8	11.3	12.8	14.4	16.5	18.0	18.3	19.1	19.0	20.5	20.6	21.0	21.5	22.6	24.4	25.5	23.1	23.2
55-64	2.6	3.5	4.2	4.9	5.5	6.5	7.8	8.4	8.8	9.9	10.7	11.0	13.0	14.2	14.8	16.8	18.1	17.6	18.2
Suicide																			
35-44	6.5	6.9	6.7	7.1	6.8	7.0	7.3	7.4	7.4	7.4	7.6	7.8	7.7	8.3	8.4	8.5	8.6	8.4	8.2
45-54	7.2	7.5	7.8	8.5	8.1	8.5	8.8	9.1	9.3	9.2	9.8	10.2	10.1	10.7	10.7	10.3	10.4	10.2	10.3
55-64	5.7	5.6	5.8	6.1	6.0	6.8	7.2	6.9	7.4	7.9	7.7	8.0	8.6	8.9	9.7	9.4	9.0	9.5	8.9

For peer review only - http://bmjopen.bmj.com/site/about/guidelines.xhtml

The RECORD statement – checklist of items, extended from the STROBE statement, that should be reported in observational studies using routinely collected health data.

	Item No.	STROBE items	Location in manuscript where items are reported	RECORD items	Location in manuscript where items are reported
**Title and abstract**					
	1	(a) Indicate the study's design with a commonly used term in the title or the abstract (b) Provide in the abstract an informative and balanced summary of what was done and what was found		RECORD 1.1: The type of data used should be specified in the title or abstract. When possible, the name of the databases used should be included.<br><br>RECORD 1.2: If applicable, the geographic region and timeframe within which the study took place should be reported in the title or abstract.<br><br>RECORD 1.3: If linkage between databases was conducted for the study, this should be clearly stated in the title or abstract.	Title and Abstract- we use publicly available death counts, so not individual level data
**Introduction**					
Background rationale	2	Explain the scientific background and rationale for the investigation being reported			p 1-4
Objectives	3	State specific objectives, including any prespecified hypotheses			p 1-4
**Methods**					
Study Design	4	Present key elements of study design early in the paper			p 5
Setting	5	Describe the setting, locations, and relevant dates, including periods of recruitment, exposure, follow-up, and data collection			p 5

For peer review only

Participants	6	*(a) Cohort study* - Give the eligibility criteria, and the sources and methods of selection of participants. Describe methods of follow-up<br>*Case-control study* - Give the eligibility criteria, and the sources and methods of case ascertainment and control selection. Give the rationale for the choice of cases and controls<br>*Cross-sectional study* - Give the eligibility criteria, and the sources and methods of selection of participants<br><br>*(b) Cohort study* - For matched studies, give matching criteria and number of exposed and unexposed<br>*Case-control study* - For matched studies, give matching criteria and the number of controls per case		RECORD 6.1: The methods of study population selection (such as codes or algorithms used to identify subjects) should be listed in detail. If this is not possible, an explanation should be provided.<br><br>RECORD 6.2: Any validation studies of the codes or algorithms used to select the population should be referenced. If validation was conducted for this study and not published elsewhere, detailed methods and results should be provided.<br><br>RECORD 6.3: If the study involved linkage of databases, consider use of a flow diagram or other graphical display to demonstrate the data linkage process, including the number of individuals with linked data at each stage.	N/A– We use national level death counts by age and sex, no individual records.
Variables	7	Clearly define all outcomes, exposures, predictors, potential confounders, and effect modifiers. Give diagnostic criteria, if applicable.		RECORD 7.1: A complete list of codes and algorithms used to classify exposures, outcomes, confounders, and effect modifiers should be provided. If these cannot be reported, an explanation should be provided.	N/A– descriptive analysis of mortality trends. Full code included and available.
Data sources/ measurement	8	For each variable of interest, give sources of data and details of methods of assessment (measurement).<br>Describe comparability of assessment methods if there is more than one group			

For peer review only

Bias	9	Describe any efforts to address potential sources of bias			
Study size	10	Explain how the study size was arrived at			Full national statistics
Quantitative variables	11	Explain how quantitative variables were handled in the analyses. If applicable, describe which groupings were chosen, and why			
Statistical methods	12	(a) Describe all statistical methods, including those used to control for confounding<br>(b) Describe any methods used to examine subgroups and interactions<br>(c) Explain how missing data were addressed<br>(d) *Cohort study* - If applicable, explain how loss to follow-up was addressed<br>*Case-control study* - If applicable, explain how matching of cases and controls was addressed<br>*Cross-sectional study* - If applicable, describe analytical methods taking account of sampling strategy<br>(e) Describe any sensitivity analyses			N/A- descriptive analysis of national mortality trends, not individual level data.
Data access and cleaning methods		..		RECORD 12.1: Authors should describe the extent to which the investigators had access to the database population used to create the study population.	N/A– publicly available national death counts

For peer review only

				RECORD 12.2: Authors should provide information on the data cleaning methods used in the study.	
Linkage		..		RECORD 12.3: State whether the study included person-level, institutional-level, or other data linkage across two or more databases. The methods of linkage and methods of linkage quality evaluation should be provided.	N/A
**Results**					
Participants	13	(a) Report the numbers of individuals at each stage of the study (*e.g.*, numbers potentially eligible, examined for eligibility, confirmed eligible, included in the study, completing follow-up, and analysed)<br>(b) Give reasons for non-participation at each stage.<br>(c) Consider use of a flow diagram		RECORD 13.1: Describe in detail the selection of the persons included in the study (*i.e.,* study population selection) including filtering based on data quality, data availability and linkage. The selection of included persons can be described in the text and/or by means of the study flow diagram.	N/A– publicly available national death counts
Descriptive data	14	(a) Give characteristics of study participants (*e.g.*, demographic, clinical, social) and information on exposures and potential confounders<br>(b) Indicate the number of participants with missing data for each variable of interest<br>(c) *Cohort study* - summarise follow-up time (*e.g.*, average and total amount)			N/A– publicly available national death counts
Outcome data	15	*Cohort study* - Report numbers of outcome events or summary measures over time<br>*Case-control study* - Report numbers in each exposure			N/A– publicly available national death counts

		category, or summary measures of exposure<br>*Cross-sectional study* - Report numbers of outcome events or summary measures			
Main results	16	(a) Give unadjusted estimates and, if applicable, confounder-adjusted estimates and their precision (e.g., 95% confidence interval). Make clear which confounders were adjusted for and why they were included<br>(b) Report category boundaries when continuous variables were categorized<br>(c) If relevant, consider translating estimates of relative risk into absolute risk for a meaningful time period			N/A– publicly available national death counts
Other analyses	17	Report other analyses done—e.g., analyses of subgroups and interactions, and sensitivity analyses			N/A– publicly available national death counts
**Discussion**					
Key results	18	Summarise key results with reference to study objectives			
Limitations	19	Discuss limitations of the study, taking into account sources of potential bias or imprecision. Discuss both direction and magnitude of any potential bias		RECORD 19.1: Discuss the implications of using data that were not created or collected to answer the specific research question(s). Include discussion of misclassification bias, unmeasured confounding, missing data, and changing eligibility over time, as they pertain to the study being reported.	N/A– publicly available national death counts
Interpretation	20	Give a cautious overall interpretation of results considering objectives,			Page 9-11

For peer review only

		limitations, multiplicity of analyses, results from similar studies, and other relevant evidence			
Generalisability	21	Discuss the generalisability (external validity) of the study results			Page 9-11
**Other Information**					
Funding	22	Give the source of funding and the role of the funders for the present study and, if applicable, for the original study on which the present article is based			Page 2- Funding acknowledgement
Accessibility of protocol, raw data, and programming code		..		RECORD 22.1: Authors should provide information on how to access any supplemental information such as the study protocol, raw data, or programming code.	Page 6– Link to reproducible data and code available

*Reference: Benchimol EI, Smeeth L, Guttmann A, Harron K, Moher D, Petersen I, Sørensen HT, von Elm E, Langan SM, the RECORD Working Committee. The REporting of studies Conducted using Observational Routinely-collected health Data (RECORD) Statement. *PLoS Medicine* 2015; in press.

*Checklist is protected under Creative Commons Attribution (CC BY) license.

For peer review only

BMJ Open

Comparing trends in Mid-life "Deaths of Despair" in the US, Canada, and UK, 2001-2019: Is the US an Anomaly?

Journal:	*BMJ Open*
Manuscript ID	bmjopen-2022-069905.R2
Article Type:	Original research
Date Submitted by the Author:	20-Jun-2023
Complete List of Authors:	Dowd, Jennifer; University of Oxford, Leverhulme Centre for Demographic Science; University of Oxford Nuffield College<br>Angus, Colin; University of Sheffield, ScHARR<br>Zajacova, Anna; University of Western Ontario,<br>Tilstra, Andrea; University of Oxford, Leverhulme Centre for Demographic Science; University of Oxford Nuffield College
**Primary Subject Heading**:	Public health
Secondary Subject Heading:	Epidemiology
Keywords:	Substance misuse < PSYCHIATRY, Suicide & self-harm < PSYCHIATRY, PUBLIC HEALTH

SCHOLARONE™
Manuscripts

I, the Submitting Author has the right to grant and does grant on behalf of all authors of the Work (as defined in the below author licence), an exclusive licence and/or a non-exclusive licence for contributions from authors who are: i) UK Crown employees; ii) where BMJ has agreed a CC-BY licence shall apply, and/or iii) in accordance with the terms applicable for US Federal Government officers or employees acting as part of their official duties; on a worldwide, perpetual, irrevocable, royalty-free basis to BMJ Publishing Group Ltd ("BMJ") its licensees and where the relevant Journal is co-owned by BMJ to the co-owners of the Journal, to publish the Work in this journal and any other BMJ products and to exploit all rights, as set out in our licence.

The Submitting Author accepts and understands that any supply made under these terms is made by BMJ to the Submitting Author unless you are acting as an employee on behalf of your employer or a postgraduate student of an affiliated institution which is paying any applicable article publishing charge ("APC") for Open Access articles. Where the Submitting Author wishes to make the Work available on an Open Access basis (and intends to pay the relevant APC), the terms of reuse of such Open Access shall be governed by a Creative Commons licence – details of these licences and which Creative Commons licence will apply to this Work are set out in our licence referred to above.

Other than as permitted in any relevant BMJ Author's Self Archiving Policies, I confirm this Work has not been accepted for publication elsewhere, is not being considered for publication elsewhere and does not duplicate material already published. I confirm all authors consent to publication of this Work and authorise the granting of this licence.

For peer review only

Comparing trends in Mid-life "Deaths of Despair" in the US, Canada, and UK, 2001-2019: Is the US an Anomaly?

Jennifer Beam Dowd*[1,2], Colin Angus[3], Anna Zajacova[4], Andrea M. Tilstra[1,2]

[1] Leverhulme Centre for Demographic Science, University of Oxford, Oxford, UK
[2] Nuffield College, University of Oxford, Oxford, UK
[3] School of Health and Related Research, University of Sheffield, Sheffield, UK
[4] Department of Sociology, University of Western Ontario, London, Ontario, Canada

*Corresponding Author
Jennifer Beam Dowd
Leverhulme Centre for Demographic Science
University of Oxford
42-42 Park End Street
Oxford OX1 1JD
United Kingdom
Jennifer.dowd@demography..ox.ac.uk
+ 44 7986830169

Key Words: Demography, Mortality, Suicide, Substance Abuse

Word Count: 3361

For peer review only

Abstract

Objectives: In recent years, "deaths of despair" due to drugs, alcohol, and suicide have contributed to rising mid-life mortality in the US. We examine whether despair-related deaths and mid-life mortality trends are also changing in peer countries the UK and Canada.

Design: Descriptive analysis of population mortality rates.

Setting: United States, United Kingdom (and constituent nations England & Wales, Northern Ireland, and Scotland), Canada, 2001-2019.

Participants: Full population aged 35-64.

Outcome Measures: We compared all-cause and "despair"-related mortality trends at mid-life across countries using publicly available mortality data, stratified by three age groups (35-44, 45-54 and 55-64) and by sex. We examined trends in all-cause mortality and mortality by causes categorized as 1) suicides 2) alcohol-specific deaths 3) drug-related deaths. We employ several descriptive approaches to visually inspect age, period, and cohort trends in these causes of death.

Results: The US and Scotland both saw large relative increases and high absolute levels of drug-related deaths. The rest of the UK and Canada saw relative increases but much lower absolute levels in comparison. Alcohol-specific deaths showed less consistent trends that did not track other "despair" causes, with older groups in Scotland seeing steep declines over time. Suicide deaths trended slowly upward in most countries.

Conclusions: In the UK, Scotland has suffered increases in drug-related mortality comparable to the US, while Canada and other UK constituent nations did not see dramatic increases. Alcohol-specific and suicide mortality generally follow different patterns to drug-related deaths across countries and over time, questioning the utility of a cohesive "deaths of despair" narrative.

For peer review only

Strengths and Limitations of this Study:

- We directly compare trends in "deaths of despair" in mid-life across the US, UK, and Canada.
- We leverage demographic approaches to understand period vs. cohort trends in these causes of death.
- Coding of deaths caused by alcohol, drug overdose, and suicide may vary across country and time, complicating comparisons.
- Disentangling age, period, and cohort effects in mortality trends is challenging.
- The study is descriptive and cannot speak to the causes of trends in drug overdose, suicide, and alcohol-specific deaths.

For peer review only

Introduction

The United States (US) has persistently lower life expectancy than its OECD peers, including 1.7-3.2 years below the United Kingdom (UK) and Canada in 2019 [1], two countries with a shared cultural, socio-political, and linguistic heritage. US life expectancy fell for three consecutive years starting in 2014, recovering slightly by 2019 prior to further declines in 2020 and 2021 during the COVID-19 pandemic [2, 3]. Explanations for stagnating life expectancy in the US and divergence from peer countries have highlighted increases in "deaths of despair" (i.e., deaths due to drug and alcohol poisoning, and suicide)[4, 5]. Life expectancy improvements have been stalling in England and Wales [6, 7] and Scotland [8] since the 2010's, raising the question of whether these US mortality dynamics reflect a broader phenomenon.

The US, the UK, and Canada have all documented rapid increases in drug-related mortality in recent years, with the US on the leading edge with an initial increase in deaths due to prescription opioids, followed by heroin and now fentanyl [9, 10]. Increases in opioid use in Canada may be related to exposure to US pharmaceutical promotional material through US media and direct marketing [11]. Within the UK, Scotland stands out with a dramatic rise in drug deaths (rising 450% since 1996) driven initially by prescription benzodiazepines and more recently by so-called 'street' benzodiazepines [12]. England and Wales have had a less extreme rise in drug-related deaths since 2012, driven by increasing numbers of opioid and cocaine-related deaths, and less noticeable increases due to benzodiazepines [13].

Besides changes in the supply and marketing of prescription and illicit drugs, increases in drug mortality may reflect demand side factors related to the loss of economic opportunity, particularly among socioeconomically disadvantaged groups [14, 15]. In Scotland, cohort trends in drug-related deaths and suicide were evident particularly in men from socioeconomically deprived areas coming of age in the 1980s [16, 17]. In the US and

For peer review only

the UK, lack of economic opportunity in middle-aged cohorts may contribute to poorer mental health and subsequent unhealthy coping behaviours [18, 19]. In Canada, socioeconomic inequalities in health measured by educational attainment and household income have increased over time, but less is known about whether this has translated into despair-related causes of death [20].

This paper compares recent trends in mid-life "deaths of despair" and its component causes in the US to those in the UK and Canada from 2000-2019. The UK was chosen as a comparison country also witnessing stalls in life expectancy. Thus far, whether UK patterns of mid-life mortality and despair-related are following the US is not known. Canada shares close economic and geographic ties to the US and has seen spill over effects of changing drug supply and the opioid epidemic [21].

Data

Publicly available all-cause and cause-specific mortality by age and sex were analysed for all countries. Because data are collected by separate statistical offices for the nations of the UK, and because of historically divergent mortality trends for Scotland, we estimate trends separately for UK nations, except for England and Wales whose data is released together. For England and Wales, we analyzed the 21st Century Mortality dataset from 2001-2019 [22], a publicly available resource that includes all registered deaths by cause as well as mid-year population counts by five-year age groups. Comparable data for Scotland is available from National Records of Scotland's Vital Events Reference Tables [23]. and for Northern Ireland from The Registrar General's Annual Reports [24]. For Canada, death counts by cause of death were accessed from publicly available Statistics Canada Table 13-10-0392-01 [25]. These records include all registered deaths in Canada each year from 2000-2019 by sex and 5-year age-group. US mortality data by year and single year of age come from CDC Wonder

For peer review only

[26]. Population counts by age and sex for all countries were obtained from the Human Mortality Database. Results are fully reproducible, and all metadata and code used in analysis can be accessed here.

Methods

We focus on mid-life mortality where increases have been documented in the U.S, specifically age groups 35-44, 45-54 and 55-64. We examine trends mortality by causes categorized as 1) suicides (excluding undetermined intent), 2) alcohol-specific deaths 3) drug-related deaths, and a combined 4) "deaths of despair" category summing all three causes, as well as all-cause mortality. (ICD-10 codes available in supplemental Table S1). In order to mitigate the impact of random variation in the cause-and age-specific mortality rates we used a 1-dimensional P-spline approach for each country, cause, sex and calendar year to smooth the observed death rates over age [27]. The resulting single year of age-specific mortality rates were then age-standardised within each of the three broad age groups using the European Standard Population [28]. We visually inspected cohort vs. period trends using plots of the 3-year rolling averages within age-groups over time, supplemented with Lexis surfaces.

Patient and Public Involvement

None

Results

Full results for all age group, sex, country and cause groupings for all calendar years are shown in Appendix Tables S2a – S6b, with summary data for 2001, 2010 and 2019 presented in Tables 1a-b and 2a-b. All-cause and cause-specific mortality trends for the combined age range of 35-64 years of age are shown in Supplemental figures S1 and S2a-c.

For peer review only

Table 1a: Male mortality rates (per 100,000 population) by cause and age group for England & Wales, Northern Ireland and Scotland

Age group	England & Wales			Northern Ireland			Scotland		
	2001	2010	2019	2001	2010	2019	2001	2010	2019
All-Cause									
35-44	190.7	168.9	162.8	192.7	207.7	190.4	274.9	244.0	284.2
45-54	461.4	393.9	371.0	465.7	443.7	378.1	649.0	520.1	529.8
55-64	1280.1	991.9	888.6	1324.3	1092.9	876.9	1708.3	1257.4	1076.2
Combined 'Deaths of Despair'									
35-44	44.8	48.0	58.0	47.6	67.9	81.9	82.2	99.5	157.5
45-54	50.5	58.0	71.1	54.9	82.6	85.0	114.9	109.1	139.2
55-64	46.6	56.0	66.1	50.0	79.6	74.5	127.9	108.3	98.0
Alcohol									
35-44	19.8	20.3	16.3	26.7	26.3	29.0	40.9	35.0	24.7
45-54	32.7	37.4	35.3	41.2	50.8	55.5	86.1	70.8	48.5
55-64	33.1	41.4	43.3	38.1	56.8	58.8	107.3	87.0	60.4
Drugs									
35-44	9.8	12.8	22.8	1.6	10.6	23.6	15.1	36.0	102.4
45-54	4.6	6.1	16.6	1.8	6.3	6.2	5.5	16.6	63.6
55-64	2.3	2.5	6.5	2.0	4.1	0.8	2.3	6.6	15.6
Suicide									
35-44	15.2	14.9	18.9	19.4	31.0	29.3	26.2	28.5	30.4
45-54	13.2	14.5	19.1	11.9	25.5	23.4	23.2	21.7	27.1
55-64	11.1	12.1	16.3	9.9	18.7	15.0	18.3	14.7	22.0

For peer review only

Table 1b: Male mortality rates (per 100,000 population) by cause and age group for Canada and the USA

Age group	Canada 2001	Canada 2010	Canada 2019	USA 2001	USA 2010	USA 2019
All-Cause						
35-44	183.6	164.1	161.7	259.2	212.2	257.3
45-54	424.3	361.3	334.6	551.3	508.9	484.9
55-64	1155.9	916.9	826.3	1226.1	1082.7	1106.7
Combined 'Deaths of Despair'						
35-44	44.4	46.3	50.1	58.2	61.0	101.6
45-54	53.7	58.2	62.6	77.2	94.1	115.5
55-64	67.9	68.3	75.5	68.7	93.0	134.8
Alcohol						
35-44	8.2	8.5	8.4	14.9	12.4	16.9
45-54	22.7	22.1	21.0	36.9	36.8	37.1
55-64	42.5	38.5	40.2	42.9	50.3	63.2
Drugs						
35-44	9.7	13.4	21.1	20.1	23.6	56.6
45-54	6.1	11.7	19.6	16.7	26.7	49.2
55-64	3.2	7.1	13.1	4.6	15.1	41.1
Suicide						
35-44	26.6	24.4	20.6	23.3	25.1	28.1
45-54	24.9	24.4	22.0	23.7	30.6	29.3
55-64	22.3	22.7	22.1	21.2	27.6	30.5

For peer review only

Table 2a: Female mortality rates (per 100,000 population) by cause and age group for England & Wales, Northern Ireland and Scotland

Age group	England & Wales 2001	England & Wales 2010	England & Wales 2019	Northern Ireland 2001	Northern Ireland 2010	Northern Ireland 2019	Scotland 2001	Scotland 2010	Scotland 2019
All-Cause									
35-44	117.5	104.8	98.7	119.4	122.3	115.0	149.2	133.4	153.4
45-54	302.5	260.4	243.0	310.2	292.7	274.0	387.9	328.6	341.1
55-64	802.5	641.2	587.8	809.9	704.9	639.7	1014.3	835.8	759.9
Combined 'Deaths of Despair'									
35-44	16.8	18.3	23.2	20.1	26.3	32.3	35.6	37.4	61.7
45-54	23.0	24.6	30.5	29.4	39.6	43.3	48.4	46.4	64.7
55-64	23.7	25.4	29.4	24.6	36.3	42.5	49.9	44.9	53.3
Alcohol									
35-44	9.6	10.2	9.7	15.0	13.9	17.1	19.9	17.9	12.8
45-54	16.4	17.3	18.2	22.9	25.9	29.9	36.7	32.2	28.4
55-64	17.7	19.6	20.5	20.8	26.1	32.7	41.3	35.6	37.5
Drugs									
35-44	3.5	4.3	8.4	1.2	4.4	8.2	4.8	10.5	40.2
45-54	2.5	3.3	7.1	2.4	4.2	6.4	2.8	7.5	26.1
55-64	1.9	2.1	4.2	0.2	3.1	4.1	2.0	4.7	9.0
Suicide									
35-44	3.8	3.8	5.2	3.9	7.9	7.0	10.9	8.9	8.7
45-54	4.2	4.0	5.3	4.1	9.5	7.0	9.0	6.7	10.2
55-64	4.1	3.7	4.7	3.6	7.1	5.8	6.6	4.6	6.8

For peer review only

Table 2b: Female mortality rates (per 100,000 population) by cause and age group for Canada and the USA

	Canada			USA		
Age group	2001	2010	2019	2001	2010	2019
All-Cause						
35-44	112.1	100.2	94.6	147.6	128.1	142.8
45-54	268.6	235.9	212.6	319.8	311.7	295.1
55-64	685.9	586.7	529.9	773.4	647.8	667.8
Combined 'Deaths of Despair'						
35-44	15.9	19.1	19.0	22.0	27.7	40.5
45-54	20.0	24.3	24.1	26.3	42.8	51.2
55-64	24.1	26.7	28.9	24.1	35.1	53.8
Alcohol						
35-44	4.0	4.2	4.3	6.4	5.5	8.6
45-54	8.6	8.5	9.5	12.1	14.6	17.7
55-64	14.7	13.2	16.4	15.8	17.3	26.7
Drugs						
35-44	4.1	7.2	9.0	9.2	14.7	23.6
45-54	3.4	7.3	8.2	7.0	19.0	23.2
55-64	2.5	5.7	5.8	2.6	9.9	18.2
Suicide						
35-44	7.8	7.6	5.7	6.5	7.4	8.2
45-54	8.0	8.4	6.5	7.2	9.2	10.3
55-64	6.9	7.8	6.7	5.7	7.9	8.9

For peer review only

Figure 1 shows all-cause mortality rates from 2001-2019 by age and sex group. Observed mortality patterns differ across nations and by sex. In Canada and England & Wales, mortality rates are low and decline consistently for most age-sex combinations, except for 35–44-year-olds for which mortality trends are flat or increasing. Upticks in mortality were more noticeable in Scotland and the US in 35–44-year-olds, with steeper increases for males compared to females. In the US, all-cause mortality among males 35-44 reached a low point of 211.8/100,000 in 2012 before rising steadily to 257.3/100,000 in 2019 (Supplemental Table S6a). Males aged 35-44 in Scotland followed a similar trend, with slightly higher absolute levels of mortality compared to US males. This youngest age group also showed signs of stalling improvements or slight increases in mortality in Canada, England & Wales, and Northern Ireland. Noticeable increases in overall mortality were also seen in Scotland for males and females ages 45-64, but not in the 55–64-year-old age group. The US was notable for its stagnation or increases in mortality across almost all age-sex groups.

Figure 2 shows trends in despair-related deaths over the same period. The US and Scotland again stand out for substantially higher and largely increasing rates across the period. Males aged 35-44 show some of the worst trends among analyzed countries, with rates in the US and Scotland increasing almost 2-fold over the period. While the absolute increases for males in England and Wales and Northern Ireland are much smaller, they reflect large relative increases (for example a 41% increase in despair-related deaths for males ages 45-54 over this period in England and Wales). Canada is notable for its low levels and relatively small increases in deaths of despair, especially for women for whom rates hardly increase. While females in most nations saw only small increases in deaths of despair, the US stood out with a trend of high and rising mortality for both males and females in all age groups.

For peer review only

Figures 2a-c break down these “despair” deaths into individual causes, where we see the salience of increasing drug-related mortality in the US and Scotland. The magnitude of the increases for males ages 35-44 in both countries was staggering (an almost 6-fold increase in Scotland from 15.1/100,000 to 102.3/100,000). In the US, these increases are seen across all age groups, while in Scotland the increases are significantly less pronounced for 55–64-year-olds. Although at lower absolute levels, drug-related mortality also increased in Canada, England and Wales and Northern Ireland over the period, especially among younger males (for example an increase of 118% from 9.1/100,000 to 21.1/100,000 for Canadian males aged 35-44).

Suicide mortality saw more year-to-year variability, with evidence of small increases among males in England & Wales, Scotland, and the US. The magnitude of these changes is very small compared to changes in drug-related deaths. Suicide rates were higher for males in all nations. Canada consistently had the lowest levels of suicide mortality across all age-sex groups. The US generally had the highest levels of suicide deaths, except for males ages 35-44 where Scotland and Northern Ireland had higher levels. Alcohol-specific mortality across most nations was relatively stable, but with sharp declines over time in Scotland from previously very high levels, especially at the older ages. In contrast, increases in alcohol-specific mortality in the US were seen in ages 55-64, especially for men. Taken together, these results suggest that drug-related mortality is the primary cause of increases in overall “despair”-related mortality over this period, with small but perceptible increases in England & Wales, Canada, and Northern Ireland, and very pronounced increases in the US and Scotland.

Figures S3a-c show an alternative view of the same data to examine period vs. cohort trends. Here we look for “non-parallelism” in the age-specific mortality curves over time within each country [29]. If the age-specific curves are parallel to each other, this likely reflects the

For peer review only - http://bmjopen.bmj.com/site/about/guidelines.xhtml

salience of period influences. Non-parallel age-specific trends suggest more importance for cohort effects (For a detailed discussion and simulations of these dynamics, see [30], pages 204-217). For overall deaths of despair, age-specific trends over time are mostly parallel for Canada, England & Wales, and Northern Ireland, suggesting period-based dynamics affecting all cohorts. The US and Scotland show some deviations from parallelism, with the youngest age group showing sharper increases than the older groups in Scotland, and the 55–64-year-olds in the US increasing more steeply (and earlier) than younger groups. For drug-related deaths, parallel trends are largely suggestive of period-based effects for Canada, England & Wales, and the US. Non-parallelism in Northern Ireland & Scotland suggests younger born cohorts have been hit harder by recent increases in drug deaths than the oldest cohort. For suicide, most trends are parallel, suggesting period fluctuations, except for the US where the oldest age group has increased more steeply. Alcohol-specific mortality mostly follows period-based parallel trends, except for the US where again the oldest age group has increased over time. Cohort-driven declines in alcohol-related mortality were seen in Scotland, with the older groups declining steeply from high levels compared to lower and flatter trends in the youngest age group. Supplemental Figures S4a-b show alternative visualizations for cohort patterns in mortality using Lexis surfaces. Overall, these visualizations highlight some limited cohort trends but also very strong period effects of increased drug deaths that affected most age groups and nations. In the US, increases in drug-related deaths affected all age groups over time, but sharper increases in alcohol-specific deaths and suicide were only seen in the oldest age group (earliest born cohort). This oldest group largely reflects the Baby Boom cohort (born 1946-1964) passing this 55-64 age group over the observed period.

For peer review only

Supplemental Figures S5a-c show trends in the proportion all deaths due to the individual despair related causes over time, with drug-related mortality standing out as an increasing proportion of all-cause mortality. From Table 1a, we can see that the all-cause mortality rate increased by 45/100,000 for 35–44-year-old US males from 2010 to 2019. Of this increase, 73% could be accounted for by an increase in drug-related mortality, and 90% was accounted for by increases in all three despair-related causes. For 55–64-year-old males, all-cause mortality increased by 23.9/100,000 over the same period, which was more than fully accounted for by an increase in drug-related mortality of 26/100,000 from 2010 to 2019. Thus, in many age-sex groups all-cause mortality would have declined or plateaued rather than increased in the US if drug mortality had stayed constant.

Discussion

We describe large relative increases and high absolute levels of drug-related deaths in both the US and Scotland from 2001-2019, with lower levels and smaller increases in Canada and the rest of the UK. Alcohol-specific deaths showed less consistent trends that did not track other "despair" causes, with older groups in Scotland seeing steep declines over time. Suicide deaths trended slowly upward in most countries. We generally found more evidence for period rather than cohort effects for these trends, apart from baby boomers in the US, consistent with previous evidence of their cohort mortality disadvantage [31].

Increases in mid-life mortality in the last two decades in the US set off a robust debate about the underlying causes of this concerning trend, with a strong focus on "deaths of despair." One narrative emphasizes the role of cumulative disadvantage triggered by worsening labour market opportunities for white Americans with low levels of education, leading to psychological

For peer review only

distress and unhealthy behavioural coping mechanisms [5, 32, 33]. A second hypothesis emphasizes the strong period trend in drug-related mortality due to changes in the availability of prescription and illicit opioids, including heroin, and synthetic opioids like fentanyl)[34, 35]. While the first hypothesis implies that "despair" deaths share a fundamental cause and typically move together, the latter argues that the trends in suicide and alcohol-specific mortality are distinct and do not fit well within the overall deaths of despair narrative [36, 37].

Our study extends this work by comparing mid-life mortality trends in the US to Canada and the UK, two countries with shared sociocultural histories with the US but different economic, political and health care contexts. The UK has also experienced recent slowdowns in mortality improvements, raising the question of whether the US is the leading edge of more general mortality trends. Overall, we find evidence that the levelling off and increases in mid-life mortality seen in the US may be emerging more broadly, but the magnitude of these changes in other countries is muted. Scotland is the exception, with upward trends in drug-related deaths surpassing the high levels in the US and contributing to increases in overall mortality trends for males ages 35-54. While drug-related deaths have increased on a relative scale in the other UK nations and Canada, deaths from these causes are at much lower absolute levels than the US and Scotland. There is little evidence of significant trends in suicide or alcohol-specific deaths contributing to changes in all-cause mortality.

Our results suggest that increases in drug-related mortality are not necessarily unique to the US, with smaller increases also seen in Canada, England & Wales, and Northern Ireland, particularly among younger males. Scotland stands out as trending even higher than the US in drug-related mortality at younger ages, while the US saw parallel increases in drug-mortality across all age-ranges, suggesting a strong period effect. We found some evidence of cohort

For peer review only

effects in alcohol-specific and suicide mortality in the US, with the oldest cohort trending up relative to younger cohorts. Suicide mortality rates were generally stable across countries for women, with slight increases for men and women in the US. Overall, with the exception of the oldest born (i.e. baby boom) male cohort in the US, we find that individual "deaths of despair" do not follow similar age and cohort patterns and thus may not reflect the same fundamental causes.

The fact that drug-related deaths have increased in other countries but not to the degree of the US is consistent with differences in prescribing practices and health care contexts. In the UK, there is no direct-to-consumer marketing by pharmaceutical companies, and the primary care system likely mitigates patients receiving prescriptions from multiple sources [38]. Nonetheless, prescribing for non-cancer pain did increase dramatically since the late 1990s when consensus statements supporting the use of opioids for the treatment of chronic pain [39, 40]. Despite this increase in overall opioid prescribing, a review of the UK Clinical Practice Research Datalink (CRPD) did not find an increase in the rate of diagnosed opioid use disorders in the UK between 2008 and 2012 [41]. While deaths from fentanyl in the UK have grown, they are still small relative to deaths from heroin or morphine, suggesting the context for drug prescribing and misuse may be different in the UK [42]. The dramatic rise in drug deaths in Scotland, with rates far outstripping those elsewhere in Europe[1] has arisen through a somewhat different mechanism to the US' initial prescription opioid-driven crises. A combination of economic, social and political factors experienced by those living in deprived areas in the 1980s led to a rise in drug-related death rates in the 1990s [16, 17], driven by opioids such as heroin, often taken in combination with prescription benzodiazepines such as diazepam [43]. Although steps were

[1] https://www.nrscotland.gov.uk/files//statistics/drug-related-deaths/20/drug-related-deaths-20-pub.pdf

taken to reduce the prescribing of benzodiazepines in the late 2000s, these were swiftly replaced by a range of ‘synethic benzodiazepines’ or ‘street benzos’ such as etizolam, which became widely and cheaply available through illicit markets [12]. The explosion in the use of street benzos has played a huge role in the rapid rise in Scottish drug deaths since 2012 when street benzos were implicated in 3% of all drug deaths compared to 66% in 2020, with concurrent use with opioids in particular the key factor – less than 1% of drug deaths in 2020 involved street benzos alone[2]. In the UK overall, , austerity measures after the 2008 financial crisis have been suggested as one cause of stalling life expectancy [44, 45]. While many have focused on cuts in health and social care affecting older age mortality [46], some evidence has linked austerity to an increase in drug deaths affecting younger and middle ages [47, 48]. While concern over the opioid epidemic and fentanyl in Canada is rising [49], the absence of overall increases in mortality thus far has minimized discussion of a similar epidemic of “deaths of despair.”

Strengths and Limitations

Our study has several strengths and limitations. We use a demographic perspective to compare period and cohort trends in “despair” related deaths in the US, UK, and Canada. Disentangling age, period, and cohort effects in mortality is a persistent challenge, and we take a transparent visualization approach to ease interpretation. When examining external causes of death such as those due to alcohol, drug overdose, and suicide, there are differences in coding practices across country and time that could impact results. The overlapping nature of these causes also means there will be limitations to any set of definitions. We largely follow the original “despair” categorizations of Case and Deaton [5] and have previously demonstrated that the use of alternative categorizations is unlikely to substantively change our conclusions [50]. Our suicide

[2] https://www.nrscotland.gov.uk/statistics-and-data/statistics/statistics-by-theme/vital-events/deaths/drug-related-deaths-in-scotland/2020

For peer review only

category excludes deaths from undetermined intent, which differs from some studies [19] and should be considered when doing international comparisons. Additionally, our study is descriptive and cannot speak to the causes of trends in drug overdose, suicide, and alcohol-specific deaths or related policy solutions. Finally, we examine trends only through 2019 and do not consider the specific dynamics of trends in despair-related mortality during the COVID-19 pandemic. Recent work showed drug deaths rising considerably in the US during the pandemic across all ages, which was not seen in England or Scotland [50]. Concerns about rising suicide rates during the pandemic seem unfounded, but alcohol-related deaths increased in both the US and UK [50]. The impact of the pandemic as a period and cohort shock to risk behaviours and causes of deaths will be an important area of future research.

Conclusions

"Deaths of despair" due to alcohol, drug overdoses, and suicide have been implicated in rising mid-life mortality in the US, but how these trends compare to peer countries is less well-known. We find that the US is unique in its large relative and absolute increases in drug-related mortality for all mid-life age-sex-groups, but that Scotland has seen a dramatic upward trend in drug deaths that now exceed the US for younger age groups. The huge increase in drug mortality across all ages in the US from 2001-2019 is most consistent with period explanations, while alcohol and suicide upward trends in the US are more focused in the oldest cohort. Overall, the trends in individual causes of despair deaths are not consistent across countries or age-groups, questioning the value of a cohesive "deaths of despair" narrative. This is consistent with other work finding different spatiotemporal patterns of these causes within the US [51]. Future

For peer review only

research should consider the determinants of these specific causes across place and time without assuming they represent an underlying phenomenon with the same underlying drivers [52].

Ethics Approval Statement

The study does not involve human subjects, as it uses publicly available death counts from national vital statistics and no individual-level data.

Contributorship statement

The authors confirm contribution to the paper as follows: study conception and design: JBD; data collection CA, JBD, AZ; analysis and interpretation of results: CA, JBD; draft manuscript preparation: JBD. All authors critically reviewed the results and approved the final version of the manuscript.

Competing interests

The authors have no competing interests to declare.

Data sharing statement

Data are available in a public, open access repository. Results are fully reproducible, and all data and code can be accessed at: https://github.com/VictimOfMaths/DeathsOfDespair/tree/master/Paper

Funding Acknowledgement

The authors acknowledge funding support from the European Research Council (ERC-2021-CoG-101002587) and The Leverhulme Trust (Large Centre Grant). The funders played no direct role on this research.

For peer review only

References

1. Aburto JM, Schöley J, Kashnitsky I, Zhang L, Rahal C, Missov TI, et al. Quantifying impacts of the COVID-19 pandemic through life-expectancy losses: a population-level study of 29 countries. International Journal of Epidemiology. 2021. doi: 10.1093/ije/dyab207.

2. Murphy SL, Xu J, Kochanek KD, Arias E. Mortality in the united states, 2017. 2018.

3. Schöley J, Aburto JM, Kashnitsky I, Kniffka MS, Zhang L, Jaadla H, et al. Bounce backs amid continued losses: Life expectancy changes since COVID-19. medRxiv. 2022:2022.02.23.22271380. doi: 10.1101/2022.02.23.22271380.

4. Tilstra AM, Simon DH, Masters RK. Trends in "Deaths of Despair" among Working Aged White and Black Americans, 1990-2017. American journal of epidemiology. 2021.

5. Case A, Deaton A. Mortality and morbidity in the 21st century. Brookings Papers on Economic Activity. 2017.

6. Hiam L, Dorling D, Harrison D, McKee M. Why has mortality in England and Wales been increasing? An iterative demographic analysis. Journal of the Royal Society of Medicine. 2017;110(4):153-62.

7. Murphy ML, Marc; Torrisi, Orsola. Stalling of mortality in the United Kingdom and Europe: an analytical review of the evidence. LSE Department of Social Policy Working Paper Series2019.

8. Minton J, Fletcher E, Ramsay J, Little K, McCartney G. How bad are life expectancy trends across the UK, and what would it take to get back to previous trends? J Epidemiol Community Health. 2020;74(9):741-6.

9. Ciccarone D. The triple wave epidemic: Supply and demand drivers of the US opioid overdose crisis. International Journal of Drug Policy. 2019;71:183-8. doi: https://doi.org/10.1016/j.drugpo.2019.01.010.

10. John E, Butt A, McQuade G. Deaths related to drug poisoning in England and Wales: 2018 registrations. Office for National Statistics, Aug; 2019.

11. Lexchin J, Kohler JC. The danger of imperfect regulation: OxyContin use in the United States and Canada. International Journal of Risk & Safety in Medicine. 2011;23(4):233-40.

For peer review only

12. McAuley A, Matheson C, Robertson JR. From the clinic to the street: the changing role of benzodiazepines in the Scottish overdose epidemic. International Journal of Drug Policy. 2022;100:103512. doi: https://doi.org/10.1016/j.drugpo.2021.103512.

13. Breen P, Manders B. Deaths related to drug poisoning in England and Wales: 2020 registrations. Off Natl Stat. 2021.

14. O'Brien R, Bair EF, Venkataramani AS. Death by Robots? Automation and Working-Age Mortality in the United States. Demography. 2022;59(2):607-28. doi: 10.1215/00703370-9774819.

15. Ruhm CJ. Drivers of the fatal drug epidemic. Journal of health economics. 2019;64:25-42.

16. Parkinson J, Minton J, Lewsey J, Bouttell J, McCartney G. Drug-related deaths in Scotland 1979–2013: evidence of a vulnerable cohort of young men living in deprived areas. BMC Public Health. 2018;18(1):357. doi: 10.1186/s12889-018-5267-2.

17. Parkinson J, Minton J, Lewsey J, Bouttell J, McCartney G. Recent cohort effects in suicide in Scotland: a legacy of the 1980s? Journal of Epidemiology and Community Health. 2017;71(2):194. doi: 10.1136/jech-2016-207296.

18. Marmot M. Social causes of the slowdown in health improvement. BMJ Publishing Group Ltd; 2018.

19. Walsh D, McCartney G, Minton J, Parkinson J, Shipton D, Whyte B. Deaths from 'diseases of despair' in Britain: comparing suicide, alcohol-related and drug-related mortality for birth cohorts in Scotland, England and Wales, and selected cities. Journal of Epidemiology and Community Health. 2021;75(12):1195. doi: 10.1136/jech-2020-216220.

20. Hajizadeh M, Mitnitski A, Rockwood K. Socioeconomic gradient in health in Canada: Is the gap widening or narrowing? Health Policy. 2016;120(9):1040-50.

21. Jannetto PJ. The north American opioid epidemic. Therapeutic Drug Monitoring. 2021;43(1):1-5.

22. [dataset] Deaths registered in England and Wales – 21st century mortality. Office for National Statistics; 2022. Doi: https://www.ons.gov.uk/peoplepopulationandcommunity/birthsdeathsandmarriages/deaths/datasets/the21stcenturymortalityfilesdeathsdataset

23. [dataset] Archive- Historical data back to 2001. National Records of Scotland. doi: https://www.nrscotland.gov.uk/statistics-and-data/statistics/statistics-by-theme/vital-events/general-publications/vital-events-reference-tables/archive

24. [dataset] Registrar General Annual Reports 2011-2019. Northern Ireland Statistics and Research Agency; 2021. doi: https://www.nisra.gov.uk/publications/registrar-general-annual-reports-2011-2019

For peer review only

25. [dataset] Statistics Canada. Deaths and Age-Specific Mortality rates. Table 13-10-0392-01 January 1, 2022. doi: https://www150.statcan.gc.ca/t1/tbl1/en/tv.action?pid=1310039201

26. [dataset] CDC Wonder. US Centers for Disease Control and Prevention. doi: https://wonder.cdc.gov/

27. Camarda CG. MortalitySmooth: An R package for smoothing Poisson counts with P-splines. Journal of Statistical Software. 2012;50:1-24.

28. Pace M, Lanzieri G, Glickman M, Zupanič T. Revision of the European Standard Population: report of Eurostat's task force: Publications Office of the European Union; 2013.

29. Kupper LL, Janis JM, Karmous A, Greenberg BG. Statistical age-period-cohort analysis: a review and critique. Journal of chronic diseases. 1985;38(10):811-30.

30. National Academies of Sciences E, Medicine. High and Rising Mortality Rates Among Working-Age Adults. 2021.

31. Acosta E, Gagnon A, Ouellette N, Bourbeau RR, Nepomuceno MR, van Raalte AA. The boomer penalty: excess mortality among baby boomers in Canada and the United States. Max Planck Institute for Demographic Research, Rostock, Germany, 2020.

32. Case A, Deaton A. Deaths of Despair and the Future of Capitalism. Deaths of Despair and the Future of Capitalism: Princeton University Press; 2020.

33. Zajacova A, Grol-Prokopczyk H, Zimmer Z. Pain trends among american adults, 2002–2018: Patterns, disparities, and correlates. Demography. 2021;58(2):711-38.

34. Masters RK, Tilstra AM, Simon DH. Mortality from suicide, chronic liver disease, and drug poisonings among middle-aged US white men and women, 1980–2013. Biodemography and social biology. 2017;63(1):31-7.

35. Alpert A, Evans WN, Lieber EMJ, Powell D. Origins of the Opioid Crisis and its Enduring Impacts*. The Quarterly Journal of Economics. 2022;137(2):1139-79. doi: 10.1093/qje/qjab043.

36. Masters RK, Tilstra AM, Simon DH. Explaining recent mortality trends among younger and middle-aged White Americans. International journal of epidemiology. 2017;47(1):81-8.

37. Ruhm CJ. Deaths of despair or drug problems? National Bureau of Economic Research, 2018.

38. Stannard C. Opioid prescribing in the UK: can we avert a public health disaster? British journal of pain. 2012;6(1):7.

39. Max MB, Donovan M, Miaskowski CA, Ward SE, Gordon D, Bookbinder M, et al. Quality improvement guidelines for the treatment of acute pain and cancer pain. Jama. 1995;274(23):1874-80.

For peer review only

40. Zin CS, Chen LC, Knaggs RD. Changes in trends and pattern of strong opioid prescribing in primary care. European journal of pain. 2014;18(9):1343-51.

41. Cooper AJ, Willis J, Fuller J, Benecke H, Leighton-Scott J, Andersohn F, et al. Prevalence and incidence trends for diagnosed prescription opioid use disorders in the United Kingdom. Pain and therapy. 2017;6(1):73-84.

42. Torjesen I. Fentanyl misuse in the UK: will we see a surge in deaths? bmj. 2018;361:k1564.

43. Jones JD, Mogali S, Comer SD. Polydrug abuse: a review of opioid and benzodiazepine combination use. Drug and alcohol dependence. 2012;125(1-2):8-18.

44. McCartney G, McMaster R, Popham F, Dundas R, Walsh D. Is austerity a cause of slower improvements in mortality in high-income countries? A panel analysis. Social Science & Medicine. 2022;313:115397. doi: https://doi.org/10.1016/j.socscimed.2022.115397.

45. Dorling D. Austerity bites: falling life expectancy in the UK. BMJ. 2019.

46. Hiam L, Harrison D, McKee M, Dorling D. Why is life expectancy in England and Wales 'stalling'? J Epidemiol Community Health. 2018;72(5):404-8.

47. Friebel R, Yoo KJ, Maynou L. Opioid abuse and austerity: evidence on health service use and mortality in England. Social Science & Medicine. 2022;298:114511.

48. Koltai, J., McKee, M., & Stuckler, D. (2021). Association between disability-related budget reductions and increasing drug-related mortality across local authorities in Great Britain. *Social Science & Medicine, 284*, 114225. doi:https://doi.org/10.1016/j.socscimed.2021.114225

49. Fischer B, Vojtila L, Rehm J. The 'fentanyl epidemic'in Canada–some cautionary observations focusing on opioid-related mortality. Preventive medicine. 2018;107:109-13.

50. Angus C, Buckley C, Tilstra AM, Dowd JB. Increases in 'deaths of despair' during the COVID-19 pandemic in the United States and the United Kingdom. Public Health. 2023;218:92-6. doi: https://doi.org/10.1016/j.puhe.2023.02.019.

51. Simon DH, Masters RK. Do deaths of despair move together? County-level mortality changes by sex and urbanization, 1990–2017. American Journal of Epidemiology. 2021;190(6):1169-71.

52. Tilstra AM. Stop Analyzing Suicides, Drug-and Alcohol-Related Mortality Together: Response to "Are We Undercounting the True Burden of Mortality". American journal of epidemiology. 2023.

For peer review only

Figure Legends

Figure 1: All-cause mortality by age and sex in Canada, the UK and the USA, 2001-2019

Figure 2: Combined 'deaths of despair' mortality (drug, suicide, and alcohol) by age and sex in Canada, the UK and the USA, 2001-2019

Figure 2a – Drug-related mortality by age and sex in Canada, the UK and the USA, 2001-2019

Figure 2b - Suicide mortality by age and sex in Canada, the UK and the USA, 2001-2019

Figure 2c – Alcohol-specific mortality by age and sex in Canada, the UK and the USA, 2001-2019

For peer review only

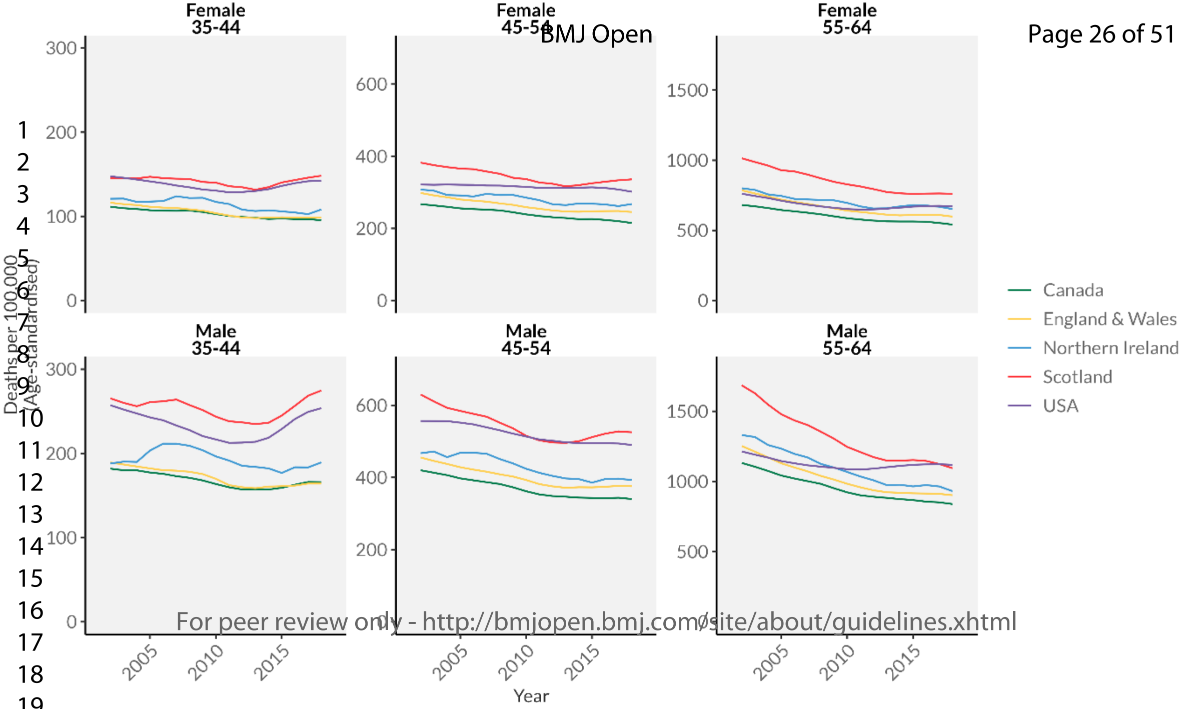

For peer review only - http://bmjopen.bmj.com/site/about/guidelines.xhtml

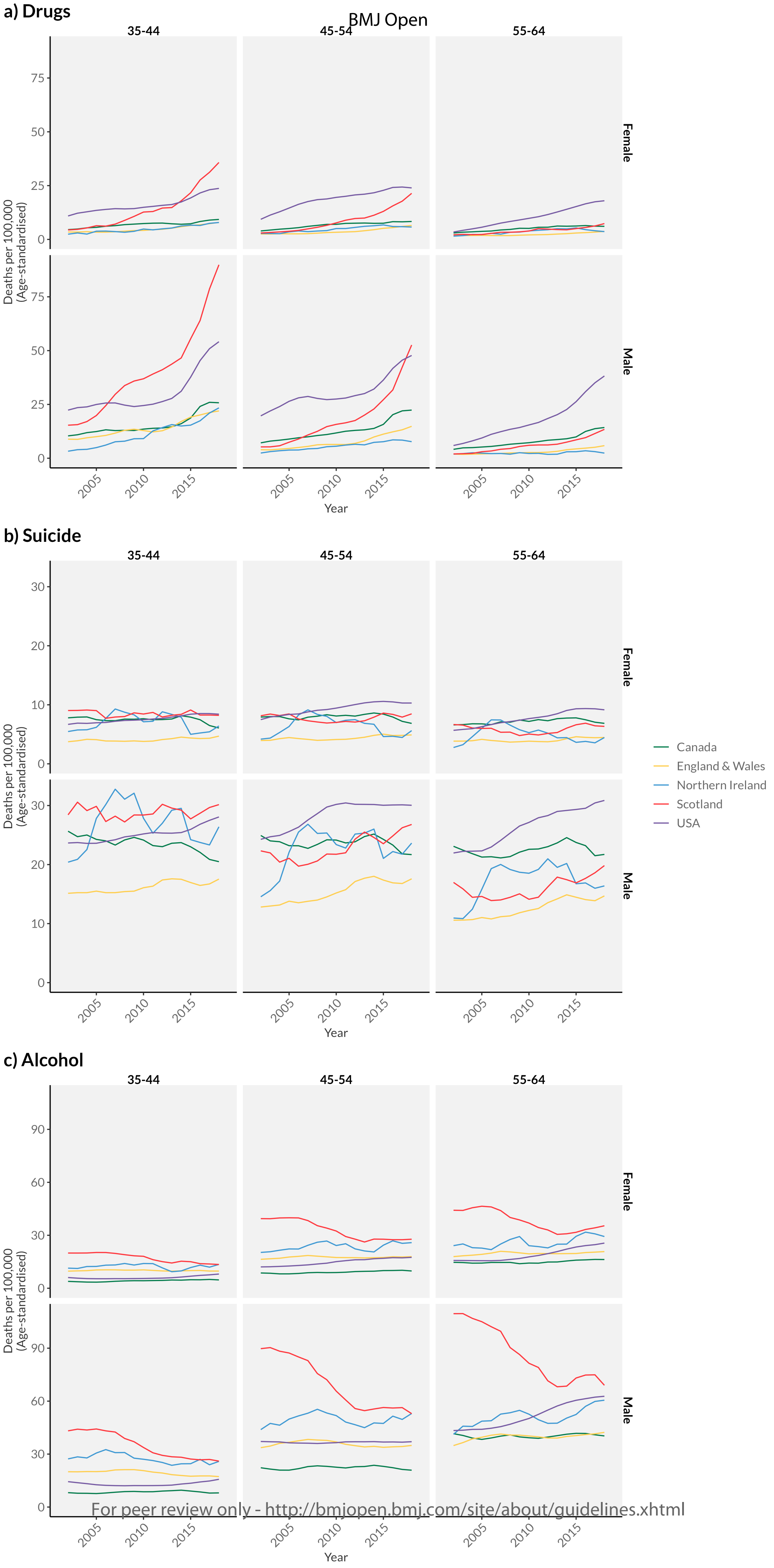
a) Drugs
b) Suicide
c) Alcohol
35-44
45-54
55-64
Female
Male
Deaths per 100,000
(Age-standardised)
Year
2005
2010
2015
Canada
England & Wales
Northern Ireland
Scotland
USA

Supplemental Figures and Tables: Comparing trends in Mid-life "Deaths of Despair" in the US, Canada, and UK, 2001-2019: Is the US an Anomaly? Dowd, et al 2023.

Figure S1 - All-cause mortality in ages 35-64 by sex in Canada, the UK and the USA

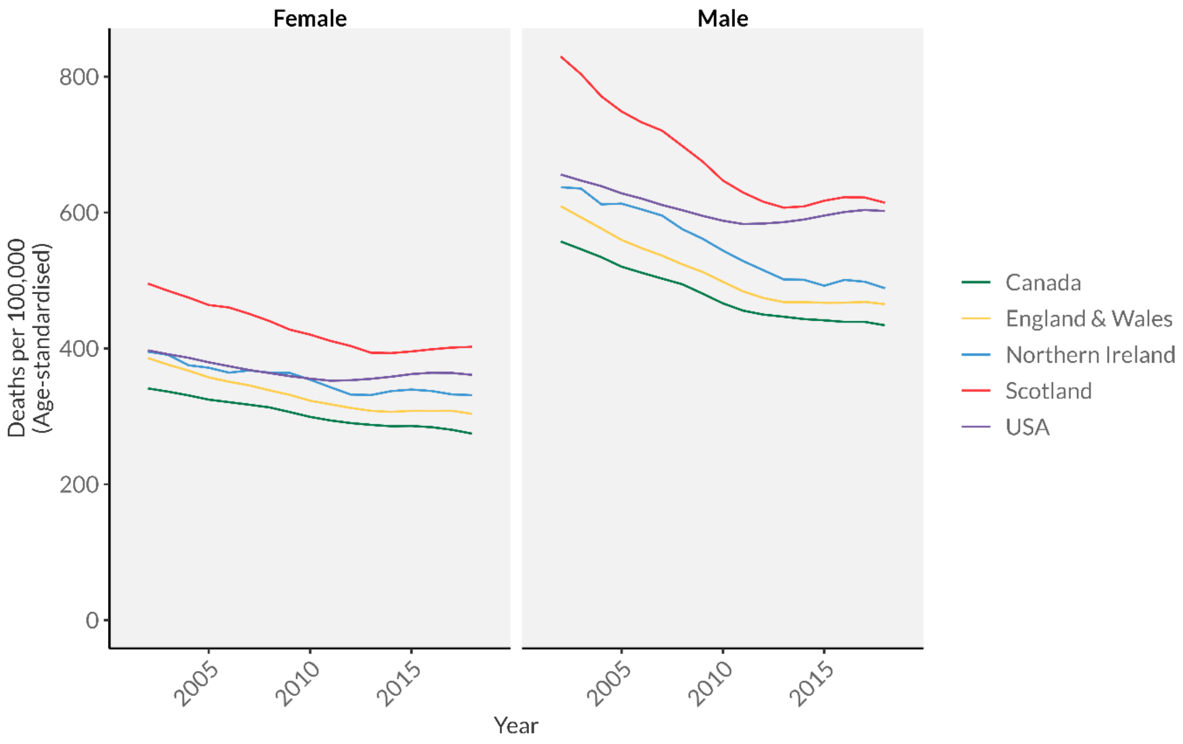

Figure S2 – Combined 'deaths of despair' mortality in ages 35-64 by sex in Canada, the UK and the USA

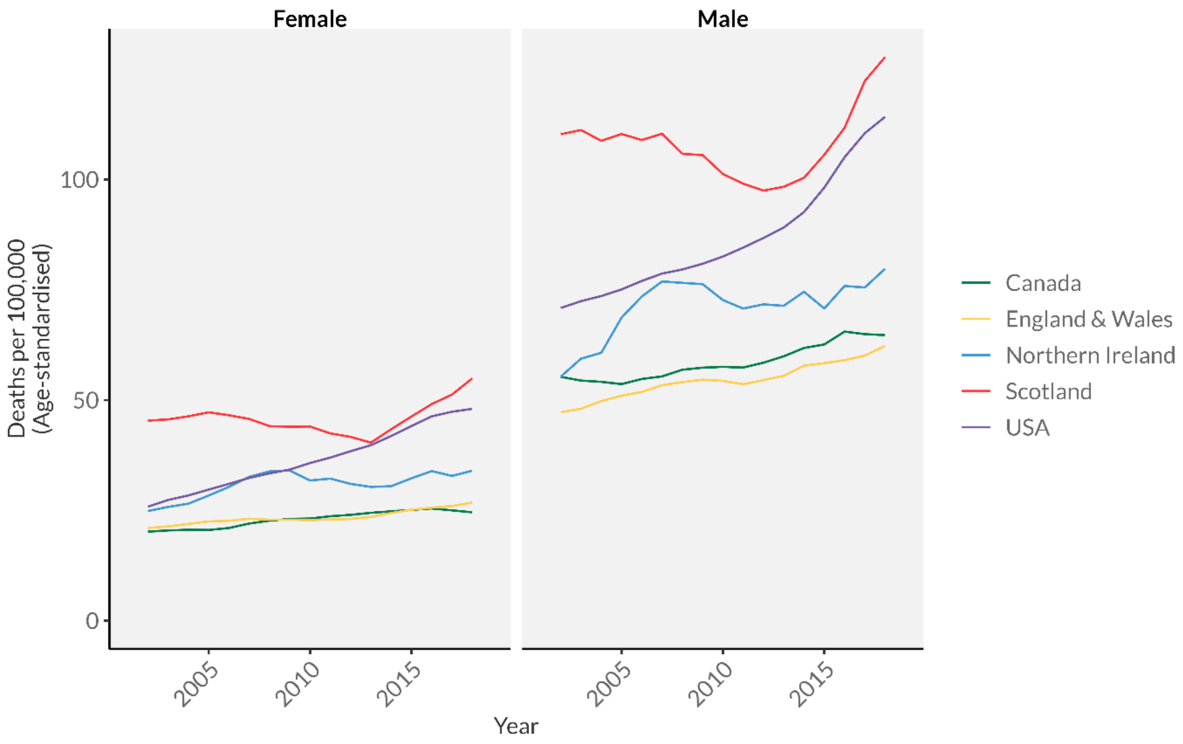

Figure S2a - Drug-related mortality in ages 35-64 by sex in Canada, the UK and the USA

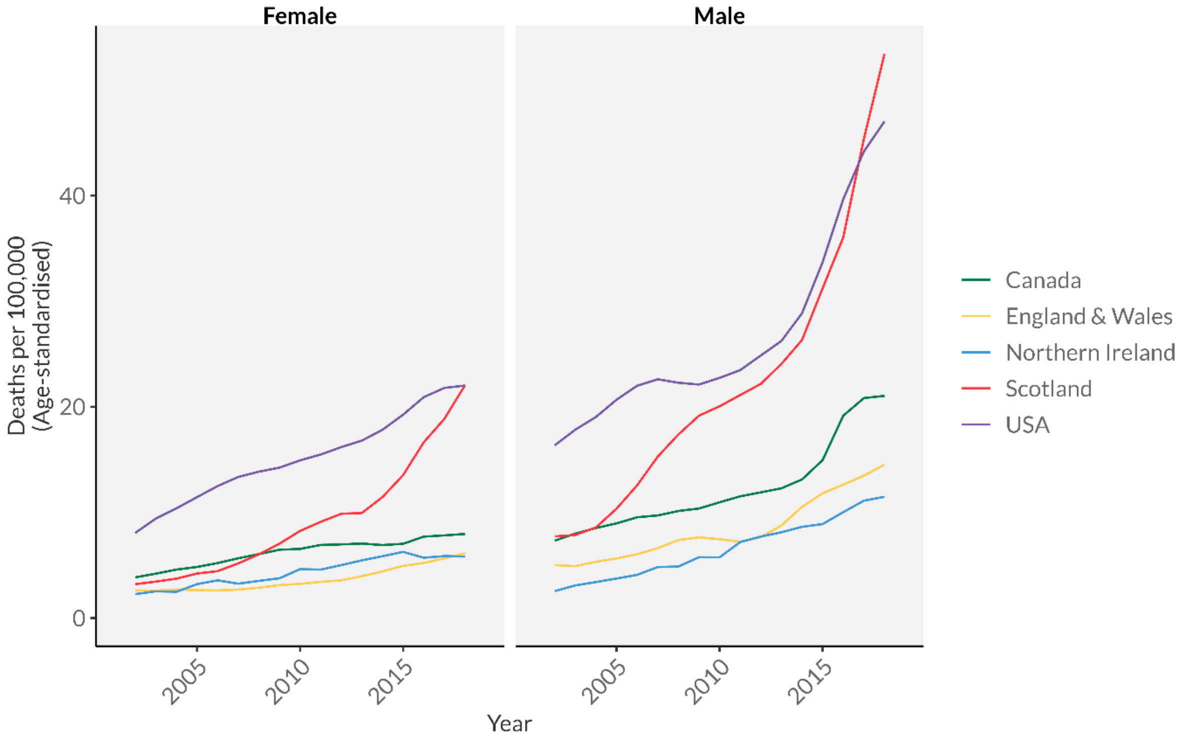

Figure S2b - Suicide mortality in ages 35-64 by sex in Canada, the UK and the USA

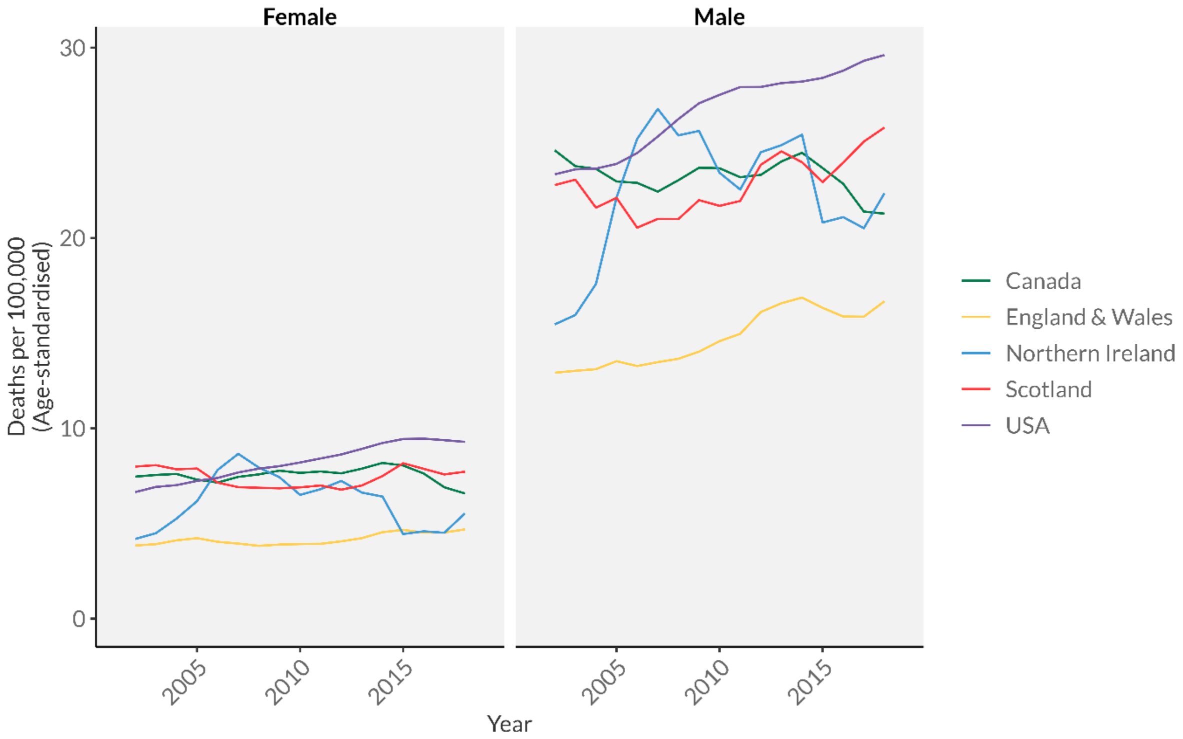

Figure S2c – Alcohol-specific mortality in ages 35-64 by sex in Canada, the UK and the USA

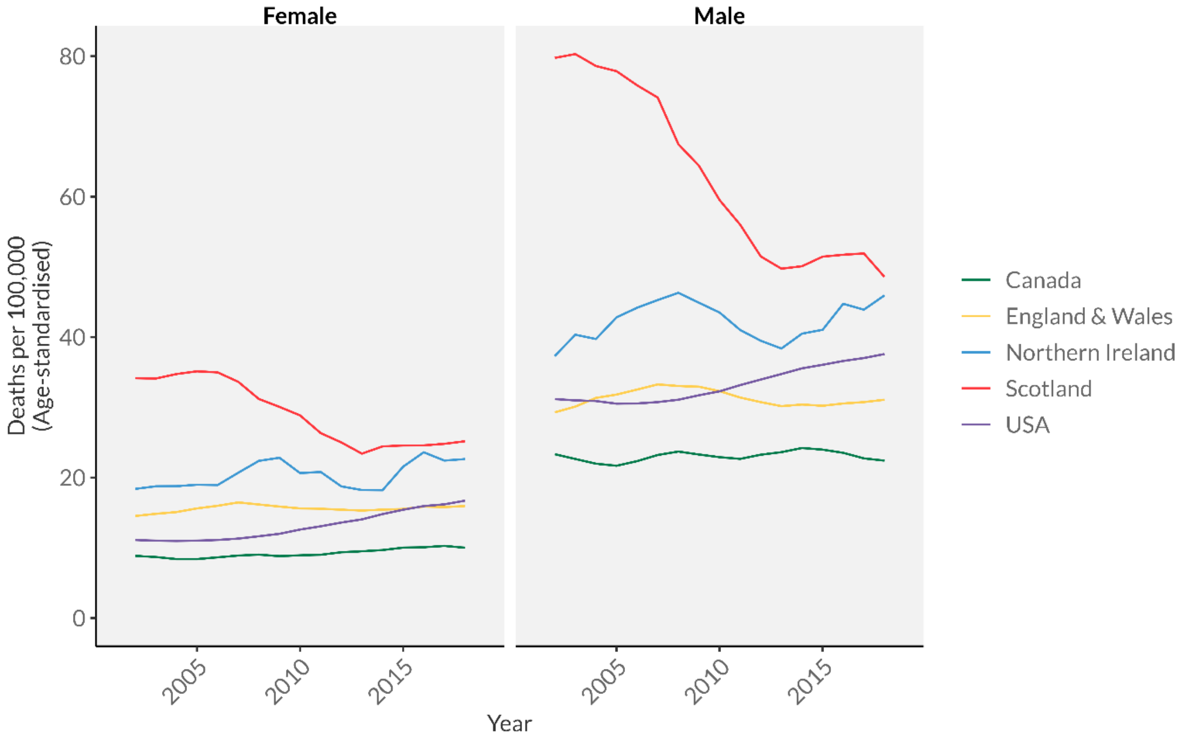

For peer review only

Figure S3 - Mortality from combined 'deaths of despair' by age and sex in Canada, the UK and the US, 2001-2019

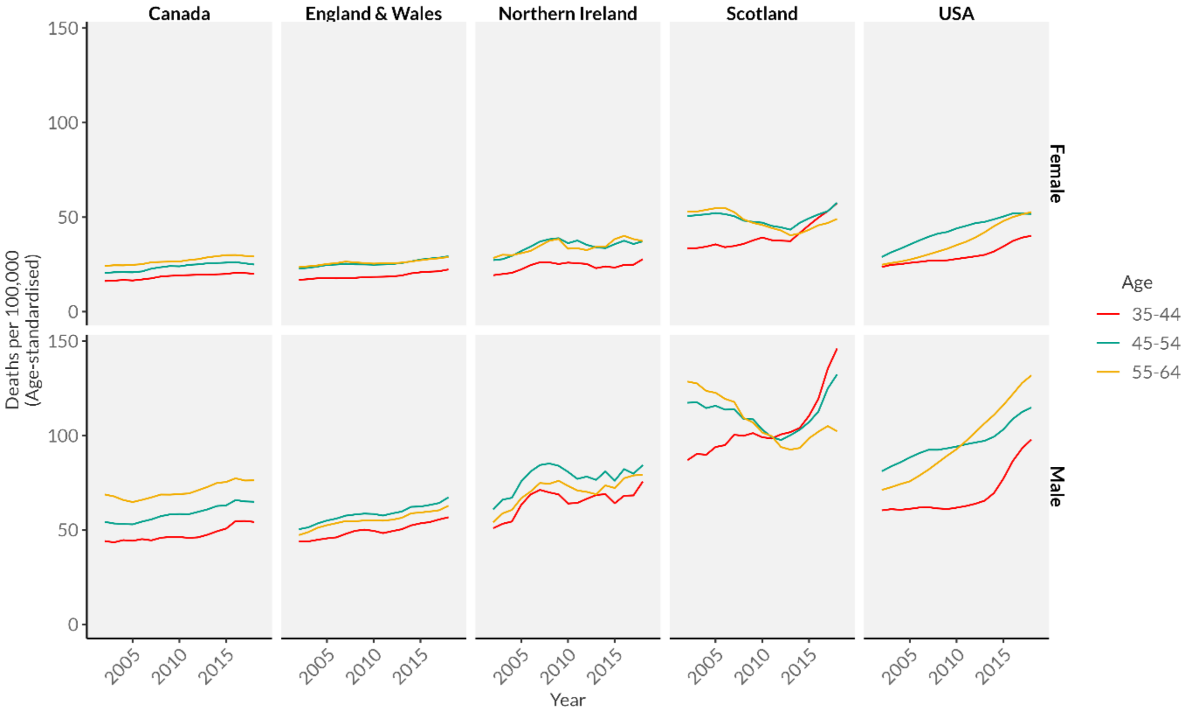

Figure S3a - Drug-related mortality by age and sex in Canada, the UK and the US, 2001-2019

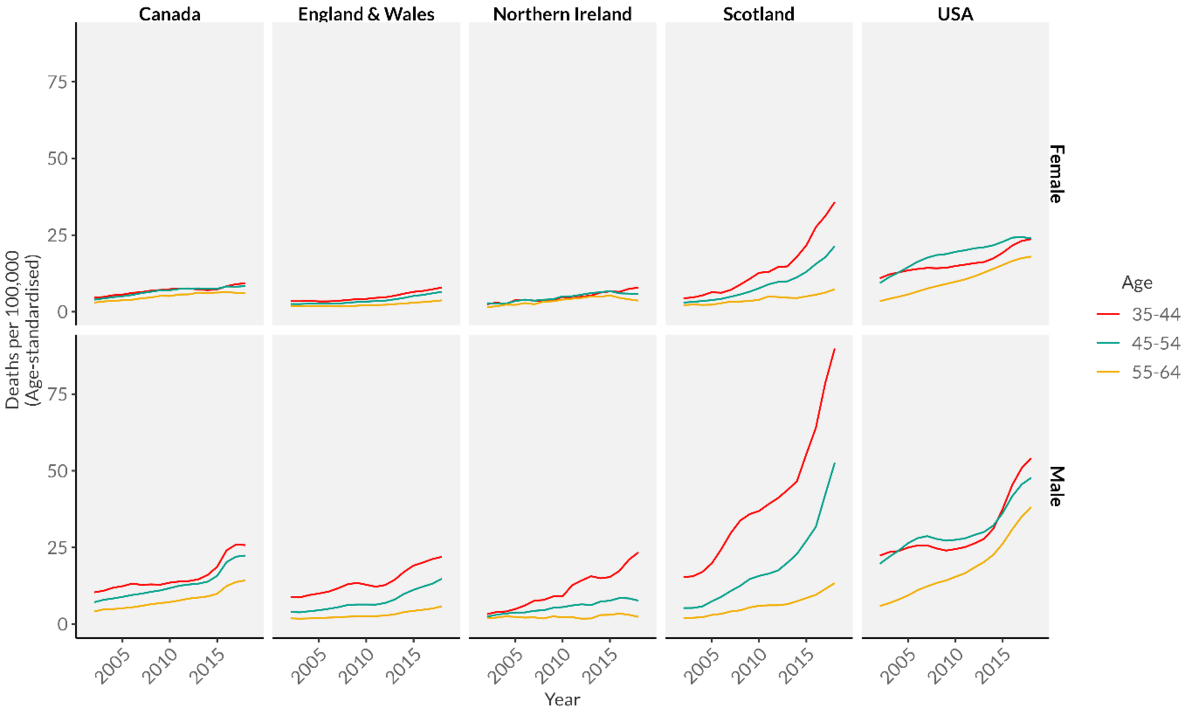

Figure S3b - Suicide mortality by age and sex in Canada, the UK and the US, 2001-2019

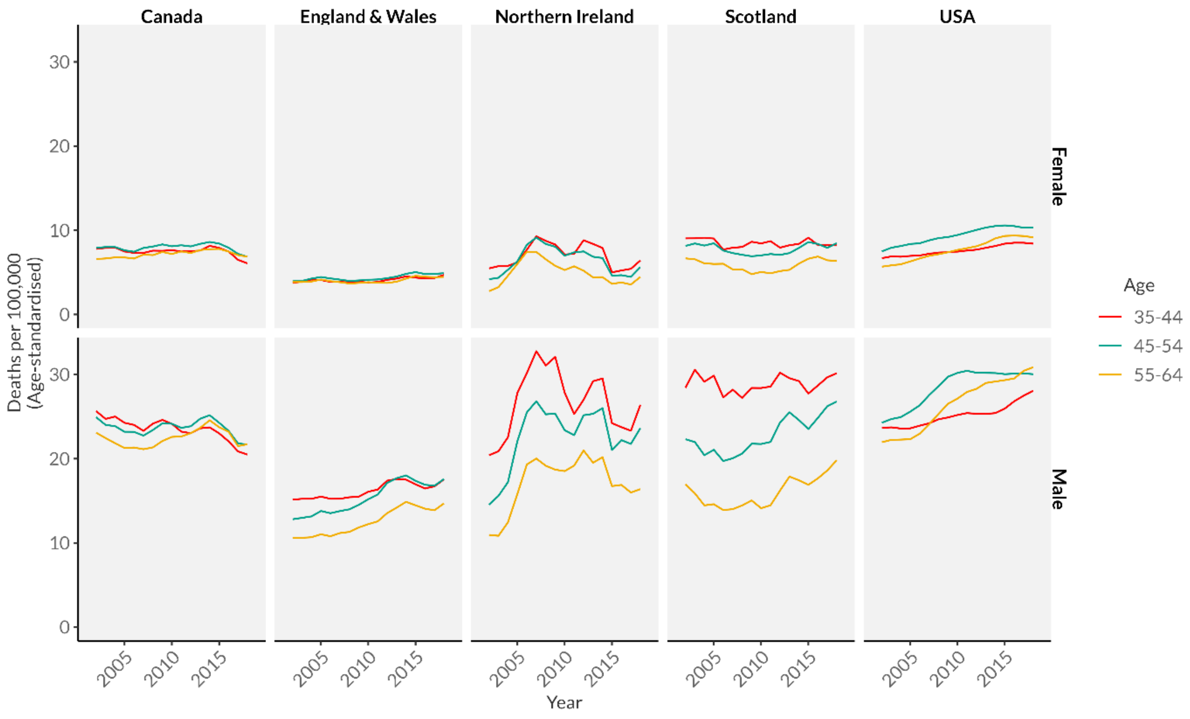

Figure S3c – alcohol-specific mortality by age and sex in Canada, the UK and the US, 2001-2019

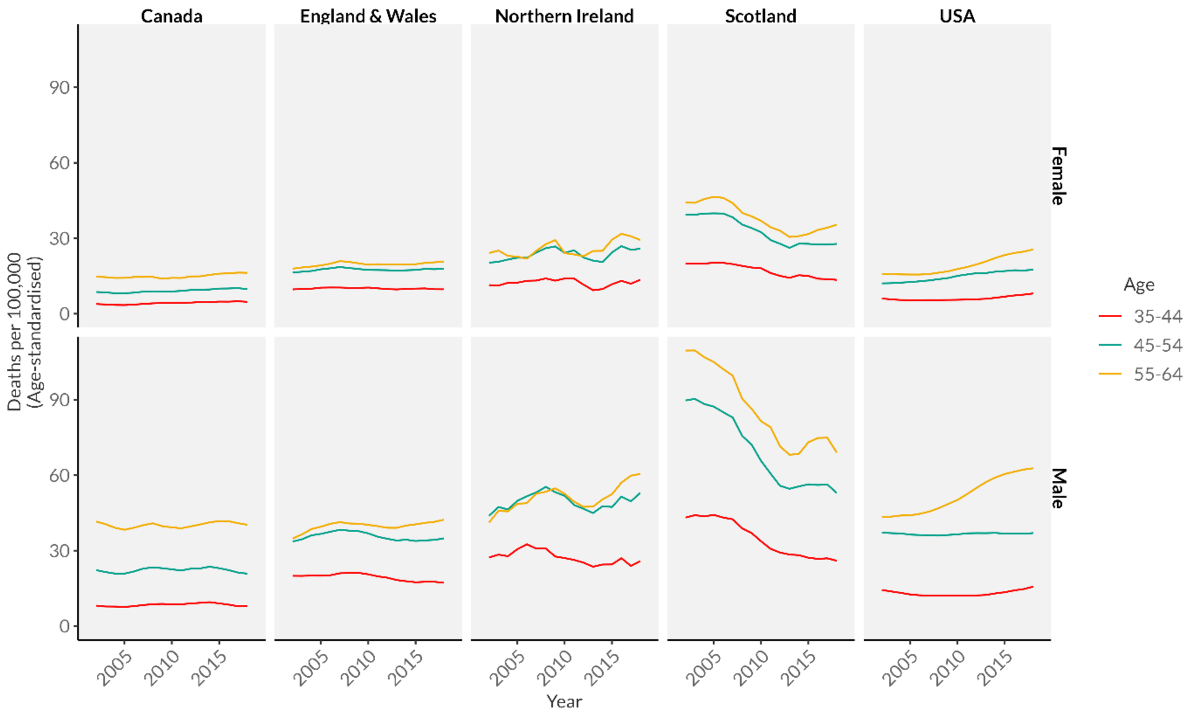

Figure S4a - Lexis surface showing age-specific mortality rates for males by cause, year and country

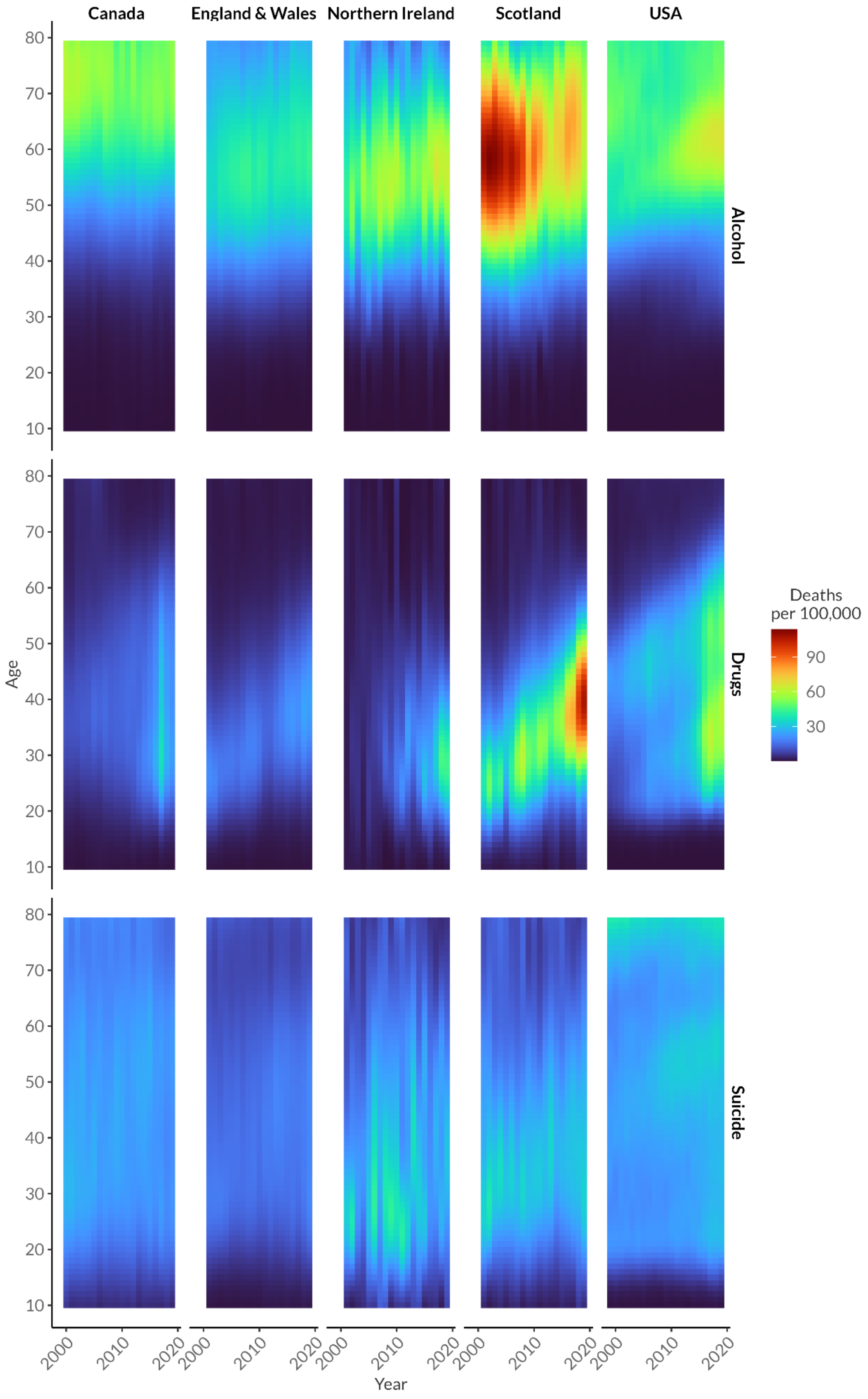

Figure S4b - Lexis surface showing age-specific mortality rates for females by cause, year and country

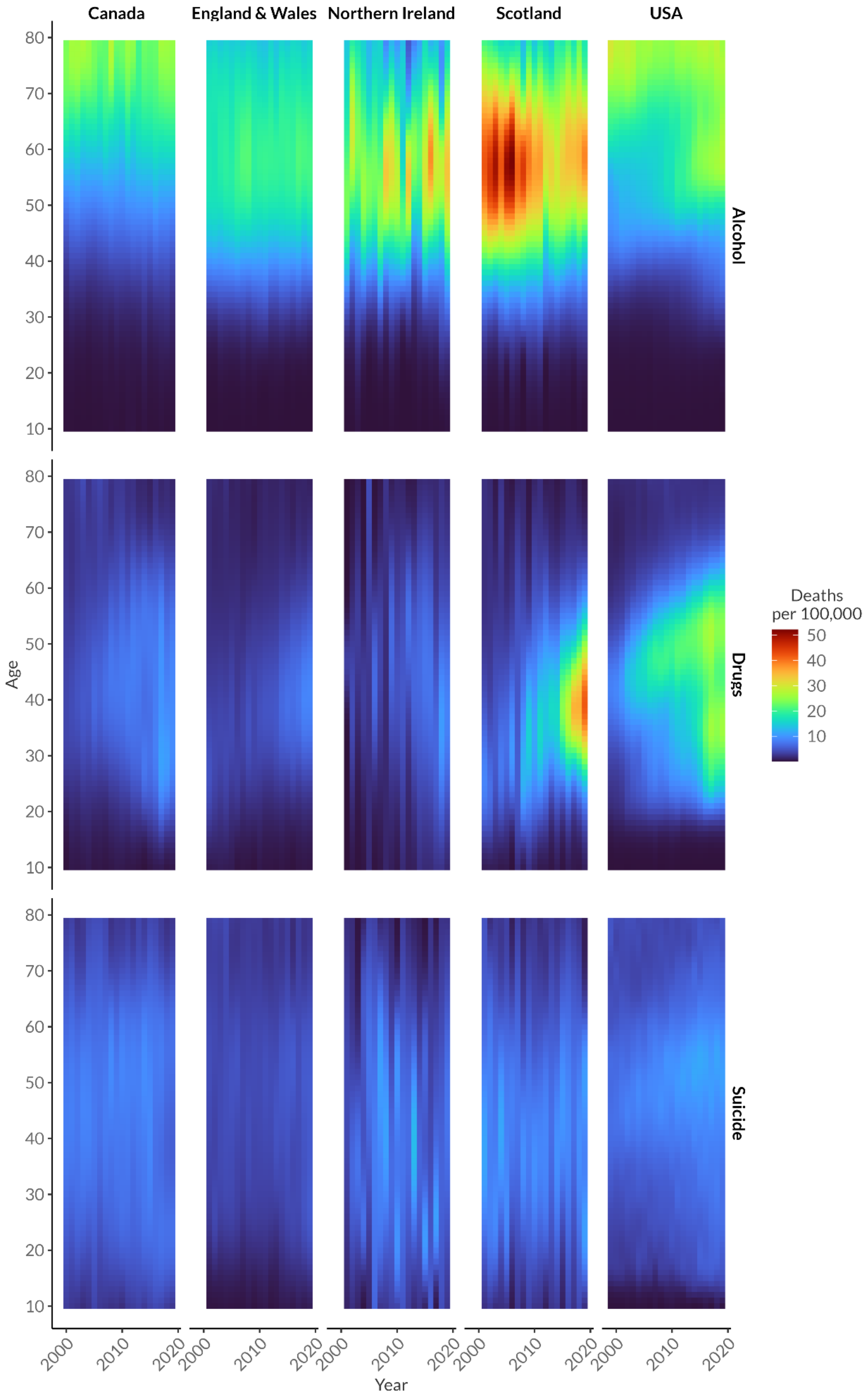

Figure S5a - Proportion of all deaths accounted for by alcohol, drugs and suicide among ages 35-44

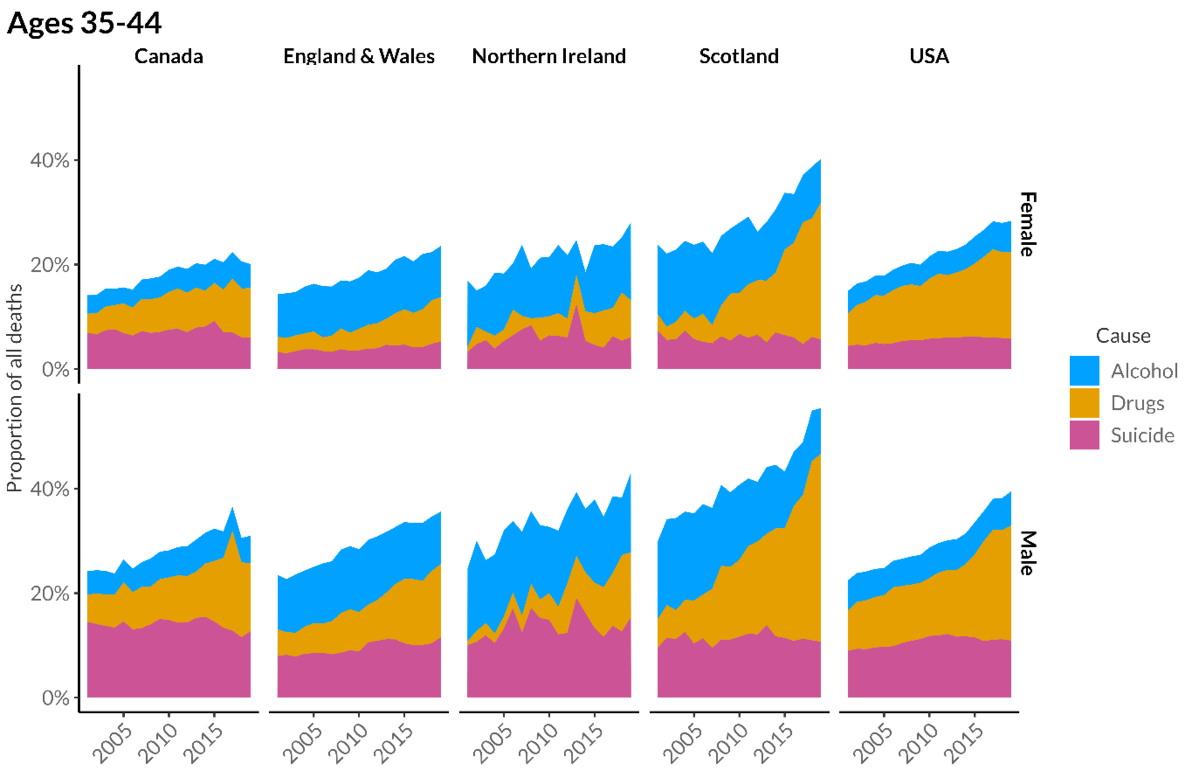

Figure S5b - Proportion of all deaths accounted for by alcohol, drugs and suicide among ages 45-54

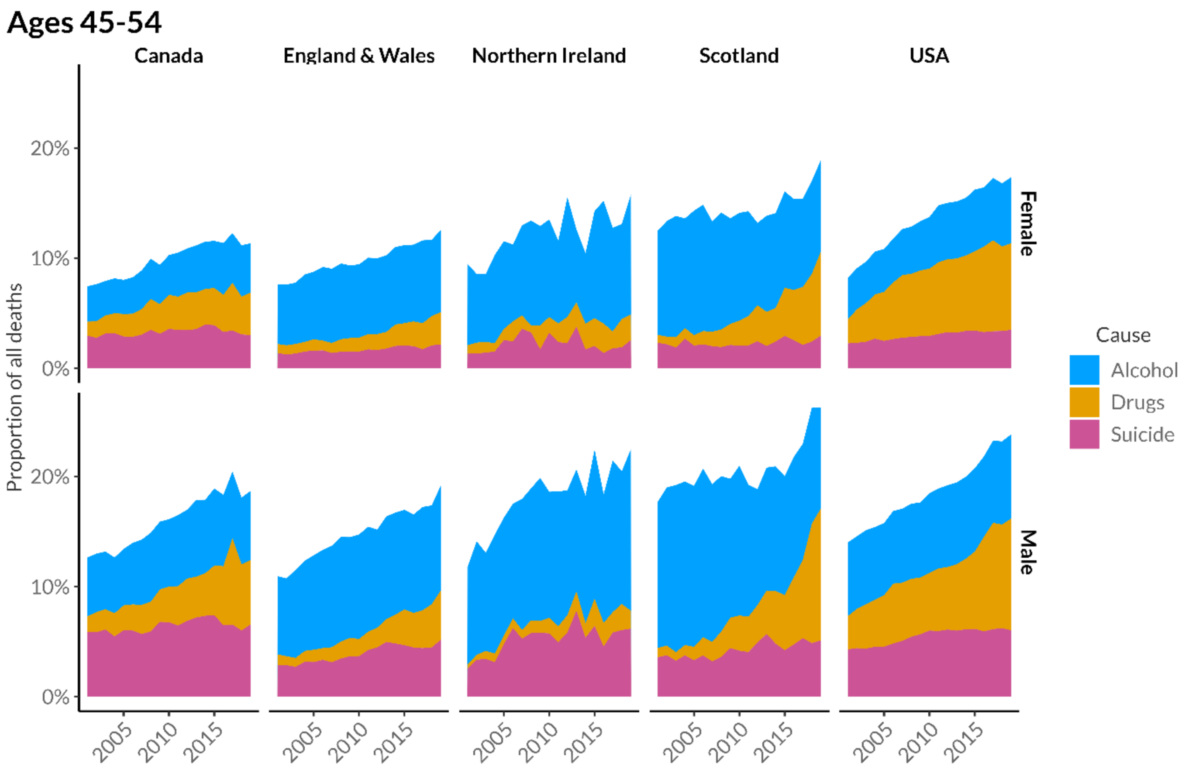

For peer review only - http://bmjopen.bmj.com/site/about/guidelines.xhtml

Figure S5c - Proportion of all deaths accounted for by alcohol, drugs and suicide among ages 55-64

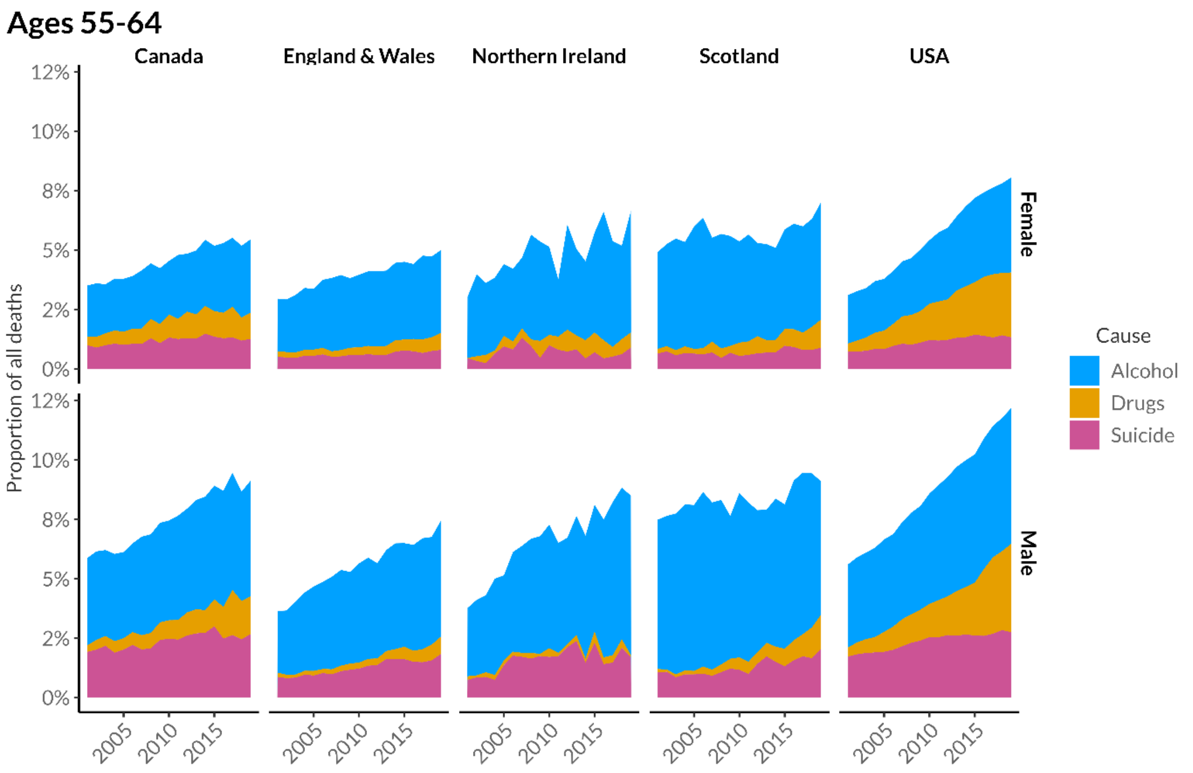

Table S1 - ICD-10 codes used to define 'deaths of despair' categories

Cause group	ICD-10 codes included
Suicide	U03, X60-84, Y87
Alcohol-related	K70, K73-74, F10, X45, Y15
Drug-related	F11-16, F18-19, X40-44, X85, Y10-14

review only

Table S2a - Male mortality rates (per 100,000 population) by cause, age group and year for Canada

Age group	2001	2002	2003	2004	2005	2006	2007	2008	2009	2010	2011	2012	2013	2014	2015	2016	2017	2018	2019
All-Cause																			
35-44	183.6	179.4	182.6	177.9	179.1	174.9	173.3	170.6	169.0	164.1	157.8	157.5	156.2	157.2	158.0	162.4	167.9	167.9	162.0
45-54	424.3	419.3	415.8	404.1	399.2	388.9	388.2	383.5	373.5	361.3	350.2	347.6	347.7	344.4	340.3	344.6	342.0	344.2	334.8
55-64	1156.0	1135.9	1104.2	1075.0	1045.1	1006.9	1010.8	988.8	949.3	917.0	901.3	886.8	884.5	877.6	860.8	863.1	845.6	845.1	825.7
Combined 'Deaths of Despair'																			
35-44	44.4	43.8	44.3	42.3	47.4	43.2	44.9	45.5	47.2	46.3	45.5	45.6	47.3	49.6	51.1	51.6	61.4	51.3	50.1
45-54	53.7	54.5	54.8	51.1	53.6	54.3	55.4	57.1	59.4	58.2	57.8	59.0	62.1	61.6	64.3	63.2	70.0	62.3	62.6
55-64	67.9	69.9	68.6	65.0	64.0	65.4	68.5	68.0	69.8	68.3	69.0	70.5	73.6	74.2	76.8	75.2	80.0	73.3	75.5
Alcohol																			
35-44	8.2	8.0	8.2	7.2	7.9	7.8	8.2	9.1	8.9	8.5	8.6	9.0	9.6	9.2	9.8	8.2	7.7	7.9	8.4
45-54	22.7	22.2	21.8	20.5	20.5	21.7	23.0	24.0	23.0	22.1	22.6	21.7	24.2	23.0	23.8	22.3	20.7	20.9	21.0
55-64	42.5	42.3	39.9	39.5	37.9	37.6	41.9	41.0	39.7	38.5	39.6	38.7	40.7	41.9	41.2	42.2	41.7	38.9	40.2
Drugs																			
35-44	9.7	10.5	11.0	11.2	13.4	12.5	13.7	12.4	12.9	13.4	14.3	14.0	13.8	16.0	18.3	21.8	32.1	24.0	21.1
45-54	6.1	7.7	7.7	8.5	9.0	9.2	10.3	10.3	11.0	11.7	12.5	13.4	12.9	13.3	15.4	18.5	26.8	20.7	19.6
55-64	3.2	4.7	4.6	5.2	5.0	5.4	6.1	6.4	7.1	7.1	7.5	8.6	9.0	8.4	9.8	11.5	16.1	13.6	13.1
Suicide																			
35-44	26.6	25.3	25.1	23.8	26.1	22.9	23.1	24.0	25.4	24.4	22.6	22.6	23.8	24.4	23.0	21.7	21.5	19.4	20.6
45-54	24.9	24.5	25.4	22.1	24.1	23.4	22.1	22.8	25.4	24.4	22.6	23.9	25.0	25.3	25.2	22.3	22.4	20.7	22.0
55-64	22.3	22.9	24.1	20.3	21.1	22.3	20.5	20.5	23.0	22.7	22.0	23.2	23.9	23.9	25.9	21.5	22.3	20.7	22.1

ew only

For peer review only - http://bmjopen.bmj.com/site/about/guidelines.xhtml

Table S2b - Female mortality rates (per 100,000 population) by cause, age group and year in Canada

Age group	2001	2002	2003	2004	2005	2006	2007	2008	2009	2010	2011	2012	2013	2014	2015	2016	2017	2018	2019
All-Cause																			
35-44	112.1	111.7	110.5	106.9	108.8	106.3	105.2	108.2	107.3	100.2	100.6	100.8	96.3	98.9	95.4	98.6	96.1	95.5	94.6
45-54	268.6	268.4	264.8	257.6	257.2	251.8	252.1	251.8	246.4	235.9	234.3	232.9	226.2	227.2	223.4	227.3	219.6	213.2	212.6
55-64	685.8	685.2	672.6	657.5	647.5	631.1	629.3	617.5	597.5	586.7	576.6	569.2	564.0	560.8	566.1	564.0	555.1	540.0	530.0
Combined 'Deaths of Despair'																			
35-44	15.9	15.8	17.0	16.4	17.0	16.1	18.0	18.8	19.0	19.1	19.7	19.3	19.5	19.7	20.1	20.2	21.5	19.7	19.0
45-54	20.0	20.5	21.0	21.0	20.7	20.8	22.4	25.1	23.1	24.3	24.6	25.3	25.2	26.1	25.8	25.9	27.0	23.8	24.1
55-64	24.1	24.7	24.0	25.0	24.6	24.7	26.1	27.5	25.4	26.7	27.7	27.6	28.1	30.5	29.4	29.9	30.7	28.0	28.9
Alcohol																			
35-44	4.0	3.9	3.8	3.3	3.3	3.7	3.9	4.3	4.3	4.2	4.2	4.6	4.5	4.9	4.3	5.2	4.8	5.0	4.3
45-54	8.6	9.0	8.3	8.2	8.0	8.3	8.9	9.2	8.8	8.5	9.4	9.3	9.6	9.8	9.5	10.7	9.9	9.9	9.5
55-64	14.7	15.4	13.9	14.3	14.4	14.0	15.4	14.4	14.1	13.2	15.5	13.9	15.2	15.6	15.6	16.5	16.1	16.3	16.4
Drugs																			
35-44	4.1	4.5	5.0	4.9	6.2	5.7	6.5	7.0	7.1	7.2	7.8	7.6	7.4	6.8	7.0	8.1	9.9	8.9	9.0
45-54	3.4	4.0	4.4	4.7	5.3	5.3	5.9	7.1	6.6	7.3	7.1	7.9	7.6	7.3	7.6	7.7	9.5	7.3	8.2
55-64	2.5	3.1	3.4	3.6	3.6	4.0	4.1	5.0	4.8	5.7	4.9	6.3	5.8	6.5	6.1	6.1	7.1	5.3	5.8
Suicide																			
35-44	7.8	7.4	8.2	8.1	7.5	6.8	7.6	7.5	7.6	7.6	7.7	7.1	7.6	8.0	8.8	6.9	6.7	5.8	5.7
45-54	8.0	7.4	8.4	8.2	7.4	7.2	7.7	8.8	7.7	8.4	8.1	8.1	8.1	9.0	8.7	7.5	7.6	6.5	6.5
55-64	6.9	6.2	6.7	7.0	6.6	6.7	6.6	8.0	6.5	7.8	7.3	7.4	7.2	8.3	7.7	7.3	7.4	6.5	6.7

ew only

For peer review only - http://bmjopen.bmj.com/site/about/guidelines.xhtml

Table S3a - Male mortality rates (per 100,000 population) by cause, age group and year for England & Wales

Age group	2001	2002	2003	2004	2005	2006	2007	2008	2009	2010	2011	2012	2013	2014	2015	2016	2017	2018	2019
All-Cause																			
35-44	190.7	188.3	188.4	183.7	181.3	181.5	177.1	179.8	177.7	168.9	162.8	155.2	161.4	159.5	160.7	162.9	161.7	168.0	162.8
45-54	461.4	453.4	451.1	433.0	428.0	424.2	412.7	410.8	402.8	393.9	382.2	368.3	375.6	370.5	372.9	374.7	374.4	382.0	371.0
55-64	1280.1	1251.8	1226.2	1159.3	1129.0	1100.6	1070.2	1044.7	1003.7	991.9	954.9	929.1	926.8	913.1	917.4	916.9	907.3	910.9	888.6
Combined 'Deaths of Despair'																			
35-44	44.8	42.8	44.4	44.7	45.4	46.7	46.2	51.0	51.5	48.0	49.2	47.9	51.2	52.1	54.1	54.5	54.1	58.3	58.0
45-54	50.5	48.7	52.0	53.5	55.0	56.6	56.6	59.6	58.4	58.0	59.0	55.9	61.5	62.0	63.3	62.0	64.5	66.4	71.1
55-64	46.6	45.9	49.5	51.3	52.8	53.6	54.4	56.1	53.1	56.0	56.3	52.5	57.6	59.3	59.6	58.9	60.9	61.6	66.1
Alcohol																			
35-44	19.8	19.1	21.0	19.7	19.5	21.0	20.3	21.9	21.4	20.3	20.2	18.9	18.7	17.4	17.5	17.5	17.9	17.5	16.3
45-54	32.7	32.0	36.1	35.5	36.7	37.9	38.0	39.0	36.9	37.4	36.4	32.8	35.0	34.3	33.7	33.5	35.1	34.3	35.3
55-64	33.1	33.8	37.5	38.2	40.1	40.3	41.6	42.1	38.7	41.4	40.7	37.2	39.4	40.6	40.0	40.8	42.3	41.3	43.3
Drugs																			
35-44	9.8	8.2	8.6	9.5	10.4	10.1	11.3	13.6	14.0	12.8	11.8	12.1	14.4	16.9	19.9	20.6	19.9	23.3	22.8
45-54	4.6	3.7	3.6	4.3	4.8	4.5	5.6	6.4	6.7	6.1	6.3	6.6	7.8	9.7	12.1	11.8	12.9	15.0	16.6
55-64	2.3	1.8	1.6	1.9	2.2	1.9	2.3	2.4	2.6	2.5	2.8	2.6	3.1	3.9	4.8	4.3	5.0	6.0	6.5
Suicide																			
35-44	15.2	15.4	14.8	15.5	15.4	15.6	14.7	15.5	16.1	14.9	17.2	16.9	18.1	17.8	16.7	16.4	16.3	17.4	18.9
45-54	13.2	13.0	12.3	13.7	13.5	14.2	12.9	14.2	14.8	14.5	16.2	16.5	18.7	17.9	17.5	16.7	16.5	17.1	19.1
55-64	11.1	10.3	10.3	11.2	10.5	11.4	10.5	11.6	11.8	12.1	12.8	12.8	15.1	14.7	14.8	13.8	13.5	14.3	16.3

view only

For peer review only - http://bmjopen.bmj.com/site/about/guidelines.xhtml

Table S3b - Female mortality rates (per 100,000 population) by cause, age group and year in England & Wales

Age group	2001	2002	2003	2004	2005	2006	2007	2008	2009	2010	2011	2012	2013	2014	2015	2016	2017	2018	2019
All-Cause																			
35-44	117.5	115.0	116.4	112.2	111.6	110.4	109.3	110.2	105.6	104.8	100.9	97.4	97.8	98.8	99.0	99.3	97.8	99.2	98.7
45-54	302.5	297.0	295.1	282.4	281.3	276.6	273.2	272.8	262.3	260.4	253.5	249.5	246.7	244.2	247.4	251.2	245.3	248.1	243.0
55-64	802.5	784.8	770.3	735.2	722.1	700.6	687.0	680.8	651.2	641.2	629.7	623.5	612.7	600.7	608.3	621.5	599.8	608.3	587.8
Combined 'Deaths of Despair'																			
35-44	16.8	16.7	17.1	17.7	18.2	17.5	17.3	18.7	17.7	18.3	19.1	18.0	18.8	20.7	21.4	20.4	21.6	22.2	23.2
45-54	23.0	22.6	23.0	24.1	24.7	25.5	24.7	26.0	24.5	24.6	25.5	24.9	25.3	26.9	27.7	28.2	28.5	28.9	30.5
55-64	23.7	23.0	24.0	25.1	24.3	26.3	26.3	26.9	24.9	25.4	25.9	25.6	25.4	26.8	27.4	27.4	28.6	28.8	29.4
Alcohol																			
35-44	9.6	9.8	9.6	10.0	10.2	10.8	10.2	10.2	10.4	10.2	10.6	9.5	9.4	10.1	10.1	9.8	10.4	9.1	9.7
45-54	16.4	16.4	16.4	17.3	17.3	18.5	18.4	18.8	17.3	17.3	17.6	17.2	17.2	17.2	17.5	17.5	18.5	17.2	18.2
55-64	17.7	17.4	18.7	19.1	18.4	20.1	21.2	21.5	19.0	19.6	19.7	19.7	19.4	19.6	19.9	19.6	21.1	20.6	20.5
Drugs																			
35-44	3.5	3.4	3.5	3.4	3.8	2.9	3.4	4.4	3.7	4.3	4.6	4.7	4.9	6.2	6.7	6.6	7.1	8.3	8.4
45-54	2.5	2.5	2.6	2.5	2.9	2.4	2.5	3.1	3.2	3.3	3.5	3.6	3.7	4.8	5.0	5.7	5.8	6.6	7.1
55-64	1.9	1.8	1.8	1.8	1.9	1.9	1.6	1.8	2.0	2.1	2.2	2.2	2.3	2.9	2.8	3.2	3.4	3.5	4.2
Suicide																			
35-44	3.8	3.5	4.0	4.2	4.2	3.8	3.6	4.2	3.7	3.8	3.9	3.9	4.5	4.4	4.6	4.1	4.1	4.8	5.2
45-54	4.2	3.7	4.0	4.3	4.5	4.5	3.8	4.1	4.0	4.0	4.4	4.1	4.4	4.9	5.1	5.1	4.3	5.2	5.3
55-64	4.1	3.8	3.6	4.1	4.0	4.3	3.6	3.6	3.8	3.7	3.9	3.7	3.6	4.4	4.8	4.6	4.0	4.6	4.7

For peer review only - http://bmjopen.bmj.com/site/about/guidelines.xhtml

Table S4a - Male mortality rates (per 100,000 population) by cause, age group and year for Northern Ireland

Age group	2001	2002	2003	2004	2005	2006	2007	2008	2009	2010	2011	2012	2013	2014	2015	2016	2017	2018	2019
All-Cause																			
35-44	192.7	200.4	170.8	199.3	199.3	211.6	223.7	199.2	204.4	207.7	177.2	189.6	188.9	173.4	184.3	172.7	193.7	183.7	190.4
45-54	465.7	497.4	438.8	479.3	450.5	475.5	480.3	440.9	431.9	443.7	396.4	398.5	417.9	375.3	393.1	388.9	405.0	395.8	378.1
55-64	1324.3	1385.5	1282.8	1282.1	1214.4	1205.1	1170.8	1137.3	1067.7	1092.9	1042.6	971.1	1011.6	940.0	977.1	977.9	967.5	947.7	876.9
Combined 'Deaths of Despair'																			
35-44	47.6	60.2	45.0	54.7	63.8	71.6	71.1	71.1	67.5	67.9	56.7	68.4	74.4	62.6	70.0	59.9	74.7	70.4	81.9
45-54	54.9	70.2	57.4	70.5	73.3	83.4	86.3	83.5	85.9	82.6	73.9	74.7	86.3	68.5	88.3	71.5	86.9	81.0	85.0
55-64	50.0	57.0	55.2	64.2	62.5	73.8	74.8	76.1	72.6	79.6	67.9	65.3	77.3	64.1	79.3	73.3	79.6	83.7	74.5
Alcohol																			
35-44	26.7	34.7	20.5	30.1	32.8	28.9	35.9	27.6	29.1	26.3	25.8	26.8	23.1	21.0	29.4	23.3	28.4	20.2	29.0
45-54	41.2	51.3	39.1	51.7	48.3	49.5	57.1	52.9	56.2	50.8	48.5	45.2	46.3	43.4	53.3	45.4	55.8	47.7	55.5
55-64	38.1	44.0	41.5	52.0	43.5	50.5	52.7	54.7	52.8	56.8	48.2	43.4	50.6	48.5	52.1	56.7	62.3	60.5	58.8
Drugs																			
35-44	1.6	4.1	4.1	3.7	4.6	6.5	7.2	9.2	7.3	10.6	9.4	18.0	15.3	13.6	16.1	16.5	19.6	26.9	23.6
45-54	1.8	2.2	3.2	3.8	3.5	4.2	3.8	5.1	4.7	6.3	5.8	6.3	7.4	5.0	9.7	8.3	7.5	9.4	6.2
55-64	2.0	1.2	2.5	2.6	2.4	2.0	2.0	2.6	1.0	4.1	1.5	1.2	2.6	1.7	4.6	2.8	3.0	3.4	0.8
Suicide																			
35-44	19.4	21.4	20.4	20.8	26.4	36.2	27.9	34.2	31.1	31.0	21.4	23.5	36.0	28.1	24.5	20.1	26.6	23.2	29.3
45-54	11.9	16.7	15.0	15.0	21.5	29.7	25.3	25.5	25.0	25.5	19.6	23.3	32.6	20.1	25.3	17.7	23.6	24.0	23.4
55-64	9.9	11.8	11.2	9.6	16.6	21.3	20.1	18.7	18.7	18.7	18.2	20.6	24.1	13.8	22.6	13.8	14.3	19.9	15.0

For peer review only

For peer review only - http://bmjopen.bmj.com/site/about/guidelines.xhtml

Table S4b - Female mortality rates (per 100,000 population) by cause, age group and year in Northern Ireland

Age group	2001	2002	2003	2004	2005	2006	2007	2008	2009	2010	2011	2012	2013	2014	2015	2016	2017	2018	2019
All-Cause																			
35-44	119.4	121.7	121.7	119.8	110.2	122.8	121.5	127.5	116.4	122.3	112.9	108.8	102.3	107.8	111.8	98.3	103.7	105.9	115.0
45-54	310.2	311.2	301.8	300.3	275.8	298.1	292.2	297.0	288.4	292.7	274.7	267.2	259.3	267.3	281.2	257.1	261.0	267.6	274.0
55-64	809.9	819.0	772.8	774.6	722.8	740.3	711.3	710.9	727.4	704.9	656.3	652.0	659.5	663.0	689.5	688.4	654.1	663.1	639.7
Combined 'Deaths of Despair'																			
35-44	20.1	18.3	19.4	22.1	20.1	24.8	28.9	24.6	24.8	26.3	26.8	23.7	25.3	20.0	26.5	23.5	24.3	26.7	32.3
45-54	29.4	26.6	25.8	30.9	31.8	33.5	37.9	39.8	37.3	39.6	31.8	41.5	32.7	27.9	40.2	39.1	33.3	35.0	43.3
55-64	24.6	32.6	28.0	29.8	31.9	31.2	33.3	40.2	39.0	36.3	24.7	39.6	33.3	30.1	39.6	45.6	35.2	34.4	42.5
Alcohol																			
35-44	15.0	8.5	10.7	14.4	11.8	10.9	16.6	12.2	13.4	13.9	14.7	13.2	6.7	8.1	14.6	12.4	12.2	11.2	17.1
45-54	22.9	19.4	18.6	24.0	22.0	20.8	23.9	28.3	26.0	25.9	20.6	29.1	17.2	17.1	27.4	28.7	24.5	23.1	29.9
55-64	20.8	28.2	23.4	23.7	21.8	22.6	21.2	31.3	30.4	26.1	15.8	28.8	24.0	22.0	29.0	37.2	29.1	26.1	32.7
Drugs																			
35-44	1.2	4.0	2.0	3.0	2.4	6.0	3.1	1.7	5.1	4.4	4.9	4.0	5.9	6.1	6.8	7.0	5.6	9.8	8.2
45-54	2.4	3.1	2.9	2.3	2.8	5.4	3.6	1.9	6.3	4.2	4.7	6.3	5.7	6.2	7.1	6.9	4.0	6.9	6.4
55-64	0.2	1.6	2.7	1.0	3.2	2.5	2.8	2.0	5.2	3.1	3.6	6.0	3.8	5.1	5.7	5.2	2.6	4.2	4.1
Suicide																			
35-44	3.9	5.8	6.7	4.7	5.9	7.9	9.2	10.7	6.3	7.9	7.2	6.6	12.7	5.8	5.1	4.0	6.5	5.7	7.0
45-54	4.1	4.2	4.3	4.6	7.1	7.3	10.5	9.7	5.0	9.5	6.5	6.2	9.8	4.6	5.7	3.6	4.7	5.1	7.0
55-64	3.6	2.8	1.9	5.1	6.9	6.1	9.4	6.8	3.5	7.1	5.3	4.8	5.5	3.0	4.9	3.1	3.4	4.1	5.8

For peer review only - http://bmjopen.bmj.com/site/about/guidelines.xhtml

Table S5a - Male mortality rates (per 100,000 population) by cause, age group and year for Scotland

Age group	2001	2002	2003	2004	2005	2006	2007	2008	2009	2010	2011	2012	2013	2014	2015	2016	2017	2018	2019
All-Cause																			
35-44	274.9	267.0	254.2	259.0	254.9	268.9	262.0	261.1	249.4	244.0	237.5	232.9	240.2	231.4	237.8	265.8	266.1	273.8	284.2
45-54	649.0	634.1	606.2	591.9	580.9	580.7	567.1	557.3	530.8	520.1	495.1	493.0	502.7	491.9	507.0	536.6	520.6	525.7	529.8
55-64	1708.3	1712.4	1635.3	1538.6	1468.8	1429.7	1406.5	1370.3	1287.4	1257.4	1194.7	1175.9	1149.4	1121.3	1172.9	1163.9	1101.5	1106.0	1076.2
Combined 'Deaths of Despair'																			
35-44	82.2	91.2	87.4	92.3	89.9	99.7	95.2	106.4	98.0	99.5	99.7	96.2	106.0	103.2	102.9	125.2	130.2	150.5	157.5
45-54	114.9	120.5	116.5	115.7	111.3	120.3	109.5	111.6	105.1	109.1	95.3	92.9	104.6	102.9	101.5	116.9	119.6	138.1	139.2
55-64	127.9	131.0	126.7	125.0	119.1	123.7	115.5	114.0	98.4	108.3	98.0	92.8	90.9	93.9	95.4	106.3	104.3	104.4	98.0
Alcohol																			
35-44	40.9	43.8	44.8	43.8	42.4	46.5	40.6	40.4	35.6	35.0	30.6	26.6	30.6	28.2	25.8	27.6	26.9	26.4	24.7
45-54	86.1	91.1	92.0	87.9	85.0	88.9	81.3	78.7	67.0	70.8	59.3	51.9	56.2	55.7	54.8	58.7	54.9	55.4	48.5
55-64	107.3	110.7	110.7	107.4	102.6	105.0	98.9	94.8	77.3	87.0	80.0	70.2	64.4	69.8	71.3	78.2	74.9	71.7	60.4
Drugs																			
35-44	15.1	16.8	14.1	15.9	21.1	22.5	29.7	36.9	34.8	36.0	40.0	41.4	42.0	47.8	50.0	68.7	73.3	94.0	102.4
45-54	5.5	5.5	4.7	5.6	7.1	9.7	10.0	12.8	14.7	16.6	15.9	16.9	19.8	23.5	25.4	32.7	37.1	57.1	63.6
55-64	2.3	1.7	2.0	2.6	2.1	4.3	3.7	4.5	5.3	6.6	6.1	5.8	6.6	7.2	8.6	9.8	10.3	14.4	15.6
Suicide																			
35-44	26.2	30.6	28.4	32.6	26.3	30.6	24.9	29.1	27.7	28.5	29.1	28.2	33.4	27.1	27.2	28.9	30.0	30.1	30.4
45-54	23.2	23.9	19.8	22.2	19.2	21.8	18.2	20.2	23.4	21.7	20.1	24.2	28.6	23.7	21.4	25.5	27.7	25.6	27.1
55-64	18.3	18.6	14.0	15.0	14.4	14.4	12.9	14.7	15.8	14.7	11.9	16.8	19.9	16.9	15.5	18.3	19.2	18.3	22.0

For peer review only - http://bmjopen.bmj.com/site/about/guidelines.xhtml

Table S5b - Female mortality rates (per 100,000 population) by cause, age group and year in Scotland

Age group	2001	2002	2003	2004	2005	2006	2007	2008	2009	2010	2011	2012	2013	2014	2015	2016	2017	2018	2019
All-Cause																			
35-44	149.2	147.2	139.8	148.5	146.7	146.0	143.7	144.5	144.9	133.4	142.0	132.1	129.8	133.1	141.3	146.4	141.8	149.7	153.4
45-54	387.9	382.8	376.1	366.9	367.0	363.6	362.0	347.5	344.8	328.6	336.3	317.0	317.7	314.8	327.2	333.5	327.8	338.9	341.1
55-64	1014.3	1009.6	1019.0	934.6	932.2	919.3	909.7	865.6	838.1	835.8	810.4	794.0	778.8	748.0	766.4	766.3	754.3	769.4	759.9
Combined 'Deaths of Despair'																			
35-44	35.6	32.6	32.0	36.4	34.9	35.6	31.9	36.8	38.9	37.4	41.4	34.7	36.5	40.7	47.7	49.0	52.6	58.0	61.7
45-54	48.4	51.2	52.0	49.9	52.5	54.0	48.4	49.2	47.0	46.4	48.0	41.9	44.0	44.4	52.6	51.3	50.5	57.8	64.7
55-64	49.9	53.1	55.9	49.9	55.8	58.5	50.3	49.2	46.9	44.9	45.9	42.1	40.9	38.1	45.0	46.8	45.3	48.6	53.3
Alcohol																			
35-44	19.9	20.6	19.3	19.9	20.7	20.2	19.8	19.2	18.0	17.9	18.4	12.3	14.5	16.1	15.3	13.7	12.8	14.8	12.8
45-54	36.7	40.2	41.4	36.5	41.5	41.7	36.3	36.9	33.1	32.2	32.0	23.7	27.7	27.2	28.7	27.5	26.3	28.7	28.4
55-64	41.3	43.4	47.9	41.0	47.9	50.4	39.8	41.7	38.8	35.6	36.4	31.1	31.5	28.9	32.2	34.0	33.7	34.9	37.5
Drugs																			
35-44	4.8	3.7	4.7	5.6	5.7	7.8	5.0	8.5	13.0	10.5	14.6	13.8	15.4	15.3	23.2	26.5	33.1	34.0	40.2
45-54	2.8	2.7	3.5	3.5	3.4	4.4	4.8	5.5	6.6	7.5	9.0	10.4	9.8	9.6	14.3	15.2	17.2	20.9	26.1
55-64	2.0	2.2	2.2	2.7	1.8	2.4	4.1	3.4	2.4	4.7	4.6	5.7	4.1	3.9	5.4	5.8	5.5	7.5	9.0
Suicide																			
35-44	10.9	8.2	7.9	11.0	8.4	7.6	7.1	9.1	7.9	8.9	8.5	8.7	6.6	9.3	9.2	8.8	6.8	9.2	8.7
45-54	9.0	8.3	7.1	9.9	7.6	7.9	7.3	6.7	7.3	6.7	7.0	7.8	6.4	7.6	9.6	8.5	7.0	8.2	10.2
55-64	6.6	7.5	5.9	6.3	6.0	5.7	6.3	4.0	5.7	4.6	4.8	5.3	5.4	5.3	7.5	7.1	6.0	6.2	6.8

For peer review only - http://bmjopen.bmj.com/site/about/guidelines.xhtml

Table S6a - Male mortality rates (per 100,000 population) by cause, age group and year for the USA

Age group	2001	2002	2003	2004	2005	2006	2007	2008	2009	2010	2011	2012	2013	2014	2015	2016	2017	2018	2019
All-Cause																			
35-44	259.2	258.0	254.3	244.0	244.0	240.4	233.5	225.5	223.9	212.3	213.6	211.8	212.9	216.6	226.2	244.2	252.2	251.7	257.3
45-54	551.3	557.1	561.1	551.0	556.2	548.7	536.6	531.3	524.2	508.9	507.4	500.0	497.9	494.0	494.0	496.7	495.4	490.2	484.9
55-64	1226.1	1216.1	1200.0	1157.0	1153.1	1126.4	1108.7	1110.6	1098.0	1082.8	1081.4	1090.8	1101.8	1112.5	1119.2	1124.2	1122.5	1124.4	1106.7
Combined 'Deaths of Despair'																			
35-44	58.2	61.6	61.4	60.1	60.5	63.0	62.1	61.0	61.2	61.0	63.2	63.8	64.6	68.1	76.0	87.3	96.0	96.2	101.6
45-54	77.2	81.2	84.9	84.9	87.8	92.5	91.6	93.2	92.5	94.1	95.9	96.0	96.9	98.7	102.5	108.4	115.2	113.7	115.5
55-64	68.7	71.7	73.2	73.0	76.8	77.5	81.9	86.5	88.6	93.0	96.7	101.3	107.0	111.1	114.6	122.6	128.4	132.3	134.8
Alcohol																			
35-44	14.9	14.3	14.0	13.1	12.6	12.1	12.1	12.1	11.9	12.4	12.1	12.0	12.6	12.7	13.8	14.0	14.8	15.5	16.9
45-54	36.9	36.8	37.8	36.4	36.5	36.2	36.0	36.2	35.7	36.8	36.8	37.1	37.0	36.8	37.4	36.3	36.9	37.0	37.1
55-64	42.9	43.5	43.7	43.5	44.8	43.8	45.3	47.5	47.9	50.3	52.3	54.8	57.6	59.3	60.4	61.7	62.1	63.0	63.2
Drugs																			
35-44	20.1	23.2	23.8	23.5	24.2	27.1	25.6	24.3	24.1	23.6	25.7	26.0	27.2	30.1	36.0	46.9	53.2	52.6	56.6
45-54	16.7	19.9	22.5	23.5	26.0	29.8	28.4	28.0	27.0	26.7	28.8	28.4	30.1	31.7	34.7	42.6	47.9	46.2	49.2
55-64	4.6	6.1	7.0	7.5	9.7	11.0	12.6	13.3	14.3	15.1	16.9	17.8	20.7	22.2	25.0	31.7	36.1	37.4	41.1
Suicide																			
35-44	23.3	24.1	23.6	23.5	23.7	23.7	24.4	24.5	25.1	25.1	25.4	25.8	24.8	25.3	26.1	26.4	28.0	28.1	28.1
45-54	23.7	24.6	24.5	25.0	25.3	26.5	27.2	28.9	29.7	30.6	30.3	30.5	29.9	30.2	30.4	29.5	30.4	30.5	29.3
55-64	21.2	22.2	22.5	22.0	22.3	22.7	24.0	25.6	26.4	27.6	27.5	28.7	28.7	29.6	29.2	29.2	30.2	31.9	30.5

For peer review only - http://bmjopen.bmj.com/site/about/guidelines.xhtml

Table S6b - Female mortality rates (per 100,000 population) by cause, age group and year in the USA

Age group	2001	2002	2003	2004	2005	2006	2007	2008	2009	2010	2011	2012	2013	2014	2015	2016	2017	2018	2019
All-Cause																			
35-44	147.6	148.2	146.9	142.4	142.0	140.4	135.8	133.7	134.0	128.1	129.6	127.9	129.4	133.1	134.6	141.0	142.9	141.6	142.8
45-54	319.8	321.9	322.5	318.1	323.8	320.7	317.2	318.8	319.9	311.7	314.4	311.1	311.6	313.9	313.5	314.1	308.8	301.0	295.1
55-64	773.4	758.0	753.7	724.8	713.0	697.9	677.4	673.1	662.4	647.9	647.4	649.3	653.5	664.3	668.9	675.4	678.4	671.6	667.8
Combined 'Deaths of Despair'																			
35-44	22.0	24.3	24.6	25.4	25.4	26.7	26.9	27.2	26.8	27.7	29.4	28.7	29.7	31.8	34.1	37.6	40.4	39.6	40.5
45-54	26.3	29.3	31.2	33.7	35.1	37.8	40.1	41.0	42.7	42.8	46.6	46.7	47.2	48.6	50.9	51.6	53.4	50.6	51.2
55-64	24.1	24.8	25.7	26.8	27.0	28.8	30.7	31.4	33.3	35.1	37.2	38.6	42.0	45.5	48.2	50.2	51.8	52.5	53.8
Alcohol																			
35-44	6.4	6.1	5.7	5.3	5.4	5.5	5.3	5.5	5.4	5.5	5.7	5.8	5.8	6.3	6.9	7.2	7.6	7.9	8.6
45-54	12.1	12.0	12.1	12.4	12.6	12.8	13.3	13.7	14.3	14.6	16.3	15.9	16.1	16.4	17.6	16.9	17.5	17.3	17.7
55-64	15.8	15.7	15.7	15.7	15.5	15.6	15.7	16.1	17.2	17.3	18.8	19.6	20.4	22.4	23.7	24.0	24.7	25.3	26.7
Drugs																			
35-44	9.2	11.3	12.3	13.0	13.2	14.3	14.3	14.3	13.9	14.7	16.1	15.2	16.2	17.2	18.8	21.8	24.2	23.3	23.6
45-54	7.0	9.8	11.3	12.8	14.4	16.5	18.0	18.3	19.1	19.0	20.5	20.6	21.0	21.5	22.6	24.4	25.5	23.1	23.2
55-64	2.6	3.5	4.2	4.9	5.5	6.5	7.8	8.4	8.8	9.9	10.7	11.0	13.0	14.2	14.8	16.8	18.1	17.6	18.2
Suicide																			
35-44	6.5	6.9	6.7	7.1	6.8	7.0	7.3	7.4	7.4	7.4	7.6	7.8	7.7	8.3	8.4	8.5	8.6	8.4	8.2
45-54	7.2	7.5	7.8	8.5	8.1	8.5	8.8	9.1	9.3	9.2	9.8	10.2	10.1	10.7	10.7	10.3	10.4	10.2	10.3
55-64	5.7	5.6	5.8	6.1	6.0	6.8	7.2	6.9	7.4	7.9	7.7	8.0	8.6	8.9	9.7	9.4	9.0	9.5	8.9

For peer review only - http://bmjopen.bmj.com/site/about/guidelines.xhtml

The RECORD statement – checklist of items, extended from the STROBE statement, that should be reported in observational studies using routinely collected health data.

	Item No.	STROBE items	Location in manuscript where items are reported	RECORD items	Location in manuscript where items are reported
**Title and abstract**					
	1	(a) Indicate the study's design with a commonly used term in the title or the abstract (b) Provide in the abstract an informative and balanced summary of what was done and what was found		RECORD 1.1: The type of data used should be specified in the title or abstract. When possible, the name of the databases used should be included.<br><br>RECORD 1.2: If applicable, the geographic region and timeframe within which the study took place should be reported in the title or abstract.<br><br>RECORD 1.3: If linkage between databases was conducted for the study, this should be clearly stated in the title or abstract.	Title and Abstract- we use publicly available death counts, so not individual level data
**Introduction**					
Background rationale	2	Explain the scientific background and rationale for the investigation being reported			p 1-4
Objectives	3	State specific objectives, including any prespecified hypotheses			p 1-4
**Methods**					
Study Design	4	Present key elements of study design early in the paper			p 5
Setting	5	Describe the setting, locations, and relevant dates, including periods of recruitment, exposure, follow-up, and data collection			p 5

Participants	6	*(a) Cohort study* - Give the eligibility criteria, and the sources and methods of selection of participants. Describe methods of follow-up<br>*Case-control study* - Give the eligibility criteria, and the sources and methods of case ascertainment and control selection. Give the rationale for the choice of cases and controls<br>*Cross-sectional study* - Give the eligibility criteria, and the sources and methods of selection of participants<br><br>*(b) Cohort study* - For matched studies, give matching criteria and number of exposed and unexposed<br>*Case-control study* - For matched studies, give matching criteria and the number of controls per case		RECORD 6.1: The methods of study population selection (such as codes or algorithms used to identify subjects) should be listed in detail. If this is not possible, an explanation should be provided.<br><br>RECORD 6.2: Any validation studies of the codes or algorithms used to select the population should be referenced. If validation was conducted for this study and not published elsewhere, detailed methods and results should be provided.<br><br>RECORD 6.3: If the study involved linkage of databases, consider use of a flow diagram or other graphical display to demonstrate the data linkage process, including the number of individuals with linked data at each stage.	N/A– We use national level death counts by age and sex, no individual records.
Variables	7	Clearly define all outcomes, exposures, predictors, potential confounders, and effect modifiers. Give diagnostic criteria, if applicable.		RECORD 7.1: A complete list of codes and algorithms used to classify exposures, outcomes, confounders, and effect modifiers should be provided. If these cannot be reported, an explanation should be provided.	N/A– descriptive analysis of mortality trends. Full code included and available.
Data sources/ measurement	8	For each variable of interest, give sources of data and details of methods of assessment (measurement).<br>Describe comparability of assessment methods if there is more than one group			

Bias	9	Describe any efforts to address potential sources of bias			
Study size	10	Explain how the study size was arrived at			Full national statistics
Quantitative variables	11	Explain how quantitative variables were handled in the analyses. If applicable, describe which groupings were chosen, and why			
Statistical methods	12	(a) Describe all statistical methods, including those used to control for confounding<br>(b) Describe any methods used to examine subgroups and interactions<br>(c) Explain how missing data were addressed<br>(d) *Cohort study* - If applicable, explain how loss to follow-up was addressed<br>*Case-control study* - If applicable, explain how matching of cases and controls was addressed<br>*Cross-sectional study* - If applicable, describe analytical methods taking account of sampling strategy<br>(e) Describe any sensitivity analyses			N/A- descriptive analysis of national mortality trends, not individual level data.
Data access and cleaning methods		..		RECORD 12.1: Authors should describe the extent to which the investigators had access to the database population used to create the study population.	N/A– publicly available national death counts

For peer review only

				RECORD 12.2: Authors should provide information on the data cleaning methods used in the study.	
Linkage		..		RECORD 12.3: State whether the study included person-level, institutional-level, or other data linkage across two or more databases. The methods of linkage and methods of linkage quality evaluation should be provided.	N/A
**Results**					
Participants	13	(a) Report the numbers of individuals at each stage of the study (*e.g.*, numbers potentially eligible, examined for eligibility, confirmed eligible, included in the study, completing follow-up, and analysed)<br>(b) Give reasons for non-participation at each stage.<br>(c) Consider use of a flow diagram		RECORD 13.1: Describe in detail the selection of the persons included in the study (*i.e.,* study population selection) including filtering based on data quality, data availability and linkage. The selection of included persons can be described in the text and/or by means of the study flow diagram.	N/A– publicly available national death counts
Descriptive data	14	(a) Give characteristics of study participants (*e.g.*, demographic, clinical, social) and information on exposures and potential confounders<br>(b) Indicate the number of participants with missing data for each variable of interest<br>(c) *Cohort study* - summarise follow-up time (*e.g.*, average and total amount)			N/A– publicly available national death counts
Outcome data	15	*Cohort study* - Report numbers of outcome events or summary measures over time<br>*Case-control study* - Report numbers in each exposure			N/A– publicly available national death counts

For peer review only

		category, or summary measures of exposure<br>*Cross-sectional study* - Report numbers of outcome events or summary measures			
Main results	16	(a) Give unadjusted estimates and, if applicable, confounder-adjusted estimates and their precision (e.g., 95% confidence interval). Make clear which confounders were adjusted for and why they were included<br>(b) Report category boundaries when continuous variables were categorized<br>(c) If relevant, consider translating estimates of relative risk into absolute risk for a meaningful time period			N/A– publicly available national death counts
Other analyses	17	Report other analyses done—e.g., analyses of subgroups and interactions, and sensitivity analyses			N/A– publicly available national death counts
**Discussion**					
Key results	18	Summarise key results with reference to study objectives			
Limitations	19	Discuss limitations of the study, taking into account sources of potential bias or imprecision. Discuss both direction and magnitude of any potential bias		RECORD 19.1: Discuss the implications of using data that were not created or collected to answer the specific research question(s). Include discussion of misclassification bias, unmeasured confounding, missing data, and changing eligibility over time, as they pertain to the study being reported.	N/A– publicly available national death counts
Interpretation	20	Give a cautious overall interpretation of results considering objectives,			Page 9-11

For peer review only

		limitations, multiplicity of analyses, results from similar studies, and other relevant evidence			
Generalisability	21	Discuss the generalisability (external validity) of the study results			Page 9-11
**Other Information**					
Funding	22	Give the source of funding and the role of the funders for the present study and, if applicable, for the original study on which the present article is based			Page 2- Funding acknowledgement
Accessibility of protocol, raw data, and programming code		..		RECORD 22.1: Authors should provide information on how to access any supplemental information such as the study protocol, raw data, or programming code.	Page 6– Link to reproducible data and code available

*Reference: Benchimol EI, Smeeth L, Guttmann A, Harron K, Moher D, Petersen I, Sørensen HT, von Elm E, Langan SM, the RECORD Working Committee. The REporting of studies Conducted using Observational Routinely-collected health Data (RECORD) Statement. *PLoS Medicine* 2015; in press.

*Checklist is protected under Creative Commons Attribution (CC BY) license.

For peer review only
